# “Without antibiotics, I cannot treat”: A qualitative study of antibiotic use in Paschim Bardhaman district of West Bengal, India

**DOI:** 10.1371/journal.pone.0219002

**Published:** 2019-06-27

**Authors:** Mohit Nair, Santanu Tripathi, Sumit Mazumdar, Raman Mahajan, Amit Harshana, Alan Pereira, Carolina Jimenez, Debasish Halder, Sakib Burza

**Affiliations:** 1 Medecins Sans Frontieres, New Delhi, India; 2 Calcutta School of Tropical Medicine, Kolkata, India; 3 Centre for Health Economics, University of York, York, United Kingdom; 4 Medecins Sans Frontieres, Barcelona, Spain; 5 Paschim Bardhaman Health District, West Bengal, India; Uppsala University, SWEDEN

## Abstract

**Background:**

Misuse of antibiotics is a well-known driver of antibiotic resistance. Given the decentralized model of the Indian health system and the shortage of allopathic doctors in rural areas, a wide variety of healthcare providers cater to the needs of patients in urban and rural settings. This qualitative study explores the drivers of antibiotic use among formal and informal healthcare providers as well as patients accessing care at primary health centers across Paschim Bardhaman district in West Bengal.

**Materials and methods:**

We conducted 28 semi-structured, in-depth interviews with four groups of healthcare providers (allopathic doctors, informal health providers, nurses, and pharmacy shopkeepers) as well as patients accessing care at primary health centers and hospitals across Paschim Bardhaman district. Qualitative data was analyzed using the framework method in an inductive and deductive manner.

**Results:**

Our results indicate that patients demand antibiotics from healthcare providers and seek the fastest cure possible, which influences the prescription choices of healthcare providers, particularly informal health providers. Many allopathic doctors provide antibiotics without any clinical indication due to inconsistent follow up, lack of testing facilities, risk of secondary infections, and unhygienic living conditions. Pharmaceutical company representatives actively network with informal health providers and formal healthcare providers alike, and regularly visit providers even in remote areas to market newer antibiotics. Allopathic doctors and informal health providers frequently blame the other party for being responsible for antibiotic resistance, and yet both display interdependence in referring patients to one another.

**Conclusions:**

A holistic approach to curbing antibiotic resistance in West Bengal and other parts of India should focus on strengthening the capacity of the existing public health system to deliver on its promises, improving patient education and counseling, and including informal providers and pharmaceutical company representatives in community-level antibiotic stewardship efforts.

## Introduction

Global antibiotic consumption, expressed in defined daily doses (DDDs), increased by 65% from 21.1 billion DDDs to 34.8 billion DDDs between 2000 and 2015, largely driven by rising consumption in low- and middle-income countries, according to a recent study published in Proceedings of the National Academy of Sciences [1]. The World Health Organization (WHO) also issued a warning that the world is entering a "post antibiotic" era where minor infections or injuries, previously manageable with antibiotics, will cost lives [2].

In response to the growing threat of resistance, India adopted the National Action Plan on Antimicrobial Resistance (2017–21) in April 2017, following the WHO’s leadership toward a global action plan on antimicrobial resistance [3]. While the National Action Plan seeks to reduce infections, strengthen surveillance, promote research, and improve rational use of antibiotics, the mechanisms outlined in the plan are yet to be fully implemented, as healthcare systems and budgets vary significantly across states in India. In October 2018, Kerala became the first state to launch a sub-national action plan to contain antibiotic resistance [4].

The Drugs & Cosmetics Act and Rules regulate over-the-counter (OTC) use of antibiotics in India and provide a mandate to identify unlicensed pharmacies, and unqualified medical practitioners [5]. Despite these provisions however, informal health providers (IHPs) who lack formal medical degrees and training in allopathic medicine can be commonly found in rural areas where coverage of primary health centers (PHCs) is more limited. While urban areas have multiple private and public hospitals, rural areas lack access to quality health care. The formal medical system is comprised of a network of sub-centers and PHCs, as well as district and tertiary care hospitals. These hospitals are staffed by formally trained nurses, doctors, and pharmacists. However, in more rural areas where the coverage of PHCs is sub-optimal, unqualified IHPs function as the first point of contact for patients in the community. These providers often lack formal schooling and operate their own clinics after observing medical doctors in practice.

The evidence regarding knowledge, attitudes, and practices (KAP) related to antibiotic use in the Indian setting is limited [6–9], with no published studies from West Bengal. A few studies have attempted to establish the level of knowledge of patients and medical students or healthcare providers, but they do not adequately address the role of IHPs with respect to antibiotic use [10–16]. The WHO recognizes that the “systematic neglect of cultural factors is one of the biggest obstacles to achieving better health outcomes and better standards of living worldwide” [17]. The contextual approach used in this study across multiple users in order to understand prescription and usage choices is one of the added values of this study.

This study took place in Paschim Bardhaman district of West Bengal, which consists of an urban city centre and eight administrative blocks. The Asansol Municipal Corporation manages urban primary healthcare in the city centre. Healthcare in the rest of the subdivision is managed by West Bengal’s Ministry of Health via village sub-centres, PHCs, and block primary health centres (BPHCs). Asansol District Hospital (ADH) is the secondary care referral centre. The total number of registered pharmacies is estimated to be above 300, with a significant number of unregistered pharmacies also present.

The main thematic objectives of the study were:

1. To understand perceptions and vocabularies of illness among patients seeking care
2. To uncover patient expectations from healthcare providers while seeking care
3. To understand knowledge of appropriate antibiotics and perceptions of antibiotic use among formal and informal healthcare providers, and
4. To understand the drivers for various antibiotic prescription choices.

## Materials and methods

This qualitative study was designed as a follow-up to a KAP survey conducted by our team in the same district. The self-administered survey was adapted from a validated tool used in Italy and contained 38 questions. 384 healthcare providers completed the survey, including 96 nurses, 96 medical doctors, 96 pharmacy shopkeepers, and 96 IHPs, who were recruited through convenience sampling. The findings from the survey are published separately in PLOS ONE [18]. We found that doctors had superior knowledge of antibiotic uses and functions in comparison to other groups, but this knowledge did not translate into practice as many prescribed or provided antibiotics for illnesses such as the common cold (which does not require a course of antibiotics). Furthermore, IHPs routinely provided antibiotics to patients who visited their private clinics, despite the fact that only licensed medical doctors are allowed to prescribe antibiotics under Indian law. This qualitative study uncovered some of the drivers for these prescription choices and explored the discrepancies between knowledge and practices among formal and informal health providers through semi-structured, in-depth interviews. Our team purposively sampled providers who participated in the survey until we reached saturation. In addition, we also conducted semi-structured, in-depth interviews with patients seeking care in the outpatient departments (OPD) of PHCs and hospitals in order to understand patient vocabularies of illness and expectations from healthcare providers.

The interview guide was developed based on a literature review and analysis of existing studies from India, as well as consultations with medical doctors and nurses to understand the gaps in the medical literature around this issue. It was pilot tested with 4 medical doctors, 2 nurses, 2 patients, and 2 pharmacy shopkeepers prior to data collection, and revised iteratively as needed. We also consulted senior doctors who had worked on the issue of antibiotic resistance to see if any questions needed to be revised or added. Unlike the quantitative survey, this process was more informal for the qualitative study since the interview guide was designed as a flexible, semi-structured document which could be revised iteratively with each interview as needed.

In total, we purposively sampled 21 providers (6 allopathic doctors, 5 pharmacy shopkeepers, 5 IHPs, and 5 nurses) to take part in in-depth interviews after completing the survey. We were unable to ascertain whether participants were licensed pharmacists, as many shops which dispensed antibiotics were not licensed pharmacies. For the purposes of our study, we were interested in understanding the KAP of individuals who dispensed medications to patients, which is why we have included all these providers together as pharmacy shopkeepers. In addition to healthcare providers, 7 patients were purposively sampled for inclusion in the study based on the following characteristics:

1. >18 years of age
2. Geographical location to garner broad representation
  1. Targeting areas from the OPD of ADH
  2. Targeting areas from PHCs and subcenters in excess of 5 km from the district hospital

In total, we approached 39 participants for in-depth interviews, and 11 participants declined to participate due to lack of time. Ethics approval was obtained from the Medecins Sans Frontieres Ethics Review Board as well as the Clinical Research Ethics Committee of Calcutta School of Tropical Medicine.

### Data collection and analysis

Participants were approached for in-depth interviews until saturation was attained. In-depth interviews were conducted in English, Bengali or Hindi, depending on the preference of the participant. One of the interviewers was a trained qualitative researcher, while the other three were counselors working in information, education, and communication. The three counselors were extensively trained through mock interviews. Interviewers were trained to ask open ended questions using the interview guide, and follow up with probing questions without overtly leading the participant in order to avoid bias. All interviews happened face-to-face and participants were asked to select a convenient, quiet place for the interview. Since the interviews happened face-to-face, all interviewers were also trained to monitor their body language for inadvertent non-verbal cues which may lead the participant and bias the responses. Interviews were digitally audio-recorded and transcribed verbatim before being translated from Bengali or Hindi into English to facilitate analysis. Certain words or phrases which may have been lost in translation (i.e. “halka phulka” antibiotics, which is loosely translated as light or low-power antibiotics) were included verbatim along with a translation. All participants provided written informed consent prior to participating in the interviews, and consented to unrestricted use of all data from the interviews, including quotes, for scientific purposes. In order to ensure confidentiality, any quotes attributed to patients in the following paper have been de-identified, using only basic demographic information that cannot be easily traced back to the participant.

Qualitative data was analyzed using the framework method, a robust and flexible method of summarizing qualitative data across cases and themes [19]. As an initial step to aid familiarization, the research team read and re-read each transcript, before carefully coding each transcript line-by-line. The coding process was both inductive and deductive, as some areas of inquiry (such as the role of informal health providers and pharmaceutical representatives) were predetermined from the literature review, while others emerged inductively from the data through the process of open coding. After reading a few transcripts, an initial coding framework emerged which was subsequently applied to all transcripts. Data which did not fit neatly into the coding framework was included in emergent, in-vivo codes and later reconciled in the framework matrix. The researchers completed this process of coding using NVIVO 11 software, but the final framework matrix was developed in Excel. Data was analyzed separately for formal healthcare providers, IHPs, pharmacy shopkeepers, nurses, and patients and also compared across these categories before being indexed and charted in the framework matrix. Ideally, we would have liked to conduct respondent validation through informal interviews with participants after data collection and analysis was complete in order to assess perceived accuracy of themes. However, this was not possible due to time and resource limitations. Instead, we relied on data triangulation for validation. In addition to analyzing and triangulating data between all respondents and within each respondent group, all four interviewers analyzed the data before arriving on a coding framework in consensus. Hence, data analysis was triangulated between different perspectives in order to minimize coder bias.

## Results

We conducted a total of 28 in-depth interviews (5 pharmacy shopkeepers, 5 informal health providers, 6 allopathic doctors, 5 nurses, and 7 patients accessing care at the OPD setting). All doctors claimed to prescribe antibiotics in their practice: some said it was due to an unhygienic hospital environment, while others said it was due to suspected infections, patient demands, or fears of a “secondary” or superadded infection. The results have been presented in two distinct categories: 1) knowledge, perceptions and practices of healthcare providers; and 2) patient perceptions and expectations from healthcare providers, in keeping with the objectives of the study.

### Knowledge, perceptions and practices of healthcare providers

The following section explores the themes related to objectives 3 and 4, namely knowledge, perceptions and practices related to antibiotic use among healthcare providers.

#### Doctors perceived antibiotics to be a vital part of care and treatment

While some allopathic doctors clearly stated that they refused to prescribe antibiotics without clinical indication, others reiterated that antibiotics are necessary in order to get cured. For cases of simple diarrhea with no blood in the stool, one allopathic doctor mentioned that he routinely included Metronidazole in his prescriptions to facilitate an early recovery:

“*See*, *the difference between viral diarrhoea and bacterial diarrhoea is that viral diarrhoea has more severity*. *The stool comes out like water*, *especially in children… if you give medicine based on diagnosis*, *then things will get worse…first*, *I always write*: *‘use boiled water*.*’ #2*: *I give ORS*. *#3*: *if the patient is going to the latrine 3–5 times*, *then I add Metronidazole*. *Without asking anything else*. *Metronidazole*, *ORS*, *boiled water*. *And I give advice that you boil water before you drink*, *cover the water*, *eat smashed rice*, *etc*.” [Male, 51, allopathic doctor]

Similarly, one allopathic doctor further noted that antibiotics always have a benefit, provided there is no gross misuse:

“*the benefit is always there*, *there’s no harm from antibiotics…but if you use the same drug over and over again*, *resistance will develop and then the harm factor comes into play*” [Male, 28, allopathic doctor].

If the condition appears life threatening in any way, doctors prescribed antibiotics without hesitation and said they were necessary to get cured. Similar views were expressed by nurses, pharmacy shopkeepers, and IHPs: one IHP even stated that he disbursed antibiotics to every single patient who visited him. IHPs felt it was an important component for care and treatment even for patients presenting with fever symptoms:

“*if [the] patient is coming with fever [since] 2 or 3 days*, *and he [has] already taken Paracetamol*, *so definitely we should apply antibiotics- without antibiotics*, *it cannot be cured*” [Male, 39, IHP].

#### Informal providers prescribed antibiotics in order to retain patients

When asked why antibiotics were disbursed so readily, IHPs indicated that patients self-medicate frequently these days, and they can only fully recover if antibiotics are provided.

“*In this [day and] age*, *there are a lot of medicines [that] patients eat at home before coming here…and after that*, *if he comes to the doctor*, *[that] means he hasn’t gotten better*, *[and] we have to apply antibiotic to them*. *Only then can they full recover*” [Male, 39, IHP].

There was a strong desire to retain the patient. IHPs perceived that patients demanded the fastest cure possible or switched providers, so antibiotics were seen as a necessary part of the treatment regimen:

“*everyone wishes that the patient will come to them more…suppose we say the patient will not be cured*, *he will right away go to another practitioner*. *It’s better that we give antibiotics and control the disease*. *Without antibiotics*, *I cannot treat*” [Male, 45, IHP].

#### Perceived demands for “quick cures” influenced the use of antibiotics

Pharmacy shopkeepers were routinely pressured by patients to provide antibiotics:

*“first [patients] will tell you to prescribe the medicine that can cure me quickly…they tell to give good medicine for quick recovery*. *This is not only the scenario [in] my place*. *This happens everywhere…we can start with Cifran*, *but Ampiclox works quickly*. *The patient does not have any patience*” [Male, 48, pharmacy shopkeeper].

IHPs similar perceived that patients demand shorter courses of treatment:

“*we tell them that they should take the antibiotic for around 2–3 days*, *but then the patient says ‘no*, *we just want a one-day course*’” [Male, 24, IHP].

Some nurses expressed surprise at how well patients could articulate certain names of medicines, despite their lack of knowledge around its uses and functions.

“*They are community people*, *so maybe they heard from someone*. *They heard stories like ‘I took this*, *I took that*,*’ and with that*, *they say the name*. *We feel shocked how nicely they say the name*” [Female, 35, nurse].

Nurses reiterated that patients were habituated to quick alleviation of symptoms from antibiotics purchased at medical shops, and demanded such antibiotics without adequate understanding of side effects, risks or uses. There was a widespread perception of patients being stubborn, illiterate and lacking in knowledge of antibiotics, illnesses or appropriate points of contact for care seeking, which made counselling even more difficult.

“*They [patients] don’t know much about antibiotics…there is a course of antibiotics which they don’t complete- after taking 2–4 [tablets]*, *they think it is done*” [Female, 53, nurse].

#### Antibiotics were prescribed to prevent secondary (superadded) infections

Doctors frequently mentioned that they prescribed antibiotics as a precautionary measure, which was linked to other factors such as unhygienic conditions at the patient’s home, in addition to poor sanitation at health facility levels, lack of follow up, and diagnostic uncertainty.

“*I give antibiotics because I think my hospital…is not so sterile [hygienic]*. *So*, *I have to give an antibiotic*, *otherwise there are chances of an infection*” [Male, 60, allopathic doctor].

While some doctors reported only resorting to antibiotics after other drugs had failed, several others claimed they regularly prescribed an antibiotic as a precautionary measure:

“*In viral [infections]*, *there is no role for an antibiotic*. *Okay*? *But even then*, *we sometimes give an antibiotic*. *Ask me why…because in a viral infection*, *the antibodies are prepared in the body*, *but the viral [infection] is damaging your tissues*. *After it damages you*, *there is a secondary infection…there can be a bacterial infection*. *So to combat this secondary infection*, *we add [an] antibiotic*” [Male, 51, allopathic doctor].

#### Lack of adequate testing facilities contributed to “empirical” treatment

Most doctors reported prescribing antibiotics without conducting culture or sensitivity tests, either due to lack of adequate laboratory facilities or time:

“*usually*, *I believe on my clinical findings simply*. *If I see that there is some infection*, *I will usually give the empirical antibiotic”* [Male, 60, allopathic doctor].

Doctors at the PHC level typically examined at least 80–100 patients per day, and many reported that it was practically impossible to conduct lab tests before deciding to prescribe antibiotics on a patient-by-patient basis. As a result, they often engaged in “empirical” treatment, which is treatment based on clinical experience alone in the absence of complete information. In some cases, doctors had existing relationships with private labs or were part of a private-public partnership; they referred patients for diagnostic testing to these labs, but it was not clear how many patients returned with lab results for follow-up:

“*for the patients*, *we have [a] lab out here*. *We have got government rates…that functions in public-private partnership mode*, *so we send there”* [Male, 31, allopathic doctor].

Doctors preferred “empirical treatment” based on an assessment of symptoms. They prescribed basic medicines like paracetamol for fevers or salbutamol for coughs, before proceeding to amoxicillin, ampicillin, cloxacillin, and other antibiotics if the condition did not improve:

“*we first give you simple treatment*, *like I give you paracetamol if you are getting a fever*, *salbutamol for a cough…after that*, *I say to gargle*, *and if he doesn’t get better even then*, *then I give antibiotics*. *And in that antibiotic*, *Amoxicillin is first*. *Amoxicillin*, *ampicillin*, *cloxacillin*” [Male, 51, allopathic doctor].

Even in cases where labs did exist, one doctor mentioned he questioned the authenticity of the reports as he found different reports for the same patients from some labs:

“*I personally won’t trust the report of the culture-sensitivity over here…because [in a] few instances*, *I found different reports from them”* [Male, 31, allopathic doctor].

In most areas, we found that government doctors were working in conditions with highly limited lab support:

“*it’s easier to say what is not available*. *Cultures and sputum tests are not available here*, *and 24 hour lab support is not there* …*people*, *as well as other resources*, *are not available*. *Only total counts and urine are done here*. *With urine*, *a culture is also not done*, *and this is a basic need where pediatric practice is concerned…there are a lot of limitations here at present*” [Male, 31, pediatric doctor].

#### Pervasive influence of pharmacuetical company representatives

Another salient theme which emerged from our interviews was the strong desire to acquire new knowledge, among formal and informal health providers alike. Pharmaceutical company representatives (PCRs), in particular, were seen as a key source of new knowledge, as many IHPs attended meetings and seminars organized by PCRs:

“*big companies like Cipla*, *Ranbaxy invite me and the speaker is a big doctor…if there is a new medicine*, *we get to know from PCRs*. *If PCRs don’t come*, *then how will we know*?” [Male, 38, IHP].

Similarly, many doctors interacted with PCRs in order to get information about newer drugs on the market. Doctors recognized that PCRs often contributed to increasing resistance by demanding doctors prescribe their antibiotics: as one BPHC doctor lamented,

“*he [PCR] doesn’t even know the use of clavulanic acid*, *but he dictates the doctor…because he works for a reputed 100-year-old multinational company*, *he thinks he has the right to dictate the doctor*!” [Male, 31, allopathic doctor].

It was also commonly noted among allopathic doctors that less than half the PCRs visiting them provided any real evidence for use of the drugs that were being recommended, even when doctors specifically asked for such clinical evidence:

“*only about 40 to 45% [of PCRs] provide evidence- real evidence*. *Even if you ask repeatedly*, *only about 50% willc ome up with with [something]…either that or they don’t turn up next time”* [Male, 31, pediatric doctor].

When asked about incentives presented by pharmaceutical company representatives, no doctors admitted to receiving any incentives of any kind.

We found that PCRs tapped into a very strong network of IHPs and regularly organized meetings and seminars:

“*like there is Frnaco India…these people like MR [medical representatives or PCRs] come and visit*. *What they do- they choose 10–20 doctors…and do a get-together…some companies also say…let us do a program in this hotel”* [Male, 45, IHP].

These meetings typically hosted up to 40–50 people at a time either at a hotel, district library or a central location such as a park, and were organized by the regional association of IHPs. In addition, most IHPs relied on networks and referrals to sustain their practice, and acquired new knowledge through on-the-job training and the information provided by PCRs visiting their practice. In general, IHPs viewed PCRs as a very helpful source of information regarding antibiotics and perceived their visits positively.

#### Erosion of trust in doctor-patient relationship

Doctors routinely reported that they were worried about being blamed for faulty treatment by patients.

“*Mischevious people…make the matter worse…they will bring 10 people and stand here*. *They create chaos*, *and say the doctor has killed someone*. *Why has he killed the patient*?” [Male, 51, allopathic doctor].

Multiple doctors expressed fear for their lives, given the threats to personal well-being from angry patients. Some doctors also blamed the media:

“*one of the faults is also with the media…they create such a notion [that] doctors are…looting the patients*, *or they are only [here] for earning money from the patient*. *They are extracting so much from the patient*” [Male, 31, allopathic doctor].

Doctors in the government setup are usually given a non-practicing allowance, in order for them to focus on their duties in the government setup without the distractions of private practice. However, more than one doctor reported taking the non-practicing allowance and operating a private practice anyway. During the course of an interview, one doctor mentioned he was losing several patients worth Rs. 250 each for every minute he was sitting here in the government setup.

“*As you can see*, *I am working and I work very fast…I also have a chamber [private practice]…if I did this much work in my chamber*, *I would get 250 rupees per patient*, *OK*? *… I don’t know how many 250-rupee patients I am losing right now*” [Male, 51, allopathic doctor].

He took pride in his ability to take no more than 5–10 seconds per patient in the government setup, but claimed the same patient would be given 20–30 minutes in his private clinic. During our field observations, we observed another doctor referring his government patients to his own private clinic, claiming that they would receive much better care and medicines at his private clinic.

We also frequently noticed that formal and informal health providers alike would doubt patients and wonder whether they were actually in pain.

“*By calling him after 3 days*, *the benefit for us is that we can have a follow-up and see how the patient’s response was*. *And how much his need is*. *If he’s really in pain*, *then he will definintely come*. *And if he’s just faking it*, *then he won’t come again*” [Male, 51, allopathic doctor].

#### Doctors routinely prescribed longer antibiotic courses in blocks of 3 days due to perceived lack of treatment adherence and poor follow-up of patients

Given poor follow up and a perceived lack of patient adherence to treatment, many doctors preferred to prescribe a 3-day course of antibiotics for patients in order to improve the likelihood of the patient returning for follow up, irrespective of the required treatment duration. Doctors easily identified the need to complete the full course of antibiotics to prevent antibiotic resistance, but many also felt the field reality dictated a shorter course of antibiotics to better gauge patient response to treatment:

“*We actually prescribe [antibiotics] for three days*. *We’ve got two intentions for that- one is after three days*, *the patient will come back to get the medicine*, *and we will know whether the medicine is responding or not*. *Another thing is- if you give a lot of medicine at once*, *the patient does not know what to do with all the medicine and throws it away…so*, *I ask my patients to take for 3 days*, *come after 3 days to show me*, *and then take for another 3 days*” [Male, 31, allopathic doctor].

Another doctor mentioned:

“*if I gave him 7 days of medicine*, *then it’s necessary that he will eat it all…in the beginning*, *I used to prescribe for 5–7 days…they stopped eating it after 3 days*” [Male, 51 allopathic doctor].

#### Task shifting related to antibiotic prescriptions

At the PHC level, there is often a severe shortage of doctors, which puts the burden squarely on the nurses or doctors on deputation who are in charge of more than one PHC. As a result, nurses informed us that they handle the OPD and give antibiotics in the absence of a doctor:

“*whatever the patients say about signs and symptoms*, *then…based on my experience seeing the doctor giving and by searching from internet*, *I give antibiotics*” [Female, 35, nurse].

A few providers confirmed this by suggesting that, in some areas where doctors were lacking, the government had permitted nurses to provide antibiotics.

“*In some PHCs*, *we have sisters [nurses] who have been treating basic pneumonias…who have been given permission from the government’s side to treat pneumonia before they access the doctor…such things can be done more frequently*, *rather than giving it in the hands of these unqualified practitioners*” [Male, 31, allopathic doctor].

Furthermore, pharmacists in the government setup often assumed the role of dispensing medications unofficially.

“*I can do the treatment for small illnesses… in that case*, *we don’t need permission…if it is [a] simple allergic case like cough*, *cold*, *runny nose and if it is symptomatic*, *then I wait for 2–3 days*. *If it is not [getting cured] by anti-allergic drugs like Cetirizine*, *then in that case*, *I start an antibiotic*” [Male, 48, pharmacist].

#### Healthcare providers blamed one another for the problem of antibiotic resistance

Among the different groups of participants, blame shifting was a very common phenomenon, where the doctors blamed informal health providers and pharmacy shopkeepers, and the informal health providers blamed allopathic doctors for antibiotic resistance. One IHP exclaimed during the interview:

“*What you are seeing now is that allopathic doctors are injecting a 1 year old child with 250 injections…after seeing the prescriptions*, *I am able to see the amount of powerful antibiotics that are being prescribed*” [Male, 48, IHP].

We frequently noticed denial of any personal responsibility from IHPs:

“*to stop this*, *only the allopathic doctors can do something*! *What can we do about this*? *Chemist shop owners also cannot do anything*. *Whatever the doctor writes*, *whatever is on the prescription*, *they give out*” [Male, 48, IHP].

Doctors, on the other hand, expressed frustration at the lack of legal action against “quacks” and over-the-counter use of antibiotics:

“*Raniganj is full of quacks*. *Everywhere you go*, *you will find quacks”* [Male, 31, allopathic doctor].

They claimed that patients were often resistant to a multitude of antibiotics, which left them with no choice but to resort to fixed dose combinations (FDCs) of antibiotics or other powerful antibiotics:

“*in this area*, *quack is there*. *So they’re using antibiotics…I’ll have to use a antibiotic or higher derivatives*. *Otherwise*, *the patient will not respond- because they are resistant”* [Male, 60, allopathic doctor].

This was also linked to difficulties in counselling patients- many doctors felt patients were less patient these days and simply desired the fastest cure possible without any hindrance. As a result, doctors often felt pressured to prescribe multiple medicines, such as Rantac, Albendazole, B vitamins, and other medicines which may not be directly relevant to the patient’s condition, but would not necessarily harm the patient either.

#### Halka-phulka antibiotics [light or low-power antibiotics] were perceived as first aid treatment by IHPs

We commonly observed dissonance between attitudes and practices among IHPs, who claimed that antibiotics should be given if other medicines do not work on the one hand, but simultaneously disbursed multiple antibiotics in our presence with no clinical indication. FDCs of antibiotics were referred to as “double antibiotics” or “high antibiotics,” and were disbursed in cases where diseases were unresponsive to a single antibiotic. IHPs routinely perceived antibiotics as first aid treatment:

“*if the patient is having excessive loose motion*, *I give 1–2 O2 [Ofloxacin-Ornidazole] tablets or something like that as first aid…otherwise patient doesn’t even come*” [Male, 45, IHP].

Most providers referred to these “first aid” antibiotics as halka-phulka antibiotics:

“*I give Paracetemol [for a fever patient]- I will have to give a halka phulka antibiotic along with that- ampicillin*, *amoxicillin I will have to give*” [Male, 48, IHP].

IHPs reported that most patients arrived at their clinics with minor illnesses, such as cold, cough, fever, vomiting, and stomach pain or watery loose stools mostly. Such illnesses do not typically require antibiotics for treatment.

#### IHPs perceived their work was appreciated by medical doctors

IHPs reported a strong relationship between informal and formal health providers: one IHP informed us that doctors often referred patients to him to administer particular injections or provide basic first aid, even as other doctors lamented the lack of legal action against IHPs:

“*There are lots of doctors in the market*, *so we send the patients to XYZ doctor…when they ask [the patient] who has sent you*, *they tell our name and the doctor comes to know that there is a doctor [IHP] who is practicing*. *If a patient goes to any surgeon doctor…if [they] write 10 injections*, *they also instruct the patient to visit me to push it [administer the injection]*. *So*, *in that way*, *patients also get help and we also earn Rs*. *5–10 for injecting*, *so our work is also done*. *It is helpful for the patient that they don’t need to go here and there- their work is done at [one] place and if [they] come to me*, *I push the injection*” [Male, 38, IHP].

Furthermore, most IHPs operated in areas that lacked medical staff and healthcare facilities. IHPs often functioned as the first point of contact for patients before referring them to particular private clinicians whom they held established relationships with. Many IHPs currently working in the field learned from doctors or nurses in private hospitals for a few years, prior to opening up their own clinic. As a result, most informal health providers had high self-confidence in their abilities and felt they were a crucial part of the medical system.

“*Doctors are…many of them are happy with us…in the night*, *we are the ones practicing in village after village*. *The doctor will give his time*, *visit*, *and then leave…the doctors support us as well*” [Male, 45, IHP].

#### Antibiotic choice is not guided by evidence

Doctors in the government setup generally claimed to prefer prescribing antibiotics which were available in that particular facility:

“*whatever medicine is available here*, *I write…first*. *If that doesn’t work*, *then I prescribe from outside*, *which is more expensive”* [Male, 51, allopathic doctor].

Amoxicillin, Azithromycin, Ciprofloxacin, Penicillin, Cotrimoxazole, Doxycycline, Norfloxacin, Ofloxacin, Levofloxacin, and Metronidazole were commonly available and prescribed as a result. Metronidazole was a very common prescription for diarrheal patients, whereas fever patients or patients presenting with a cough and cold were usually given Paracetamol, before being given any antibiotics:

“*for cough*, *I give a Paracetamol…if he doesn’t get better and says he still has a cough*, *then I prescribe antibiotics”* [Male, 51, allopathic doctor].

Generally, doctors voiced support for monotherapy over FDCs of antibiotics, claiming that FDCs were unnecessary for the patient and could lead to resistance:

“*monotherapy is better than combination therapy obviously…suppose one patient comes to you with a cold…and you prescribe him or her a medicine that contains 4 or 5 drugs*, *but actually*, *he needs 1 or 2 medicines*. *That is sufficient for her symptoms to subside”* [Male, 31, allopathic doctor].

When doctors resorted to FDCs however, they usually prescribed medicines from outside the facility, with the patients bearing the costs of the medicines themselves. Since generic medicines were provided as monotherapy in the government setup, one doctor mentioned combining 3–4 drugs at the same time, especially given poor perceptions of treatment adherence among patients:

“*I have to combine a lot of things…to get the cure*, *to get the result*, *because here the people will see the result*. *They won’t take the entire duration of the treatment*” [Male, 28, allopathic doctor].

Furthermore, there was a widespread perception of lower quality of generic medicines in the government health setup among medical doctors. Brand-name medicines were assumed to be of higher quality and made patients recover more quickly:

“*you take the generic medicine*. *Maybe you get cured in 3–5 days…but the quality [of brand-name medicines] gives you a better treatment…you are getting cured within hours or something”* [Male, 28, allopathic doctor].

However, one doctor did state that generic medicines were more affordable and noted that brand names were only perceived to be of better quality due to marketing by PCRs:

“*a brand-name company has to invest at least 200% of the manufacturing charges of the drug for marketing*, *but generic doesn’t have that situation*. *Along with that*, *another thing I heard from one friend is that if brand medicines get expired*, *the company takes it back…but for a generic medicine*, *if it gets expired*, *it is the shopkeeper’s loss*” [Male, 31, allopathic doctor].

Some doctors even felt concerned about the regulations enforced upon doctors-

“*[rather than] compelling the doctor to [prescribe] generic medicines*, *the government should impose price controls on the medicines*” [Male, 31, allopathic doctor].

#### Knowledge of antibiotics and antibiotic resistance varies widely

When asked what were the various causes of antibiotic resistance, most doctors immediately identified incorrect duration, incorrect dose, lack of communication between doctors in transitions of care, and self-medication by patients as causes. In addition, doctors understood that resistance was a naturally occuring phenomenon:

“*whenever a bacteria will take the antibiotic*, *any person’s chances of resistance will increase*. *You are consuming antibiotics for no reason*, *so the bacteria mutates and changes…the more you prescribe antibiotics*, *the more the resistance develop*” [Male, 51, allopathic doctor].

Additionally, doctors mentioned that inadequate sanitation resulted in a dependence on antibiotics rather than focusing on prevention.

Among pharmacy shopkeepers and informal health providers, FDCs and “bhari-barkam” [heavy-duty or powerful] antibiotics were seen as being more prone to antibiotic resistance, and yet antibiotics were frequently dispensed to prevent other illnesses or multiple antibiotics were combined individually to create combinations of antibiotics. Some pharmacy shopkeepers had no idea what caused antibiotic resistance and attributed “bad weather” as a causal factor:

“*this much I know*. *Something related to weather leads to antibiotic resistance*” [Male, 43, pharmacy shopkeeper].

Despite the dissonance between knowledge, attitudes, and practices, pharmacy shopkeepers did believe antibiotic resistance posed a major threat in the future:

“*those who are sitting in the chemist shops- if [they] are not guided*, *then in the future*, *it will be more difficult*. *Today*, *AmoxiClav is working on me*, *but maybe tomorrow it will not*. *That is very bad*, *very dangerous*. *Nowadays*, *[this is] what is happening in most of the chemist shops*” [Male, 31, pharmacy shopkeeper].

### Patient perceptions and expectations from healthcare providers

#### Vocabularies of illness among patients and limited knowledge of antibiotics

Patients usually named environmental factors as being a cause of illness:

“*water…does not come clean*. *That’s why we fall sick*. *Also*, *the population is so big*, *and there is pollution in the cities- it happens because of this*. *There are many reasons*, *but mostly*, *it’s because of water*, *changing seasons*, *like this*” [Male, 31, patient].

It was further understood that illness could be seasonal, even if the cause was unknown:

“*whoever has an illness will have it*. *This happens normally once or twice in the year on its own*” [Male, 31, patient].

Finally, one patient also identified negligence as a factor:

“*the carelessness we have*, *that is the reason for the illness*. *Not paying attention to cleanliness*, *not paying attention to a small illness when it occurs…all this*” [Male, 28, patient].

In general, patients had no conception of what constituted an antibiotic. Patients appeared to draw distinctions between halka-phulka [light or lower-power] medicines and more powerful medicines, and some even associated the name “antibiotic” with a faster cure, but there was no knowledge of functions and uses:

“*antibiotic means for cold*, *cough*, *fever…all this*” [Male, 38, patient].

Overall, patients had a very poor understanding of antibiotic resistance or associated causes.

#### Patients switched medical providers frequently in order to seek the fastest cure

Patients switched health providers frequently if the treatment was perceived as being too slow or ineffective. In addition, patients did not hesitate to approach a combination of Ayurvedic (traditional medicine), homeopathic and allopathic doctors- the underlying motivation was the best cure in the fastest amount of time. Some patients used Ayurvedic products such as “Chawanprash” (herbal paste), but preferred allopathic medicines over homeopathic treatments, as they were perceived to cure the disease more quickly. If the prescribed treatment was not working however, patients did not hesitate to switch providers:

“*I [went] to the first doctor but didn’t get cured*. *Then*, *I [went] to another doctor and [took] 2–3 medicines*, *but I still [was not] cured*. *Again*, *I [went] to another doctor and [took] medicine for 3 months*, *but my condition didn’t improve*. *Finally*, *if the medicine of the next doctor works for me*, *then I go to that doctor every time*” [Male, 50, patient].

IHPs also perceived that patients frequently switched health providers in an effort to seek the fastest cure possible- in order to retain the patient, informal health providers constantly sought feedback and gave in to perceived patient demands for antibiotics:

“*In today’s age*, *if a patient comes*, *he does not even wait 2–4 days with one doctor*. *He wants quick relief* …*he changes doctors 2–3 times*. *If he does not get cured in one day*, *he will not come back to me tomorrow; he will go to a second doctor*. *If the second doctor does not cure within 2 days*, *he will go to a third doctor*. *He changes doctors 4–5 times- in today’s day and age*, *the patient does not have any patience*” [Male, 39, IHP]

#### Patients’ unmet expectations from healthcare providers

Patients who sought treatment expected to be treated with respect and provided more than a minute or two for consultation. One young mother informed us that she wished the doctor would actually touch and see if something was wrong, rather than simply prescribing medicines quickly based on his visual judgment. Patients noted that doctors, especially private practitioners, treated them more like business customers rather than patients. The same doctors who would hesitate to spend 5 minutes with a patient in a government setting would spend much longer observing the patient in private practice, but they would also prescribe more medicines and tests, which occasionally made patients suspicious of the motives behind such decisions:

“*if you come once*, *then you have to go again…the bigger doctors outside- they have high fees*, *and nowadays it feels like they make a customer out of us*” [Male, 31, patient].

#### Government health facilities were perceived as being inadequate

Patients confirmed what doctors, nurses, IHPs, and pharmacy shopkeepers already stated in terms of the government healthcare system being the last resort for patients. Patients preferred to reach out to IHPs who sat directly in their communities or self-medicate with over-the-counter “halka phulka medicines” [light or low power medicines] at chemist shops wherever possible.

When asked why the government health system was seen as a last resort, patients usually cited long wait times and lack of adherence to work timings on the part of doctors. However, patients perceived this as a normal associated cost of free care:

“*where you go without money*, *you will have to bear a little trouble*” [Male, 38, patient].

Additionally, there was a perception of better treatment if recipients were well connected politically:

“*here*, *there is still this thing with the local politicians*. *If you are referred by someone*, *then you will be treated well*. *If you’ve just come like that*, *then you’ll be treated like normal*” [Male, 31, patient].

As a result, if patients perceived the problem to be relatively minor, such as a cold, cough, or watery loose stools, they preferred to visit a pharmacy shop or a local IHP.

Complaints about the public health setup came from all stakeholders and ranged from unhygienic conditions at the facility level to severe staff shortages to inadequate roads in and around PHCs.

“*See I don’t cut or do anything here*. *The setup is lacking*. *I actually come here on deputation*. *There is no Medical Officer here*. *I was told to come for 6 months*, *and I have been doing it continuously for the past 2 years…the road here is so bad*, *so the silencer in my car broke*. *The Government tells us that doctors should come on time- do this*, *and do that- but for that*, *they will have to see the other facilities too*, *right*? *They have to see the roads and reduce the influence of politics*” [Male, 51, allopathic doctor].

Overall, all five groups of participants suggested numerous strategies to counter the threat of antibiotic resistance. Counseling of patients featured prominently among these strategies, along with encouraging patients to complete the full course of antibiotics:

“*public awareness is needed…make them aware that [they should] only use antibiotics when it is needed*. *Don’t take antibiotics by yourself”* [Male, 51, allopathic doctor].

Strict legal action was suggested by doctors, nurses and a few pharmacy shopkeepers, especially in terms of banning OTC use of antibiotics and taking action against informal health providers. Additionally, continued medical education, trainings, workshops, and seminars were all suggested as ways to improve knowledge around antibiotic use in the medical community. All doctors who were interviewed reported attending at least some events organized by their medical colleges in their vicinity, and expressed enthusiasm for the role of continued medical education:

“*[continued medical education] is very important*! *This is very important not only for antibiotic resistance*, *but also [to cover] basic topics…until and unless your basics are clear*, *your treatment is all in vain*” [Male, 31, allopathic doctor].

## Discussion

Our study analyzed data from semi-structured interviews with formal and informal healthcare providers as well as patients seeking care to understand knowledge, attitudes, perceptions, and practices related to antibiotic use in a particular district of West Bengal. While other studies have similarly reported poor knowledge of antibiotics among patients and the general community in multiple settings [20–24], we could not find any studies from this region which triangulate these findings with perceptions among healthcare providers. Other studies from India in the literature primarily focus on surveying medical students or clinicians at the tertiary level, without effectively taking into account the role of IHPs and PCRs [7–10, 12–16].

Our findings demonstrated that perceived demands for antibiotics influenced prescription behavior, particularly among IHPs. Moreover, healthcare providers conceded that PCRs played a critical role in influencing the behavior. This has been reported across multiple countries and contexts: Workneh et al found that approximately half of the medical doctors sampled from and working in Northern Ethiopia reported that their prescription decisions were influenced by PCRs in the last year [25]. Similar results have been reported from Yemen, Pakistan, Peru, Saudi Arabia [26–28], as well as developed countries such as Germany [29]. Lieb and Scheurich reported that doctors who saw PCRs frequently in Bavaria, Germany had significantly higher total prescriptions and total daily doses [29]. In India, Waheed et al found that tangible rewards for doctors by PCRs led to prescription loyalty and Kothani et al found that newer antibiotics were used on recommendations made by PCRs who often relied on skewed studies [30–31]. However, virtually no studies examined the links between PCRs and IHPs.

We found that IHPs do not just provide care or antibiotics to patients; they are also well-organized and operate within active networks across multiple blocks in the district. IHPs reported that PCRs often leveraged these networks to market new antibiotics and organized conferences for IHPs, given their desire to acquire knowledge. This has not been well-documented in the region and presents promising avenues for further research and investigation.

Current initiatives to tackle ABR focus on implementing antibiotic stewardship at the hospital level, setting up surveillance systems, restricting access to over-the-counter antibiotics, and generating awareness among consumers. However, these networks of IHPs and PCRs are largely left out of these initiatives. Some studies examined the roles occupied by informal health providers in the community: the findings generally indicated poor quality of service provision, gaps in knowledge, and mixed results regarding efforts to retrain them [32–37]. Future research should examine the long-term impacts of such training and assess how IHPs and PCRs can be influenced to promote a more responsible message around antibiotic use. Four pharmaceutical companies, including Glaxo-Smith-Kline and Pfizer, recently reported separating pay from antibiotic sales volume for all sales staff, and similar initiatives can be implemented and rigorously assessed for national pharmaceutical firms in India [38].

More broadly, the reliance on IHPs reflects a lack of trust in the existing government health system as reported by patients in our study. In order to alleviate the lacunae in the public health system, policy makers must invest in improving existing infrastructure and increasing the presence of allopathic doctors at PHCs. Our study found that doctors lacked the resources to conduct culture investigations prior to prescribing antibiotics in many settings, which exemplifies how shortfalls in healthcare delivery can contribute to irrational use of antibiotics and subsequent antibiotic resistance.

We argue that improving the capacity of the existing public health system to deliver on its promises can indirectly influence antibiotic prescription decisions and form an important component of curbing antibiotic resistance. While the short-term risks posed by the lack of awareness around antibiotic uses and functions are largely self-evident, there is also a larger long-term risk at play. Our study uncovered a fraught relationship between doctors and patients, where doctors feared physical assault or worse if the treatment did not go as expected, and patients claimed doctors treated them more like business customers. This erosion of trust in the doctor-patient relationship is exacerbated by the lack of resources in the public health system and the unwillingness of doctors to shun private practice for government service. Strengthening the existing system is likely to go a long way in reducing the reliance of patients on informal health providers. Balancing the demands between access to and overuse of antibiotics remains a major challenge for policy makers at present.

### Methodological considerations

It must be noted that while we attained saturation in the study as a whole, we did not attain saturation in each analytical sub-category (i.e. allopathic doctors, pharmacy shopkeepers, etc.) which must be explored in future studies. Furthermore, we were only able to include patients accessing care at health facilities. Future studies must look to also include patients who are unable to seek care. It is quite possible the care-seeking behavior of this segment of the population differs from patients accessing care at the hospital or PHCs.

## Conclusions

Efforts to curb antibiotic resistance in West Bengal and other parts of India require a holistic approach, including but not limited to strengthening the capacity of the existing public health system to deliver on its promises, improving patient education and counseling, and including IHPs and PCRs in community-level antibiotic stewardship efforts.

## Supporting information

S1 File Interview guides. (PDF)

S2 File Transcripts. (ZIP)

S3 File English questionnaire. (PDF)

S4 File Bengali questionnaire. (PDF)

## Abbreviations

ABR: Antibiotic Resistance
ADH: Asansol District Hospital
ANM: Auxiliary Nurse Midwife
AYUSH: Ayurveda, Yoga and Naturopathy, Unani, Siddha and Homeopathy
BPHC: Block Primary Health Centre
CMOH: Chief Medical Officer of Health
DDD: Defined Daily Doses
ERB: Ethical Review Board
FDC: Fixed Dose Combinations
IEC: Information Education Communication
IHP: Informal Health Provider
KAP: Knowledge, Attitudes, Practices
MOH: Ministry of Health
MRSA: Methicillin Resistant Staphylococcus Aureus
MSF: Medecins Sans Frontieres
OPD: Out-patient Department
OTC: Over-the-counter
PCR: Pharmaceutical Company Representatives
PHC: Primary Health Centre
WHO: World Health Organization
