# Supplementary material for: “Without antibiotics, I cannot treat”: A qualitative study of antibiotic use in Paschim Bardhaman district of West Bengal, India
Source: PLoS One. 2019 Jun 27;14(6):e0219002. doi: 10.1371/journal.pone.0219002 (PMC6597109; doi:10.1371/journal.pone.0219002)
Supplement: S1 File — (PDF) [file pone.0219002.s001.pdf]

IN-DEPTH INTERVIEW GUIDE

Formal (allopathic) doctors + informal (non-allopathic) practitioners

1. Describe your typical OPD setting:
    a. Patient load?
    b. Time per consultation?
    c. Are you typically the only doctor?
2. How would you describe your prescription patterns?
    a. Describe a typical patient visit, diagnosis, and prescription.
    b. Do you use generic names or brand names?
    c. Do you prefer monotherapy or combination drugs?
    d. How often do you use broad spectrum antibiotics?
        i. For what conditions?
3. How often do you encounter common skin problems?
    a. What do you prescribe?
    b. How often do you prescribe topical antibiotics?
4. How often do you perform minor surgical procedures (i.e. suturing, nail evulsions, cyst removals, etc.)?
    a. Do you prescribe antibiotics? Are they indicated?
5. What is the availability of microbiological testing?
    a. Do you have a microbiological testing facility?
    b. Do you use microbiological testing in your practice?
        i. Specifically, culture and sensitivity?
6. How do you treat upper respiratory tract infections and diarrheal illnesses?
    a. Have you confirmed with sputum or stool routine cultures before prescribing?
    b. *For this scenario, confirm if use of antibiotics is appropriate*. *If used, name the commonly used antibiotics.*
7. How frequently do you prescribe antibiotics?
    a. For which illnesses or ailments?
8. Explain your decision making process when prescribing antibiotics:
    a. What factors drive this decision? [Is cost a factor?]
    b. How do diagnoses factor in? How about diagnostic uncertainty?
    c. Do you have an in-house pharmacy?
        i. If so, do you prefer to prescribe drugs available at the pharmacy?
9. How much time do you take to council the patient?
    a. How does this affect your prescription decisions? Is there enough time?
10. What do you do if a patient asks you for an antibiotic if it's not necessary?
    a. How do you think patients perceive doctors who don't readily prescribe antibiotics?
        i. Incompetence?
11. How are patients involved in the prescribing process?
    a. How do you manage patients when they:
        i. Take improper dosage?

ii. Can't afford treatment?
iii. Self-medicate with over the counter medicines?
iv. Go to several doctors for their needs?

b. What are your thoughts on delayed prescription practice?
    i. For example, what would you do if you feel an antibiotic is not required immediately, but a patient lives far away and/or you are unsure of follow-up?

12. **If you practice alternative medicine (homeopathy, Ayurveda):** how often, if at all, do you prescribe antibiotics?
    a. Do you consider antibiotics to be allopathic medication?
13. How do you weigh the risks and benefits of prescribing antibiotics?
    a. Can you describe this process? Do you do it for every patient?
    b. What else drives your decisions?
14. How frequently do you come across patients with drug resistance?
    a. What effect, if any, does your prescription practice have on drug resistance?
    b. How are these cases managed?
    c. Do you think the steps you take to combat resistance at your level will make a difference at reducing global antibiotic resistance?
15. Please describe the availability and utility of any guidelines related to antibiotic use that you are aware of.
    a. Are you aware of any such guidelines?
16. What are your attitudes toward continued medical education (CME) and training programs in relation to antibiotic prescription/resistance?
    a. Do you regularly attend such programs? If not, would you consider attending them?
    b. Do you find such programs useful?
    c. What factors influence your decision to attend CME?
17. What is your level of interaction with pharmaceutical representatives?
    a. How often do they interact with you? In what capacity?
    b. How, if at all, do your interactions with pharmaceutical reps shape your prescription decisions?
    c. What impact, if any, do they have on the prevalence of antibiotic resistance?
18. What recommendations would you suggest for reducing the threat of antibiotic resistance?
    a. What strategies have been tried? What new strategies should be tried?

Pharmacists:

1. Which antibiotics are kept in stock?
    a. Why? Please describe this process.
2. What drives the supply chain for antibiotics?
3. What does the distribution of broad spectrum expensive antibiotics in the community look like?
4. What is the doctor-patient-pharmacist dynamic with regards to antibiotic use?
5. Availability and preference for stocking and distributing combination antibiotics?
6. How do you tackle oversupply of nearly expired broad spectrum antibiotics?

7. To what extent are you involved in prescribing and advising patients in the use of antibiotics?
    a. Please describe a typical encounter with a patient.
    b. What drives your decision to provide antibiotics?
    c. Do you provide anything less than a full course? If so, why and how frequently?
    d. What is your level of confidence in advising patients about antibiotics?
        i. Do you ever prescribe antibiotics without a prescription?
        ii. If the prescribed antibiotic is unavailable, what do you do?
8. What would you do if a patient comes to you with the following symptoms?
    a. Cough/cold/runny nose
    b. Fever
    c. Watery diarrhea with or without vomiting
    d. Stomach pain
    e. Rashes (on hand or leg)
9. What recommendations would you suggest for reducing the threat of antibiotic resistance?
    a. What strategies have been tried? What new strategies should be tried?

Community members:

1. Under what circumstances do you access medical care?
    a. What kind of ailments?
    b. What actions do you take when you are not feeling well?
        i. Have you ever used leftover antibiotics from a family member who was previously ill?
    c. What are some reasons people typically fall ill?
2. Can you describe the purpose of antibiotics?
    a. What are they?
    b. When do you use them?
    c. Do you think antibiotics help you get better more quickly?
3. How would you describe the availability of antibiotics?
    a. Where and how do you get them?
4. What are your perceptions toward antibiotics?
    a. What drives your decision to take antibiotics?
    b. How effective, if at all, are antibiotics according to you?
    c. Have you ever faced a situation where a doctor has not prescribed you an antibiotic and you felt you needed one?
        i. What did you do in this case?
        ii. Were you explained the reason behind this? If not, do you think an explanation would have made you more comfortable with the decision?
5. Suppose you have been prescribed a full course of antibiotics for a particular illness. After 2-3 doses of taking antibiotics, you start feeling better. What do you do?
    a. Do you finish the course or stop?
        i. Do you throw away unused pills or save them for future sicknesses?
    b. What would you do if you start feeling worse?

6. How do you decide whether to go to an allopathic doctor or an alternative medical practitioner?
    a. What factors drive this decision?
    b. If you have been to an alternative medical practitioner, have you ever been prescribed antibiotics? If so, for which ailments? Do you remember any names of antibiotics?
7. What expectations do you have from healthcare workers when you feel ill?
    a. Does the doctor explain the prescription to you?
        i. Do you know which ones are antibiotics, if any?
        ii. How often do you find antibiotics in your prescriptions?
        iii. What do you think about a doctor who doesn't prescribe antibiotics?
        iv. Do you check the expiration dates of medications you are prescribed?
8. How well do you understand the negative consequences of antibiotic use?
    a. Has anyone explained these to you during any consultations?
    b. How do you weigh the risks and benefits of taking antibiotics?
    c. Have you experienced any negative consequences?
9. What is your level of awareness around the environmental uses of antibiotics (eg. in crops or livestock)?
10. How can the understanding of the community around the appropriate uses of antibiotics be improved?
    a. How can the risks of misusing antibiotics be explained to the community?
