# Supplementary material for: “Without antibiotics, I cannot treat”: A qualitative study of antibiotic use in Paschim Bardhaman district of West Bengal, India
Source: PLoS One. 2019 Jun 27;14(6):e0219002. doi: 10.1371/journal.pone.0219002 (PMC6597109; doi:10.1371/journal.pone.0219002)
Supplement: S2 File — (ZIP) [file pone.0219002.s002.zip › S2_Transcripts/KAP 141.docx]

KAP 141

Age- 31

Sex-Male

Occupation-Pharmacist

Highest Education- Diploma in Pharmacy

Work setting- Pandabeswar PHC

I: How long you are working here?

R: almost more than 2 years.

I: More than 2 years. Ok, is there doctor sits here every day?

R: Yes he is.

I: What are the medicines you have in your stock?

R: Antibiotic, means you are mainly speaking about antibiotic or all??

I: Mainly antibiotics.

R: Mainly antibiotics. There is **Amoxicilin** with that **Azithral**, for kids there is **Amoxy kit, Ceptan,** **Ceptan D** and **Ceptan kit** is also there, these antibiotics are here and **Cifran** is there.

I: Cefran? Why these antibiotics are kept? Is there any reason?

R: **Amoxiciclin** is a 1^st^ line drug so we have to keep that. As **Amoxycilin** does not work on everyone that’s why **Azithral,** it came recently, not for a long time, as Government is supplying, we asked and it is given. **Azithra**l is needed in some case that’s why it is kept. **Amoxycilin** is not effective every time that’s why **Azthral** is kept and given.

I: So what is the supply chain process here?

R: We indent online, that is Swastho Bhaban through DRS, Burdwan, there is drug store and supplied from there.

I: Who takes the decision of what drug you will keep, what antibiotic will be kept?

R: Doctor obviously.

I: Doctor obviously. Then you make a list?

R: Yes I prepare the list. Doctor is seeing patient so what is his requirement he tells me and I indent accordingly.

I: Then you directly Swastho Bhaban

R: It is done online, they can check from Swastho Bhaban, understand.Actually that goes to DRS, they check the quantity in DRS and supply accordingly.

I: What is the full form of DRS?

R: Drug Reserve Store.

I: I see.

R: This is done.

I: So that’s the process of supply chain?

R: Yes

I: So what is required how do you find out?

R: Doctor understand that, means how much patient is improving, may be he starts with Amoxyciclin, but patient is not curing, in such case whatever decision doctor takes and it is understood that Amoxicilin may not be effective. Suppose there is Typhoid , in that case Amoxicilin may not be effective, then after blood test whatever doctor says we understand the requirement. Mainly this.

I: Do you purchase monthly?

R: Quarterly

I: Quarterly. So there is a stock and some drug will be near to expiry.

R: We indent in such a way so that it does not expire. According to patient quantity we indent so that it does not expire and if there is short expiry then we don’t bring from there. We show them there, we go for checking. When quarterly indent is done then pharmacist from every PHC or BPHC have to go there, then we have to check the stock, we have to match the quantity of supply with that manufacturing and expiry is checked. If there is short expiry then we don’t take, we asked to change that. In this way it is done.

I: Ok. Every medicine is having expiry. Whatever [medicines] you have will expire one day.

R: Obviously

I: So how do you handle those medicines which you have in your stock and near to expiry?

R: 1^st^ come 1^st^ serve means whatever has come 1^st^ will expire 1^st^ , I give to patient accordingly. It is done in this way.

I: Suppose you are having a large quantity of drug which will expire in one month or one and half month. So does it make any effect on prescription process?

R: Obviously I have to take care of what will expire and I say to doctor whtever you feel comfortable you write that and if this also not works and I see there is one month then I request to BPHC, the patient quantity is more there, I take a signature of doctor and sent there saying that you are having more patient there and disburse these drugs. It is done in this way.

I: I see means you sent to BPHC.

R: Yes then I sent to BPHC.

I: And those which you already have in your stock and expired?

R: Till now it did not happen.

I: I see.

R: It did not happen. We maintain the quantity like that and did not happen.

I: If it happens [expired] then what is the process?

R: We have destroying process for that.

I: Do you do it here?

R: If it expired then audit is done, in audit it is written expired in our register, after auditing whatever they do the report goes to Swasthobhaban. Then we say to BMOH that we have expired medicine batch no and quantity is said, and the report is already sent to Swasthobhaban, then we destroy, it is done here.

I: What is the process of destroy?

R: If it is a tablet then we defoil and destroy in the water. In this way.

I: Ok. There are three people involved in giving a medicine, doctor who is writing, pharmacist and nurse. According to what is the relation between these three?

R: Obviously if the relation is not good between Doctor, nurse and pharmacist and its very difficult to work, very difficult. Means it is very much needed. Doctor takes main decision. If to inject or someone is needed then best care is given by nurse. My role is to make people understand so that they understand the medicine, take properly, he don’t mistake, if take medicine in a wrong way may be it will be less effective. Like if Azithral is taken after food then it is surely wrong, if takes before food it will be more effective means will work better. So it is important to say all these. The role of three is surely needed. Means one is related to each other. What else to say.

I: Does people ever ask antibiotic directly from you?

R: They don’t ask antibiotic directly. But now sometimes if we say means they say there is cough and we give a cough syrup, now a days one two means literate person will say they you did not give antibiotic. They say but if they say we will give we don’t do like that. I say you take this cough syrup and it does not cure then you will take antibiotic. And they don’t say to me actually they say to doctor. If there is no doctor for any reason then may be say to me.But we give medicine means I can’t prescribe, suppose there is emergency then I give. I can’t do more than that. This happens in this way.

I: It is seen many times that patients take medicines from chemist shop.

R: Yes they obviously do that.

I: What is your opinion regarding that?

R: According to mr those who are sitting in the chemist shops if are not guided then in future it will be more difficult, means peoples drug resistance power against that drug what to say[*trying to remember*] immunity is growing automatic. Means today Amoxy is working on me may be tomorrow it will not, that is very bad, very dangerous, now a days what is happening in most of the chemist shops. May be there is no pharmacist always, may me the person who is working gives the medicine, asked [patient]. Again may be do not complete the course, may be the patient is having less money he gives only 2 antibiotics and leave, for this course is not completed, that is harming people, 2^nd^ time medicine is not working. This problem is happening. This is very dangerous.

I: In which kind of illnesses antibiotics are mostly used here?

R: Here see infection, infection with that fever aa mainly fever, infection and injury, in some injury case it is needed, in case of deep injury we should give. Other than that there are few TB patients, they should also be given, these are, with three are AG means in case dysentery, diarrhea we have to give.

I: In such cases which antibiotics are mostly used?

R: For fever Cefron, Amoxi and if ARI infection mainly for ARI infection Azithral is given, and in case of injury Cefran is given 1^st^. If patient is not cured, not improved then Amoxicilin is given. And surely for AG, dysentery Norflox and Metrozil is given.

I: When you are giving antibiotic to a patient, you are having doctor here always, suppose he is not here for any reason, so in such case you have to give

R: Yes on emergency basis I have to give.

I: Yes, you personally how much confident feel then?

R: When I feel confident I give only that, if I don’t feel I don’t give, that’s right for me. I am feeling that patients condition is bad then I send to Khandra, Ukhra BPHC that you go there and if more serious then I say to Durgapur sub division hospital, and when I see that patients condition is roughly ok if I give it will be cured then I give otherwise I don’t give. If there is fever I don’t give antibiotic then, by giving Paracitamol I say that I am giving paracitamol for three days if it gets better than good otherwise you go to other doctor or our doctor will come tomorrow come and visit him. I say this.

I: If it happens sometimes like there is no prescription but you have to give antibiotics?

R: No, I did not do that much.

I: You did not do?

R: No, I give with prescription, I say to bring prescription from home, I say again and again, if come and say I forgot then I say you go home again bring then I will give.

I: Why this, why so?

R: Here most of the people say I forgot, yes there is tendency that the paper is missing, few days back a kid came and he already took antibiotic syrup for 3 day no 6 days. Probably from January 8 to and coming on 28 and saying that there is same problem again so doctor, till then doctor did not come, he went to BDO office but he[patient] is saying no give me, doctor will be late I will take. So I said that the course of it is done I can’t give so you see the doctor. May be he became little angry on me but I send him back to home and said to come next day but he did not come, so this happens. May be took but did not eat, I don’t know what he will do, may be bring to home and keep it stored, in future if it happens then will give this. Means they themselves do the treatment many times that’s why I don’t give. Without prescription it is not given.

I: If they do like what you said, it is again given in future then what is the risk there

R: There is risk see he don’t understand if it is viral fever or other fever, so to take this is very dangerous, means for kids, for adults for human beings it is very dangerous. There may be lots of side effects. This can happen.

R: Suppose doctor has prescribed some antibiotic which you don’t have in stock, what do you do in that situation?

I:In that case I say to doctor.If the stock is over I say to doctor that the stock is going to be over, may be few more daya it will have. If we nned emergeny suppose now its February , in march quarterly indent is done, there is more days to march for indent, so in such case we call to DRS like if we can do emergency indent if they say yes, its available then we indent, emergency indent comes within 1-2 days. If they cant supply then nothing to do, e say to doctor and doctor is compelled to write medicine of outside. If there is in BPHC, if there is sufficient quantity. If there is quantity or in DRS there is no problem. So in this way it is done.

I: Here always full course of antibiotics are given?

R: full course is given.

I: Means at a time full course?

R: No, not given at a time because always have to think about quantity so patient is said to come again, they come normally.

I: After how many days?

R: Roughly it is given for three days here, and then we say to come again. Then according to the condition of the patient doctor prescribe again. It happens like that.

I: You say to come so what is the rate of this come back?

R: Come back rate roughly , here most patient are regular, they come again and again, new patient will come in future may be, they are coming I understand that but the rate is 60-70 %.

I: Come, they come back again?

R: Yes, it’s not that they don’t come, they come. We know the faces so I understand that they are coming.

I: So then you give the same prescription or doctor need to write again?

R: No, we say again and again that bring your old prescription so that doctor can understand what happened to you, it will be helpful to write so that he don’t prescribe other srug, we say again and again and they bring that. There are some people who miss, I will not say that they don’t miss, they miss and I say to them to consult the doctor. New card is done, they go to doctor if he remembers that this thing happen to him or that thing then whatever he thinks he does.

[*Pause*]

I: Do you have combination antibiotic over here?

R: No, we don’t have combination antibiotics. We don’t have combination antibiotic here, may be it is in some other places but not here.

I: In this PHC?

R: No, not in this PHC.

I: What is the availability of combination antibiotic in the market? What is your perception [regarding that]?

R: Market availability is now very much.

I: Very much?

R: Yes but recently it is said not to use combination antibiotic so may be few company has stopped supply but I think it is available, as I have seen.

I: Your preference means what is your personal preference, combination antibiotic or single?

R: It is needed in some cases, not in all cases but in some cases it is needed.

I: Like?

R: Like sometimes dysentery goes to that level so you have to give Ofloxacin and Onidazole, Many times it happens that Norflox, Metrozil don’t work then o2 which is available in market Onidazole and Ofloxacin needs to give, you have to give, nothing to do, if you don’t give this may be the patient will not be cured. So in such cases it is needed, it is not needed always, it is needed in some cases.

I: Is there any bad effect of using combination antibiotic?

R: Every drug is having side effect that is obviously there.

[*Pause*]

I: What is the availability of broad spectrum antibiotic?

R: [*Pause*] Are you saying about market?

I: If available here?

R: We are giving whatever we have; we don’t give antibiotic out of that. Other than that there are available in market a lot, every company make broad spectrum antibiotic.

[*Pause*]

I: If you can remember any typical interaction with a patient regarding antibiotic you can share with us.

R: No, I shared one that is roughly, other than that such case did not happen but only paracitamol means there is only fever I gave only paracitamol and with that one anti allergic. Suppose saying there is scratchy throat then I give Livocetirizine so in that some patient say, it is not that they don’t say, they say only two medicine will work? I say you take, if don’t work then I will give antibiotic. So they say like that these two medicines will work except that there is no such incident.

I: I will tell you some illness; in such case what will you do please tell. A patient came with cold, cough and runny nose.

R: Came with cold, cough and runny nose. I will give paracitamol and Livocetirizine.

I: Any antibiotoic?

R: Antibiotic no, I said that I don’t give antibiotic 1^st^. Generally I don’t give until doctor prescribe.2^nd^ time if I see there is no doctor then if I see the same patient I always ask the name of the patient and check the prescription, if don’t get cured then I give Amoxi 250 which is plain Amoxicilin. So it is going like that.

I: Means you will not give 1^st^ time, next time

R: No, I don’t give 1^st^ time, I give at 2^nd^ time.

I: I see

R: If there is prescription then I surely give. If there is doctor’s prescription then I give obviously, but if new patient then I don’t give.

I: Came with fever.

R: Came with fever.

I: Then what will you give? Only fever.

R: Only fever. Only Paracitamol.

I: Ok, only paracitamol.

R: Only paracitamol, because now a days fever can’t be understood without blood test, many fever is not only high fever, there is dengue, malaria, there are lot in that giving only paracitamol is safe, other thing should not be given. In case of dengue other thing cannot be given, paracitamol is safe, which is always shown in TV.

I: So in that case for how many days you will give?

R: I generally if there is doctor

I: No, suppose he is not there

R: Suppose he is not.

I: you are giving

R: Then I give for 2 days.

I: For 2 days?

R:I am saying for 2 days, no, I give for three days I say take this and if fever is decreasing then tomorrow you see the doctor sorry I say to take that for three days, it will decrease. If don’t get relief then you must visit doctor. You are seeing that fever is increasing, you took medicine but after few hours again it decrease then you can do whatever you want even in Government place. To consult a doctor is good.

I: watery diarrhoea with or without vomiting?

R: With vomiting, without vomiting.

I: Tell the both

R: In that case I give Norflox, Norfloxacin, Metronidazole and if there is vomiting then Domperidone if there is supply Domperidone otherwise Ondem, Ceptran and with that ORS. In case of ORS I say to melt a pouch in 1 litre of water and to drink it throughout the day. This and with that surely medicine of Gas, whichever is having supply, I give may be Omez or Rantac.

I: In that case for how many days you will give?

R: In this case obviously ,oh yes I forgot to tell one more ie Zink, Zink should be given in case of diereahhoea.

I: Zink?

R: Yes, Zink sulphate tablets. Here we give for three days, whatever is given is for three days. In three days they are almost cured, if did not get cure in that then the patient comes back even then also don’t cured then they get admitted at Khandra Ukhra [BPHC]. Here also in some situation like few day back in [can’t hear the name of the place] there the water is not good, there was wedding, so ate at wedding with that the water was little bit contaminated, some patient 2-3 patient were admitted there, their treatment was done there, they were given saline.

I: In such cases is there any test done before giving antibiotics? In case of Diarrhoea or ARI case?

R: No, test is not done.

I: Test is not done. If there is pain abdomen means stomach then what will you give.

R: In that case we give, 1^st^ we suspect that it may happen for gas so for gas we give a antacid, with that we have supply of Oxyphenonium bromide tablet we give that. In some case if there is a lot of pain in abdomen then I give Dotaverine. I give in this way.

I: Among these is there any antibiotic?

R: I don’t give antibiotic, it gets cure with this, if antibiotic is given then doctor gives it, I don’t give. In case of emergency I give these.

I: I see. There are rashes in hands, body, in such case?

R: Levocertizine, Cetrizine whatever is having supply I give accordingly.

I: Means in what basis do you do?

R: Suppose he is saying itching with that there are red rashes, I suppose it can be allergy so I give it. If it is scabies, in scabies it will be seen in the gap between fingers, it is very much itching, in that case we have supply of Benzyl Benzoate lotion, and we give that and with that Levocetirizine, no antibiotic is given.

I: Is there no supply of any antibiotic to put on skin?

R: Aa we have antibiotic to put on skin [*thinking*]

I:Tropical antibiotic?

R: We have mean we have Fluconidazole, Miconozole, these are given for bacterial infection.

I: Like in which kind of illness there are used?

R: For bacterial, fungal like there are red rashes which are less itchy

*Someone came and we paused the recorder*

*Again continuation*

I: We were speaking about antibiotic to put on skin

R: Yes Miconazole is given with that there is Mupirocin, Mupirocin is given in some cases, like there is insect bite or may be it is a black spot for long time may be it was rash, for itching it became black spot in that case Mupirocin is given. These two are given. Other than that for boils there was supply of Fusidic acid earlier, fusidic acid was given for boils, injury, in case of injury Mupirocin is given.

I: It happens many times that there is no doctor for any reason, you have to run the OPd, in that case can you do the prescription?

R: No, I can’t prescribe.

I: Then how do you see patient?

R: We are having a register, in that register we write name, age, sex, and give medicine accordingly, if the patient comes again on next day then we ask the name, many times patient say that I took medicine then we ask the date, after asking the name we check what medicine is given, then we say to doctor. In every case we don’t say, but mostly said, in this way it happens.

I: It is seen many times that you say the patient to come back after 3 days, and you can give medicines for 3 days so patient stop to take medicine. What is your opinion regarding this?

R: Patient don’t strop, I have seen they don’t stop, but the tendency to take medicine is more. Especially they need the tablet for gas, if other medicine is not given its ok but you have to give tablet of gas this kind of things happen. In that case may be they don’t always bring prescription, but I have to give tablet for gas, they force us, sometimes we have to give, we have nothing to do. It’s going on like this.

I: Antibiotic resistance which we discuss earlier, if you can say more about that? What is the reason or such things?

R: cause means many times asa I said from retail shop or everyone is not registered medical means RMP actually

I: RMP

R: They may be don’t understand correctly, may be 1^st^ time they give Azithral syrup to a kid, may be he don’t have such infection like there is infection in respiratory tract infection, may be he is having infection in lungs, in such case he may not need Azithral, 1^st^ line Amoxycilin would work, but he is given Azithral 1^st^, in 2^nd^ time if he goes to a practitioner he will give Amoxicilin, that amoxicillin might not work as he has taken Azithral 1^st^. it is going on continuously for long time and then Amoxicilin will not work on him.Means in this way the resistance is occurring. That is obviously harmful because antibiotics are not discovered every day, whatever is available is using now.

I: That’s right.

R: So we have to cure them through this in this way means there is shortage of medical practitioner and this makes the problem, many people are doing treatment by themselves, many are practicing from retail that is very dangerous.

I: What do you think about what other reasons contributing to resistance?

R: Other than that bacteria mutates on its own and increases the resistance power in the environment, creating antibody, so that is very bad, this can be a reason, except that I think, these two are main.

I: So to combat this we those who are working in health set up , earlier we said that doctor, pharmacist and nurse are involved in medicine disbursing process , in case of giving antibiotic. To combat this antibiotic resistance what can be the role of these three?

R: There can be role of three, means those who asks for antibiotic means after compleating three days course he feels there is little bit cough or fever, then they come and asks for 3 more days medicine, may be say by themselves, many asks, it is not that they don’t ask, in that case we should make them understand that your course is completed and now whatever you have either it is cough that can be cured by a cough syrup. You don’t need antibiotic for that, because whatever you have inside weather bacteria that are already your course is completed, it is died so it will be cured by cough syrup. Mainly if patient is made understood then most of the work is done. Many times patient understand, all patient don’t try to understand, mostly they understand so this we should make them understand in a very good way.

I: Doctor

R: Except that those who are working outside like you people are conducting many conferences or they are doing many things. The more publicity is done weather by TV, in Tv most of the news channels the programmes are done then if it is written in newspaper regarding this, most of the people follow newspaper, so if there is publicity then it will be good, in this way people will be benefitted.

I: Do you think Government can take any step at their level?

R: Government is trying, it is not that they are not trying, sometimes trainings are provided.

I: To whom?

R: The training is provided to everyone, for doctors, for nurse, for pharmacist also, training is provided to almost everyone, it is not done always, and they are trying. May be in future it will be better.

I: What can be done to do better, what steps can be taken according to you? From village level up to administrative level?

R: Sometimes mainly it will not be enoygh to provide training to the Government worker, I think those ho are working at Panchayet at panchayet level there are many people working so to them also, wheather it is panchayet member or other, if they understand the whole thing they can say it to the people in a good way. And I only said about nurse, doctor, pharmacist with them if ASHA,ANM are given training time by time, the ASHA who are working, they work in the ground level, they are visiting every home, they can make people aware if they are given training. This can be best.

I: We talked about RMP earlier, so they are also involved in giving medicine at rural area, how can we make them more efficient according to you?

R: See they are doing a course, after completing the course they start practicing but it can’t be said that if you practice then you will be experienced. I think they should have an exam, if an exam of them is taken we should check whether they pass or fail. Whether they understand properly or not, otherwise same mistake will be carried on. In this way they should be guided, except that there is nothing to do, now doctor is not available everywhere.

I: Means you think they should have training by Government?

R: From Government training is already started.

I: For them?

R: Yes training is started, that will surely not be stopped.

I: Here do you meet medical representative, do they visit here?

R-Not with me, they directly meet with doctor.

I-Do they come here?

R-Yes they come here.

I-So how do you interact with them?

R-That I don’t know, that happens with doctor, I don’t know that.

I-It is seen many times, earlier I said that many patients don’t complete the course, you give for three days still they don’t complete even if you give for 5 days then also they don’t complete. How much is that ineffective or dangerous according to you? Does that affect antibiotic resistance?

R-We try to complete the course, they do, most patient do. If the course is not completed then bacteria or whatever you call may supress and some remain in the body, may be displaced from there but in most cases it relapse. If it is fever or fungal infection, that can relapse in future. I think so.

I-So does it need to be completed?

R-Obviously it needs to be completed.

I-What is opinion regarding Continual Medical education, whether it is training or seminar?

R-No, it is obviously needed, training or seminar it needs to be attended, if it happens then well. As we are working we forget old things many times, I think that will be revised means that would be great.

I-Are you interested to participate in such things?

R- Interested but we don’t get time always.

I-That is another thing. I just want to know the interest level.

R-Yes, if I get time I like to attend specially if there is a discussion on medicine, or as you said antibiotic, if there is a vast discussion I am very much interested to hear that.

I-Would you like to say anything else about antibiotic use or antibiotic resistance?

R-No, overall what I know I said already.

I-Ok. Thank you.
