# Supplementary material for: “Without antibiotics, I cannot treat”: A qualitative study of antibiotic use in Paschim Bardhaman district of West Bengal, India
Source: PLoS One. 2019 Jun 27;14(6):e0219002. doi: 10.1371/journal.pone.0219002 (PMC6597109; doi:10.1371/journal.pone.0219002)
Supplement: S2 File — (ZIP) [file pone.0219002.s002.zip › S2_Transcripts/KAP 18.docx]

Interview: KAP 18

Age: 28

Education: B. Pharm

Gender: Male

Occupation: Pharmacist

I: For how long you are here?

R: I am here for almost 2 years and 7-8 months.

I: Ok. When you came here how was the situation, how many staffs were there?

R: You speak in Hindi no problem, he [referring to Mohit] will understand.

I: Towards Mohit, he wants in hindi.

Mohit: Ok

R: See when I when I came here then there were all medical dresser who are totally non medical staff. ok.Their education level is not related to any medical background. So when I came here I saw like puffed rice like a patient came with a little problem so he was given antibiotic and no dose were mentioned like you have to it these many days or these many days. They also don’t know which antibiotic should be given for how many days, ok. But here it was three shift, when I came 1^st^ I did not say anything, I just observed for 1^st^ one month. Aaa I observed like my survey was like it was very shocking how in three shift antibiotic means medicines were given. I used to count that number of strip of antibiotic like these antibiotic are these much and when I came next day I saw like Livoflox’s dose is for 5 days per day you shoud take 1. Livoflox were given 10, 2 time per day , Livoflox 500 mg. The doseof Azithro shoud be 3, 1^st^ day, 2^nd^ day, 3^rd^ day but here it was disbursed 6 . The strip is still here [*Showing a strip*] you can see . You see here the strip is 5 or 6, how it happened? This is the proof, ok. This is the proof that misuse is happening here. I can’t say anything; if I say my supervisor say that who will run the dispensary? You alone will run? So this is the problem here. So dosing is the problem here means dosing is not actually the problem, actually the staff those are deputed here never said to the patient. They are giving wrong dose still they they should say that you have to take these many times.

I: That is also not done?

R: That is also not done. So then what the problem is- this is all of my frustration coming out. So this patient will take for two days and will start feeling better then say am ok, felling better and stop the medicine. After 3 days or four days will come and say I fall sick again. So then I had to tell him the dosage of antibiotic and have to give him highest antibiotic which I don’t like. So this is happening here, this is the problem here, ok. Actually dosing is not dione here properly and how many days you should take the medicine properly that is also not told and proper antibiotic is also not used for proper disease. Ok.

I: When you told your supervisor 1^st^ time then how many times did you say ?

R: I said many times.

I: You said many times?

R: I did not give in writing because it would not get me into trouble, ok, I said verbally. But I am also seeing it [Dispensary] is running in this way.

I : I see

R: ok

I: Means other staffs do like this?

R: Yes, everywhere it’s the same.

I: Ok ok. So when you see this then most of the patient ask from you [Medicines] or patients are asking the doctor or doctor is giving by himself?

R: Aaa here you see the patients are not more literate but doctor but in case of medicine they are literate means sometime it happens they come and ask like give this medicine, that medicine, I ask what happened to you say me , say me the problem then say I am having this illness. So when am giving another low dose medicine they say ‘No, this does not work, I took a lot of this, it will not work, you give me something else’ means he is having plain diarrhoea then I say see nothing will happen, something wrong happen to the body mechanism which is coming out, wait for two days, if not two at least wait for one day , you just drink water with ORS [ Oral Rehydration Solution], After that you come I will check. After that hen he come if I see that patient is detoriating then I give only Metrozil ok Metronidazole, 400 mg, ok. After that if still detoriate , that medicine is for 3 days, I said after two days report me ho is the patient , if see he is good then say ok continue the medicine for that many days . After that if I see that it is detoriating then atleast I am having time of three days to observe , then I charge antibiotic like highest antibiotic Oz, Ofloxacin, Onidazole, or Cifro T, Cifrofloxacin, Cifrofloxatinizole. So after that I see that patient is cured so I wait for sometime. It happens here not only here I saw in many places , earlier I was in Burdwan Medical college there also such thing happen like antibiotic is charged at the very 1^st^ day. So what happens with that is the doctor also get relief that the patient will not bother him many times and the patient also feel that the doctor is God, one medicine and the illness is gone[*snapping his fingers*] or it also happen like Oxiflox oz or Cifro TZ whatever highest level medicine is needed for dysentery or direahhoea that time , there is also having a dose [of those medicine]but here that dosing is also not mentioned because someone gives 4, someone gives 2 , someone gives 6. They also don’t know the actual dosing. Patient also take 2-3 dose and when they start feeling better they stop taking it , some people don’t say here. They should tell here that if you feel better you should complete the course because bacteria will become resistant. So next time when you will come if you pray by holding hand this medicine will not work then, so this is problem. This is everywhere. There is problem in the whole health system, ok. How many people means how many health related staffs make the patient understand? You see they don’t want to say more. Actually this is in two part, 3 people are related to this , one is doctor who at the time of prescribing he should say that am giving you this medicine, these side effects will happen, you don’t need to worry like I am giving you a simple example of Iron. I have seen a patient who was given iron and I laughed seeing him. So after taking the medicine for two days the patient came to me on third day saying what the doctor has given, I am having black stool may be its bleeding. I asked him that who has said you its bleeding, what are you eating say me , I needed the patient history. So I think that before starting the treatment if the patient’s history is taken properly, if he is asked not like a patient but like a close relative then he will say a lot and then I thinku should u need only one antibiotic rather lots of antibiotics and you will treat them properly without hampering his resistance power so immunity problem. Ok children are coming here, people don’t understand and say give antibiotic for the kids, give antibiotic I will make them it by making ½, 1/3, ¼ but I will make them eat anyhow, he is having common cough, cold, you think. But I am against of these by sometimes I am being pressurized then I give. But I make them understand that taking this medicine many times is not good. Ok, there is another thing medicine for pain. It is very much Heparotoxic as well as paracitamol also, so I also tell them that using paracitamol may damage his liver but people don’t understand. When they are coming with complain of pain here and there then I charge simple **Paracitamol**, and with that I must [*firmly*] give medicine for gas, I give some antacid because it will secrete higher acid so more heparotoxic can damage the liver, ulcer can also happen that’s why I give antacid but people don’ take. If you surbey the whole area you will not find a single person who takes antacid with **Paracitamol**. [*Sound of whistle of passing train*] 1^st^ they take the medicine and left, second day they come and say give me medicine of pain, give me correctly, you know nothing [*laughs*]then what will I do, I give **Orizox Diclofenac** which is banned, **Orizox** should not be given, with this there will be huge heparotoxic, but in such situation I have to give it and then the patient leave happily. Ok. This is the problem means if I say taking one by one, not only antibiotic but in every medicine there is problem. Actually the problem is in using them. If the patient is counseled, if he is made understood and given themedicine then there is no problem but no one is doing this here so. I was saying that three staffs are connected to this: one is doctor who is writing the prescription so 1^st^ that patient is not given any chance to speak, that is 1^st^, 2^nd^ number is nurse who is seeing the prescription of medication after doctor, nurse also don’t say anything, they [Nurse] don’t want to speak much after that it’s us the Pharmacist where they come to take medicines.So here you see patient load is less so we can make them sit and speak but in some places there is such rush like I was in Medical college, ok **Penta** is written so give **Penta**, patient is told eat like this eat like that. So to make them sit, to make them understand like this medicine is written 3 but you need to take 5, after taking 3 come here, doctor will check and after checking he will decide whether it should be given 5 or should complete the course or need to change the medicine, there is no time to say such things [*sound of whistle of running train*]. But here I will not do such thing because I was taught, I was taught in Pharmacy ethics that 1^st^ we should use the proper use medication so then all other things. So this is my ethics that I don’t leave the patient like that, I ask them what happened, after taking history I give medicine. That’s why I am very bad; my reputation is very bad here like not good medicines are available here, so it’s ok. I think if I give proper medication to one patient then it will be my achievement.

I: So you were like this from the beginning or you realized it later?

R: [*Starts speaking before he finishes*] No no no, I am like this from the beginning because something

I: This is form training or means how did it happen? You mean this

R: See my college is Jadavpur, I did my B.Pharm from Jadavpur and diploma pharmacy from Kalyani, so there means specially when I was doing diploma there I was taught because my hospital training was at NRS so there I saw lot of mistakes like this because I just passed then and when I was studying and the place of working environment means aa I saw the upper level as well as underground then I realize what the condition is, there is no similarity with study. Then I thought that I will see one patient or treat one patient but I will do it correctly, otherwise will not do. I don’t have any power to do anything wrong to anyone, I can’t do anything good to one but I don’t have the power to do wrong to one. I am deputed here because patients have faith on me that I know something different, this thing is like that. Medication is such thing that it can kill someone as well as can treat someone. Patient are coming here thinking me means they treat the medical profession like God so I should do justice to that. So it’s alright that I get a single patient but I will make him cured- that is my way of doing things.

I: Hmm Hmm, you were speaking about training, are these taught during training?

R: Yes obviously, after diploma, when pharmacy diploma is done, after diploma there is 3 months hospital training, that is exclusively course related, so in that see it is taught that this is the medicine , give it like this or that, this is ethics, this comes automatically during study. This is not taught by anyone, this is during study means during study there is no subject on it. The ethics come during study while learning, this is ones total idea nothing else.

I: What do you think doctor are trained and doing practice so the knowledge and practice and earlier you were speaking of doctor, you were speaking of pharmacist, and you were speaking of patient, so how is the interaction of these three? What is the dynamic in giving medicines look like?

R: Yes you asked a very good question. See the interaction between three aaa there are some doctor who do drug -drug interaction, like means think about pregnant mother,here [referring to survey form ] there is a medicine **Gentamycin** which should not be given during pregnancy because it damages the fetus very badly. Some doctor in a hurry write this medicine mistakenly but I think if you are seeing one patient see him attentively, may be there are 10 patients waiting but when you are seeing the patient give him time, take his full history. From the beginning I know one thing that if the doctor takes history properlythen his diagnosis means now if you go to a doctor he will write 10 tests without any need. But if you take the history then may be you don’t need to write any test that is my belief. You were speaking about relation so sometimes doctors do some mistakes because drug- drug interaction happen then what we do when we follow the prescription and there we see that drug-drug interaction will happen because we know which drug will do interaction with which drug so this drug will do interaction then we don’t say anything in front of patient, we say to the doctor separately that sir this will cause drug-drug interaction, please change this or you have written dosing wrongly, so change this dosing. When I came here 1^st^ I caught a doctor means it should not be told, leave it. Here the dosing of **Azithromycin** was wrong means dose was totally all over the place. It should be given one but one and half were given so I said sir how you can write one and half. Then he realized and said stop, I am changing. So these types of mistakes happen here means it happens everywhere. So you were speaking of relation one patient, one prescription, the proper procedure is one prescription should go to the patient after seen by three- doctor, nurse and after that pharmacist. But here it does not happen like that, ok. Doctor writes and patient goes to pharmacist leaving nurse, and pharmacist see these medicines are written and give them. If everybody do their duty properly then the whole profession will change. But I will blame everyone because in some places there is such rush that the doctor also becomes concerned like how he will finish the whole. That’s why it is told that one prescription will be seen by three persons, doctor will see, nurse will see and the pharmacist will see. Because it is taught to all the three but here it is not done, this is the mistake in this medical profession. If I start saying then there are lots of horrible cases I have seen means what to say they are doctor, you can’t say anything to them. Not only here but in every places there are lots of horrible cases like this small mistake how they did? So its ok. Anything else?

I: Ok so here you said that the prescription will be seen by three persons and you also said that sometimes patients directly come to the pharmacist with the prescription. So how many times it happens that community people directly come to pharmacist saying I am having these problems and they don’t go the doctor?

R: Ok

I: Does it happen here many times or not?

R: It happens here. I am a pharmacist here so patients directly come to me and said I am having these problems, so now you fix them. So as I am saying like see we are taught about medicine but we are not about the condition of patient aa the disease condition of patient .That’s why we like those patients are coming to us mainly I am speaking about myself, those who are saying common thing I give medicine[*sit down to one patient entered during interview*] so I give “halka fulka” medicine for common things. After that if I see this is going out of my control, I keep under observation for 1-2 days, if I see in those two days that it is going out of my control then I say to them either go to a good private doctor or specialist right away, visit our ECL hospital [Area hospital of ECL, Kalla]. I don’t make any patient wait; I think one thing that patient has faith in me why should I break that? If something wrong happen then nothing will happen to me, I will say that I have not done this or patient forced me but ultimately I will know that I gave him wrong medicine, made him wait. That’s why I do to that level which I can mean I don’t use tough medicine, highest medicine. I keep under low dose, yes where it’s needed, I see that this case is in my knowledge, I have seen such case, like I am telling you a case, few days ago a man came, my coliary worker [*You people have timetowars us*]

I: Obviously, we have time as much you have

R: Few days ago a worker came and took little medicine for cough and cold then said, I asked how your family is? Means I am familiar with all because who knows from whom, I always try to update my knowledge, ok. So how knowledge is upgraded I told you that the harder you work, the more knowledge will be upgraded. So which patient is under what treatment, which is having what medicine, sometimes they come to me to verify what other doctor has written about medicine and test. They have faithon me and my knowledge also grows. So that day I was asking the patient what is going on at your family? So he said that in the morning his aged mother wake up and took my blessings, she could not recognize him. I have seen this case because here the problem is lack of sodium potassium. This happens to the aged person ok. When will it happen you don’t know. When there will be problem of minerals then his will be lost. I told him to call at his home and to give her glucose water, what to say; she was at home so initially I had to do something. So I said to him to tell at his home to give her sugar and salt water because in that there will be sodium and glucose so it will work little, and do the test of sodium potassium today as early as possible either doctor writes or not, I am writing, and go to your family physician with the report. He listened to me, did the sodium potassium test and went to the family physician and started the medicine given by him. So his mother got cured. If he waited for two days or one and half days his mother would expire because this case is known to me, it happened to my grandmother. So there are such things where I am having knowledge then I deal that by myself, whatever doctor has written I don’t care. I know, I am having the confidence then I deal it by myself. So if there are such diseases then I keep it under me otherwise I leave it. That’s I counsel different types of patient differently, what has happened, what did they do, what treatment is done, that is one of my hobby. So that’s it. I follow some journals, what is happening, what research is going on.

I: What are the journals you follow?

R: My means the journal that is published from my college- so in that all of the medicines that are mentioned, meaning for example there is a new journal for cancer medicine now like with the leaves of the tree, they make medicine of cancer. I mean what do I say? [can’t recognize the name] like that there is a new medicine called **Toxoclomic** [ check the spelling]. So there is some research on this drug, so I look at what is going on or not with respect to these kinds of drugs. Understand? Now, there are new branded medicines coming out. It has no benefit for me, it has no relevance for me, because…I always believe, it’s very important to me that I always avoid combination drugs. Understand?

I: Why so?

R: This because as much as you combine the drug it toxicology toxic effect will increase. It will increase the resistance power of the medicine means the resistance power of patient, on the immunity power. Because every medicine when it is combined then then there will be side effect of every combination. So patient will suffer more. May be he will be cured fast but his sufferings will increase. May be with normal medicine he will be cured in one weak and with combination drug he will be cured in two –three days. But I don’t know about the immunity level of the patient and he also dot know that. But patient comes to me and say that doctor has given me good medicine but you give bad medicine but I know what he is giving and what not. But I can’t say that to the patient, if I make the patient understand then doctor will hit me. But I say them to take normal generic medicine and say to don’t combine medicine like with this to with that medicine. Take normal medicine as much as you can. Then they say with that it takes time to cure and I say so what, it’s true that it takes time but your immunity means now you don’t fall seek regularly , so if you are falling seek in an interval then it will be cured by lately. So that’s why I try to make them understand but they don’t understand, what can I do? I can’t go to his home and say. Here thre was treatment of TB, cat 1, normal phase means 1^st^ phase ended, after that I send him for sputum test then it came positive, ok. When it came positive I said you did not take medicine correctly, because the criteria of Dot is that patient should take medicine in front of you but it is not done like that because we have faith on them and say to take medicine at home no problem. Sometimes they are having work so can’t come but if I am there I make them eat in front of me. So his resistance increased for TB. So I had to extend his medicine. It happens. Now patient should also follow what we said what not, if they use their own methodology then what will happen. That’s why I think to make the patient cure, health profession should be correct, ok. Like a chair is having four legs and if every leg is ok then you can sit, just like that patient will automatically be cured. So that’s the thing.

I: As you said you avoid combination drug so here there is no combination drug?

R: It is but I avoid. You see here I am not keeping any personal drug, whatever the company is supplying. Still I write to area medical officer like sir you please discard these combination drugs and give this. Sometimes they listened to my request and sometimes not because those combination drug is having demand here,ok. Patient becomes cured fast so they feel good.

I: So what are the drugs you keep here? What antibiotics?

R: There is antibiotic, pain killer, aa anti histamine, anti-allergic medicine.

I: What antibiotics are there?

R: Antibiotic variation?

I: Yes yes

R: There are **Azithromycin, Cefixime, Cefalexin**, aa **Levofloxacin, Erithromycin.**

I: Ok

R: **Amoxycinlin** is there.

I: It means how is the supply chain? Who brings?

R: Supply chain means see we don’t purchase anything locally, ECL directly purchase it from companies. So there are lots of companies. ECL purchase it, ECL kalla at Kalla hospital, they keep there, and from there area wise these are distributed. [Sound of whistle of running train.] This area is under Sripur so we prepare the indene that we need these medicines then I or someone else go and submit the indene and bring the medicines. Supply is done like this.

I: So means how you decide what medicines you will take?

R: See, over here, actually if I’m talking about the rules here, then the medicines that are being given frequently- if they decrease in quantity, I indent those drugs. Understand? Even then, I indent some other medicines because I see some other types of diseases. Understand? A diseased patient comes- for whom I often give 3-4 medicines, so I indent other types of medicines as well, but those are generic medicines. Those combination drugs…I was told why are you giving these generic medicines? Take these combination drugs- I [ECL hospital authority] will purchase. I told them- no you give me generic medicines. Or don’t give it to me- no problem- but I am not going to sacrifice my ethics. By giving something I know is bad- I cannot sacrifice my ethics. If you don’t want to give it to me, don’t give it, but give generic medicines if you are giving me anything. But a simple combination drug- don’t give that to me.

I: How is the distribution of broad spectrum antibiotic look like in the community? Who is taking means how many patients are taking? Who is prescribing?

R: See doctor are prescribing broad spectrum antibiotic but not mentioning the dosing correctly and the doctor also means I said you that here everyone wants their woke to be done in short span. If he is extended for long time then it is problem for him [doctor] also because the patient will come again and again and he has to prescribe him so everybody wants to give narrow spectrum medicine, ok. In narrow spectrum the dosing is less, they don’t need to make the patient understand that’s why everybody tries to use narrow spectrum antibiotic. The rational use of broad spectrum is less now.

I: So how the rational should be of broad spectrum?

R: rational use see if someone is writing Azithromycin for common cough cold it is right? If there is normal cough cold then 1^st^ give him **Paracitamol.** If someone is coming with common cough cold I check the immune system 1^st^ what is the inner immune system of the patient. So 1^st^ I give Paracitamol and leave so they say here nothing is done. So 1^st^ I give **Paracitamo**l and say do gurgling at night. After that I see what feedback comes,. I say them to come on 2^nd^ day after one day, and tell me the condition like if the sore throat has decreased or sneezing has decreased or fever has decreased? Then some patient says that fever has decreased, and sore throat has also decreased then I don’t give antibiotic. I only give Paracitamol means whatever dosing is suitable, and medicine for gas. With that I say mix salt with lukewarm water [I learned this word here] and do gurgling at night and sleep by covering it. So I say these ok. If someone is having nasal blockage here there are some people who gives nasal drops for that but nasal drop is also having its side effects it is also[can’t hear the word he said] . If you start using it once then the patient will be happy by thinking that one drop is making his nose clear but this is smooth muscle relation so there will be one day when nose will not be clear without using it. That’s why what I do is if it is a simple case I advise to take vapor of hot water by covering completely. If it is cured then alright otherwise take a carvol plus in it and inhale the vapor, it will be alright. Mostly I don’t use that nasal depargise [spelling check] that is nasal drop, I avoid it. So I treat like that.

I: And if you compare broad spectrum and narrow spectrum antibiotic then how it affects s in antibiotic resistance? If you compare the two.

R: See in narrow spectrum there is highly resistance, ok. Broad spectrum patient means totally depending upon dosing. There are some diseases where you have to give narrow spectrum and there are some diseases where you can give broad spectrum not narrow spectrum and make the patient cure.so if I give broad spectrum then I say to the patient that review is needed. If you are cured still you have to take the medicine and take the patient to me to check how is he, is he ok or not. So it depends upon how you counsel the patient, how you are making the patient understand. So depending upon that you can use broad spectrum and narrow spectrum. But there are some diseases where you have to give narrow spectrum antibiotic and there are some diseases where you have to give broad spectrum antibiotic. If doctors say this while giving broad spectrum antibiotic then our problems will decrease but they don’t say that you will feel better still you have to come, you have to take this medicine for 5 days or 10 days, no one says. If they are cured after 3 days then ok otherwise the patient will go to another doctor in a jiffy [*snapped his fingers*]. They will go and say that I am having these problems but they will not say what medicine they have taken earlier. That doctor will again charge a medicine, this contributes to antibiotic resistance.

*[Pause]*

Anything else?

I: So means only you are here, any doctor?

R: Yes there is doctor, there is a doctor- actually, this area falls under that doctor’s jurisdiction.

Actually there is shortage in ECL, ok. So lack of doctor not only in ECL but everywhere. As there is shortage of doctor, one doctor is in charge of many places so they need to travel much. Today he is in somewhere, tomorrow he will be somewhere else and day after tomorrow he will be here so in this way patient can’t be treated. Patient also thinks that here doctor is coming one day and not the 2^nd^ day so how many days i will run behind him? Lets go to outside doctor. This medicine is finished, go and see another doctor or take it from outside. These types of problems occur, ok. So this is the problem. To how many you will say?

[Sound of coughing]

I: What do you think how to tackle?

R: Tackle see tackle

I: You tell about community and then India wide how will you do?

R: Community wise see if community is fixed, then India will also be fixed. If small places are fixed, then big place will automatically be fixed.

I: Hmm

R: If I say directly then educate those are connected to medical profession, those who are connected to medicine, those who are connected to patient. They all are educated but they need to utilize the skill. If one professional uses his skill properly leaving professionalty then it will automatically be finished.

I: The broad spectrum antibiotic you mentioned and other antibiotic which expires then what happen to that and how does it affect the supply?

R: Expired? Near to expire?

I: Yes means those are near to expire.

R: Yes yes

I: So what is done with that?

R: See the antibiotic I see, expiry date is calculated accordingly. Here there is a confusion regarding the expiry date also. Here people think that means its [Showing a strip of medicine] expiry date is 2^nd^ February, 2018, ok.

I: Hmm

R: February 2018 means I will use it till January [2017].

I: ok

R: As soon as 1^st^ day of January starts its expiry will start. Ok means it will start to decompose, its carcinogenic effect will increase. So actually people thinks here means you asks anyone, its written 2/2018, February 2018 means it will be used full February. But actually 2/2018means 1^st^ day of February it will be expired.

I What is done to it after that?

R After that we prepare a list like these medicines are expired in that much of quantity . Then we buried them under soil means what to say [Towards Rina]

I You can speak in Bengali, no problem.

R We buried them or burn them means they are destroyed. Here there are lots of wells, coliary is there so we put them there.

I How it affects in prescription decision? Assume there are lots of medicines and they are going to expire soon then does it affect in distribution?

R [Starts speaking before the interviewer finishes] See distribution

I Means how many people are giving not giving?

R See distribution means it affects.

I Means the medicines you are giving to patient, whether antibiotic or not, does it affect that?

R If your question is like those which medicines are near expiry, do we disburse them more. But I think this is wrong. Ok, if those are going to expiry then leave it. My mode of action means the medicine I am giving to patient should be exact. May be they are expiring but patient is not a cow whom you can make eat anything, they are not like that, they are human being. So I means if it expiring then leave it.So whatever I am giving should be exact. May be it is not here, for example we are speaking about Azithromycin, may be it will work till January, ok. After January it will expire, so I will throw. May be I am not given from Area [main hospital] but I will work with it till 6 months then it will be wrong., absolutely wrong. That means I will not give medicine then, I will give poison. [*Pause*]. Anything else?

Rina There was a patient , can we pause?

Mohit [Towards respondent] You see the patient we can pause.

R [Restarts again] ok so during cough syrup like when you are taking any cough syrup and ask how to take then the shopkeeper or the doctor will say like take three time two spoons or shopkeeper can say see this is the nib, take two nibs three times. No one will say that don’t drink water till half an hour after taking this.But this is rule, you cant take water because its explanation is if it does not stay in the viscuss portion in the throat then how will it be cured? How will the cough be cured? Irritation will then be relieved. [sound of car horn] So if you take water just after taking the medicine then it will be erased from sore, then there is no use of it.Then patient will come and complan that youy gave cough syrup but it did not work. So when youy are giving medicine and not saying aa how to take the medicine then how will it work? Its side effect also, may be I forgot to tell him [referring to the patient just visited] but its side effect should also be explained like with this you will have little drowsiness, with cough syrup drowsiness occur but nothing to worry about it. If a small girl comes then I ask her 1^st^ if she is having any exam nearby? If she says no then I give and say after taking this you will have little drowsiness, you will feel sleepy but don’t drink water till half an hour after taking it. Now you think if you have to say these many things for a cough syruip then you think how many things are to be said if a antibiotic is given.Does anyony say? This is the problem.

I How is the patient load everyday?

R Average is 40-45 daily.

I Normally how much time do you spend with a patient?

R Till I don’t understand him properly as well as he don’t understand me properly, I don’t leave him.

I Ok means whatever the rush you will not leave him?

R No

I Very good. Do you give full course of antibiotic anytime?

R I give full course, always I give that.

I I see

R I give less then full course ,there ius a reason for this. The reason is that if full course of a medicine is 5 days then I give the patient for 3 days. Then I say you come after three day s or before fininshing the medicine, after two days, I will check you, I will check how are you,if you are ok then I will give you this medicines for another two days otherwise I will change the medicine. So it works in two ways like 1stly I can see if the patient has taken the medicine and 2ndly whatever medicine I have given is correctly or not, 3rdly if the medicine is doing any harm to patient or not and when the patient come then I counselling the patient again like you have to take this medicine two more days. So these works are get done with this.To that’s why I think whenever you are giving medicine don’t give full course, if you give the full course then patient will go home and you will not know whether he has taken that medicine or not, or thrown them or took for two days , felt better then left. That’s why I think so.

I So

R [starts speaking before he finish] there are some patient who become resistant to sulfar drugs. If I give sulfar drug to those who are having resistance to sulfar drug like Cotrimoxazole then it is very dangerous. May be death cause happen. That’s why review and counselling of patients is very important.

I- So now it happens like you said to patient to come after three days but patients don’t come, so how many patients come truly?

R- Yes this happens, this happens in maximum cases like I counsel them and send home , but the ratio of I am saying the ratio is 3 per 10. But I think that those 3 patient who came are my achievement. Its ok , start with 3, I think this way. But whom I will treat that should be done properly. I am saying you again that I can’t do good to one but I don’t want to be the reason of something wrong to someone.

I: So how does this full course of antibiotic affect the antibiotic resistance?

R: See if this is for a patient like I say take this antibiotic for 5 days three times per day or 5 days ,two times per day, for example take Clavum, Amoxycilin, Potaciam Clavolonet ok ,its dosing variate according to patient’s body weight, patient’s age. For someone it will be for days three times per day means , for someone it may be days two times per day. So I give medicine for three days to the patient. I always say while giving medicine that see you will feel better after taking this medicine for 3 days but you have to take this for 5days. That means you must come. You have to come to take the medicine and you have to complete total course even if you are cured. If you don’t complete then when again you will come then means I say them in their language like if you don’t complete it now then again when you come crying for the same disease and say give me this medicine that medicine, I will give but it will not work on you then you don’t come to me because no medicine will work. If the person understands is ok or those who don’t understand you have to make them understand in a good way.Because by any means I have to complete the full doing to the patient, that’s my responsibility, because they don’t understand. That’s it.

I-So means how do you take the decision to whom you have to give antibiotic and whom to not? What are the drives?

R: See some patients here are I mean this is a company’s hospital and maximum patients are the family members of the workers, so I know maximum patients and which antibiotic will suit them, what antibiotics are going. If some new patient come then 1^st^ I check their resistance. I make them wait maximum for two days, ok, he is seek for few days so two more days. I check their resistance in two days.

I: Means by giving medicine or with testing?

R: By giving medicines, not with testing. I take history 1^st^, after taking history I don’t go for a test 1^st^. If I understand after taking history that this I can handle then I say them. Few days ago there was dengue season, any patient came I gave Paracitamol and said to do their blood test. I said for blood test immediately and don’t come here with the test report; go to your family physician or to the Kalla Central hospital, because from there they will againg send for test so what I am doing here? So I gave them Paracitamol and said to go for blood test. This is another thing and if there is common thing like the Direahhoea patient [Referring *to the patient just visited the clinic*]came, if come tomorrow and say that this medicine did not work then I will charge antibiotic saying that take these antibiotics for these many days may be your child is cured but she should take these many days. Metrogil is having a side effect,metallic taste, it changes the taste of mouth. This is also need to be said otherwise after taking two days they will say this is not a good medicine and will not complete the course. This you need to make understand the patient, so these are the problems. Guide the patient properly, say them about proper dosing, side effect should be explained. Then if patient is having faith then he will automatically listened to you. That’s what I think.

I: So you give medicine without prescription? Antibiotic or not?

R: See actual rule is to give with prescription but here patients are known to me, if there is a new patient then I obviously give proper prescription. I give because it works in two ways. See I can’t remember every time what medicine I have given to which patient, here I am having a register [*showing his register copy]* , I wrote that’s another thing. I can recall with this I have given to whom but if that patient goes to another doctor then he will not be to say what medicine he has taken so I write it. He will show that to the doctor like I have taken this medicine for these many days but m not cured, say what to do. Then that doctor can charge another medicine or more doses of the same medicine according to his understanding. Ok, that’s why prescription is important.

Someone came and we paused

After restarting

I: When you are seeing a patient then what is your confidence level like this is the correct medicine to give? Suppose there is no prescription.

R: Ok see this is also important. If your opposite person is not confident then how will you believe him? That’s why I should be confident that whatever diagnosis I have done is exact. After that the patient will understand that yes he is confident means it’s correct, so we have to take the medicine. Ok. So 1^st^ I have to be confident about my diagnosis, about my medication and then the patients also have faith on me.

I: Ok so we were speaking about expiry drug, about disposal. So as you are having area hospital, you send the expired drugs there for disposal or you do it here yourself?

R: See there is no rule that we have to send area hospital. We prepare a list like these many drugs with batch no, manufacturing date, quantity, dose amount had expired. So a list is prepared where I signed, doctor sign or any agent or manager sing. After signing those expired drugs are buried or burnt in front of him. In this way the disposal is done. Then we send the report to area hospital.

I: Is there any proper place or anywhere?

R: [*Starts before the interviewer finish*] No, proper place means in colliery means where normal people can’t reach because if I throw at road and someone take thinking that good medicines , he don’t know what it is. If he eats one then its problem. No one can touch me but he will be in trouble. That’s why it is taught during our study that you have to disposal means aa the medication where the human cannot reach.

I: If the antibiotic you want to give or the prescribed antibiotic is not here then what will you do? What normally happens in this situation?

R: If the antibiotic I want to give is not here?

I: [*Interrupting him]* It is not in stock

R: If its not in stock then I tell them in their language that if you want to be cured then you need good medicine, don’t take these medicine[available in hospital] because if I tell that the medicine I want to give is not available here, you take from outside then they will not take. He will say means his version will be like whatever medicine is available here, give me. Patient party those who comes here, among them 99% patient’s mental set up is like this is a free hospital, medicines are free here so we have to take medicine from here whether its available or not they need it from here. I am seeing patients for long so I have idea how to treat them and I tell them in their language. So I say them that with this medicine you will not be cured so I will write and you take that from shop and take the full course but come to me after taking three days. I don’t say that I want to know how you are but I say come to me after three days like whether the shopkeeper has given correct medicine.

I: I see [*Laughs*]

R: With this the patient become cautious and they come after three days.

I: That’s a good idea. [*Laughs*]

R: [*laughs*] I have to make the patient understand. He says see these are the medicines you have written. Most of the shopkeepers don’t do mistakes and I can see that he has taken the medicine. I say, it’s the correct medicine [*firmly*], take it for two more days and you will be cured.

I: [*laughs out loud*]

R: So this is the idea.

I: Ok if a patient come and complain about some symptoms then what medicine will you give?

R: Some symptoms like?

I: Means I will tell you the example now.

R: Hmm

I: Like a patient says that he is having cough and cold, running nose, only that nothing else then what will you give to that patient?

R: Having cough and cold so 1stly I will ask him if he is having body pain. See running nose is there, cough and cold is there then there must be body pain. Ok, fever type tendency must be there because from that infection it happened. So I will give Paracitamol and age wise if a adult person then I will give 500 mg 3 times per day TDS and will say to take that for two days and to meet me after that. For the running nose I will say that this is the inner cough which is coming out and its good for you, if it dried its not good so use handkerchief. With that I will advise to take vapor at night if he face nasal obstacle, if that does not work then mix a Carvol plus with that and take that vapor, it does not have any side effect.[*Speaks with someone*]

I: After that?

R: After that if says cough is there then I will give cough syrup and while giving that I will surely mention to take that after dinner at night and in the morning after breakfast, three times per day. Take that after breakfast and after drinking water and take the medicine one spoon but don’t eat or drink anything till half an hour after taking the medicine , do the same after lunch also. While explaining how to take at night I will say in a friendly way because if you speak in that way they will understand better. So at night after your dinner and taking other medicine take it just before sleep, you will have a good sleep and you will feel better. He will think this is ok.

I: If there is fever?

R: If fever is there then I will only give Paracitamol, if he feels better with Paracitamol then its ok. In these two days I will understand his immunity and while giving antibiotic I will understand the dosing. If he comes after two days and say there is no fever or having little fever only at morning and night then I will charge low dose antibiotic.

I: Ok

R: Or maybe I will not give antibiotic, only Paracitamol .

I: if there is watery direahhoea with or without vomiting?

R: Aaa if there is watery direahhoea with or without vomiting then see if there is vomiting its ok but for direahhoea I need to ask for how many times , that’s a vital issue. If for one or two times then I will advise to take ORS. If the patient comes to me and ORS is available here then will give that otherwise I will advise to take water with salt and sugar, whole day with interval, nothing else, don’t take any wrong food , only take food that will make your stomach colder like puffed rice with water and sugar, smashed rice , swallow it. But don’t take any rich food. I will see for two days, ok, or may be one day. If I see that it has increased in one day then I will charge antibiotic. I will charge antibiotic according to the condition of the patient. If with vomiting I will say the same and few minutes earlier there was a patient came and I said that during this period all the body minerals are going away, that’s why there is chance of dehydration. I will say like all the body water will extract so you will not be able to stand up, you will be lying down all time so take this water for few more days. If there is vomiting then I will write an anti-vomiting like **Domestila** , if vomiting increases then there is **Ondem**, take this. I will not charge antibiotic 1^st^.

I: So in this situation like direahhoea , before charging antibiotic is stool test is done?

R: You see actually it should be done but I don’t do. You can think this is my fault or whatever you think. After charging antibiotic if he is cured then its ok. While a patient is suffering from loose motion and I say for stool test then he will say that you leave, I will take medicine from outside. So it’s better to take medicine under my treatment than taking wrong medicine from outside.

I: Aaa if there is stomach pain? Only stomach pain.

R: If there is stomach pain, then I will not give anything for the stomach pain directly, because I will make him understand that this is anti-amoebic dysentery. There are aam in the stomach which is why-

[*His phone rang and he said to pause*]

*Continuing-*

This is common, whoever is having dysentery or diarrhoea he will have stomach pain, because if amm is there then periosteal movement will not be ok , so stomach pain must be there. So I will not give medicine for stomach pain, I will make him understand as soon as the aam will go out; stomach pain will also go away. So I will not give medicine for stomach pain when not needed. Because medicine for stomach pain is also carsino or Heparotoxic . So why should I increase his problem.

I: I see, so no antibiotic and no pain killer also, mean in that situation you will not give Paracitamol also?

R: In that situation for stomach pain

I: [interrupting him] so you will not give medicine

R: First I will take out the aam, and then automatically, if they get better then good, otherwise I will think of other medicines.

I: If there is skin rashes in body?

R: If there is skin rashes then 1stly I will take the history like what he has eaten, what is the problem, how does it happen, ok. See during diarrhoea it is also allergic means allergic symptom. If something does not suit him like egg, there are some people who will have loose motion and skin if he eats egg . So I will take history like what he has eaten in last one or two days.as soon as he tell that I will check if there is any such food which is allergic. If there is a food of allergic tendency then I will obviously give him anti-histamine, anti-allergic drug to take that.

I: So if we want to work on antibiotic resistance in this country then what should we do at policy level? What are the strategies?

R: At Policy level see 1^st^ we should counsel the patients. 1^st^ patients should know what will have if there is antibiotic resistance. After that all the health professional those who are health professional

I: who will be among them? [Health professional]

R: Aa like there is doctor, nurse, pharmacist, medical dresser means those who are related to medicine all those should be cautious about their profession like I am treating the patient, treating a human being not any animal those who don’t understand , I am treating a knowledgeable person. So they should be counseled like I have given you antibiotic for that many days, you have to complete the dose even if are cured.If you don’t take then you will come by crying and then it will not work on you by any means, and I will not be able to give you any medicine, if then also don’t understand then I you should say if this medicine does not work then you need to be hospitalized and injected. Injection will be given to you dear. We need to make him understand that if you don’t take medicines regularly then you will die. [*Someone calls from outside and he nodded to him*]. By anyhow we should make him habituate to take medicine by proper dosing and duration. If he is habituated once in it then I don’t have to put much time on him. So 1^st^ we have to counsel the patient, we have to give proper knowledge to the patient that about the dosing and about the adverse effect of medication. I will tell about dosing like you have to take that many days but I should also tell about the side effects that after taking this medicine you can have such problems but don’t worry, you have to take the medicine ok. Like Calcium, I can say confidently that 99% doctor don’t say while prescribing Calcium that after taking Calcium you will have constipation ok. So patient take for 2-3 days after that they leave. So what is his benefit, zero. So just like I need to tell patients about the dosing and how many days he has to take as well as I should say about the adverse effect of the medicine. There are some medicines, antibiotic with which you can’t take some food, for example **Tetracycline**; you can’t take milk with tetracycline. There are some medicine which should not be taken during pregnancy. Some antibiotics as in…some antibiotics meaning some medicines as in…when they should not be taken- this needs to be explained to the patient, that see you can take this medicine now, but who knows, patients who are more knowledgeable- they will say- look I went to the doctor with so many problems and they prescribed this medicine. He keeps the wrapper of the medicine. Understand? Even if he eats it, he saves the wrapper. He got better, but he kept the wrapper of the medicine. Next time it happens, he will show the wrapper to the chemist shop and buy the medicine- he will not come [to the doctor first **sounds regretful**]. But he/she may have another ailment then, they might be pregnant, understand? They shouldn’t be taking that medicine in this case. Needs another medicine. So, the patient will not understand, which is why I also have to explain that look this is your condition now- you can take this medicine for this condition, but if you have another ailment, you must come to me first before taking the same medicine. Or go see another doctor. Do not simply go and buy this medicine on your own. I have to explain this to the patient- they don’t know anything, and at the same time, they think they know a lot. Understand? So we have to treat them accordingly. Anything else?

I: Is there anything done, meaning, do you know the Government has done anything to control this?

R: yes Govt does trial means trial is done in phase 1, phase 2, phase 3, phase 4, phase 5, phase 6, they do trial, clinical trial is going on in it but now if those who are related to it are not right then what is the use of trial?

I: And is govt doing anything to control that mean antibiotic resistance?

R: Yes they are doing like they said to give generic medicine, and to make the strip according to doses,ok, with this patient will understand better that doctor has written for that many days, then will take a strip from shop and will complete it. Like it is going on in the market that for Azithromycin the strip is of 3, you will not get 6 or 10 in a strip and the shopkeeper can’t say to take that because he will have problem to sell the cut strip. So govt is making this kind of strip for little highly resistant medicine.

I: Do you want to say anything else in regarding anything?

R: I want to say one thing that we are different from normal general public, general public treat us like God, so don’t play with them, listen to him with care whatever he want to say . If a boy doesn’t give time to his girlfriend then she will also leave him [*laughs out loud all*] so like this if he don’t listen to her then she will leave being frustrated just like this if you don’t listen to the patient then patient will also not believe you. So treat him in a way that he will have faith on you then it will not be tough to make the patient understand. If a patient start believing you from the 1^st^ visit then you will not have problem whenever he comes. Ok, that’s simple.

I: Every time patient comes here themselves or send their relative like I am having this problem or take this medicine for me?

R: I see, this is a very important question because it happens like patients send their relatives, but if it is a common disease common disease means there is no vital issue, no need of antibiotic, if such common disease like just now a lady came said about her daughter and I just gave ORS and told to report me after one or two days , if she is cured then ok otherwise I will change the medicine. I don’t give any important medicine any critical medicine without seeing the patient, don’t even think about antibiotic. If I have to give pain killer then also I only give Paracitamol but I don’t give the pain killer like **Diclofenac or Ibruprofen .** I say to bring the patient and after seeing I will give medicine. Because when the patient will come then I will ask him two three question like if there is pain in stomach? Do you have acidity problem? How many times do you suffer with pain? So I will give medicine accordingly. If his 1^st^ complaint is suffering from pain in a regular interval then I will not give medicine because he may have ulcerous tendency so it can increase. Anything else?

I: Ok. Thank you.
