# Supplementary material for: “Without antibiotics, I cannot treat”: A qualitative study of antibiotic use in Paschim Bardhaman district of West Bengal, India
Source: PLoS One. 2019 Jun 27;14(6):e0219002. doi: 10.1371/journal.pone.0219002 (PMC6597109; doi:10.1371/journal.pone.0219002)
Supplement: S2 File — (ZIP) [file pone.0219002.s002.zip › S2_Transcripts/KAP 185.docx]

KAP -185

Gender: Female

Age: 35

Occupation: Nurse

Highest education: GNM degree

I-How long you are working over here? How many years?

R-I completed one year here.

I-You completed one year. How is the patient load here?

R: Patient load is quite good. Load means average 75-80 per day, someday there may be 150 and other day it may be 10, so average is that.

I: What are the diseases commonly seen here?

R: Here disease means ARI, Tuberculosis, worm infestation aa [*pause]* little bit cough, cold etc those which are ARI, diabetic, hypertension. Mainly there is Tuberculosis, ARI, Cough and cold which occurs for the dust, allergy.

I: We have seen a lot of dust.

R: There is lots of dust here.

I: Does skin problem seen over here?

R: Yes there is skin problem; there is lot of skin problem like scabies.

I: Please explain a little more what else?

R: Mainly scabies is more, itching is more, there is 2-4 filaria patient, not much but there is and spots in the face. Mainly there is scabies, itching, maximum patient are coming for scabies.

I: Here you have a medical means a small medical store. What medicines are generally kept especially antibiotic?

R: Antibiotic there is **Azithromicin,** **cetron**, **metrozil** then there is **cetron, metrozil, amoxiclave** **capsule** and **amoxicili.** For kids there is **metrozil ,cefodime** syrup and **Azithromnicin syrup** that’s all.

I: Who takes the decision of what antibiotics will be kept ?

R: Doctor and the pharmacist together they take, they indent and whatever comes from there.

I: It happens many times that in absence of doctor you need to handle OPd.

R: Yes, it happens.

I: So in such case how do you see patient?

R: How whatever the patient say about sign and symptoms then whatever my experience, we have a pharmacy course book- pharmacology, so from there and some from my experience seeing the doctor giving and by searching from internet I give.

I: So then I am saying in case of his absence when you are handling the OPD, then how much time it takes you to see a patient?

R: Actually in this busy schedule 5-10 minutes, it is also not given, like they said diarrhea, if say diarrhea he is given medicine then and there, they say about the symptoms of diarrhea means it is not possible to give more than 5-10 minutes. It is tough to give 10 minute because it is not possible to see 100 patients in 1-1:30 hour.

I: Can you prescribe?

R: No, we can’t prescribe.

I: Then how in his [doctor] absence?

R: We have a register, we write the name there and medicine is given.

I: I see, you give the medicine directly?

R: Medicine is given, when the doctor comes, if any problem occurs we open it and get all information about what is given.

I: Ok, you can’t write the prescription directly?

R: No, we don’t have the right of prescription.

I: Is there tropical antibiotic which is used for skin?

R: Yes there is mEtronidazole then candid, candid is not antibiotic, candid is there and [trying to remember] these two and candid B is there, no only candid, there is plain candid and Metrtonidazole and mupirocin ointment.

I: I see so these I means is is the tropical antibiotic used?

R: Yes, candid is given.

I: It is used.

R: Less but used.

I: Less ok, as you have to handle OPD sometimes, you do so, so I am saying some diseases and you say what you will give to patient in such case. Cough, cold and runny nose.

R: Runny nose?

I: Cough cold and runny nose [*repeats the same*]

R: Cough cold and runny nose then Amoxiclave will be given and for fever paracitamol, for runny nose cerzin and with that an antacid will be given.

I: Ok, for how many days will you give?

R: We give for three days, if it cures after three days then its ok, if not cures then we say to come again.

I: Only fever.

R: For only fever paracitamol and antacid.

I: Paracitamol, in this case you will not give antibiotic?

R: No, I will not give antibiotic.

I: Why you will not give antibiotic in this case?

R: Because fever can happen in many case, so I can’t do it. Then if doctor is not in the OPD among three days if there is Saturday, he comes within 3 days so I tell the patient to come again.

I: I see, so you will see for three days 1^st^?

R: Yes

I: Watery diarrhea with or without vomiting?

R: Cefron, Metrozil is given, if there is no cefron then norflox is given, norflox is not running, cefron is given, cefron, metrozil, a ORS and antacid whatever is available is given.

I: For how many days will you give in this case?

R: In this case also it is given for three days, most of our medicine here is given for three days. If there is having a old infection of someone then it is given for 5 days.

I: Stomach pain.

R: Stomach pain. In stomach pain if say the upper portion

I: [*interrupting*] you say everything.

R: If say burning then antacid is given, antacid and Dotin is given.

I: No antibiotic in this case?

R: No, no antibiotic

I: Rashes, there is rashes in the bosy.

R: BB lotion is given and Cerzin

I: If there is respiratory tract infection

R: [*Interrupting*] ARI

I: Yes

R: In that case Amoxicilin is used.

I: Amoxicilin?

R: Amoxici CV

I: CV

R: Yes, if there is history of cough, cough for long time then amoxi caps capsule is used.

I: So in ARI and diarrhea cases do you give antibiotic only after testing?

R: No, Testing is not done. !st medicine is given then if there is any problem, doctor writes, we don’t write, doctor writes.

I: Is there any availability of testing?

R: There is but that is not enough. With the collaboration of govt one sits from outside, there diabetic is done but not something big is done.

I: Normal urine, blood these?

R: these are done.

I: You have indoor here?

R: Yes, we have indoor.

I: Of how many bed?

R: Here it is of 10 bed.

I: I see, there only maternity dept is working?

R: No, maternity, diarrhea, maternity, diarrhea and something small cases are done. No big case is kept.

I: So small micro surgery or something like that?

R: No, surgery is not done here. Any kind of surgery is not done here.

I: Means there is no such infrastructure?

R: No. there is no infrastructure.

I: Small stitching, saline these?

R: These are done, these are done mainly f there is a doctor.

I: Means there is arrangements, I wanted to know that. [*Pause*] When suppose you only give in absence of doctor, if there is doctor you will not give, so when in absence of doctor you are giving antibiotic to someone then how much confident do you feel yourself?

R: Confident is ok in these case, means it is ok in small cases, but when I see there is something big happened then we don’t attend, then they are sent. If there is cough, cold, fever with that like there was a patient with headache, stomach pain with that cough cold so we referred that case to khandra [BPHC] We give any antibiotic, there is stomach pain, headache is also there, in such situation we send them to Khandra, khandra is not so far, in between 7-8 km, we send there.

I: In such case means by seeing what do you take the decision that this patient needs antibiotic? When you are giving then what factors do you look for?

R: Seeing the condition of the patient, means if there is diarrhea there will be a condition, after seeing that condition medicine is given.

I: You mean after by seeing sign and symptoms?

R: By seeing sign and symptoms.

I: How much diagnosis factor in giving antibiotic?

R: Diagnosis matters a lot. If diagnosis is not done correctly, then correct antibiotic will also not be given. That is a huge thing, for that testing is also needed, what is done here is not correct but we are forced to, cause if there is not proper diagnosis then proper antibiotic can’t be given.

I: In such case there might be fear of what?

R: Many things like if wrong antibiotic is given then there can be side effect plus that will not be cured for what he has come.

I: Basically for what illness you will prefer antibiotic? Normally the diseases you are seeing among them?

R: Here the dust problem is very much, for ARI a antibiotic must be given. For diarrhea antibiotic is not needed much but here people live in unhygienic way that they must be given, because they are very much unhygienic, like they drink raw water, don’t maintain any hygiene, in that case there is a fear of spread, in that case here it must be given.

[*Pause*]

I: How much patients are involved in giving antibiotic here?

R: They are involve very much because they want to be cured fast, even there are some patient who says that you did not give the medicine which you gave last time, after taking that medicine it was cured. The thing is they don’t have any knowledge regarding the course; here there is lack of knowledge here. In maximum case it happens that if one doesn’t prefer to give antibiotic then patient want it very much. Actually if you give antibiotic then signs and symptoms are cured fast so they don’t want to wait.

I: According to you why do they think so?

R: They don’t have knowledge about antibiotic, what is antibiotic, what is its side effect, how it can harm them, they know that if I take antibiotic I will be cured fast. They don’t have any idea that it is having side effects. If is become resistant it can harm them later, they don’t have such idea. Actually they are habituated by taking antibiotics from shops; they see that one dose of antibiotic decreases the illness, means they think sometimes that medicine from outside cures fast.

I: what is your opinion about taking medicine from outside?

R: This is a very bad thing because the most important thing is that who is giving medicine in the shop himself know anything, those who are giving don’t have knowledge, because I think no pharmacist is sitting in any shop here, I belong to this area, as far as I know they don’t sits here. Here 7^th^ pass, 8^th^ pass, 10^th^ pass people are sitting in the shop, seeing prescription of doctors that for this kind of sign symptoms they are giving this antibiotic and they are also giving accordingly. Means the antibiotic can have side effects, they don’t have knowledge of that and they [shopkeepers] are giving like this. I myself is a sufferer, by giving me paracitamol again and again my liver has been damaged, they did not give any antacid with paracitamol, from childhood I had a pain in leg that’s why they used to give that, my liver is damaged by taking in this way, they don’t have the knowledge that with paracitamol an antacid should be given. By giving in this way maximum people here are becoming resistant and they don’t know even.

I: Many people don’t take proper dose of antibiotic.

R: No, they don’t take proper dose, in this case maximum patients are responsible because as their sign symptoms decreases they start negligence, I am cured I will not take anymore, this I have seen sometimes, in my family there are some like they are cured and will not take anymore.

I: Why don’t they want to take?

R: I think absence of knowledge, absence of knowledge is a great thing, means antibiotic can harm, it will not work later, in this case this knowledge and there are some orthodox kind of people here, if we try to make them understand they don’t, he is cured, ok, nothing will happen anymore. Kind of overconfidence, it is cured, nothing will happen to me.

I: Do they ask for antibiotic directly over here?

R: Yes, they ask, some people ask.

I: They ask? Do they say by name or how they say?

R: No, after giving like is this antibiotic? I don’t know how much they know about antibiotic or not, they say like you gave this, is it antibiotic?

I: Why they are tending to antibiotic? What do you think?

R: They think if they take this they will be cured fast, taking this means I will be cured fast.

I: Means it can be called superstition?

R: Yes, superstition. Knowledge about its side effects, having knowledge about its work like if I take this I will be cured, but it can harm you this knowledge is very less here.

I: In many cases many people go to RMP, What is your opinion about that? Means what is your opinion about RMP?

R: They can’t be blamed because in this case best medical means we don’t have that much medical officer, absence means very less here, we are having our doctor here and in ECL, in ECL they will not see patient except employee of ECL. Otherwise there are 2-4 doctors who are for very less time, they come for 2 hours and go, many times they are not available, in that situation they are forced, communication is also not very good so they are compelled to go there.

I: And what is your opinion about them means RMP?

R: What will I say [*laughs*] about them. In some case they really help people, saying this is wrong, when there is no one they only help, but this a illegal work, nothing is to be said about them. Some people are benefitted but maximum people are harmed.

I: When you are giving antibiotic to one there is a risk as well as benefit, how do you see this?

R: There are some cases where it is to be given, which will not cure, in that case we must give. There is little risk in everything but I have to see the god side that it will cure, there is such case where if you don’t give there is nothing.

I: if you can cite some example of what are the risk may have?

R: There may be risk, in many case it can be life threatening like someone is having allergy in sulfur drug in this case if he takes Cefron then it may have problem, in this case there will be nausea, vomiting, dizziness. The most afraid thing is in case of pregnant women; in case of them there is a matter of fetal anomaly, in such case pregnant women are not given, we don’t give means we avoid.

I: Is there any fear of miscarriage?

R: There is a chance of miscarriage, fetal anomaly, there is chance of harming the baby, we generally don’t give antibiotic to pregnant women, we refer them to doctor.

I: It happens many times that patient live far away

*Someone came and we paused*

*Continue*

A patient lives far away and you felt that he needs antibiotics for long days, in such what will you do?

R: Long days means we don’t give more than five days.

I: You can give for five days?

R: We give for five days, not more than that, if he has problem after that we tell them to come again please come , nothing to do, you have problem, you have to come.

I: Just now you said that you give medicines for three days.

R: We give for three days but if there is some old infection then when we see that it will not cure, we give them for five days.

I: Ok so you can give medicine for three days at a time, so in case of antibiotic also you give for three days 1st, so what is the rate of coming back of those who need more?

R: Mostly they don’t come.

I: Follow up?

R: Follow up means those who are TB patients they come, and diarrhea cases are cured.

I: So those whom you tell to come like take medicine after three days?

R: If they are cured they don’t come anyhow.

I: I see.

R: Here follow up is done for TB patient, diabetic patients come, and HTN [hypertension] patients come.

I: Those whom you are giving normal antibiotic daily, what about their follow up?

R: No, mostly they don’t come, those who are not cured come. In many cases those whose work means if medicine of hospital don’t work they buy from outside, their perception is that medicine of hospital don’t work.

I: Why do they think so?

R: Actually we are giving less power antibiotic which work slow and there 1^st^ time they will give high power 4^th^ generation drug, it works fast so they think that the medicine of hospital don’t work.

I: Ok, so in giving a medicine there are three people involved, one is doctor, nurse and pharmacist. What is the role of these three in giving antibiotics?

R: 1st doctor prescribe and pharmacist give, we are not that much involved, we are involved in patient care; in absence of these two we give. Mainly doctor and pharmacist are there, doctor prescribes and our pharmacist gives. We do like checking pressure, checking BP, dressing etc. But when we see that both of them are not there then we involve ourselves.

I: When you handle OPD.

R: Yes

I: Did you get drug resistant patient here?

R: Not such patient came. Maximum come like TB cat 1 failure, their case is they leave, when they discontinue it happens. I did not get such case; I am here for one year.

I: Any conversation with a typical patient means asking for medicine or don’t want to listen to you, or such typical incident. If you can remember 1-2 such incident.

R: Yes there are such cases, maximum is old women mean she comes everyday saying give me this medicine, give medicine for gas, after everything give me medicine for itching, after doing this check my pressure, she is aged woman, if she come it takes minimum half an hour. Weather there is a need or not everything, give me medicine for gas, if calcium is of different colour, many times colour is changed why you did not give me white colour medicine, medicine for pain why you did not give me blue colour medicine these kind of, we say grandma you seat for sometime, let us see everyone then will see you. There is another one Kiran Maji, he comes and say give me that medicine, last time you give capsule for gas why you give tablet this time, pan 40 these are changing, there is omez then pan 40, omez is capsule why you did not give.

I: Many patients come to you and ask for medicines without going to the doctor, so how do you manage those patients?

R: No, when there is doctor we say that there is doctor, go to him after that we are here, If there is doctor we don’t give medicine at the counter.

I: Suppose he is not here, and a patient comes to you asking for a medicine by name, how do you handle that?

R: Those are not given, tell us what happened to you and will give medicine accordingly. Give this, give that, what problem is your and if it requires in that I have to check that, it is not given in that way, if they tell the name of medicine it is not given. If he is having problem, that medicine is truly needed, if I think that he need that, then only given. In case of gas they say that I am having gas and given, that is another thing.

I: Antibiotic?

R: Antibiotic is not given in that way, no one is given in that way.

I: Do you know any guideline related to antibiotic or you follow?

R: I search from internet, but such guideline, when I was studying then there was a book. [*Paused*]. I read Drug Today when I was studying, there was a book called Drug Today.

I: What is your opinion about continued medical education, trainings?

R: These are very much needed because in medical line there is new invention everyday, new changes are occurring, if these are not organized, if not updated then we will be backward, there will be no difference of lay man with us. When I did my training that knowledge and today after 10 years there is a huge change. This is very much needed, otherwise how will we be updated? We remain the same.

I: If it is conducted will you be interested to participate?

R: Yes obviously, why not? Who wants to remain backward? [*Pause*] It is needed, very much needed.

I: What are the drives that encourage you to participate in such training? What influence you that yes I will I will participate, makes you interested towards these trainings, seminars?

R: when I see there is a new invention or when I see my junior girls are doing better, hers is better than I think that I should also know that, these are needed, to have knowledge is needed or what I know may be, like when I learned in training when I 1^st^ joined in child ward then I saw that they used to do burping. Burping is tapping in the back then after one year I saw that they are not burping but towards upside [*showing by hand*] like from stomach towards upside. These knowledge I did not know these so I learned by watching them, so these should be known. Then while doing channel we learned in a way and saw they are doing in another way, then I saw theirs is better, so everybody need to know that as we are in this line.

*[Pause]*

I: If you please explain what is your opinion regarding antibiotic resistance?

R: My opinion regarding antibiotic resistance like if an antibiotic is not used completely then there is a chance of resistance and viral in case of viral it is not needed, as bacteria or something like that they become strong depending upon their feature then no low antibiotic will work on it and there are some antibiotics, some bacteria which do not respond on some antibiotics.

I: What other reasons can be of antibiotic resistance?

R: Bacteria or what has attacked that germ becomes strong or it is exposed several times in front of any antibiotic in that case they themselves become strong then it became resistant, I think so.

I: Is common people responsible for antibiotic resistance anyhow?

R: Obviously they are responsible, they don’t know proper use, they don’t use properly, they are going to RMP or there is a huge difference between the knowledge of RMP and a medical officer. I think doctor knows better than RMP, what I will prescribe if it is done by doctor or pharmacist his knowledge is much more. They don’t go to them; don’t take medicine from proper person like those who are giving in medicine shop they give for two days dose, not for 3 days dose. Many antibiotic is having 5 days course, 7 days course, by not giving money they are giving 2-3 days dose then there must be resistance, next time it will not work or when by taking something they see that there is no sign symptoms, I am cured so they use half and not the other half, in that case there will be resistance.

I: How can it be combated?

R: This to give proper knowledge, education is a huge thing.

I: In health sector we three are involved-doctor, nurse and pharmacist. So what can be the role of these three to combat this?

R: To combat main thing is to explain them properly. May be no one will understand in one day’s explanation but if I explain to 10 patient and make one understand that is also my benefit, I make one people understand that you take this completely otherwise it will not cure, may be it is curing outside and you are not understanding but your illness will remain inside, next time this medicine will not work, in this way explain again and again to people, one day he will listen to you.

I: Who should to do it among these three?

R: All should do it, all three should explain, I think to whom the patient is exposed should explain. Here it is done also, it is not that it is not done, may be now they don’t listen but slowly they will listen, may be the rate has decreased from earlier, it needs to be explained in this way.

I: Do you know if Government has taken any strategy to combat this?

R: I don’t know exactly.

I: what do you think what strategy can be taken in every level from up to grassroots?

R: 1^st^ is training should be provided to those who work in the health sector, to provide training to make them understand properly, there are some people in some level they themselves don’t know like ASHA, ANM they just passed Madhyamik level and took a training, they should also be provided training regarding antibiotic, they go to the close the ASHAs.

I: Close to the people.

R: They go close to the people to the grass root level, because they are attached with everyone, mostly they are selected from neighborhood, to them means those who works at lower level and they themselves should take the responsibility that to make understand everybody and if sometimes campaign is organized then there may be some benefit.

I: who should do it you think?

R: Govt should do it. Everybody should do it; we are also having some responsibility.

I: In case of using or giving medicine what is your preference-brand name or generic name?

R: Generic name is preferred more because brand name keeps changing in that case when we see, we see the generic name.

I: Among two what is basic preference, what is mostly preferred by people?

R: People preferred brand name more because they don’t have idea about generic name and many cases generic name is large, to remember that is also becomes a problem for them, they also can’t tell the brand name properly.

I: In case or effect of medicine does it affect as brand name or generic name?

R: It should not be, generally it should not. If brand means same composition then how can it be? It should not be.

I: what is your opinion on broad spectrum antibiotic means antibiotic which work on many diseases? Means what is the use and availability of that?

R: It is not given here very much.

I: Here means outdoor

R: Nothing like that.

I: what is the availability in the market? Is it easily available, what do you think?

R: In our case we have to order, here it is not used much. There may be but I don’t know but here there is not much.

I: How much you are involved in case of counseling patient?

R: I am involved, it is not that I am not involved, mainly doctor does, in many case when there is patient for doctor and I am there then I help the doctor.

*Pause*

I: How frequently do you give antibiotic means I am talking about in absence of doctor you are handling the opd, what is the parentage, suppose there is 10 patient so among them how many?

R: See it depend upon what kind of patient is coming; I don’t prefer to give antibiotic cause to be true regarding antibiotic what should have like I don’t have that much that I can do prescription, thinking that I don’t prescribe because will it become resistant or something else so it’s better I say you wait two days, take this if it decreases then.

I: What is your opinion about combination antibiotic?

R: I don’t have much knowledge about combination antibiotic.

I: As a nurse what particularly you can do to combat antibiotic resistance?

R: Mainly when I am giving antibiotic to someone I should say clearly that this is antibiotic, I can mainly do the counseling, I can’t do more than that or when I was in the ward then when I gave a patient , I gave proper dose so that antibiotic works properly, don’t become resistance. Now I am giving to the patient, I am not present while they are taking, I can say him or I can say about the side effects means if you don’t take or if you don’t complete the dose then next time it might not work.

I: When you are asking someone after three days then how do you understand weather they have taken the medicine or not?

R: The medicine should not be there, the medicine is there, and if I say whether the medicine is finished if he says no then I will understand that he has taken and if say that it is remaining then I will understand that he has not taken.

I: What do you do in that case? Do you continue that or change it?

R: We send them to doctor that go and speak with the doctor.

I: Many times such cases come like they took medicine from outside then came to you so how do you handle such cases?

R: I ask them like did you bring the medicine with you, if they bring then we can see what they have used, and if don’t bring half of the people can’t tell the name then they say the colour , then we have nothing to do.

I: Then you from 1^st^

R: We start our dose from beginning.

I: If you want to say anything else regarding antibiotic or antibiotic resistance?

R: It’s ok.

I: Thank you.
