# Supplementary material for: “Without antibiotics, I cannot treat”: A qualitative study of antibiotic use in Paschim Bardhaman district of West Bengal, India
Source: PLoS One. 2019 Jun 27;14(6):e0219002. doi: 10.1371/journal.pone.0219002 (PMC6597109; doi:10.1371/journal.pone.0219002)
Supplement: S2 File — (ZIP) [file pone.0219002.s002.zip › S2_Transcripts/KAP 19.docx]

KAP -19

Occupation-IHP

Age-38

Higher Education-H.S

Work Place- Private Clinic

I: Before starting can you say something about yourself means where are you from? How long you are here? Since how long you are running your clinic?

R: I am a resident of Asansol, railpar babutalaw, I am practicing as a RMP nearly for 14-15 years, I don’t have any degree, I am telling you the truth. I am practicing for 14-15 years.

I: you are running you clinic here for 14-15 years

R: yes in this clinic.

I: So how many patients do you see per day on average?

R: Since morning to night average I see 15-20 patients.

I: 15-20 patients I see. You only work at this clinic or you do home visits also in your nearby places?

R: If there is a need to push a injection or doctor has written to put saline then I go to do that in the local area.

I: Ok, what medicines do you prescribe?

R: We don’t prescribe medicines. Actually we normally starts with Amoxycilin, other good antibiotic we start after seeing the specialist specially antibiotics. Among high antibiotic we are using Azithral now except that there is Amoxycilin, Amoxyclave 625 for the elders and for the kids also available. In that amoxycilin and clevonet are combined, we normally use that, except that Cefixm also. What else.

I: You said you use Amoxycilin and there are higher level antibiotic also. So in what kind of illnesses they are used?

R: Amoxycilin or something more powerful we use when we see there is cough or there is fever for long time.

I: How do you decide that what will be the right medicine for different illness or what antibiotic will work on that?

R: Yes we follow a reputed doctor that what he has given when we refer. It happens that we treat a patient for 2-3 days and if he don’t feel better then we refer them and when they come back we see the report what medicine has given and in that way we get a chance to learn.

I: You said that you refer in the case of major illness, so in which illness do you refer?

R: Major illness means fever is not going for long time then we refer to another doctor. Except that in pain means if there is abdomen pain then we don’t take risk, we refer. We work neat and clean here, mainly we see cough cold and fever.

I: What are the illnesses mostly seen here?

R: Here we see cough, cold and fever or if someone comes with loose motion, little bit stomach pain, we are mainly doing here first aid.

I: Do you write any test before giving medicine?

R: No I don’t write any test. There is no need of any test because I don’t keep a patient for long time. I give medicines for one to two days, for fever I give medicine for two days and if see he is not getting better then I say to visit another doctor where he can get better treatment.

I-So as there is a course of antibiotic so do you always give full course or half? How do you give?

R- No I give one by one.

I-You give one by one means when it’s a course of 5 days then how do you give?

R- If it is 5 days course then today I will give 3 dose, tomorrow 3 dose just like that everyday.[*smiles*]

I-I see and what do you tell to the patients after giving it [Antibiotic].

R- I tell to the patients to give me the feedback weather you are getting better after taking it, we do it like this. Means here patients are mostly poor, there are such patients those are not having money and come to take medicine, and this is a poor area so we give medicine in Rs 20, in that Rs 20 only we mix medicine and fees also. Whatever the patient needs we give normally and if needs antibiotic for 5 days then we give every day, like today, tomorrow and complete it. If I give whole in a day he will not be able to give money as well as he will not be able to complete his treatment.

I-In this case most of the patient come back or not?

R- Yes they come.

I-They come?

R- Yes they come.as I said I am seeing 10,15-20 patients everyday , they are repeating .

I-How means if you have given antibiotic to 10 patients then how many of them get relief at 1^st^ visit? can you please say how many patients continue[Treatment]?

R- Yes, see normally I don’t give antibiotic to every patient. As I feel like there is a case of respiratory, RTI, having cough then I must give antibiotic to that patient, without that it will not work so we give in that case. Normally if there is fever then we don’t give. In most of cases like that we give **Crocin,Rantac 10-20[***don’t understand the word***] .** I only give antibiotic when there is a patient [*sound of horn*] with cough.

I-You always give medicine or sometime you write prescription also?

R-No, I don’t write prescription. I give medicine, don’t write prescription.

I-So you are having a little medicine with you?

R- Yes, we buy medicine from asansol.

I-Where do you buy from?

R- From market.

I-Market , you mean wholesale?

R-Yes , from wholesale market.

I-Ok, now tell me one thing that what do you do with the medicine which will expire soon? I mean the medicine which will expire next weak.

R-We throw them away.

I-where do you throw them?

R- We throw them in the dustbin, I mean we have place, we destroy that in that way so no one can use it. See there is a big pond *[indicating back to his clinic*], who will take if you throw there?

I: I see, means you

R: We throw them in a way so no one can use like if it is a tablet then we open and destroy, if it is a syrup then we open the cap and throw it away.

I: Hmm, Those patients who are coming to you directly ask for medicines by name or say about the disease?

R: No, it’s not like that.

I: It never happens?

R: No, they never ask for medicines, whatever we give they have to take.

I: Do patients directly ask for antibiotic?

R: No, they don’t ask for antibiotic.

I: Did ever someone ask about strong medicine, healthy medicine or by any such name?

R: Yes, there will be 1 among 100 patients. Now they are not coming to us, now there are lots of medical shops so they take that [medicine] from there. Normally what we saw is cough, cold, fever and we use for that.

I: Can you say what antibiotic resistance from your point of view is? What will you say? What is your opinion about this?

R: Yes I will tell. I think if you don’t take right doses like the course was for five days but did not take or if you take overdose then resistance can happen.

I: What is this resistance?

R: Medicines don’t work on body.

I: Medicines don’t work on body?

R: Ya

I: And meaning do you think, that as you are practicing as an RMP, that this could have any effect on antibiotic resistance? Or that you can do something to stop it?

R: Yes it can, If we all RMP understand the dose of medicine, how many days it should be taken, it should be taken minimum 3 days. We mostly know about antibiotic, we did not do any class still we say by guessing that normally antibiotic should be given for 5-7 days. So we follow like that still we don’t give to everyone, we give only to those who need. If we give then always in full course. It is not like that we give for 2-4 days. There are patients who do not complete the course by themselves. Big doctors [meaning MBBS dr] prescribe for 5 days and if patient is not taking for 5 days then we cant do anything.

I: You were saying some times ago that you give one day’s dose.

R: [*Starts speaking before he finish*] What I said, there is a reason behind giving one day dose, I give today and said to come back tomorrow just to understand if the patients are coming means do the patient are worried about their illness or not meaning they want to be cured. It is carelessness that I took the medicine but did not it, so we call them to know that I gave medicine for one day and did them use or not. We ask and give medicine again for that. We can give not only for one day but also for 5 days, we don’t give thinking that they don’t loose it, that’s why we give for morning and evening.

I: Did it ever happen like a patient needed a course of 5 days and you gave for 2-3 days and after taking that he was feeling better. So when you offer medicine for rest of the days does he take or refuse?

R: No, we make them understand that they need to take the medicine, that’s the course. For example cough, for that Azithal is needed for three days, daily one. If someone takes less than that then it will not work [*a patient came and said something*] . If you don’t take for three days it will not work, you have to take minimum three days. Some patients take.

I: How do you know that patient took the medicine that you gave?

R: Some patients say. See whatever patients are saying after coming back we have to believe that he took the medicine. I myself or someone else is not going to check at his home. As they say that they took for three days we believe.

I: So can you please tell us the full process of how you treat patient from coming to you till they leave with medicine?

R: I don’t understand what you mean?

I: Please tell us the total treatment process.

R: Patient cone, I ask what happened; why he has come if there is cough, cold, fever. After listening the whole thing I come to result like he is having cough, cold, fever and decide what should be given what not. After listening the whole thing we decide what we should give or what not.

I: So how do you decide where to give antibiotic?

R: Yes we decide, like someone came with severe pain, I felt it can be some old pain, or may be this pain is for food poisoning. Many times it happens that we take some wrong food at night and suffer from loose motion so it is obvious that there is food poisoning so we use medicine for food poisoning.

I: Does cost become a factor in deciding what medicine should be given and what not?

R: No

I: It doesn’t matter?

R: We use according to our knowledge, and it is not like that we have done wrong.

I: do you ever consult before giving [antibiotic] or you give according to your experience?

R: According to our experience

I: Understand. Suppose a patient came to you asking for antibiotic like I need that medicine then what will you do?

R: What the patient says does not matter- there are a lot of patients who just go to the chemist shop and take it. The people who ask for medicine do not go to the doctor and ask. The only people who come to the RMP- who- the ones for whom the medicine worked who come regularly- they are the only ones who take medicine according to what the doctor says.

I: Ok, so it never happens?

R: No they don’t come and ask like give me this or that. Now a day there are lots of chemist shops, so they push themselves.

I: If it happens in future like someone come and ask for medicine like I need that then what will you do?

R: No, I will not give like that.

I: You will not give?

R: No, I will not give.

I: Why will you not give?

R: I will not give because I don’t know where will he use that, those that has the need will come for themselves not for others, understand. I don’t work like that.

I: Always the patient come or some of their relatives?

R: No, patients come.

I: Patients come?

R: Yes patient come

I: If such person comes then?

R: Patient?

I: Means someone come for his daughter.

R: No, if such people come and I know that he needs then I also visit home to see patient.

I: So it does not happen like you give medicine without seeing the patient? Sometimes their uncle comes, sometimes father comes.

R: No, I never do like that; I make me trouble and visit the patient at home and decide after seeing the patient.

I: Ok tell me one thing, I will tell you some scenario and tell me what you will do in that situation. Like you came to know that one patient has taken wrong dosage.

R: Ok, if one has taken wrong dose

I: Antibiotic

R: Antibiotic? I have to know 1^st^ for how many days he has taken the dose. If he had to take for 5 days and he already finished that then I will stop medicine for 5 days, no medicine for 5 days.

I: If someone comes and says that he does not have money and having [*sound of horn*] such problems then what will you do?

*Paused the recorder*

*Continue*

I: If a patient comes with complaint of cough, cold and running noses then what will you give in this situation?

R: We normally use **Lemolate** for cough and cold.

I: Ok, antibiotic?

R: No

I: If there is fever?

R: It is included in that, there is everything in Lemolate.

I: In Lemolate

R: Lemolate

I: Lemolate?

R: Yes, or **Coractor plus [check spelling]**

I: No one is antibiotic among these two?

R: Or **Fabrex plus.** We use three medicines- fabrex Plus, Corector Plus, Lemolet .we only use these medicines.

I: if fever doesn’t come down?

R: Fever comes down in that.

I: Comes down? If there is loose motion with or without vomiting then what will you give?

R: For loose motion we give **Metrozil 400** for adults and syrup for kids.

I: Only that? Do you give **ORS** or not?

R: Yes I give liquid.

I: You give? With that or 1st ORS or Metro 1^st^?

R: No, I give with that. [Metro and ORS at a time]

I: You give at a time. Suppose there is stomach pain?

R: If there is stomach pain then I give pain killer.

I: Pain killer? Is there antibiotic in it?

R: No

I: If there is rashes in hands?

R: We give lotion for that.

I: Lotion. Do you give tropical creams which are applied in body; do you give antibiotic in that?

R: No

I: You don’t give?

R: No

I: so you must have heard about antibiotic resistance?

R: Ya

I: so what do you think, what do we do to decrease this in community? How can we decrease it?

R: Yes I think antibiotic should not be given and taken for every single reason, understand. If you are having problem or any doctor has prescribed then only you can give and take antibiotics. Normally we use antibiotics very infrequently. When antibiotic is needed that this patient needs antibiotic then only we give.

I: Anything else?

R: Before giving antibiotic you should tell the dose means for how many days you have to take, overdose should not be given. Without making them understand it should not be given. If you are giving without making aware then it is harmful for body, it affects, and patient must understood that he has to take full course, if he takes half course then it’s a loss for him.

I: so do you think that patient understand this?

R: Yes [*happily*] they understand. If people like me tell them they understand.

I: so you think that you get time to make them understand?

R: yes I give full time to the patient. there are not a lot patient at morning and evening, so I tell them.

I: Ok tell me one thing, there are formal doctor like MBBS, pharmacist, Rmps who are responsible for giving antibiotic. What is their interaction when giving antibiotics? Meaning what is the relation among them?

R: Yes, I saw reputed doctors also don’t use antibiotic very quickly.

I: what do you mean?

R: Bade doctors also don’t give antibiotic directly, we saw they 1^st^ understand the disease then only give antibiotics, means for normal fever they giove normal medicines. If they see it is not working then the give antibiotic. It is not like that they give antibiotic at 1^st^ visit.

I: And pharmacist?

R: No we not by chance. We give antibiotic very fearfully, we are afraid of antibiotic, not with anything else.

I: The pharmacist those who are sitting at shops?

R: No, no one do like that. We told them many times to give everything [*except antibiotic.*]

I: So what is your thought on patients those who are directly going to pharmacist, shopkeepers directly? Why do they do so?

R: They don’t go to buy antibiotic directly, they go for other medicines.

I: I see, so they don’t go to buy antibiotic.

R: No

I: What do you think who are going to RMP and who are going to MBBS doctor I mean hospital?

R: Those who are poor patient [repeats] earning Rs 50-100 daily go to RMP for cough, cold, fever , latrine. Those who are earning Rs 400-500 daily, can give the fees go to MBBS doctor. See those who are earning Rs 50-100 daily, if they give fees Rs 200 then they will not be able to take medicines. So they think in that Rs 100 they have to run their families so they come to us, take medicines for Rs 20 and with Rs 80 they run their family.

I: So when you are giving medicines do you give generic medicines or brand name medicines?

R: No, mostly I use generic medicines.

I: Why so

R: Because the price of generic is less, sometimes I use brand name also. If I feel that this patient needs special medicines then I prescribe 2-4 antibiotics.

I: So what do you mean by special medicine? How do you find out?

R: I understand because I am practicing for 16 years so having long experience, so I understand which is good and which one is not. Those who can buy take. Now there are fair price shops at hospital where generic medicine are sold so it’s not that they don’t work.

I: Do the MRs visit you? I mean Medical representative.

R: Yes they Come.

I: They come?

R: Yes

I: What conversation do you have? [*Children chattering*]

R: When they come for advertising medicine they say about the full package like what is the dose etc. for example they if they say about**Crocin** then they will say what is combined with that, if there is **Paracitamol** in it or not, dose, price , company everything they say.

I: So how it affects your decision to give medicine I mean which one to give?

R: No, we get to know a lot from them, if I say they are our main teacher.

I: They are main teachers? So you mean after they come you come to know which medicine

R: Yes the dose.

I: So do you follow what they say

R: We hear, we understand, we get a lot of help. There are lots of new medicines came to market, reputed doctor knows about it but we don’t get any information about that. So when they come they tell about complete range which helps us.

I: In Rs 20-30 how do you manage the cost of brand name medicines as well as consultation fees?

R: No, I said you earlier that I write the brand name.

I: Oh yes you don’t give, just write.

R: Yes I don’t give. Actually there is nothing like fees here, if I am giving 3-4 dose for Rs 20 then from there we get Rs 4-5, understand. After that if I feel that there is something good to give then I write, I don’t get anything but it’s good for him.

I: And how much confidence do you feel while giving medicine that this is the correct medicine?

R: Yes in this case I am practicing for long time so I am confident, I learnt,[] following others, from MR, by following reputed doctor.

I: From who do you learnt more?

R: I think I learnt more by following reputed doctor.

I: Ok, so where do you see bade doctor?

R- We work with bade doctor like a patient comes to me and don’t get relief from me then I take the patient to bade doctor and say to check so I follow what he gives then. I check what I have given and what he has given. 2ndly we are guided by MRs.

I-You said about another doctor so do you take patients to another doctor?

R-No, we send the patient to them, there are lots of doctor in the market, so we send the patients to XYZ doctor, so when they ask them who has sent, they tell our name and the doctor comes to know that there is a doctor who is practicing. If a patient goes to any surgen doctor with some cut or abscess then they operate and if write 10 injection then also instruct the patient to visit me to push it. So in that way patients also get help and we also earn Rs 5-10 for injecting, so our work is also done. It is helpful for patient that they don’t need to go here and there, their work is done at a place and if comes to me I push the injection.

I-So some small thing like stitching and saline are done here?

R- No we don’t do risky work like stitching, we know it. If a doctor writes then only I do saline.

I-If it happens that a patient needs a medicine and you don’t have that antibiotic then what will you do?

R- I say to take that from a chemist shop. If I don’t have a medicine or doctor has written which I don’t have then I say to take that from outside but must take that. I say for the 2^nd^ time not to alternate the medicine that doctor has written.

I- Did you ever see a patient who is resistant in some medicine? Some medicines, antibiotic don’t work on him?

R-Yes there are patient, I have seen. Suppose a patient come to me with cough[*Sound of horn*] and I gave **Azithral** 500 mg but it did not work. So I wanted to know why it did not work then I came to know that it was to be taken for 5 days or 3 days but he did not take in between one day. So it’s also harmful.

I-Ok so what did you do in this situation when you came to know that?

R-Yes as he said that there was a gap for one day so I finished that and made a new course for 3 days.

I-You added a dose with the previous one?

R-No

I-I see

R- As there was a gap for one day, I told to gap it for another day then I made a new dose.

I-Means there is no test to verify if there is resistance?

R- We don’t come to know, but we know that if you take a gap for one day then it will not work, so we say to make another gap and to leave it totally, don’t take medicines for 2-3 days, after 2-3 days I give one new medicine.

I-Means if you gap for 2-3 days or few days then it will work again?

R-Yes, it will work.

I-there are risks of antibiotic so can you say what these risks of antibiotic are?

R-Yes there is risk if it does not suit. We have seen when the reputed doctor says to test it in skin 1^st^ before injection, because if it is given and there is problem like swelling then he should not be given that. If you push it without testing then there is risk even he can die.

I: So how do you see it means keeping in mind both[Risk and benefit] should it be given or not?

R: Risk or benefit, risk is that where we feel we test before giving [*horrible noise outside*]

*[Mohit instructs Rina to hold the recorder closer to the interviewee]*

That whether there will be side effect in his body or notlike in that we test IM , and when the bade doctor writes many antibiotics then they write to test in skin and after that to give. When I push it if I found that there is swelling after giving that then I don’t give it. Then we say the patient that this medicine will harm you so you can’t take this as given by doctor. Then we send them to someone senior to us to understand that. It’s not that I give it by taking risk, I stopthe treatment there.

I: Ok so you said about injection but there are few antibiotic tablets and how do you test that?

R: Yes there isfew medicines given by doctor react on body so we send that patient to specialist to check why it happened. There are lots of drugs which react on your body but 1^st^ time you have to give so that he gets relief. So next time we send them to understand why it happens to his body. So after going there we come to know that there was some skin problem or may be other for which it [drug reaction] was happening.

I: [*Interrupting him*] where do you send to check?

R: XYZ any doctor.

I: To any doctor?

R:Yes I mean I send to bade doctor. We write and say the patient to say that he has taken this medicine and it happened for taking that, so you go and understand why it happened,. Ehen he return back then I get to know or I ask the doctor like what happened, so we come to know that he had problem of skin and this medicine will not suit on him. There are lots of medicine which reacts on body, you know sulfur group, there will be side effect of sulfur group medicine like [*don’t understand the word he said*]**Ceftron** and many more which directly harm you, so we understand that he should not be given this medicine. In that way patient is safe as well as me too safe.

I: There are few medicines which work on lots of diseases which we call broad spectrum antibiotic, so what is the use of that in your practice?

R: We give them. There is nothing like

I” You mean you give?

R: Yes, We give means we prescribe. We prescribe the high antibiotic for minimum 5 days, we write the patient to take for 5 days, and they take.

I: For which condition you give that? How do you know that you should give that only? Which situation? [*Sound of horn*]

R: Mostly if I see there is cough then I give antibiotic like Azithromycin 500 mg for five days or three days. I have seen that most patients are getting relief with that.

I: And the combination antibiotic?

R: Yes, combination means which are combined?

I: Yes

R: Yes after that I give 2^nd^ antibiotic like **Sensiclav** 650 mg

I: What name you said?

R: **Sensiclav**or **Amoxiclave 625** mg

I: Amoxiclave?

R: Amoxyclave is combined by two- Amoxycilin plus Clavonet, we prescribe that and patient take that.

I: Means you give these also?

R: Yes I give when I feel it should be given, very rarely.

I: So it is not used mostly?

R: No, we use it less. If total patient is 100 then we give 2, 4 or 5 patient where we feel it is needed otherwise we use normal medicine. [*Phone rings*]

I: Ok so according to you what is the thought about the RMP those who are not giving antibiotics

[*Phone rings so we paused for few minutes*]

*Restart, again continue*

So as I was saying what does the patient think about the RMP, Doctors who are not giving antibiotics?

R: What patient will think? Depending on you patient has come for treatment. Now it depends upon doctor how much he is having knowledge, is not it. He doesn’t know about what you are giving. When patient comes to you or goes to any bade doctor he doesn’t know what doctor will give, he will use whatever doctor has given.

I: See there are few diseases where you don’t need any medicine which will cure by itself like you need to rest. So in this case if doctor don’t give anything what patients normally thinks in this situation?

R: Patients don’t think anything; they follow whatever doctor has said.

I: So you have seen like this in you experience?

R: Yes, I see in this way.

I: Like you only gave paracitamol and no antibiotic.

R: No, he does not know what ABCD has happened. But in my profession I have to work with honesty, whatever medicine is to be given to patient must be good, understand. We fixed our rate Rs 10-20 so patient gets lots of benefit in it and we are practicing for long time. Patients never say like give me this or that. [*Noise of car]*

I: What about the training?

*Paused the recorder*

*Restart*

Do you use or follow any particular guideline about antibiotic use?

R: See I have said about antibiotic guideline that we get help from MR, we ask them about many things like dose or what are combined, and we try to understand that. So we get guideline from MR.

I: Means do you follow any particular book or jurnal?

R: No, we have a old book called CIMS.

I: CIMS?

R: Yes we get a lot help from that, from the time we got mobile we don’t feel need of that.

I: Mean you read from internet?

R: Yes, from internet we get help.

I: Do you follow any website or where do you see?

R: Yes, we see in Google.

I: You see in Google?

R: Yes

I: So will you take part if there is any trading or workshop or something like that regarding antibiotic?

R: Yes, surely. I attend lots of meetings called by Mr.

I: Where, which meeting?

R: Big companies like **Cipla, Ranboxy** invite me and the speaker is bade doctor. So in that we ask according to our group so that we can learn something. They also say they if anyone needs to ask something or wants to know then to stand up without any hesitation. So lots of people ask, they understand, and they also explain well. We make note on our copy that it is important information for us.

I: What is your interaction with other RMPs mean do you discuss what they are giving or what should be given?

R: see all the RMPs meet at a place.

I: Tell me more about it.

R: we are having a association of RMP, though not approved by Government still we meet at a place if there is such programme. If a company [Medicine company]organizes then we all meet also.

I: The association is of local level I mean it belongs to here?

R: No, it is of Burdwan district level.

I: You mean it is of district level?

R: Yes

I: How many times the meeting is held?

R: Not many times, 2-3 times per year.

I: So you go regularly?

R: Yes whenever there is a programme I go. The committee is not known like a organization, it is made so that we can meet at a place.

I: who organize I mean who arrange all these?

R: we all locally do it, no one from outside.

I: Average how much people come at a year?

R: Average 40-50

I: From the whole district?

R: Not from district, from Asansol.

I: Ok, so what will be the total?

R: I can’t say about the total, 40-50 persons always meet.

I: Does it ever happen like you refer to some doctor and some patients come to you after visiting another doctor?

R: No, I don’t get that kind of patient. Refer patients comes to us like doctor has told to follow mostly inaction cases like we have to push that.

I: Not the refer case, [*someone shouting outside*] I mean someone is seen by other doctor but not cured then came to you.

R: Yes, comes

I: Such cases come?

R: Yes come but I don’t treat. We make choice like which doctor will be good for him and then send the patient there by giving name and address.

I: Ok so you don’t treat such patients?

R: No, I don’t treat, never. I can’t do that thing so why to do that forcefully? I work only on which I have knowledge; I never tried to do more just for that I have to do it in a good way. It is not that without knowledge I give him.

I: do you want to say anything else regarding this means to reduce antibiotic resistance at community or in India?

R: Yes yes, in order to reduce, I would like to say one thing that to reduce [resistance], we have to get all the RMPs together in one place.

I: ok, please say something more about it.

R: see first the illness comes to small doctor [RMP]. F or cooking good food you need a good cook, the cook should know how to cook. If we bring cook with less knowledge then the food will not be good. So I think that the knowledge we have should be updated. It should be understood that antibiotic should not be given directly. So for this, there should be one room, meaning some arrangement, a meeting should happen, a program should happen, where they are treated- meaning those people, meaning who you are giving medicine to and if you don’t know about the dosing, so if we don’t know about that, explain to us if you have knowledge. If not, call the doctors and they have to give us some training. But I think something is missing.

I: Who should the training be given? To the RMP or People?

R: No to the doctor 1^st^, we should understand because we don’t know what other doctor is giving. I think that there should be a good meeting at local level. As I said when we gather at meeting and everyone ask questions there. These types of meeting are held sometimes. This kind of programmes should be held ones or twice per month. There should be programme twice per year where everyone gather and discuss so that we understand something.

I: What do you think who should take the initiative?

R: You see the government, there are health department in every place, we also see at local health dept that people working over there are not having knowledge. So government should take care of that, they should recruit the person or doctor who is having knowledge on it so that people are benefitted but not get harm. [*Pause*] If crime increases then there is police to control it, it is also like a crime. Don’t think that this is small thing. So I think there should be a meeting for doctors so that we understand whatever we are giving to patients is proper dose or not. In that way the body is getting harm. I don’t know the medicine should be given for 10 days or 5 days, still I am in this practice for long time so now I think there is a change than earlier. To make it better there should be a meeting in every 6 month. In this way this [resistance] will decrease in the community.

I: as you said there is a change so can you say what has been done till now?

R: Yes, things have been done. See those who are RMP don’t have any degree or certificate, they are practicing after learning from someone. But as they are working since long it is under them that now antibiotic is easily available so it helps to follow bade doctor. Now there is a need of guideline.

I-There is a need?

R- Yes there is need so that we get more help. As I said about my working style, I refer patient whom I can’t handle because there should not be any harm to human body, there should not be any misshapen, understand. That’s why it’s my responsibility to tell him the right path. Others are also doing so, it’s not like that, when we meet in meetings we discuss about everything and we find that it is also helpful for our patients. Now I feel that in this line the more you learn is less. Today you learn one thing, tomorrow there is a new disease, a new medicine is coming so who will tell to a RMP about that? If there are such kind of meeting then it will work, if there is a new medicine we get to know from MR, if MR don’t come then how will we know?

I-Do you like to say anything else?

R-What else I can say, I answered whatever you asked.

I-Ok, if you want to say anything by yourself.

R-I want to say that whatever initiative has taken, if you go to everyone then those who don’t have knowledge or have less knowledge regarding this will increase. Today you are recording and speaking and if you keep into file then it will not work. See I am walking through the road but is it right or wrong others can say that. So there is a need of guideline for this and as I said there should be a programme at least once in 6 month anywhere in Asansol belt. You some people to arrange this and for that you need to go everywhere part by part.

I-Ok, Thank You.
