# Supplementary material for: “Without antibiotics, I cannot treat”: A qualitative study of antibiotic use in Paschim Bardhaman district of West Bengal, India
Source: PLoS One. 2019 Jun 27;14(6):e0219002. doi: 10.1371/journal.pone.0219002 (PMC6597109; doi:10.1371/journal.pone.0219002)
Supplement: S2 File — (ZIP) [file pone.0219002.s002.zip › S2_Transcripts/KAP 262.docx]

KAP-262

Age-27 years

Gender- Female

Block -Raniganj

Occupation- Nurse

Highest education- GNM degree

I-What is the name of your work place?

R-Ballavpur rural hospital.

I-Ballavpur rural hospital. If you say something about the OPD and IPD setting over here.

R-Setting means what will say means how many patients do we get everyday all these?

I-Yes

R-In OPD the patients who are coming they come regularly.Some patients get admitted from our OPD. Indoor is for emergency patients those who are admitted here. There are many departments in our OPD like Ayurveda, homeopathy plus our doctor at OPD means there are every department here. With that there is blood test, ICTC test, sputum test.

I-Those who comes to outdoor mostly come with what kind of illnesses?

R-Those who comes to outdoor means there are every kind of patients. But here most common problem is diarrhea; COPD means these are mainly seen. Otherwise there are every kind of patient like burn, fever, every kind of patients come.

I-those who are coming to indoor means who are mostly get admitted?

R-Mainly diarrhea and COPD. Except there is labor [pregnant] but it is not a disease so am not telling about that other than that I said fever, cough, cold, weakness patients are admitted.

I-If you can say me how many patients come to OPD and IPD? Average.

R-I can’t say about outdoor but it varies according to season like in winter it is less and in summer it is more minimum 250-300 patients per day and in indoor it is same like varies according to season, now it is winter now our average patients are 25-26, but in summer or rainy season it is 45-50 as it is a 50 bedded hospital.

I-Do people come with skin problem?

R-There are not many people come with skin problem but sometimes come.

I-If they come then what do you prescribe?

R-Doctor prescribes that.

I-Generally what is given?

R2-Cetrizin is mostly given.

R-For skin problem? Here for skin problem we means without doctor nothing is given,

R2-Skin problem, allergy is also skin problem.

R-If there is allergy what is given in allergy is Cetrizen mainly that is in outdoor.There is one.

I-I did not undersytand , please say again.

R-For allergy Cetrizen is mainly given, sometimes Decadron is given. These are given.

I-Is any tropical antibiotic given?

R-No.

I-For putting in the body any ointment?

R-Yes one ointment is given. The name of that, what is the name of that sister? [*Asking another nurse*]

R2-Micronazole

R-Yes Micronazole

I-Is it antibiotic?

R2-No that is anti fungal.

R-Anti fungal

I-Thank you. If there is any minor surgery done here, any surgery process?

R-Only the injury repair is done.

I-In case of injury is antibiotic prescribed?

R-If the injury is deep then obviously they prescribe antibiotic. If it is not deep then antibiotic is not prescribed.

I-Can you say what antibiotics are available at your set up?

R-Here there is Ceftriazone, Ceftrizone sulbactum [not audible] sometime here Ceftazidime is supplied and Amikasin, Gentamicin all these are given.

I-What antibiotics are mostly used?

R- Ceftriazone, Amikasin are mostly used. Ceftriazone, Ceftrizone sulbactum, Amikasin there are mostly used.

I-For which disease these are mostly used generally?

R-Diarrhoea, Ag, fever.

I-Is there any facility of testing means microbiological testing facility in the hospital set up?

R2-Only the routine test are here.

I-What are there among routine test?

R2 -ARBS, SBTC, urine test, sputum

I-Any culture and sensitivity test?

R-No no

R2-They are sent to outside through our hospital, here it is not done.

I-If you can say a number roughly like how many patients are sent for such test, sensitivity test or culture test, if you can give a number?

R-That is very less.

*Paused*

I-Yes so how many patients are sent for sensitivity test?

R-It varies, what will I say?

I-A rough number means a average.

R-Means if there is 10 patients then we will send 4 patients.

I-For culture test?

R-Yes

R2-If it falls under COPD then are send.

R-That is for sputum culture means I said that if there are 10 patients we will send 4 patints among them. It depends upon patients.

I-If someone comes with respiratory illness or diarrhea then how do you treat that?

R-If respiratory patient comes then 1stly we give deriphyllin that is supplied, now a days we are having supply of Budicot, Duolin which are used for Nebulisation then there is supply of Hydrocortison

R2-Tablet is there

R-Tablet means those who comes with acute probem

I-So in this case is antibiotic prescribed?

R2-Yes it is done

R-Ampicilin

R2-Here **Ampicilin** is given. If it does not work then Amoxiclave is bought from outside, that works good in Lower tract infection. If it works with that then it is not needed.

I-In that case is there any cultural routine test or stool test or sputum test is done?

R-Sputum test is done.

R2- Sputum test is done.

R-Sputum test is done in case of every COPD patients.

I-Other culture, routine culture?

R-Blood

R2-If there is fever then blood test is also given.

I-As I said earlier among 10 patients how many patients are given or how many patients are given antibiotic those who come with cough, cold? How many patients are given antibiotics?

R2-Antibiotic is given to everybody.

R-To everybody.

R2-If come with cough cold then antibiotic is given to antibiotic. If simple respiratory distress then

R-Deriphylin injection

R2- Then he is given nebulization but if there is cough then he is given antibiotic.

I-When you are giving antibiotic to someone then how much you are involved in that?

R2-Totally involved because you have to know everything about the medicine whether it is dose or side affect, everything.

I-When a patient need antibiotic then what do you think?

R2-1^st^ skin test is done. Which are tested that according to

R-No 1^st^ we see the advice what the doctor has prescribed. If prescribed then I will check the expiry date, dose, root of the antibiotic. Then we will give according to the instruction. Then before giving we will check the sensitivity of the patient whether it is suiting the patient.

I-Sensitivity test is done for everyone?

R-Obviously.

I-When you are giving antibiotic?

R-We wait for 15 minutes then if there is no reaction, it is given.

I-That test is available here?

R-Yes

I-In absence of doctor if a patient come then what do you do?

R-Meaning, say again.

I-Suppose a patient came and there is no doctor in the emergency, then what do you do?

R 2-It varies upon patients like what emergency the patient have.

R-But doctor is always here as it is a rural hospital, there is always doctor here.

R 2-It happens like doctor went to his room

R-He go to bathroom

R 2-Here we can make wait the patients who are coming normal but if there is emergency like respiratory distress then whatever we can do we do like nebulized, providing oxygen, after that medicine or injection is given as per doctor’s prescription.

I-When you are giving or prescribing antibiotics to one then so you counsel the patient?

R2-Yes while giving antibiotic we say actually they don’t understand what the antibiotic is, while testing in skin then it is said that there will be little bit pain.

R-We say them if there is redness in that place or itching or swelling then say to us. We say that.

R 2-One more thing is said that you are having the infection so the medicine is given.

R-We also say that the dose like 5 days or 3 days to complete that. We say that.

I-Suppose patient come and already taken antibiotic from somewhere then do you counsel in that case?

R 2-No, 1^st^ we need to check what antibiotic he has taken, if it did not cure with that then there is no use of giving that . If the doctor prescribes anything then it is given.

I-Do patients ask antibiotics by themselves like give me antibiotic?

R-No, very less.

R2-Actually many patients comes from outside , have seen to another doctor, outside doctor has written injection for some days then we don’t give directly, the doctor write and sign then we give. If there are patients coming from far and taking im like gentamicin at morning and night then they are given that. It is not given by us.

R-Means patients also don’t say by themselves like give me antibiotics. If doctor orders then only happen.

I-Patients coming to your clinic, I am saying about opd, so there among 10 how many patients are given antibiotics?

R2-You mean indoor patient?

I-Outdoor patients.

R-Outdoor patient, among 10 if, it varies upon what kind of patients are coming.

R-6-7 patient.

R2-COPD I said if there is cough then everyone is given, and if there is only breathing problem then deriphyllin or nebulization is given. If there is fever then it is given to everyone who comes.

I-Patient means doctor don’t write antibiotic to everyone. So did you get any feedback from patients like they did not give me antibiotic, I needed it.

R2-Many patients come and say that. Those who know more like I have learned a lot, give me this or that, but this is not right, what the doctor thinks good or what he prescribe, taking that is right. Not many, very less come like that.

R-No, they don’t come, it is very less.

I-If you can tell the number?

R-1-2, means very less.

R2-1-2 among 100, not more than that.

I-How much time do you spend on a patient to counsel. Like you gave antibiotic to a patient then how much time do you spare counselling him?

R- 5 minute

I-In that case what is the reaction of the patient?

R- Patient listen, if we say to tell us if there is any problem and they listen to that, patient party also cooperates, so we don’t face problems.

I-The prescription process I told, or doctor prescribing and they are telling you so how much patients are involved in that, means you gave full course but they don’t take , if you have such incidents?

R-Here patients are very punctual means whatever we say they do like if we say to take for three days they will take three days and may be more than that but they listen, what is dose of one they have to complete so they listen that.

I-Did you see patient has taken improper dose?

R-No no, at least here I did not see. Sometimes they take overdose.[*laughs*]

I-Overdose meaning, what kind of?

R-Means they continue, like they bought and one is extra then they say sister give that but we don’t give because there is a dose that is of for 5 days or 3 days or 10 days. Mainly for 10 days it is not given, it is mainly for 5 days. So I am saying that we give dose wise, the patient don’t know what may happen if the dose is increased or decreased, it is not known to them, so we say them then.

I-So patient comes with overdose?

R-No overdose means as I said suppose they bought and there is one left then they say sister one is left, give me that injection and I will go home, it is like that. We don’t give that. Then we say change it and other given medicine or whatever you want. That is not given.

I-How frequently do you get Self medicated means I took from outside, the number?

R2-Means prescribed by other doctor?

I-Taken by oneself.

R-No, taken at home, that is very less. Roughly you can say 5-6 patients.

R2-If there is something small happen they come here.

R-If they don’t sleep at night then also they come like why I am not sleeping? These are the thing here.

i-Someone is not able to afford treatment or can’t buy medicine.

R-That is given from here means they say to doctor like doctor I can’t buy means what we have will work less in those diseases but then it is given from here.

I-The whole is free?

R-Yes

I-The given tests are also

*Paused the recorder*

*Continue*

The test is done at free of cost if they cant afford?

R2-No, its half means half or may be less than the total that I don’t know exactly.

R-The brother at pathology can say that.

R2-They are sent there and speak there.

I-So there is discount?

R-Yes there is. It is done with less, little less.

I-Suppose a patient needed antibiotic but was not prescribed then how you deal with that like antibiotic was needed but not given at that time. Later he was given or not?

R-Suppose a patient admitted and antibiotic is not prescribed may be doctor understood that it will work without it so only other medicine given. Here everyday there will be a round, we are always in touch of patients so if we see that it is not decreasing in 2-3 days then prescription is checked and antibiotic is started. Antibiotic is given from 1^st^ but if it is not given then later it is started. May be not 1^st^ day but it is started at 2^nd^ day.

I-What is your view on delayed prescription means if a patient comes with COPD or with other thing?

R-No may be patient don’t give us proper history, if they don’t give proper history then why will we do, many times patient only say I am having breathing problem but did not say that I am having cough or there is blood with cough, they don’t say this like they say half and not the other half so it is not possible for us to understand. So in that case doctor prescribes in that way.

I-What I want to say is delay prescription like one patient come and after seeing 1-2 days give medicine.

R-No, not 2 day it’s after one day.

I-That is one day?

R-Yes, one day

R2-No now they don’t wait, if see there is such problem then antibiotic is prescribed, because we already have that available, if they see it is needed to buy from outside then they wait for 1-2 days.

I-I see. According to you what are the risks and benefits of antibiotic use?

R2-Benefit is that the infection or bacteria which is occurring will come under control and the risk is there may be sensitivity for which something big can happen.

R-It may be life risk.

I-If you can say what kind of life risk you are talking about?

R2-See there can be anaphylactic shock in case of any medicine, Understand. That can cause death also. So skin test is given, in which skin test is applicable then skin test is given or where iv test is applicable it is given. If there is no reaction then only we give.

I-You said about the side effects of antibiotic so can there be any other side effects of antibiotic?

R2-Any other side effect?

I-Yes

R2-See side effect means

R-There can be loose motion

R2-Loose motion, vomiting these is common side effects.

R-These are common side effect.

R2-Other than these there may be fever after giving antibiotic.

R-Nausea, vomiting these are common.

[*Someone asks something, they answered*]

I-How serious do you think is drug resistance?

R-After giving skin test if there is any reaction for example Ceftriazone is given and we see reaction after skin test then it is changed and other available antibiotic is given, if it suits then it is given but if there is any reaction then it’s better not to give, there can be any kind of life risk, then it will be difficult to understand where the life risk is happening with what.

[*Phone rings]*

I-I was saying about antibiotic resistance so if you find one resistance as you said sensitivity test is done so in that case how you manage case?

R2-Suppose there is some allergic reaction then Decadron or cetrizin is given according to their availability.

R-No, the 1^st^ thing is we check 1^st^ whether it is suiting or not. If it does not suit then we don’t give, we cancel it.

R2-No it happens many times that they are given 1^st^ dose and it don’t react in skin test then after 1^st^ dose there may be something then Decadron or Cetrizin is given and we stop the medicine[antibiotic].

I-This is about reaction; I am speaking about resistance, antibiotic resistance. Drug doesn’t work, you gave one antibiotic and it is not working.

R2-See actually we give for 3-5 days, if we see it is not working then it is changed and if there is any better antibiotic available here then that is given, if not then it is said to patient party to buy that. If then can afford its good otherwise we manage it from here.

I-As a nurse how does you see antibiotic resistance means what are the reasons of it?

R2-Causes? Why it is not working?

I-Ya means causes of resistance.

R2-One is if the health is poor, 2ndly if there is Diabetes

R-If regular means one patient is admitted and taking regular antibiotic in that case also resistance happen. [pause] If there is any problem means he is having any severe disease.

R2-Mail is Diabetes Mellitus, in this may diseases get delayed to be cured.

R-Many times we don’t get to know if there is any unknown history.

I-So from your place what steps will you take to combat antibiotic resistance?

R-At 1^st^ I will tell the patient about antibiotic, why we are giving that many times they take for two days and go home in that case I will tell to complete the dose and go home or if he takes at home then also I will say to complete the dose weather it is of 3 days or 5 days. If he faces any problem after that then I will surely tell him what he needs to do if the problem arises. Mainly I will say to complete the dose if he completes it then may be he will get relief from the disease.

I-Is there any guideline on the use of antibiotic?

R-Guideline means how many dose and how many times we are giving we follow that. We follow the dose, duration, and root.

R2-Mainly it is written in the medidcine.

R-It is written in the doctor’s prescription weather it is BD or TDS , we follow that. We also check which medicine we will give with normal saline and what we will give direct.

I-If there is any written guideline with you?

R-Yes, order of doctor.

I-If there is any training or medical education on antibiotic then would you like to take participate in that?

R-Yes I would like to.

R2-Surely I will.

I-According to you what is the benefit of participating in this kind of training?

R-We will come to know about many things because with this kind of survey we are coming to know many things and will know more, so that we can learn more because there is no end of knowing. We are interested to know more.

I-If you have to regular for that then will it be possible for you to do that?

R-It will depend upon location.

R2-We have to do that after managing duty

R-If there is training and our higher authority allows us then we don’t have any problem in that. The location should be good and we don’t have any problem.

I-I asked you earlier and again I am asking your suggestion to reduce antibiotic resistance? You said about making patient aware other than that what steps we can take? You can take.

R2-1^St^ we have to diagnose what is the main problem. Actually every antibiotic is not used in every case so that we have to diagnose 1^st^ to give correct antibiotic so that it works. Another is we have to counsel the patients for taking full dose and for taking nutritious food so that they can work.

I-If you can say me patients encounter means a patient has come, suppose with COPD so in that case your encounter with patients means how you treat, if you please say about that.

R2-Any health talk or something like that means you are speaking about nursing?

I-One patient came with COPD and how will you treat him from 1^st^.

R2-See COPD 1^st^ means will come with respiratory distress so 1^st^ nebulization

R-At 1^st^ position I will give him position, after position is oxygen.

R2-After position what the doctor will prescribe will be given weather it is tablet or injection.

R-Those who comes to us we 1^st^ do positioning then we give oxyzen, after giving some oxyzen nebulization is given, then doctor comes and we give the injection according to the doctor’s order.

I-Do you ever give less than full course?

R-Antibiotic?

I-Antibiotic

R2-No it is not given but there are many patients who want to go home

R-They don’t want to stay here.

R2-We will not take, we will not stay here, leave us. [They say]. Counselling is done that your dose is not completed; you can have the illness again in that case those who go by themselves in that case it happen.

I-So for how many patients it happens?

R-Vey less

R2-It is less

I-How many among 10 patients?

R2-2 among 10 patients.

I-Those who leave without taking full course?

R and R2-Yes

I-In that case do you give medicine for home?

R2-For home some medicines are prescribed like you continue these. Tablets are written.

I-Do you get any feedback of that?

R-They don’t come back.

R2- They don’t come back but those who come always come. So we don’t get feedback in that way.

I-Your level of confidence on advising antibiotic means how much are you confident while giving antibiotic to someone?

R2-Totally confident otherwise medicine can’t be given. [*Laughs*]

I-I want to know how much comfort you will feel if you need to give antibiotic to one?

R-Obviously in case of mild, we will not prescribe any antibiotic for massive, we will say for something mild like cold, fever etc but if there is something severe then we surely will take doctors prescribed.

I-Did you ever give antibiotic without prescription or you have been compelled to give?

R2-No, because this doesn’t comes under our rule so we don’t do that.

I-Suppose there is a need of an antibiotic and that is not available with you so what do you do in that case?

R2-If it is very much needed to the patient then we have to see whether the patient can afford that or not then it is brought from outside. 1^st^ we counsel the patient and if he say that he can buy from outside then he is said to buy from outside otherwise we work with what we have.

R-It works with what we have. There are many types of medicines.

R2-May be it is late but we have nothing to do.

I-I will say some illness, according to you what should be done say. If someone comes with cough, cold and runny nose?

R2-Cough, cold, runny nose, with something on skin?

R-Cough with sputum or dry cough means nothing like that?

I- Cough, cold and runny nose. Came to you.

R2-Means what medicine will I prescribe?

I-Yes

R2-1^st^ we can give Azithromicin otherwise Dox then we can give cetrizin for runny nose

R-Paracitamol for fever

R2-Mainly these.

I-Watery diarrhea with or without vomiting?

R-Aaa for diarrhea 1^st^

R2-For watery diarrhea 1^st^ we have to admit him and put saline

R-Infusion metrozil must be given

R2-That is according to dehydration, with that antibiotic will be given and with that metrozil or ciprofloxacin .

R-Ofloxacin is given many times.

I-Ok, stomach pain

R-Stomach pain?

I-Yes

R2-For stomach pain at stat Decolin injection will be given then medicine.

I-Rashes

R2- For rashes

R-Cetrizin and decadron injection.

I-Thank you
