# Supplementary material for: “Without antibiotics, I cannot treat”: A qualitative study of antibiotic use in Paschim Bardhaman district of West Bengal, India
Source: PLoS One. 2019 Jun 27;14(6):e0219002. doi: 10.1371/journal.pone.0219002 (PMC6597109; doi:10.1371/journal.pone.0219002)
Supplement: S2 File — (ZIP) [file pone.0219002.s002.zip › S2_Transcripts/KAP 318.docx]

KAP-318

Age- 34 years

Gender- Female

Designation- ANM

Highest Education- Secondary

Work setting- Sub centre

I-What is Your age?

R-34 years

I-Under which block is this area?

R-Salanpur

I-In which health centre do you work?

R-Ethora sub centre.

I-How long you are working?

R- 5 years 3 months.

I-In which post you are?

R-2nd ANM

I-So in Ethora sub centre what proportions of patient daily come?

R-Everyday in Sub Centre?

I-Yes.

R-12-15 (patients) everyday.

I-12-15 patientseverday. So who treats them?

R-We have !st ANM and supervisor here, they treat.

I-They treat. Ok, who gives the medicines?

R-We three give medicines.

I: So you three give medicines. So who mainly takes the decision of giving medicine?

R: Decision to give medicine here in sub centre our responsibility is divided for three days. So who is here and whose patient has come we give medicine accordingly. If sister [Senior ANM] is there she give or I give.

I: Ok so what kind of patient mostly comes?

R: Fever, cold, cough, vomiting, loose motion cases are more here. Otherwise we are having DOTS patients they come regularly and Leprosy.

I: Those who are coming to you among them how many patients need antibiotics?

R: I can’t say to how many, like if patient comes with cough cold, as patients come we give accordingly. Someone come for vomiting, loose motion means someone comes for fever then we give Paracitamol, if there is antibiotic then we give or otherwise don’t give.

I: If there is antibiotic means?

R: There is not so much supply of antibiotic.

I: So if I take 10 patient per day so among 10 patients to how many patients you need to give antibiotic?

R: 4 patients.

I: 4 patients need to give. So which antibiotics do you have supply?

R: Here there are Ciprofloxacin, Norfloxacin [*Trying to remember, showing something*], this sulfomethoxzol which is TDS, these three are here.

I: For which kind of illness you need to give antibiotic?

R: Cough and cold.

I: Ok so does people with skin problem comes here means skin illness?

R: Yes

I: In that case what do you do?

R: So in that case we give BB lotion for skin problem if there is supply in the centre.

I: Any antibiotic is given in that case?

R: No, antibiotic is not given.

I: Any tropical antibiotic is given?

R: We give the BB lotion.

I: Ok, BB lotion. Understand. Is there anything for surgery?

R: No, nothing for surgery.

I: Nothing for surgery. You go to field, and as you see patient in the field what kind of medicine do you give? I mean what kind of illness do you see, means mostly seen?

R: Mostly the illness for which they come to centre like fever, vomiting, loose motion. We are mostly working with pregnant mothers, mostly work with children and pregnant mothers. So while visiting them in any fieldif someone says that he is having fever then he is given medicine if there is medicine in bag.

[*Small pause*]

I: So in that case which antibiotic do you give? [*Phone rings*] if there is any facility of test means blood test or other test?

R: Blood test means here haemoglobin and HIV test is done. Means for pregnant mother these are done.

I: Any test before giving antibiotic?

R: No, nothing is done like that.

I: So if anybody needs antibiotic how will you understand that he needs antibiotic?

R: Antibiotic is not used here mostly. If there is heavy cough and cold, not decreasing then we give. Otherwise antibiotic is not used here in mostly.

I: Ok if someone is having direahoea or you are giving antibiotic to someone for direahoe then for how many days do you give?

R: If thre is direahoea then mosly we give Metrozil and ORS. We give Metrozil for three days then we say if not feels better then go and see a doctor because we are not doctor and there is no doctor sitting over ere.

I: Other antibiotic which you are giving, for how many days do you give?

R: For 3 days.

I: You give for three days, if not cured after that?

R: Then we say to visit doctor.

I: Then you say to visit doctor. If comes after three days and say that medicine is not working on me?

R: Yes then we say to consult doctor.

I: Where do you send?

R: We send them To Pithaikary or Asansol SD.

I: Ok .Is there any facility of Sputum tests here?

R: There is no facility of sputum test here, we know the symptoms if a patient comes we send them to PHC for cough test.

I: Ok if any sputum test comes positive then in that case which antibiotics are given?

R: If comes positive we don’t any medicine here. If comes positive then we are sent DOTS from Pithaykari and we give that accordingly, we don’t give any antibiotics separately.

I: What is the name of that? [medicine]

R: Which one?

I: The medicine is sent.

R: Cat 1, Cat 2

I: I see

R: It comes as DOTS medicine.

I: When you counsel patient how much time does it take?

R: How much time, minimum 10-15 minutes.

I: Which kind of patient needs more time? [*Pause]* I mean you do counselling.

R: Yes

I: Which kind of patient do you counsel?

R: To the DOTS patients. When they come for 1^st^ time, want to know then we have to make them understand.

I: If you explain a little bit means what do you say while doing counselling?

R: Means what happened, from when, for how many day you are having this problem these things. [*Pause*] What else to say?

I: I mean when you are explaining to DOTS patients then how do you do it if you can explain the process?

R-When the patient comes 1^st^ he said about his problems than seeing the symptoms I ask what happen, for how many days means we say this kind of things and after that we send to doctor.

I-Ok so in this case does the patient take full course?

R-Yes the take.

I-Is there such patient who don’t eat medicine after taking from you or don’t complete the full course?

R-Yes there are such patients.

I-Aaa if

R-Yes this makes problem, we are having patient who goes out for work for one month or two month, those patient miss the course , otherwise they complete the course. There are many who get cured from here.

I-If I say about general antibiotic in that case how many patient complete the course? Suppose there are 10 patient among them how many patients take medicine sand how many don’t?

R-Everyone take. We don’t give medicine for long days, may be we give for three days and yes they take for three days.

I-In that case do you tell to take the full course?

R-Yes that must be told.

I-In that case you tell. How do you tell?

R-Means we say the time like you have to take within this time, gave for three days, you have to take three days, and you have to complete whatever we gave. If don’t decrease after that then you visit doctor.

I-You keep medicine over here. So when you are giving the available medicine to one then how much it become effective?

R-If little bit happens then our medicines works. People are taking for fever, cold, cough, loose motion and it is working. If ones fever is not decreasing, it is more than 3 days then he must go to doctor. Otherwise one comes for headache then he takes medicine and get relief.

I-No, you said all medicine are not available with you.

R-Yes, all medicines are not available.

I-So if you find you don’t have then medicine which is needed then what do you do?

R-If it is not available then we say to buy outside. If it is not available at that moment we say to buy it outside.

I-The medicine which you have if comes near to expiary means that will expire soon then what do you do with that medicine?

R-after seeing the expiary we cant give more to patients, we have to give that much which is required to the patient. If expired what to do, we throw them away.

I-Where do you throw?

R-Where do we throw, we are having dustbin here, we throw there.

I-Ok, after that where does that go?

R-It [dustbin]is situated at the health centre.

I-After that what happen to the dustbin? Where is it thrown?

R-Dustbin –it gets collected in the dustbin only.

I-Ok, understand. When you are giving antibiotic to one or explaining something about antibiotic then how much do you feel like I am

R-[*interrupting him*] Here there are not much antibiotics if it is then very less, and we don’t have the rule to give antibiotic.

I-No, I mean when you are giving antibiotic then how much confident do you feel to give that?

R-How can I feel too much confident? Whatever we are taught, we learned, we give accordingly, we cant give beyond that.

I-Did you get a training on antibiotic?

R-No, we did not get training on antibiotic separately.

I-Then who taught you?.

R-Doctor I mean the medical officer who comes over here, I saw him to give And learned from there.

I-Ok so in case of taking or giving medicine how much patient are involved mean how much they demand from you?

R-They have demand because most of the population her are poor, they come to take medicine from here.

I-Those who don’t take proper dose what do you do with them? What do you say?

R-If they come and say thet they are not feeling better then it is said to them that you did not take the medicines properly.

I-What do you say them? How do you counsel?

R-We say to take medicine timely.

I-In this case any result after that?

R-Yes, many of them get relief, if not then they go to doctor.

I-No I mean do they listen to you?

R-yes they listen.

I-Those who cant afford treatment means those who don’t have the ability to afford the treatment they? What do you do to them?

R-In such case we do whatever we can here then we sent them to Pithaykari or SD hospital.

I-If someone buy antibiotic from chemist shop and take then how do you manage them? After that comes to you them how do you manage them?

R-What to say him? Nothing to say him.

I-In that case what is your treatment?

R-In trhat case we say to vuisit a doctor.What you have taken or not, we don’t have that much knowledge because we are not doctors. Our main task here is immunization and with pregnant and antenatal mother.

I-When patients demands antibiotic from you then how do you counsel them?

R-how to counsel them, we say that we are not doctor whatever we have at sub centre we are giving if you feel better after taking this then good otherwise you consult a doctor.

I-Ok suppose one patient came, understand, and you feel like this patient does not need antibiotic then what do you do?

R-If there is no need we don’t give. If I feel there is no need to give antibiotic I don’t give.

I-I am giving you an example. Suppose a patient came with the complain of loose motion for few days, fever, so what is your opinion regarding this? I mean 1stly.

R: If one says about loose motion, vomiting to us we give Metrozil , ORS etc.

I: If then also don’t feel better?

R: If don’t feel better then we say to consult doctor.

I: To other doctor?

R: Yes

I: If you say in details about the use of antibiotic. What is your opinion regarding the use of antibiotic?

R: No I feel means for any type of illness antibiotic is needed. Thats the thing, what else I can say?

I: In which case it is needed more and in which case less if you say?

R: In which case?

I: Which disease?

R: What disease will i say? Cough and cold don’t be cured without antibiotic.

I: What else?

R: Dycentry, if there is dycentry for few days then antibiotic is needed.

I: As you said about antibiotic means you said about the diseases where antibiotic is used. So when you are giving antibiotic to someone how do you give it?

R: It will work fast. It will be cured fast.

I: No I mean how do you give? How you explain that the antibiotic should be taken?

R: If something happens antibiotic works, i give by thinking this. As we are said, we have seen to give we give accordingly. How to give by my own opinion? Whatever we have seen and learned we do like that.

I: Antibiotic resistance or if I say medicine don’t work, did you see here such case?

R: Yes, many patients come and say sister the illness did not decrease, it happens many times.

I: In you workplace means here does it [antibiotic resistance] create any problem?

R: Yes. Suppose he has taken medicine from here ant did not get better.

I: In such case what do you do?

R: In such case what to say, we say to visit a doctor. If comes here and did not get better then we don’t say to go back home and no medicine is needed. In such case we say to consult doctor or if he is poor person then we send to Pithaikari, doctor sits there, so to consult him.

I: Do you givem medicine for few more days to him?

R: No

I: Or some other medicine?

R: No, not any other medicines.

I: So as drug resistance is happening and you are facing problem in your work place so what step should be taken to stop this? According to you.

R: What to say [*laughs*] ?

I: Whatever you think.

R: The govt should take some steps.

I: What kind of [steps]you think?

R: Supply some medicine which will work on them, which will help to cure the illness.

I: What steps should be taken to stop this? According to you what steps should be taken to stop this resistance which is happening?

R: I cant say what steps they should take.

I: Ok, do you know how antibiotic resistance happen?

R: No, i don’t know that.

I: Resistance like if we don’t take medicines routinely then resistance takes place.

R: I understood, it happens in case of DOTS.

I: Yes in case of DOTS you said In case of DOTS it happens. So in this case resistance is happening. As resistance is happening that means the medicine is not working anymore. So to stop this in the community, you yourself tik that antibiotics are overused in the community. To stop this what steps should be taken? As a ANM what do you think?

R: What to say, i can’t say anything.

I: Do you think antibiotic resistance is a problem?

R: Yes it is a problem. [*Firmly*]

I: Thats whay i want to know what steps can be good or to decrease this?

R: The patients should be made aware about the use of antibiotics, they are not taking or doing such things, people should be made aware why the antibiotic is needed, or how much it is important.

I: What else?

R: What else, whatever we can do we do.

I: What you people do to combat this?

R: We aware people, we can’t do anything other than that. We aware people, whatever is needed we give; if not get better then we say to consult doctor.

I: Is there any guideline on antibiotic?

R-No, there is no guideline.

I-Is there any guideline from Government side?

R-No there is no guideline from Government side.

I-Do you know personally any kind of such guideline?

R-No

I-If there is any training or seminar organised in future on antibiotic or on antibiotic prescription would you like to participate on that?

R-that is the problem, where will they say to go, to go there is problematic.

I-No, I men if it is done?

R-Yes I would like to, why not, I would.

I-Ok, will you be able to participate regularly?

R-No, I will not be able to attend regularly.

I-So if you cant, you said you will not be able so do you think it is needed to attend such kind of programme regularly?

R-If you want to know you have to go, if you want to know something. But for work it will not be possible for us to attend regularly.

I-So do you think such kind of programme will be useful for ANM ,GNM or in your work?

R-Yes why not, we will come to know about antibiotic.

I-If you say little more why will it be useful? Or you want that if such kind of programme is done you will participate?

R-we should have the need to know, knowledge will increase about that, it will be helpful for our work, we are giving antibiotics to people so we will be able to make them aware better if we know.

I-as you are keeping antibiotic with you or you are having stock of antibiotic so why particularly these antibiotics are kept, why not others antibiotics are kept?

R-Whatever government is supplying, our block is supplying to us. We cant say by ourselves like give me this or that. [Medicine]

I-So as these antibiotics are kept in stock in that case by seeing what the government is supplying such medicines like this kind of patients are coming so these antibiotics

R-[*Interupting the interviewer*] Because sub centres are situated in the villages and in villages people are mainly suffering from fever, cough, vomiting, loose motion.

I-Ok, that’s why these antibiotics are giuven. Ok tell me in detail, a patient came to you, ok, seeking antibiotic like give me antibiotic, if you share such kind of experience with me ?

R-No, I don’t have such kind of experience.

I-Or in case of giving antibiotic, or not completing full course such kind of experience if you could share with us?

R-Not such kind of things. I said patient comes to us for fever, cough, we give whatever antibiotic we have and say take this for three days means we give for three days , some patients complete the course, they take for three days and there are people who don’t don’t take. If someone comes and say I took but did not get cure then we say to visit a doctor.

I-In such case I wanted to know the number, you said many don’t do [complete course] so suppose to 10 patient you

R-Nunulal Baski was a DOTS patient, he stopped medicine after taking for few days,. Another one was Uttam Konra MDR patient, means he has 2 year’s course, he started from October, in next October it would be completed, he stopped in august. For 2 months he did not complete the course. Such kind of MDR patient means injection, medicine, he took injection for 6 months, in holidays he called me from home saying sister I have injection. In October it would be completed, it was 2 years course, he left the course in august, means he stopped in august. I mean this kind of things mainly happens in case of DOTS patient.

I-In case of DOTS. In case of DOTS patients how many means how many among 10?

R-Among 10 may be 1 or 2 patient.

I-So what do you do in such case?

R-What to do, we make them understand by going their home, there are ASHA sisters who goes to the field, so who is having patient in their respective field they make them understand. If someone don’t come then what can be done? Many people are going outside for work then the course is sopped.

I-So when they go [for work] then you give them the full course?

R-No that is not the rule, the rule is to take medicine from our centre. If a patient is seek then there is DOTS provider they go and give the medicine at their home.

I-Those who stopped means discontinuing for going outside, is there any process for them?

R- What will do to them means for how many days I will give medicine, we cant give excessive medicine. In case they left for 15 days then suddenly one day comes back and ask for medicine like sister give medicine and takes. But that medicines are not working anymore, such things are happening. And there are patient like this one [*showing*] took 1 month and did not come again.

I-I will say the name of few disease, what you do in that case oif you say to us.Cough, cold and runny nose. Cold, cough and runny nose.

R-Yes

I-such patient came to you what you do?

R-Whatever medicine is available at sub centre, whatever we know we give accordingly.

I-What do you give?

R-If there is runny nose then he is given Cetrizine, other than that there is AmoxycilinMox which is also given.

I-If comes with fever?

R-Paracitamol.

I-If there is waterey dirahoea ?

R-ORS

I-If comes with stomach pain?

R-There is supply of Beledona, it is given.

I-If comes with rash, if there is rash in body?

R-Then if there is BB lotion then it is given.

I-You give only ORS in case of watery Direahoea?

R-We give ORS, if there is loose motion then Metrozil, Norflox is given.

I-Norflox is given.Ok last question, when you are advising someone either at field or at clinic how much confident do you feel that I am giving this antibiotic?

R-Which I know that if I give this medicine then it will be cured then I feel that it will decrease. This antibiotic should be given for this illness; it can be given, thinking that we give him.

I-In such case do you consult with anyone?

R-Yes, there are sisters [ANM, supervisor]I ask them like if this can be given if they say then I give, when they it can’t be given then I don’t give.

I-Aaa there is no doctor?

R-No there is no doctor.

I-If you have to do prescription any day?

R-No, there is no need of prescription.

I-Means you only give the medicine?

R-Yes, we have to write at our register like this patient came with such things, he can be given this [medicine]. We give Metrozil, ORs and write his name at our register.

I-At the very end once again I am saying which you understand, you said your opinion about antibiotic and your opinion to combat antibiotic resistance, these two things.

R- What to say? I [*smiles*] don’t understand anything.

I-Whatever you feel as an ANM.

R- Yes some arrangements should be done from government’s side regarding antibiotic.

I-What kind of arrangements?

R-What kind of will I say?

I-In what you think that it will be good, beneficial for everyone.

R-If there is supply in the sub centre then a

I-Supply of what?

R-Means Antibiotic or if a doctor sits, if a doctor is given at the dub centre ten the sister will also come to know that which antibiotic should be given for which illness, how much effective it will be?

I-I see, and to combat resistance?

R-People should be made aware.

I-What will you say to people?

R-That what will happen if you take this, if you leave the illness will increase, may be decrease if you take this.

I-do you think any other people should be made aware?

R-Public should be made aware.

I-How you feel regarding this whole thing?

R-Yes it is very good.

I-Do you want to say anything to us?

R-What will I say, you are doing this work, see if anything can be done.

I-Ok, Thank you.
