# Supplementary material for: “Without antibiotics, I cannot treat”: A qualitative study of antibiotic use in Paschim Bardhaman district of West Bengal, India
Source: PLoS One. 2019 Jun 27;14(6):e0219002. doi: 10.1371/journal.pone.0219002 (PMC6597109; doi:10.1371/journal.pone.0219002)
Supplement: S2 File — (ZIP) [file pone.0219002.s002.zip › S2_Transcripts/KAP 362.docx]

**KAP 362**

Gender: Female
Age: 53
Occupation: nurse
Highest degree: Graduate

I-Tell something about your OPD, how much patients come here everyday?

R-Yes our OPD is community based OPD because here every person after work , someone may be in the middle of the work, someone comes at the end, most of the population are daily labor so we need to wait for them a little, and we have to open early also. So everyday 30-40 means 40-50 patients come to the OPD.

I-Does the doctor come everyday or in alternative day?

R-No, in alternative day.

I-In alternative day. How many staffs are there now?

R- Here we are two nursing staff, one pharmacist one group D [clerical post] and a doctor.

I-How do you manage patients in the absence of doctor?

R-In the absence of doctor we do the symptomatic treatment then when doctor comes we refer to him. If it is a serious patient then we refer to doctor.

I-what are the diseases you see regularly here?

R- Skin disease, most [not audible] because they don’t use purified water, so water related disease like skin disease happen much. Skin disease and diarrhea, water related disease.

I-What other common [disease] seen?

R- Common I am saying means this is TB endemic area, means most of are TB.

I-For that there is DOTS.

R-There is DOTS running, they don’t maintain hygiene, dint get food properly, community based people are like that, they don’t get food, as they don’t get food they are mostly affected by TB. But here the treatment is good, medicines are supplied.

I-Here how the medicine supply is looks like? Means how it is done?

R-All that are from BPHC.

I-From BPHC?

R-Yes, supplied from Panagarh BPHC.

I-Per month?

R-Per month, per weekly they bring, you are speaking about our medicine? Medicine of PHC?

I-Yes, of PHC.

R-Medicine of PHC comes from Bardhaman, bardhaman DRS.

I-Ok, can you please name some antibiotic among the medicine you are having here?

R-[*Loud sound of car horn*] there is CEfron, CEtron D, Metronidazole, nothing else, in injection form there is nothing else.

I-What are there in tablet or syrup form?

R-I said the tablet form, [*repeats the sentence*] and patient means for kids there is metrozil syrup and [*trying to remember*] nothing else, for kids there is metrozil syrup. There are lots of syrup PCM, paracitamol, cough syrup but antibiotic there is metronidazole.

I-I see. How much time it takes to speak with a patient?

R-2-3 minutes

I-2-3 minutes. You said there are lots of skin problem seen here. How do you treat them?

R-Treatment means skin problems means most of them come with itching, I said in Bengali they come with itching, for itching we give BB lotion or probably cerzin tablet, we give cerzin, they almost get cured by this and we say them to take bath in good water, as they take bath at pond there cows and buffalo also take bath so may be the germ come so we say them to take bath in tap water.

I-Any tropical antibiotic here?

R-Tropical antibiotic aa if it becomes very serious then we give cetron DS in tablet form, we don’t give Cetron Ds to everybody, may be we give Cefran, as cetron ds is means having reaction so we don’t use cetron ds without doctor’s permission.

I-Is there any facility of microbiological testing?

R-No, earlier it was.

I-There was?

R-I heard there was but now there is no lab.

I-So if you think that someone needs attest then?

R-Then we send to Panagarh BPHC.

I-Even blood and urine are also not done here?

R-No, we need to refer everything to Panagarh BPHC.

I-That’s tough. Is there any facility of minor surgical programme?

R-Minor surgical, no, nothing. There is no OT.

I-Is stiching done here?

R-Yes, stiching is done here, if someone comes with cut, minor cut we stich that.

I-How frequently do you get respiratory tract infection or diarrheal cases? Is it frequent?

R-lots of cases.

I-You get lots of?

R-RTI or diarrohea is most common here.

I-How do you handle respiratory disease?

R-We don’t give antibiotic 1^st^, if it is in primary stage then we do symptomatic treatment as I said then if it becomes severe then we give antibiotic.

I-What antibiotics are used in this case?

R-Aaa Cetron DS, Cefron and Amoxicillin.

I-Amoxicilin?

R-Yes Ampiclox is also used, Penicilin is used.

I-Aaa how do you handle diarrheal disease?

R-We give Metronidazole then Norflox with that a packet of ORS.

I-So these thing means when you are giving antibiotic whoever is in the OPD and giving antibiotic to one then do you go for a test before giving antibiotic?

R-Na, about test what we know that in Cetron DS there will be a shock which is reaction, I will not say shock, reaction, and rest suit to everyone, safe, safe antibiotic.

I-How frequently do you use antibiotic here means do you give frequently, how do you give?

R-It is given to some people, not to everyone.

I-Not given to everybody. When you are deciding that you will give antibiotic to someone then how much confident do you feel?

R-If that kind of disease then confident if it is such kind of disease then we have to consult otherwise

I-[Interupting] with whom do you consult?

R-I consult with my colleague. I do with colleague otherwise I stop , we wait till the doctor come.

I-In case of antibiotic for how many days normally it is given?

R-3 days.

I-3 Days?

R-3 days.

I-So do you ask them to come after three days or the course is of three days?

R-If it is cured then we say not to come, a antibiotic can be given for three days otherwise we make it lengthy, may be for 5 days or 7 days.

I-Suppose you fe;lt that someone should be given for 5 days or 7 days, in that case also you will give for three days?

R-Yes we give for three days, then doctor decide for how many days he will give according to the gravity; he gives according to the gravity.

I-So in such case where you felt he need for 5 days but you gave for 3 days do they come back?

R-Some patients come.

I-Some patients come.

R-Yes

I-What is the rate of that if I say among 10 people.

R-Very less, that is very less.

I-Follow up is less?

R-Yes

I-What do you think why the foillow up is less?

R-Mostly get cured. They get cured, may be 2-4 people come then we say there is doctor, go and visit the doctor, doctor knows better what else to be given. Otherwise we would not be able to treat, we would not be here.

I-I see. Does it happen like patients come to you and directly ask for medicine like give me this medicine or that medicine by name?

R-Yes they say but we don’t listen to them much, they don’t know much about antibiotic, because as they are habituated like there is little illness and pharmaceutical said name of some medicine, they buy and take that, but that is not done in proper way, there is a course of antibiotic which they don’t complete, after taking 2-4 they think it is done. Thinking that we choose what to give and what not, if they say we don’t pay heed to them much.

I-[*laughs*] How do you handle such situation?

R-The situation we need to tell them in a good way because they are community people, we need to behave well, whatever medicine we give or not, the 1^st^ thing with them is behaving with them. If the behaviour is not good then there will be problem, they are community people and we need to make them understand, we need to speak much which is not needed in sub division medical college. Here we need to speak much with them, we need to speak with them like family, and they think that we belong to them. There is a probing in Bengali ‘they belong to us’ just like that.

I-Here you are having a pharmacy store so there are always some medicine, some medicine are near to expiry, how do you handle that?

R-We exclude that. We give by seeing expiry. The ones which are expired it may happen , it is a small hospital, maybe we brought once but it is not disbursed that we do in proper way like dispose by digging soil and put them there.

I-Does this happen here or you send to BPHC?

R-No, done here and some medicines also send to BPHC like the vital medicines are sent to BPHC.

I-After expiry?

R-Yes, after expiry we need to send.

I-How do you handle those medicine which will expire in next 15-30 days?

R-Those we give little more.

I-You give?

R-We give means may be other antibiotic could work but we try to give that antibiotic, in case of Vitamin tablet may be 10 was needed but we give 20, if they consume that it is not harmful, that is not harmful. This way means antibiotics can’t be given in that way, it can’t be given, these medicines we give.

I-Is there any needed to counsel patients here?

R-Counselling here there is not much scope.

I-No, the way you speak that’s.

R-Yes counselling we need to speak in that way [*repeats the sentence*]

I-There is nothing to do in different way?

R-No, nothing to do in different way.

I-You give medicine to one patient, she did not take proper dose, what will you do in that case? He came to you later again.

R-In that case we need to start medicine again. May be he stopped after taking one day, he accepts, he says, they are community people, they are simple, mostly they say like sister we did not take, then we start again from beginning, yes we start from beginning.

I-That medicine or another?

R-We give that medicine because it will resist means if we give another medicine it will so we give. If they take one day we say take 3 more days or 5 more days, we advise that.

I-You were saying most of the people goes to pharmacy mean chemist shop 1^st^ then takes 1-2 medicine and if they come after that how do you handle ?

R-In that case the total means what we feel we complete 3 days course.

I-Means from beginning?

R- From beginning.

I-You do it yourself?

R-By ourselves means we don’t know that correctly, the strip we see there we can’t recognize the name. So we start from beginning by ourselves.

I-Suppose on patient came to you with a strip suppose as a example I am saying he took 2 Amoxicilin then came to you, so you continue amoxicillin or according to you?

R-We continue Amoxicilin. If it is available with us good otherwise we say you buy this for 3 days or if available with me then I give.

I-many times it is seen they take medicine from shop by themselves. What is your opinion on that? What do you think?

R- This should not be done[repeats the sentence]Without doctor’s prescription or without Medical personnel’s advice it should not be done because if you take medicine this ways it will be resistant and it will not cure, hmm it should not be done.

I-There are many patients that go to one doctor today another doctor tomorrow, what is your opinion on this?

R-See this thing if one is not satisfied with one doctor he will go to another doctor I think we should leave it on them.

I-Many times it happen that one patient came to you and you think that according to present condition antibiotic is not needed now but after seeing 2-3 days you want to give, so does such case happen to you?

R-yes they come, we say if it does not cure with that then you will need antibiotic, come again.

I-Do they come again?

R-Yes they come.

I-We are speaking about the word resistant so what do you think why resistant happen?

*Pause*

R- Actually why resistant occur if we use one medicine again and again or what to say may be not completing may be they don’t complete the course and use then our bacteria or germ that we have inside which they do may be they can’t work on them properly. May be that is called drug resistant, I don’t have much knowledge.

I-Means is there any particular reason or 1-2 reason like if we do that then resistant will occur or if we don’t follow that then resistant will occur?

R-No, course complete aa I can’t say this properly.

I-We use antibiotic that is having risk and benefit, how will you explain this?

R-When you need if you use at that time then it is ok and if it is used frequently then there will be a harm means there is a risk. Excessive use of antibiotic is not good may be some medicine taken without antibiotic, with that his immunity grows but if immunity grows and antibiotics are used frequently then it will harm more.

I-Did you see any patient here who become drug resistant?

R-[*Pause]* No we did not get such.

I-Is there any way to know if it became resistant?

R-No, that is may be exam in lab medicine is tested like in case of typhoid it is tested which medicine is resistant then medicine is chosen. In case of typhoid it happens, I don’t know anything else, this is a small hospital.

I-No, that is not a problem. I will tell you some disease so you will tell what will you give or how will you treat. Someone came to you with fever.

R-Hmm, with fever. Only fever or there is running nose, headache?

I-You say both if both is there.

R-If there is running nose or come with headache or with temperature then we do symptomatic treatment. If we see it is not cured then we give for 3 days or 2 days and then also did not get cure then we use antibiotic. Ah what else?

I-What kind of antibiotic will you use in this case?

R-Amoxicilin.

I-Amoxicilin?

R-Hmm. As there might be a direahhoeal problem in Amoxicilin so we give a protection may be we give a metrozil.They take that. We learned this from doctor, means doctor use this, it is a small hospital, if there is no doctor then we need to do these some small work.

I-Came with cough cold and runny nose. What will give then?

R-as I said symptomatic treatment.

I-In this case also?

R-Yes, should I say the name of tablet?

I-Yes say.

R-We give cetrizin, may be at night after dinner, at night they take rest for 12 hrs or 8 hrs, with cetrizin there is little bit dizziness so we say to use at night and give paracitamol take after food for body ache or headache.

I-I see so in this case you will not give antibiotic in the beginning?

R-No, will not give.

I-In case of watery diarrhea with vomiting?

R-If there is watery diarrhea we give a full course of Metronidazole, take one three times may be sometimes give for 5 days, sometimes by understanding the gravity may be give for three days and with that may be we give Norfloxacin, 6 tablet and with that ORS must, we say to take that by melting with water. If it is very serious then we refer because in that case saline is needed.

I-Ok, if there is vomiting with this?

R-In case of vomiting is same, in vomiting the water get extracted, aa if there is vomiting?

I-Vomiting with diarrohea.

R-Then we need to give tablet of vomiting.

I-What is that?

R-Domeperidone. We give Domeperidone.

I-If there is no vomiting then only

R-Then we give only that, that treatment goes on.

I-Your referral is BPHC?

R-Referal is BPHC

I-One came with stomach pain?

R- Pain? In that case we see for indigestion there is a pain abdomen that is indigestion. For what the pain is happening we don’t understand from the 1^st^ day, so we for indigestion we use Pan D, Pantaparazole and with that if they say lot of pain with that we give two pain killers for stomach pain. If then also did not get cure then we don’t take the risk. In that case we for pain abdomen as they come with pain abdomen we refer, we say to visit doctor.

I-So you refer at the 1^st^ day or?

R: No we don’t refer at the 1^st^ day.

I-You see for 1-2 days.

R: Yes we see for 1-2 days.

I:In this case you give any antibiotic or?

R-No, we don’t give any antibiotic.

I: Suppose there is rashes in the skin [*repeats the sentence in bengali*]

R: In such case we say to use good soap or we say some lotion to use or not to use oil as there is itching and we give 2-3 tablet for itching.

I: What kind of tablet?

R: Cerzin is given [repeats]

I: So any antibiotic in this case?

R: No, we don’t give antibiotic.

I: Do you interact with MR? Do they come here?

R: No, not with us, if they come they visit doctor. If there is no doctor then they show some sample to us but we don’t prescribe so it is of no use.

I: So this does not affect you in case of giving medicine?

R: No.

I: I am again coming to the training question. If there is educational training what is your thought on that?

R: Absolutely these should be taken.

I: It should be taken?

R: yes it is very much needed, because we are handling this, in some part we face problem, if it is clarified then it will be more helpful.

I: What other reason for which you think you should take these training?

R: As we are handling people, if we give any wrong medicine or if there is training it will be helpful to choose correct medicine, when we should give what, if there is a training, we will not give wrong medicine to the people, as we need to handle the PHC till the doctor comes, in our PHC there is not doctor everyday so we can treat that day correctly.

I: So you think these should be organized?

R: Obviously

I:If there is such programme then you

R: Participate?

I: Yes

R: Yes I want to participate.

I:What do you do to update yourself mean you read books or internet something like that?

R: Yes I read books, we have some books and this is an era of internet, I see something in internet.

I: Do you follow any particular journal or something?

R: No, I don’t follow such.

I: Ok we spoke about resistant ones, we will speak again, to combat this resistant what do you think what can be done to combat antibiotic resistance? How can it be combated?

R: Actually to combat antibiotic resistance we should no use antibiotic t the very 1^st^ moment.

I: It is not good?

R: Yes as there is a disease its immunity grows inside that immunity exist for few days, in some case it works without antibiotic so I think we should not use antibiotic at 1^st^. But it has gravity, if it is very serious then antibiotic must be given but at 1^st^ means as we take like puffed rice we should not take antibiotic like that.

I: If I say about every level who should do what according to you from upper level means from state level or district level? To combat this can anyone do anything?

R: For this there is a need of public education means camp should be done; camp with people means this kind of some work should be done.

I: Any other example except camp? What other thing can be done?

*Pause*

R: You can take time, no problem.

I: As the promotion is done means leaf means what is said?

I: Leaflet?

R: yes, we can use leaflet, we can distribute to the people.

I: Means to aware people.

R: Yes to aware people from the root level we can aware people. If we are given leaflets, one by one patient come, we give to the patient with medicine. They read that at home very carefully, I have seen in some cases, as we give, sister has given or it is given from health center so they give importance like we aware the pregnant mothers by giving leaflets, if we do the same may be resistance will decrease, they will not take like puffed rice from here and there, they will come to health center, one judgment will be made by him, if antibiotic is used after correct judgment then it is good.

I: Do you think in government level anything can be done?

R: Yes in government level obviously, govt can do everything [*repeats and laughs*]

I: If I say in PHC there are three people involved in giving antibiotic. 1st is doctor who is prescribing, nurse and pharmacist. So how does their role matter in case of giving antibiotic or to combat antibiotic resistance?

R: Dr is 1^st^, doctor is obviously, to combat resistance doctor is very much then there is pharmacist, he

I: *[Interrupting*] if you say how doctor can?

R: How doctor will may be he will not give antibiotic at 1^st^, without giving antibiotic he will treat then may be say to come then after seeing again may be use antibiotic, this is enough to make resistant because if we give at 1^st^ then the antibiotic will not have means our normal immunity that grows we don’t give that to work. Pharmacist also can do the same then there is pharmacist as he has studied about pharmaceutical so he knows well about medicine, we studied some part, not more was taught, nursing care was given more importance, as we are in nursing profession.

I: Do you know if govt has done something to combat antibiotic resistance? Di the take any step?

R: I don’t know this well, I don’t know.

I: you see RMP, mostly seen at village area those who are called quack, what is their role in case of antibiotic resistance? How much they are responsible?

R: they have role means I don’t know if they are educated or not, as they are non-educated, I will not say non educated person, am not saying, they should have proper knowledge about antibiotic, what is its effect, what is its reaction everything should know then they can use antibiotic, but if they use it without knowledge then it is very harmful. Very harmful.

I: So what do you think means they are also responsible?

R: Yes they can take part in this; they can take responsibility to combat resistance.

I: suppose one patient comes and says you give me antibiotic and you feel that he don’t need antibiotic. So how do you handle such cases?

R: in that case we say hinm that you don’t need antibiotic now, you use this for now, if don’t decrease after that then we are having this. So they do like that, some people say that I need then we understand the thing and may be give some tablet to him and say in this there is some amount of we have to say in this way. We give some paracitamol then say there is some amount in it, with this you will be cured. So they understand that and leave.

I: this is very good

R: [*laughs*] Nothing else to do, we have to tackle in this way.

I: That is right; you have to make them understand in this way. [*She asked if it is one pm then we should stop the interview*]. In many case as I said they demand antibiotic from you so how do they ask, do they understand that this is antibiotic or do they ask by name?

R: Yes, they are community people so may be they heard from someone, they heard stories like I took this, I took that, with that they say the name, we feel shocked how nicely they say the name, so they say in that way, we get everything.

I: No, in many places it happens like give me the blue tablet or white tablet.

R: Yes they say that, say that, that blue tablet if paracitamol comes in another form suppose I say if paracitamol comes in different strip or in another colour then they say like I need the blue one, there comes a blind belief among them.

*Pause*

I: There are many antibiotic means broad spectrum antibiotic, means one antibiotic can be used in many purpose, so what is the availability of that?

R: Its good, it is available here.

I: It is available?

R: Hmm

I: Is it easily available in the market?

R: I can’t say about market but we have in the hospital supply.

I: Is there combination drug or antibiotic?

R: Yes there is.

I: You prefer that much or single one?

R: It depends upon the disease, what will work on which, is used accordingly.

I: In case of using what do you think like combination will affect more or single will affect more this should be given or that should be given, anything like that?

R: This is chosen by doctor, broad spectrum as you are saying is chosen by doctor, we are not in that.

I: I see. I would like to know your personal view-you prefer generic more or brand name?

R: Brand name no I like generic more.

I: why the generic?

R: What is the meaning of generic?

I: Generic means which is made by the main thing, brand name is according to company.

R: Yes I like generic because see brand name how company will produce may be in that the things are not used properly; it is only for publicity, so we use generic.

I: Thank you. It was very good.
