# Supplementary material for: “Without antibiotics, I cannot treat”: A qualitative study of antibiotic use in Paschim Bardhaman district of West Bengal, India
Source: PLoS One. 2019 Jun 27;14(6):e0219002. doi: 10.1371/journal.pone.0219002 (PMC6597109; doi:10.1371/journal.pone.0219002)
Supplement: S2 File — (ZIP) [file pone.0219002.s002.zip › S2_Transcripts/V5.docx]

V 5

Age: 36 years

Gender: Female

I: 1^st^ you say something about yourself, how old are you, where do you live?

R: I am 36 years old and I live at college para and regarding me I want to tell that I always come to Govt hospital because the medicine of here suits me, I rarely go to another hospital and if I take medicine from here I get cured. When I see the case has worsened, am not curing then I go to outside and see another doctor.

I: who are there at your home?

R: At my home I am having my brother, his wife with whom you speak was the wife of my brother, my niece, my husband, my son.

I: So you are having a big family.

R: *[laughs*] yes a big family.

I: What are the diseases you face mostly among yourselves?

R: Diseases means we are little bit worried about the kid, she is a seizure baby so we mostly need to come here for her.

I: What mostly happen to her?

R: She is mostly having cold, cough, if there is cold then fever also.

I: Except that what else to the elders?

R: The elders don’t suffer much means we always want to be cautious. And ist not that it don’t happen to the elders, it happens and when it happen we come.

I: What kind of illness?

R: What kind of means every kind of, people suffer with fever, loose motion, what is not there, everything. It is not mandatory that I will mostly suffer with loose motion and not with fever or there will be fever but not stomach problem, everything happens.

I: should I speak in hindi, will it be helpful?

R: No [repeats] you can speak in Bengali , hindi whatever you wish. No problem with that. If you want to ask anything then ask, what I should say.

I: If you suffer with any illness then where do you go mostly?

R: I come here [Refering to Raniganj BPHC] mostly, I mostly come to Govt hospital, and if it is not cured in govt hospital then we go to some other places.

I: I see. 1^st^ you come here?

R: Yes we come here 1^st^. I want if I get cured here without spending money then why will I go to outside. That’s why I mostly come to govt hospital and the doctors here are also good, there was Chakraborty and now there is Ghoshal, they are very good doctor.

I: you come to hospital 1^st^ for everyone?

R: Yes for everyone 1^st^ here, I come here for my husband the kids those we have, we come for bigger ones also or if we come and say then also we get medicine.

I: You mean if the patient can’t come then?

R: if the patient can’t come then we can have medicine by saying the doctor means we often come to hospital.

I: everybody knows you.

R: they know [laughs] . If we say, they give so we don’t face that much trouble.

I: What do you think the illness you said about, why that happens to us?

R: That I can’t tell, how can I tell the reasons?

I: No what do you think that why fever, cold, cough, stomach problems are occurring many times?

R: It may happen for our fault.

I: Like?

R: May be we eat ‘ulto palta khabar’[meaning unhygienic food] or may be for indigestion or for fever we don’t pay attention to our health or we don’t pay attention to our food for that also there may be illness, there is pressure and everything, if we have any problem with food then we feel dozy, we feel sick they we are compelled to come, for all these it happen, what else.

I: When you come to doctor then what do you think, what is your expectation?

R: with what expectation means we expect that we are coming may be we will be cured, if it get cured then well, we discuss at home also [*phone rings*] that we are going to govt hospital if it cures then very good and if don’t cure then may be we will go to some other hospital. We come for that. We come thinking that as early as we are cured whether its kid or elder its good. Sometimes it is cured, sometimes not, sometimes we need to go outside, which is whose means

I: Suits

R: Suits

I: as you are saying outside when it is not cured here so outside means where, any private doctor?

R: We visit private doctor, there is in school para.

I: I see. Are they MBBS doctor or?

R: No, MBBS doctor, they take fess Rs 150-200, we are poor people, and we can’t afford that so we mostly come to govt hospital.

I: Here no fee is charged for anything?

R: No, no fee is charged and we get good medicine.

I: They give medicine at free of cost?

R: Yes and which medicine is not available here, they write from outside.

I: They write and you have to buy that?

R: We have to buy that.

I: Most of the medicines you get?

R: Yes, most of the medicine we get and which is not available doctor says that this is not available, you have to buy this. We buy that according to our affordability and if we can’t then leave it.

I: you don’t feel scared to leave that?

R: What to do if there is no money, we will not get that at free of cost.

I: That’s true

R: Then

I: when you come here with some illness then what do they ask you?

R: They ask what happen, they don’t see by touching, as we are sitting now we do like that means doctor asks, write prescription and we get cure with that, we come with that expectation whether they touch or not but we get cured.

I: What do they ask [*repeats]*?

R: What they ask means they ask what happened and we say what happened to us, cold, cough, fever means what is there we say, and doctor writes that and if there is very much need, and if there is kid and doctor feels that he needs to check then he check by touching otherwise doctor rarely touch.

I: he sees with much time or less time?

R: No, doctor don’t get time to check for a long time, there are lots of patient, you saw now how many patients are there, if he check by touching every patient then it will take the whole day. So doctor quickly checks the patients.

I: if there is any need of test then is it available at hospital?

R: Yes test means if there are blood test or urine test these are available.

I: these are done at hospital. Free or you have to pay?

R: Free and with money also, there are two here, one is beside which is with money and another is free. Who is having what kind of test and money is charged accordingly. Here there is no problem, everything is available.

I: There are small doctors, who are not passed doctor, and called quack doctor, do you go to them?

R: Means?

I: There are many doctors you will see in your neighbors.

R: Yes they sit opening a dispensary

I: Yes

R: no, we go to them rarely, we don’t go to them. [*laughs*]

I: you don’t go to them. Why?

R: Because I don’t believe them. [*Laughs*] Belief is the most important thing and I don’t allow anyone to go, we are having many at our college para means they are siting by opening small dispensaries, they give medicines, you will say that they take money, it is not for money but I don’t believe whether I will be cured there or not so it’s better to go to govt hospital, it will be little late that’s ok, we get good medicine, they give good medicine, I have faith on it , that’s all nothing else.

I: if there is something small

R: if there is something small then we buy medicine from dispensary, there is little fever, or headache

I: You bring from medicine shop.

R: We bring from medicine shop, if there is small we can’t wait at line, we can’t come by leaving our work at home, so we bring from dispensary and get cured.

I: For how many days they give you medicine?

R: Who?

I: When you go to chemist shop.

R: in chemist shop as you want, if you say 2 they will give 2, if say 4 they will give 4, if I say give me one

I: It depends upon you how much you want.

R: yes. They don’t force that you have to give for 4 days or 10 days, whatever doctor writes they say that you are having 10 days course you have to take the whole.

I: When they [doctors] say and when you go by yourself?

R: No, if I go by ourselves then don’t say, if I say give me for 5 days I don’t have money, I will take later then they give for 5 days or 2 days.

I: What do they ask when you directly go to them for medicine?

R: They ask, what will they ask, we go and say give me 2 tablet for headache, they give if we say, don’t ask anything else and we don’t need to say anything.

I: You go to homeopathy more or allopathic?

R: No, I don’t go to homeopathy more, I go to allopathic.

I: Why so?

R: Homeopathy suited me when I was child but I give allopathic to my child and my niece. Homeopathy takes time, it works but takes time so I visit homeopathy very less.

I: What do you think when you come to hospital they give more medicines or its ok?

R: if they give more than it is not needed to take all that, suppose they give for three days, I take one day or two day and get cured then I don’t need to take for three days. Doctor gives medicines for three days. If I don’t get cured after taking the whole medicine, after three days medicine then if I come to doctor again he gives another medicine or he prescribe good medicine from outside like if this medicine is not working them am writing , you buy from outside. Then we buy from outside.

I: Suppose doctor has given for three days and you feel better after two days then you don’t take the rest of the medicine?

R: No, then it is not needed, why to take the medicine then.

I: what do you do with that medicine which is left?

R: The leftover medicine remain, if we see within less period one become seek then we give him otherwise we throw, it will not work.

I: suppose you took medicine for kid, and there are some medicine left, he is suffering from fever and there are some medicine left then after 5 days you are also suffering from fever then do you take his medicine?

R: No, the medicine for kids, now doctor is giving same medicine for elders and kids, so may be we took for kids and there are some left, so may be doctor said to give half to the kid and I take the whole.

I: OK, so you use the one given to kid.

R: yes it can be used. If there is someone’s kid get sick around or we then we say that I am having medicine for fever or loose medicine

I: that is used

R: That is used, I know my kid get well with this, and your kid will also get well. They get cured.

I: It happens many times that I got seek and I took medicine or I have the prescription. So next time do you take medicine by showing that prescription in the dispensary? Do you do like this?

R: No, we need to bring that prescription, doctor writes after seeing that paper. Doctor asks what happen then we say that I am having this this then he writes in that paper.

I: if you don’t come to the hospital and directly buy from chemist shop?

R: no, they don’t give that medicine, it is written not for sale so it’s not available in the shop, and they give another one like the medicine written here is not available but we have another from the same company and give.

I: I see. Did you hear the name of antibiotic?

R: yes

I: Why it is useful? Why we take this?

R: That I can’t say.

I: whatever you think. You take it so you have heard why antibiotic is useful or why we take antibiotic or is it different from other medicine?

R: I have heard antibiotic is good medicine, it is said if we are seek antibiotic gives lot of things to us, if there is wound then antibiotic is given, in fever also it is given so we take.

I: You take only when doctor gives or sometimes you buy from chemist shop?

R: In medicine shop we don’t need to say the name, we have to say what happen and medicine is given.

I: So you take antibiotic from them also?

R: They give by themselves like if you take this it will be good.

I: When they give antibiotic do they say like I have given this this medicine?

R: Yes they say, doctor say like this is antibiotic or something, you have to use in this way, you have to take in this way, they say.

I: So you take as long as it is said or you stop after you get cured?

R: When I think this is good or I am taking this for 5 days and I need to take for 10 days I will be cured totally then I complete the course.

I: you complete the course even if you get cured?

R: Yes and if I think that its ok if I don’t take then I don’t take that.

I: So what is good or what is not good or not needed, you take this decision by asking doctor or?

R: No, doctor says like if I take complete course of it, then will be good, we are not doctor by ourselves, how will we know what we should take what not or the gents of our family brings sometimes like take this medicine it will be good.

I: Is there any course of antibiotic like 2 days or 5 days or 7 days?

R: Yes, there is a course of antibiotic.

I: there is?

R: yes it can be of 2 days or 5 days or 15 days. It may happen that many people don’t have money, we also don’t have money always, doctor gives medicines for 15 days, we take for 5 days, and we can’t take 10 more days.

I: Say more

R: It happens like that, it all depends upon us, and if there is no money we stop the medicine in the middle.

I: with that is there anything

R: With that we don’t feel anything but after few days again the illness occurs, then we thing it would be better if we would complete the course, we left so it happens again.

I: I see

R: [*Laughs*] We feel so but can’t say to anybody

I: have you ever heard that medicine does wrong to us, there is side effects of medicine or bad effect?

R: It may be

I: Did you hear such thing or have you seen someone at home?

R: I have heard there can be some infection, or some other thing happen or may be doctor has written one and I took another from dispensary after taking that something can happen. That’s why if doctor prescribe outside medicine, we buy and show the doctor then go home. It happened many times like doctor has given medicine and written also so we show him and he said I did not write this and I showed the paper that you wrote and he said it is not this you take the one I wrote.

I: so when doctor prescribe which you have to buy from outside then do the shopkeeper make you understand how to take or not?

R: Yes, they make us understand and if we come here the doctor also make us understand like you have to take this medicine in this way. If we don’t understand there we bring here.

I: It happens many times that if there is need you buy from dispensary so whatever medicine you ask they give even without prescription?

R: Means if there is something serious they don’t give but if there is headache or pain in waist, after operation I am always having pain in waist, we say to them and take, we need to take in every 2-4 days. So how many times I will come to hospital?

I: they give the small ones?

R: they give for small things but if there is something serious they don’t give. There is loose motion or fever or headache they give 1-2, if it is cured with that 1-2 then ok otherwise we come to hospital. When we see its getting worsen, it’s not getting well, it’s increasing from one day to two day or three day, it not getting cured then we think that it will be good if we go to hospital, after taking medicine it’s getting worse.

I: Does the shopkeeper give you antibiotic without prescription?

R: No, they don’t give.

I: they don’t give antibiotic.

R: No, if we take written from doctor like I came to govt hospital, doctor prescribe good antibiotic, my niece is having infection at head, if I say doctor write good medicine, ointment cream from outside if it is not available here then doctor writes or if any antibiotic is to be taken then write.

I: do you ever say like write this antibiotic or that antibiotic?

R: No, we don’t say that, doctor will say I am doctor or you, so we don’t say such things to the doctor.

I: you only say about the illness?

R: Many times they themselves write by understanding.

I: Did you ever hear that medicine is not working on body?

R: yes it happens, many times it don’t work on us, may be I took but fever is not decreasing, headache is not decreasing. May be I can’t eat and for that my body is becoming more weak then I say doctor and he say if it is not working I am writing from outside, may it also don’t work then we need to visit private doctor.

I: Yes say

R: then we consult private doctor.

I: what do you think, why medicine don’t work? Medicine is for patients.

R: It may happen for disease, I am sick but doctor did not check me by touching and gave me medicine for something else, it can be for that. So we think that if we consult doctor may be they check us well, may be blood test or urine test is done may for other thing we go to him we can be cured, so we go to private doctor, it may happen that medicine is not given according to the disease, it happens many times that I have something and am taking medicine of something, for that it can happen.

I: When do you go to private doctor or outside doctor in what condition?

R: In what condition means before 2 months I became really serious, I got malaria, I became bed ridden, I visit govt hospital abut did not get cure, then in our neighborhood there is a dispensary, I visit there but there also I did not get cure at last I had to visit another place.

I: So you don’t go them at the very 1^st^?

R: we don’t go 1^st^ because we don’t have money, we are poor people, we think if it is cured spending less money, am going to govt hospital if I need Rs 100 as fee then I can save that, if it is serious then doctor will prescribe outside medicine, then I say doctor if you don’t have medicine then prescribe something from outside so that I get cured.

Pause

I: you come to hospital many times, there are many kind of illness, you have to take different types of medicine so do you know name of any medicine or antibiotic, if you can remember 1-2 name, can say? What medicine do you use more or 1-2 tablet or name of syrup if you can remember?

R: No, I can’t remember such name.

I: Dis you ever hear about side effect of medicine?

R: side effect means?

I: means something wrong is happening after taking the medicine.

R: I did not hear such things, it is said when we seriously sick, many times medicine does good many times bad. I have heard this when you take improper dose according to the gravity of the illness in that case also we fell sick means now I am speaking and at the same time I am feeling dozy or something else may be I go to PHC and say give me this medicine may be after taking that it increases, it may not work, may be I am feeling dozy but said I am having headache then that can also be bad for health.

I: We use antibiotic but is it used in case of others like animals, birds, do you know such?

R: everybody use antibiotic.

I: how it is used in case of others can you say that?

R: In case of others means birds, animals everybody is having antibiotic because we used to rear up goats so when they get sick we used to go to doctor, that is also govt hospital and the doctor say this antibiotic is needed, your goat is very sick, so we know that everyone needs antibiotic.

I: We are eating chicken and mutton, so do you think is there any chance of that antibiotic given to them to come to our body?

R: No, I did not hear such thing till now.

I: The people around you or you people have knowledge about health, about antibiotics, what do you think?

R: there are many people who are educated know about it, what the uneducated people like us will know *[laughs*]

I: no, no you spoke about very good things till now. What can be done to make more people aware?

R: What can be done means as you came from health center, every people should make aware that medicine from outside should not be taken or it is good to take the medicine prescribed by doctor. To discuss about these is good, it’s not bad.

I: According to you how can we do this means speaking with individual person like this or how to speak with a large number of people?

R: If you want to speak with a large number of people there are lot in govt hospital means those who are standing at the queue you can ask by gathering them regarding this, what to do what not. I think its better than going to para [hamlet] as you came from health centre you will get a lot of patient in govt hospital, here you will understand many things.

I: What do you think means you often comes to the health center am not only speaking about Raniganj, other places also, what other basic facilities needed for which people will come to govt hospital? What needs to be improved?

R: What is to be improved, we don’t understand that many times [laughs] we can neither say very good or very bad. Suppose we come here for delivery, sometimes it will be normal but they say seizure is needed, and then they send us so we can’t say that health center is very good or very bad.

I: What can be done to make it good, what other facilities can be provided?

R: Facilities can be provided like you can say to the nurse that which will be normal you can try to do it normal or you can give some time to the patients or you can say to the doctors that you are giving medicine without touching the patients as there is rush, if you can give medicine after checking properly by touching then the patient can be cured in short time.

I: Is doctor sits here everyday?

R: Yes doctor sits everyday.

I: Do you think if more doctors sit here then will it be beneficial.

R: doctors here

I: If many doctors sit together?

R: many at a time, two doctors sit, there are two-one sees ladies one gents, then there is one eye doctor, then there is one for kids, sits in the new outdoor, so if there are many doctor then also it will not be possible to check well because the rush will be more, will be jam means what this doctor is saying what that doctor is saying will not be understood.

I: You said earlier that you want the illness not to occur means you want to be saved from disease. Is that?

R: Yes

I: What you prefer more to visit doctor after the illness or the illness not to occur?

R: No we want that we don’t have illness so that we don’t need to go to doctor again and again because we also need time, our is single family, if we fall sick who will do the household work, who will cook for our husband that’s why we always try to be healthy.

I: for this do you do anything at home for this means regarding health, for not to be sick.

R: There is lot like they take medicines from here and give us for loose motion or other thing, many times they go to our home, and going at our home they ask and give us medicines, after taking that medicines people gets cured.

I: Do you do anything for not to fall sick again and again like I am having cough and cold again and again, to prevent that you do anything?

R: For that we do like we don’t ‘jol ghata kom kori’ [meaning try work less with water]or try to save from cold, other thing for doing which i am getting harmed, we do that less like if I am doing this am facing more problem, family members also understand that for doing this it is happening again and again then I do that work less or I have to take care of my body, eat healthy, there are many people who don’t take care for work may be the breakfast which we needed at 9 am we take at 2 pm for that also people fell sick, so to take care of that.

I: I see. Anything else you want to say about this?

R: No, nothing else, I said a lot.

I: Thank you
