# Supplementary material for: “Without antibiotics, I cannot treat”: A qualitative study of antibiotic use in Paschim Bardhaman district of West Bengal, India
Source: PLoS One. 2019 Jun 27;14(6):e0219002. doi: 10.1371/journal.pone.0219002 (PMC6597109; doi:10.1371/journal.pone.0219002)
Supplement: S2 File — (ZIP) [file pone.0219002.s002.zip › S2_Transcripts/V6.docx]

**V 6**

Gender: Male
Age: 42

I-Your age please.

R-I am 42+

I-Who are there at your home?

R- Me, my Mrs and my son.

I-Generally what kind of illnesses do you see at your home or surroundings?

R-If I say at home like in some case everybody is having fever, cold, cough everyone suffer from this, these are mostly seen at home. Sometimes there is stomach problem also like loose motion so we suffer from this kind of problems.

I-If you see something at your neighborhood?

R-Neighborhood, see we can’t get news regarding everybody, everybody is busy with their own work so in this case if there is someone close and share with us then we

I-Come to know

R-Ya, come to know.

I-I see, as you said about the diseases like fever, cold, cough so what is the reason of these illnesses? Why does it happen?

R-If I say like people are irregular in many case, they are irregular then there is weather, for weather many people can have cold cough. So these are the

I-Means how the weather?

R-I mean weather is changing like there is cold and suddenly there is summer , so in such case it happens and in some case I said irregularity, for that there is cough and cold.

I-In which season it is mostly seen?

R-Specially in case of mine, am speaking about myself, in winter I will have cold, cough, sometimes I get headache but if I take rest for sometimes then it is cleared.

I-In winter it is mostly seen.

R-Ya, in winter it is mostly seen. Recently I had body ache, this happens to me sometimes.

I-If these happen where you do go?

R- If these happen, there are some medicine kept at my home, that I kept after consulting doctor, these medicine are there at my home, like if there is loose motion where will I go in that case I have some medicine at my home, I use that.

I-So you don’t prefer to visit doctor?

R- Doctor means I did not face such situation, If I see I am having fever and it is not curing in two days then I go to doctor, I consult with doctor.

I-Otherwise?

R-Otherwise I said medicines are kept at home and we use those medicines.

I-what medicine do you use in that case?

R-In case of there is Paracitamol, for gas there is Rantac, Gentac, gelosil and combiflame.

I-there is combiflame?

R-Yes, then in summer Glocon D is used, sometimes we use sugar-salt water that is in summer.

I-For stmach problem which medicine do you use? For loose motion?

R-Metrozil

I-If there is cough and cold

R-I don’t remember the medicine for cough and cold, a small tablet, I don’t remember the name.

I-Is it cerzin?

R-What?

I-Cerzin

R-no its not cerzin, a small tablet, I don’t remember the name.

I-Ok, leave it. You said that if the fever don’t comes down for 2-3 days then you go to doctor. Then what kind of doctor do you visit?

R-General physician.

I-Means hospital, or health centre or PHC or private doctor?

R-no, near to our house there is Akalpur health centre we go there.

I-Health centre.

R-Yes our councilor told us that there the treatment is good so if anything happens to you, 1^st^ go there. So in case of my son I went to the health centre so the general physician who was there saw every report and said go to our main govt hospital at Asansol. So we visit there. But when we came to Akalpur I saw there is very good arrangement, they take care, I liked it very much. After seeing the doctor told I am not seeing such symptoms. After 2-3 days they came to our home and saw our household environment, gave us various kind of advice.

I-They came from hospital?

R-Yes, from hospital. They said don’t let the water stag at your house, if you see someone else advise them to put some Kerosin or pora mobil in that water then the germ will not be there, it will be destroyed.

I-Generally you go to hospital or private doctor?

R-No, I said you Akalpur is near to my house.

I-so you go there in every case?

R-Yes.

I-I see. You said that you keep medicine at your home by consulting a doctor so before how many days did you show that to the doctor? If you can say averagely.

R-Before how many days?

I-Yes means before how many days you showed it and kept that medicine?

R- May be 6-7 months.

I-So then he gave for the illness?

R-Yes

I-If something like that happen then you use it again?

R-Yes and I have a friend Khokon who runs a medicine shop, sometimes I take tips from him , he is at Asansol, Aghoripara, he is known to me, very close.

I-The ones you keep at home do you check the expiry date of that?

R-Yes, obviously. My Mrs is such that if brings a food, a biscuit she will check the expiry date 1^st^. In many case I don’t check sometimes I overlook, in that case my Mrs will ask what did you bring today, that’s the list, checking the expiry date she will say the expiry date is over, go and return it.

I-So as you are saying you keep the medicine for 6 months. So what do you do to that medicine which is already expired?

R-We throw them.

I-Means is there any particular place for that?

R-Dustbin

I-Dustbin?

R-Yes we throw at dustbin.

I-Tell me one thing when you visit doctor, does the doctor make you understand anything about the medicine?

R-about medicine, yes.

I-What do they say?

R-They say if there is headache don’t take medicine at 1^st^, the much you avoid medicine is good,1^st^ you see, wait then at last if there is such condition then take medicine, the much you avoid medicine is good means you keep distance from medicine, when you see you can’t bear anymore then take medicine.

I-When you go to doctor then what do they generally ask, the doctors?

R-Doctor asks for how many days do you have this then they check many things like eyes, tongue, they ask for how many days it is there? When my son had dengue then they asked whether I had been to outside home either Kolkata or somewhere else. I said no sir; I did not go such place. They said there is nothing to worry, I don’t find such symptoms, the symptoms in case of dengue occur, and dengue is such a virus that don’t exists after eight days as the doctor said, within eight days it will show its power.

I-Do you know about antibiotic?

R-Antibiotic, I heard that in many case surgeon give antibiotic sorry the general physicians.

I-From where did you hear?

R-Doctors say antibiotic is given with medicines.

I-What do they say?

R-They prescribe and say take this antibiotic.

I-Do they say the rules like how you have to take for how many days? Do they say like that?

R-Yes they give that like take this for two days, take this after food, before food, take this two per day, one in morning one at night.

I-When you buy at medicine shop do they make you understand?

R-Yes, they also say, they write on the envelop, they write like this is in morning, afternoon and night, three per day, this after lunch, this after dinner, this one is before food.

I-If you get cured before the said date then do you continue the, medicine?

R-see me for myself if doctor gives me for three days and I get cured I will take that for three days. If doctor say you come after three days, or four days, say me how you are in that case I go and give the report like sir this is the thing, I have completed the medicine as you said and now I am cured.

I-Do all the family members do so?

R-Hmm

I-So do you think there is any side effect of medicine?

R-See, there must be side effects of medicine; I heard medicine has side effect in some case. But I heard that who take homeopathy there is no side effect. I don’t know that in details, how much it is true.

I-In case of antibiotic did you hear there is any bad effect of antibiotic?

R-No, I did not hear such.

I-What else do you know about antibiotic? When it is given or in which it works or why especially antibiotic is given?

R-see I don’t know about it in details still am saying antibiotic is given to destroy some germ, this is I am saying.

I-Yes, you give your opinion whatever you think.

R-Yes I think in this case it is given, now there are many kinds of fever, ‘jorer kono thik ney’[meaning there are various kind of fever] earlier when I was kid, there was a doctor at our village who used to give a red medicine marked like take up to this mark at this time, we got cured many times with that medicine but now as the days are passing the diseases are growing.

I-What is perception about misuse of medicine? Regarding misuse. Does it happen ever? How does it occur?

R-What are you meaning by misuse of medicine? Suppose it is given but am not taking, in that case?

I-It can be. What it is according to you?

R-See in that case I never faced the need of that I always take the given medicine.

I-If I say irregular use of medicine then what is your opinion about irregular use of medicine?

R-Irregular use of medicine is not right, many people take medicine for something simple, in small case, in small case, I have noticed this in many cases, suppose there is something, there is headache he took Saridon

I-According to you what is the harm in this case?

R-there may be side effect, must be, in many parts of body there can be side effects.

I-Do you know name of any antibiotic as you use?

R-No

I-what is your preference-homeopathy or allopathic?

R-My choice1st I used to see homeopathy, but homeopathy don’t suits me so now at present I prefer allopathic.

I-If there is sudden emergency then what do you do?

R-emergency means?

I-Suppose there is any emergency, at night means you became sick then what do you do?

R-If there is any emergency if it suddenly increases then we go to hospital. Nothing to do if it is that much serious then hospitalized.

I-Which hospital?

R-I said you Akalpur, Akalpur health centre , their treatment and if they refer to the govt then we go to the govt [referring to ADH]

I-When you are going to any hospital for some illness then what do you think, with what expectation do you go there?

R-See in my case, what I am saying we go there to be cured, I am having some problem, doctor gives me medicine and I get cured, a belief, we go to doctor to be cured. Is no it?

I-What else do you think? Means what will he do, what do you think?

R-Doctor , he will see. After seeing he will give medicine accordingly.

I-Did you ever feel that the medicine you need is not given by doctor?

R-no, I did not feel so ever.

I-Or you did not get cured after taking doctor’s medicine? Does it happen to anyone to your family that he did not get cured after taking medicine?

R-Yes it happened in case of my son, I visited a doctor 1^st^, he I will not say the name

I-No no

R-So he checked and said take this medicine for three days, he took but the fever was still same, the fever means may be decreasing and again risen up at night, then I visit one at asansol, he gave medicine and got cured. Then I interacted to this brother, my son was hospitalized, I interacted with brother. There they said the same like we do not fine any symptoms so there is nothing to worry.

I-Do you ever say to the doctor that write this medicine or write good medicine?

R-No, whatever doctor thinks he writes.

I-Does doctor give any test before giving any medicine?

R-Yes

I-Is it done in every case or in some cases?

R-not in every case. It happens in some cases like blood test, urine test, sometimes they say for the X ray.

I-Is antibiotic used only in case of us or in case of others like animals, birds etc?

Pause

Do you know if it is used in case of birds or animals?

R-It can be, we are alive they also, there is no difference, it is same. It can be, why not.

I-do you know such thing?

R-I don’t know but I think it can be.

I-how to make people aware about the proper use of medicine or the proper use of antibiotic? How can they be aware? In what way? How can the information be spread to more people?

R-That you have to [pause] promote, advertise.

I-How

R-It is to be spread among people, have to gather people and discuss, in this way it will be means I know and I have to tell others, in this way it will be promoted, through public opinion, and media.

I-If I ask you what is your opinion about the irregular use of medicine?

R-I will say medicine should not be taken irregularly for there can be great loss to health.

I-how will you combat that? What do you think?

R-You should tell not to take irregular medicine who is taking irregular medicine, in this way you have to make him that taking irregular medicine is harmful for health.

I-Do you have quack doctors around you?

R-No

I-Medicine shop?

R-There is medicine shop.

I-When you are taking medicine from chemist shop then you always give prescription or sometimes you take just by saying?

R-No, in some shop I have seen without prescription they don’t give medicine.

I-what is the case at your place?

R-By seeing prescription.

I-There is little bit cough cold and you take medicine from there, does it happen?

R-No, taking medicine by saying is not right. Medicine should be taken by showing doctor’s prescription. I have seen in much case that I visited doctor he wrote prescription and that medicine is not available so went to doctor and said that sir this medicine is not available but there is other medicine and he told me to ask what other medicine then I showed him again and sir said It is not needed now.

I-Means they don’t give medicines by themselves at shop?

R-No

I-Thank you.
