# Supplementary material for: “Without antibiotics, I cannot treat”: A qualitative study of antibiotic use in Paschim Bardhaman district of West Bengal, India
Source: PLoS One. 2019 Jun 27;14(6):e0219002. doi: 10.1371/journal.pone.0219002 (PMC6597109; doi:10.1371/journal.pone.0219002)
Supplement: S2 File — (ZIP) [file pone.0219002.s002.zip › S2_Transcripts/KAP 1.doc]

Interview: KAP1

Gender: Male
Age: 24 years
Education: H.S. pass
Occupation: Chemist shop owner/pharmacist

I: From when have you been running this shop?

R: Normally, I don’t sit here.

I: Normally, you don’t sit here?

R: I don’t sit here. Papa sits here, and…Papa has gone out today. *[inaudible- truck horns blaring in the background. Respondent seems very hesitant to communicate openly- keeps deflecting to the fact that his father is the one usually runs the shop*].

I: I see, I see. So, what do you normally do?

R: I have a shoe shop.

I: Oh, you have a shoe shop? I see, I see…what kind of antibiotics do you keep in stock? [*respondent is reading paper*]. Go ahead and read- no problem. [*pause*] so what kind of antibiotics do you keep in stock?

R: Antibiotics…we have a lot.

I: Such as? Which ones?

R: Cipr…ah…you have your Norflox. After that…uh…[*inaudible name*] lots of ones like that.

I: And, how do you decide what stays and what goes? I mean, this must change, right, from year to year? What does this process look like?

R: Based on our knowledge- for example, we see that there is a fever and it is not going away. [*chattering going on in background]* is not leaving. Or there is a cold and fever and it’s not going away.

I: Yes.

R: So we give it accordingly. However the doctor gives- we follow that and give. For example, the doctor gives all this, so we follow that and give [medicine].

I: For example, so you see prescriptions coming and a lot of cases of one kind are coming- based on that, you prescribe medicines?

R: Yes.

I: I see, I see, I see. And, regarding antibiotics, how many do you think you have in stock?

R: I don’t have a good idea. I have been here for around 2 months.

I: Around?

R: I have completed two months here.

I: You’ve completed two months here?

R: Yes.

I: So where do you order medicines from?

R: We order it from Asansol. [Indistinct word]

I: From Asansol? So, from the wholesaler?

R: Yes.

I: I see, I see, I see. And, when does the medicine come? Once in a week, twice in a week- how often?

R: We go three times per week.

I: I see. And if you have to change things- for example, if you need something else, you tell them directly?

R: Yes.

I: I see. And do you have knowledge about antibiotics? Meaning…knowing what broad-spectrum antibiotics are, the antibiotics that work for a lot of, I mean…[*respondent interjects:* yes, illnesses] works for a lot of medicines. Works for a lot of illnesses.

R: I don’t know that much. [*inaudible chatter drowning out respondent]*

I: So, tell me this…[oh yes-*had to pause recorder while he handled a patient]. [Resuming recording]:* Yes, so, you were telling me about these broad spectrum antibiotics.

R: Yes.

I: So, do you ever give those? You know, the antibiotics that work for many illnesses. For example, you have TB. There is one specific antibiotic for TB.

R: Yes ,that’s specific-

I: [*cuts off respondent and keeps talking]*-that’s only with a prescription.

R: Yeah, that’s only with a prescription.

I: So suppose someone is coming to you, and they have a cold and fever. They have something like that and they don’t have a prescription. And you have to give a normal antibiotic- so do you ever give that?

R: Yes, we do give.

I: Okay, so what all do you give?

R: We give Norflox and [*inaudible recording*]

I: So with that, you don’t need a prescription? Meaning…

R: No, no.

I: Oh, no? I see, so…suppose someone said that they have a cough, cold, or runny nose. What [medicine] would you give for that? Or, what do you do in that case? And it’s just that. Cough… cold… or there is a runny nose.

R: Yes, there is a running nose.

I: So what would you give for this?

R: For this, I would give Norflox.

I: Yes. Give it just like that?

R: [*nodding yes*]

I: I see. And if there is this…if there is a fever?

R: For that, for fever also…meaning if there is a fever and it is not going away. Meaning it is coming and going…after going away, it is coming back again.

I: Yes.

R: So then we would give it.

I: Otherwise, you wouldn’t give it?

R: No otherwise, we don’t give it.

I: So if it is coming and going, what do you give? The same thing?

R: Yes [*nodding yes*].

I: And if you have this, loose motion, you know the watery one.

R: Yes.

I: And you have vomiting or not. What would you do then?

R: We give Safeguard (**check spelling**) for that.

I: Which one?

R: Saveoguard (**check spelling**).

I: I see…

R: And…

I: Oh yes, tell me you were saying something.

R: With Ciplox, we also give [inaudible medicine name].

I: And if there is stomach pain? There is a patient coming to you who tells you he has a lot of stomach pain. Nothing else.

R: There is just a medicine for that, for ladies and for gents.

I: I see. So what do you give?

R: For ladies, we give Cyclopam. And for gents, we give Nustobam or [Isonid…inaudible name].

I: So if someone comes to you and says they have stomach pain, this is what you would do.

R: Yes. Meaning, it depends on why the stomach is hurting. The stomach can hurt if you need to go to the latrine as well, and even normally.

I: So you would ask about all this?

R: Yes we would ask.

I: I see, I see. And this all goes even when there isn’t a prescription? Meaning…

R: Yes.

I: And lastly, if a patient comes to you with rashes?

R: We give a cream or ointment for that.

I: Is that a topical antibiotic or just an ointment?

R: No, just normal.

I: I see. So, not an antibiotic?

R: Yep.

I: So how do you decide when an antibiotic may be required?

R: We see, suppose the medicine is not working [for the rash]. Not working even after you put it on.

I: Meaning after you give a normal medicine, if it isn’t working?

R: Yes, yes. After that, we give antibiotic and then normal medicine.

I: So, first you give normal medicine to the patient?

R: We give normal medicine.

I: In every case?

R: In every case.

I: Even if there is a fever?

R: Yes, even then.

I: I see.

R: If it’s still not getting better. Then, we decide to give antibiotic.

I: So if you give antibiotics, what kind of course do you give? Meaning- a full course, 1 day course? How do you give?

R: Around 2-3 days course.

I: 2-3 day course?

R: Yes.

I: And if the patient does not complete this course…says that he got better after one day. What does the patient do with the rest of the medicine?

R: [*long pause*]. He should continue for two days maximum [*respondent appeared to intend to say for at least two days rather than maximum two days]*.

I: I see. So you tell them that they should complete the full course?

R: Yes. Yes we tell them that they should take the antibiotic for around 2-3 days. So we give it, but then the patient says ‘no we just want a one-day course.’

I: I see, I see.

R: That’s according to his wishes.

I: So according to his wishes, you give him a one-day course?

R: Yes.

I: If he asks.

R: Yes.

I: Uh…suppose a patient comes to you. Can you tell me what a typical interaction would look like? Suppose I’m coming [as the patient]- I just came to your shop. What kind of conversation would you have? What all happens? What happens first?

R: We say ‘what happened? What kind of problem?’

I: Yes.

R: After that, they say ‘I have a fever’ or ‘I have this problem.’ We talk to the patient accordingly. So, if the patient says they have a fever, we ask: for how many days have you had a fever? And what else do you have? Tell me what all you are feeling. [*loud truck horn blaring]*. Does he have a fever…a cough…we get the details and then we decide what medicine to give.

I: I see, I see…in this process, when do you see the prescription? If It’s there, you give according to the prescription. Or…?

R: When the prescription comes, we see it.

I: I see, and if the patient doesn’t have it, then you just talk to them.

R: Yes, then we just talk to them.

I: So, in what situation would you send the patient to the doctor, meaning, to tell them and go and get a prescription? [*other conversation happening in the background*]

R: If a patient requires sleeping medication, for those things.

I: For sleeping medication?

R: Yes for this, we ask for a prescription. We tell them we can only give the medicine if they have a prescription.

I: Without it, you don’t give the medicine?

R: We cannot give.

I: Okay, but if a patient has fever or cold, and they say they have not been to a doctor, and…they say ‘I don’t have fees to go to a doctor, please give me medicine.’

R: Yeah, we give medicine [*very nonchalantly*].

I: So you give it?

R: Yes, we give.

I: And, in that situation, would you give a normal medicine first or an antibiotic?

R: We would give ‘normal.’

I: And what would you tell the patient?

R: If the fever gets better, then good, or else come again in two days.

I: I see. So you tell them to come again.

R: Yes. [*long pause with chatter in the background*]

I: Your, I mean uhhh….what kind of confidence do you have when giving medicine to patient? Meaning, do you always know this is the antibiotic you should be giving. Or, that you should do this in a particular situation. Or do you have some doubts in your mind?

R: We ask for how many days the patient has had a fever. Then, only we can decide, right?

I: Yes, yes.

R: According to this [what the patient says].

I: But, you are confident of how to act in a particular situation?

R: Yes.

I: I see, so do you ever get doubts? In terms of, I mean…suppose you don’t know there is a fever and other symptoms. And you also don’t know which antibiotic to give. Does this ever happen?

R: No, this…it used to happen occasionally before, but now it doesn’t happen.

I: Doesn’t happen now, I see. And if it happens, what do you do? If you don’t know what medicine to give.

R: Then, we don’t say anything.

I: Then you don’t say anything?

R: No.

I: So what do you tell the patient in that situation?

R: We tell them, do one thing, go show the doctor, we say.

I: And if the patient says no, we don’t have money to show to the doctor, or we don’t have time to go to the doctor. So what would you do in this situation?

R: We would just give them normal medicine.

I: Okay. You don’t give an antibiotic?

R: Nah, we don’t give antibiotics.

I: Okay, tell me this. The drugs, you know, the ones that are nearing their expiration date…what happens to them? Suppose there is an antibiotic that is expiring in one week, what happens to it?

R: We don’t give that antibiotic.

I: You don’t give?

R: No,

I: Then, what happens to that antibiotic which is nearing its expiration date? Do you give it back to the wholesaler or what happens?

R: There are some medicines that the wholesaler takes back and there are others that they don’t [take back] from the company.

I: I see, and what happens to those they don’t take back?

R: We have to throw them away.

I: Throw them away? How are they disposed? [*pause*] Where do you throw it, do you know?

R: We throw it away in the drain.

I: You throw it away in the drain, I see. Okay, and do you do this yourself or do you give it to somebody who takes it back?

R: Nah nah, nothing like that.

I: No one does it. You do it yourself?

R: [*mumbling*] We have to do it ourselves.

I: I see, I see. [*long pause as patient approaches*]. How many times do you, approximately…go ahead [*pharmacist gets busy with a patient*]. How often does it happen that a patient comes and he is not completing the full course [of antibiotics]? Or that you are not giving a full course of antibiotics. How often does this happen?

R: That many patients have not come yet. When they come, then I will know.

I: So however many patients have come to you till now… have you given all of them a full course of antibiotics?

R: Yes we have tried to give a full course, but while taking the medication, they ask us to give only one day’s course.

I: Only asking for a one-day course. Meaning, they don’t want to pay for it.

R: Yes they don’t want to pay for it.

I: So, in that situation, you give them a half course? A 1 day course?

R: Yes, a 1-day course.

I: How often has this happened?

R: Must have happened about 4-5 times.

I: 4-5 times?

R: Yes. [*blaring horn in the background. Indistinct chatter.*]

I: In a day, how many patients do you see approximately?

R: Now we have a new shop, so approximately, 10-15 patients.

I: 10-15 patients? From those, how many ask for an antibiotic? Would you know? Or how many do you give antibiotics to?

R: About 2-3.

I: In a day? 2-3 in a day.

R: Yes, yes.

I: I see. What kind of relationship do you have with the doctor? For example, there is the doctor, patient, and the pharmacist. All three have a role when it comes to antibiotic prescription, right? Meaning each of them has a part. So what do you think- what kind of dynamic exists between them?

[*continues talking]* If there is antibiotic resistance- have you heard of antibiotic resistance?

[*long pause with chatter in the background- did not get around to answering the question*].

I: What kind of dynamic exists between the doctor-pharamacist-patient? What are your thoughts about this?

R: Between the doctor and pharmacist?

I: Yes. In terms of medicines, meaning antibiotics.

R: I can’t say anything about this.

I: I see. So, with respect to antibiotics, are there lots in the community or less? Based on the need, is there more or less in society?

R: More than what is required.

I: I see. Why do you say that?

R: Because if the fever isn’t going away, you have to give a antibiotic after waiting for a few days.

I: I see.

R: Now…what is happening…

I: Yes, tell me.

R: If the fever is persisting for 2-3 days and not going away, we have to give antibiotics.

I: We have to give [antibiotics]? So, in terms of this, are there lots of antibiotics out there or few antibiotics?

R: 50%

I: 50%?

R: Hm (yes).

[*long pause*]

I: Okay. Would you like to say anything about this? About antibiotic medicines? Meaning if we want to stop…have you ever heard of antibiotic resistance? When the medicine does not work for the same illness? Suppose there is a medicine that works for a fever or to reduce diarrhea, and you keep the giving medicine and it is not working.

R: I have not heard anything about this.

I: You haven’t heard anything about this?

R: No.

I: I see. And there a few medicines that are not working against TB now. Have you heard anything about this?

R: I don’t know anything about TB.

I: You don’t know? Okay and regarding any other diseases? Have you heard anything about this?

R: No.

I: Okay. Thank you very much.

Field observations:

There were two gentlemen sitting in the shop when we walked in, one of whom was only present temporarily. Both were very willing to talk to us, but each had an excuse regarding why they may not know the answer to some questions. One of them claimed he doesn’t sit regularly in the shop, while the other said he had only been here for two months. Both eagerly filled out the questionnaire, asking questions along the way, and the person who normally distributes medicines (since the past two months) agreed to participate in an in-depth interview. He appeared to contradict himself in a few places and did not seem to be all that familiar with antibiotic resistance. He was very willing to give us his time, but continued to see 1-2 patients in between the interview as they came to the shop. Despite being located near a main road, the shop did not seem to be overly busy.
