# Supplementary material for: “Without antibiotics, I cannot treat”: A qualitative study of antibiotic use in Paschim Bardhaman district of West Bengal, India
Source: PLoS One. 2019 Jun 27;14(6):e0219002. doi: 10.1371/journal.pone.0219002 (PMC6597109; doi:10.1371/journal.pone.0219002)
Supplement: S2 File — (ZIP) [file pone.0219002.s002.zip › S2_Transcripts/KAP 118.doc]

Interview: KAP 118

Age: 48

Education: Higher secondary

Gender: Male

Occupation: IHP

Workplace: Private clinic

I: Tell us a bit about yourself. How long have you been working here?

R: Here, it’s been 17 years for me.

I: 17 years?

R: Yes. Before that, I was in Bihar for 10 years.

I: How did you get into this line of work [RMP]?

R: I got into this work…for example, even in my village, there are 4 practitioners. By watching them…

I: Meaning, in Bihar?

R: Yes, in Bihar. I got training in nursing home, trained under a doctor. Was there for 5 years. After that- *long pause*

I: Approximately how many patients come to you every day?

R: There is no such time. It depends on the season, right? Right now, it’s the off season. 8, 10, 15?

I: 8, 10, 15 people? Morning, or…?

R: More in the evening.

I: More in the evening? You only sit two times?

R: Yes.

I: You sit right here [in this clinic]?

R: Yes.

I: And do you also do home visits?

R: Only- only if there is a critical case. If a patient cannot come to the clinic…then I go.

I: Critical case meaning? what kinds of illnesses?

R: Like the patient isn’t in a condition to come to the clinic. He is vomiting, or he is too old- he cannot come. In these circumstances, I go.

I: Normally, what kind of illnesses do you see most commonly?

R: Who will really come to us? People who have a cough, cold, cough, fever, vomiting- those kinds of patients.

I: Only these kinds of folks come to you?

R: Yes, just these kinds of folks.

I: And what about the critical cases you were speaking about?

R: Those who are vomiting, or who are not in the condition to come to the clinic. Cannot get up from the bed. Those conditions. *Long pause.*

I: Suppose a patient comes to you. How- meaning from start to finish- how would you treat this patient? What do you ask them?

R: I only get general patients who come to me. Cough, cold, fever mostly. I check how many days he’s been feeling these symptoms- by understanding this, I give him medicine. *Long pause*-

I: So, uh, for each consultation, how much do you take? For each patient?

R: It depends on the patient’s condition or position. Accordingly I give him time.

I: Can you give me an example? Such as-?

R: Suppose a patient has fever, cold, cough- first I check his fever. How much or not? Okay? After that, I see what other kinds of problems he may have. Trouble breathing or any other problem. After checking everything, I see and give him medicine.

I: So as you said- a patient comes with cold, cough, and running nose. For such a patient, what would you typically do? *Pause.* What medicine would you give?

R: So, I would see the patient depending on his position, right? [*Impatient tone.]*

I: So if those are the only symptoms. A patient comes- with a cough, cold, and running nose. So what would you give him in this situation?

R: I would give him cough syrup. I would give him an antibiotic if it seems necessary, otherwise just an anti-allergy medicine like a **Paracetemol**. Or something else.

I: In what condition would you give him an antibiotic?

R: If he is having a lot of difficulty breathing- phlegm has gathered- bronchitis is causing a lot of trouble breathing- in these cases, you can give him an antibiotic. If he is having a lot of trouble, then we can give.

I: And with respect to antibiotics- do you give the full course or…splitting up in 1-2 days? How do you do it?

R: No, I give him a 2-day course only.

I: Only a 2-day course?

R: Yes.

I: Why?

R: To see whether the patient has gotten better or not. The position can change- if it is going well, then we can continue for 5-7 days.

I: This, uh- in this condition- such as cough, which antibiotic would be appropriate?

R: *interrupting quickly*- **Amoxicillin**.

I: And?

R: Mainly, this is what is being given right now.

I: Amoxicillin?

R: Yes.

I: And do you also give a combination? Combination antibiotic such as, you have **Amoxiclav**. So do you give this as well?

R: Yes. But very little.

I: Very little? Why?

R: Double antibiotic is being stopped.

I: Being stopped? Meaning, is it being stopped in the market, or…?

R: Not in the market. It’s causing trouble. More side effects, which is why it is being stopped.

I: I see. What kinds of side effects can you see?

R: That much I do not know. We are just a provider. When the government does some research on this, and is seeing that it is causing more side effects and trouble in patients, they are stopping it.

I: Okay- so if a patient comes with a fever- what would you give in this case? Only a fever, nothing else.

R: **Paracetemol.**

I: Paracetemol? You wouldn’t give an antibiotic in such a case?

R: No.

I: And if a patient is having loose motion? With or without vomiting?

R: If it is happening with vomiting, then **inaudible medicine names** Norfloxacin Tindazole.

I: I see. And if there is a stomach pain?

R: There is **Cyclopam, Mepthylspaz,** depending on the condition, if it’s worse you can also give **Dotin?**

I: But normally, you would give an antibiotic?

R: What is the need for an antibiotic in this?

I: You don’t need it?

R: Not for a stomach ache.

I: Uh- if there are rashes? On the arm or leg?

R: What?

I: Like itching, rashes-

R: **Livocitrazil**. Some kind of anti-allergic medicine.

I: Hm hm- do you ever give a topical antibiotic?

R: No.

I: The cream that you have-

R: *long pause-*

I: So have you ever seen a situation where you are giving a patient an antibiotic, and the antibiotic is not working later on? Because the bacteria in the body, they have become resistant to the antibiotic. So have you ever seen anything like this?

R: No.

I: So most patients who come get better from the medicine?

R: Yes.

I: I see. Does the patient ever come back to you to say the medicine is not working? Never happened?

R: No this has never happened.

I: I see. The medicines you give here- uh, so where do these medicines come from?

R: We get this from the Medical- meaning from the medical shop.

I: I see, so the medicines that are getting expired- what do you do with those?

R: I throw them away.

I: You throw them away? Where?

R: Here- out back-goes away.

I: I see. In the drain?

R: No there is a carton- we throw it away in that. And then it goes away in the drain.

I: And what is already done, you do that to it? Meaning the ones that have expired you throw like this? What about the drugs that are near expiry?

R: I throw it away one month before.

I: One month before the medicine is set to expire?

R: One month before.

I: So how often do you check the medicines?

R: Before giving medicines, we check- for antibiotics. What the date is.

I: Sounds good. Does an MR (Medical Representative) ever come to you? From the companies? *Nodding yes.* So what are they like? What do you talk about?

R: They say that- to prescribe my medicine- it is great- but we do not have the power to prescribe medicine like that. We can only give according to our capacity. I have information, but however much we are allowed to give based on our certificate- that is all we can do. So they come and go- many come.

I: So uh- what kinds of antibiotics may you have taken from the MR?

R: The simple ones- Ampicillin, Amoxicillin, like uh… Cifodoxin, all of this.

I: So where do you find out about antibiotics? One is the MR, then do you take information from any other sources?

R: I have information from my 5 years of experience at the nursing home. I have understood all that. Plus, there are some books that come.

I: Books?

R: Hmm.

I: I see. When you disburse antibiotics, do you prefer generics or brand name?

R: Brand.

I: Brand- why so?

R: Generic doesn’t work.

I: Generic doesn’t work? You always give brand name?

R: Yes.

I: Why do you think generic medicines do not work?

R: I have seen after looking at patients. The results don’t come. And if we give the brand-name or the same medication, it works well on the patient.

I: It works, I see. And you were talking about the combination medicines- so do you give combination antibiotics frequently or not?

R: No, no.

I: No, you don’t give? Hm- have you ever heard of broad spectrum antibiotics? Broad spectrum?

R: Broad spectrum as I was saying about the cough medicine.

I: No, as in, you have your Amoxicillin- one medicine that work for a lot of illnesses right? It’s not like a specific medicine.

R: No not narrow.

I: Yes, so do you give broad spectrum antibiotics usually, or…?

R: Mostly, this is what is given.

I: This is what is given? I see. Why?

R: We cannot provide such medicines generally. Suppose we give a high antibiotic to a patient- the patient does not get better and he goes somewhere else. The doctor will see who prescribed this medicine. What book keeping does he have? On what basis was this medicine given? Did you have the power to give this medicine? What will I say at that point?

I: Hm hm- so you avoid giving?

R: Yes.

I: So, if…in what situations do you give antibiotics? Meaning, why do you give antibiotics?

R: For instance, if someone’s wound is not healing, for the wound to heal, we give antibiotics. And suppose, what do I say, for a cough…if there is a lot of phlegm, if it’s a pneumonia patient, you can give it in such a case.

I: So suppose you have 10 patients coming to you in a day. How many would you give antibiotics to?

R: Average we can assume 50%.

I: 50%? So you give antibiotics to 5 out of every 10 patients?

R: Yes.

I: …*pause.* Mostly, what kind of illnesses do you see?

R: As I said, cold, cough, fever, vomiting, etc.

I: And if we see in other seasons?

R: In other seasons, what, well I mean you see vomiting more in the hot season. Comes along with diarrhea. Loose motion?

I: Mhm. So what do you give during a diarrheal episode?

R: Like I said, Norflox-Tindazole

I: I see. Do you ever give ORS?

R: Yes we give ORS.

I: But, is it given along with antibiotics or?

R: Yes. Why do you need antibiotics during diarrhea? You don’t give?

I: What is the need?

R: If it is very critical, then we can give an injection. We have stopped doing any kind of stitching.

I: So, uh, do you have any kind of RMP association? Which everyone belongs to?

R: Yes from where we did our CMA course **inaudible names** …

I: So does everyone meet ever? In one place?

R: No, no. We did this uh…class right? After we did the course, we had an exam. After the exam, we got a certificate, which is renewed in 3 years.

I: But you all never meet in one place every year or anything?

R: No. No. That doesn’t open.

I: I see. I see.

R: There was an association, but it got cancelled.

I: Mhm mhm mhm. Among the people who come to you, do you give medicines to everyone or do you just write a prescription for some people?

R: If I don’t have the medicine, I write a prescription for it.

I: And if you have to refer somebody, then?

R: There is a referral- to the doctor in Raniganj. If I cannot understand what is going on, I told him somewhere else he can go.

I: In Raniganj?

**nodding…long pause**

I: If a patient is coming, and after coming to you, he saves the empty medicine packet and goes to a pharmacy to get more medicine in the future- how would you handle such a situation?

R: Meaning, I don’t understand- **phone ringing**

I: Meaning, as I was saying, suppose a patient is coming, and you gave him medicine. And then, do you, do you ever tell them to come back after two days?

R: Yes.

I: To see?

R: Yes.

I: So do patients come back?

R: Yes.

I: So that medicine packet- does it ever happen that patients take that empty strip and go fill the medication themselves at a pharmacy?

R: No.

I: This doesn’t happen?

R: No.

I: I see. So they always see a doctor first?

R: Yes.

I: Okay…so when it comes to prescribing antibiotics, some roles are very important? Such as a doctor, a nurse, RMP, pharmacist, etc. So what are all of their roles in controlling or disbursing antibiotics?

R: I don’t understand. What did you say?

I: Earlier, we were talking about this- resistance- when a medicine does not work for the bacteria. If we want to stop this from happening, what can each individual do? If we want to reduce resistance? Each individual meaning doctors, nurse, pharmacist, whoever gives medicine-

R: First of all, whatever antibiotics there are that people are prescribing, has already started affecting the body. You have to reduce the power first. If you start off with a high (powerful) antibiotic, a low (less powerful) antibiotic will not work. So first, we have to start with the halka (low power) antibiotic. Then, the situation will be controlled. What you are seeing now is that whatever medicines are being put out there by MBBS doctors- a 1 year old child is being pumped with 250 injections- there’s no nothing- **mumbling**

I: Who is doing this? MBBS?

R: MBBS yes or…we do not have the power to do this. After seeing the prescriptions, I am able to see the amount of powerful antibiotics that are being prescribed. Right now, it is still in control, but in the future, the patients will face more trouble…*pause*…they have the power, the license, they can do anything they want **laughing**. Right or not? I have seen a prescription with 500 injections for a one year old child! Imagine! What will happen to the child? ...We will never give.

I: So, uh…

R: This should be 125.

I: So, to stop this…?

R: **interrupting** To stop this, only they can do something! What can we do about this?

I: I see, and what about the chemist shop owners? What can they do about this?

R: They also cannot do anything. Whatever the doctor writes, whatever is on the prescription, they give out.

I: Do you ever feel like a patient asks for antibiotics without a prescription? Taking medicine without prescription?

R: Only the medical person or medical shop owner can tell you about that. They don’t come here for that.

I: I see, they don’t come to you for that. So, does it ever happen that a patient is coming to you to ask you directly for medicine, that I only want this particular medicine?

R: No.

I: Doesn’t happen?

R: No- okay.

I: And if someone asks, then what do you say?

R: I don’t give **emphatically and without hesitance** I don’t give. Why would I give?

*Long pause.*

I: The medicines that the MR recommends- do you only give that medicine or what are your thoughts on this?

R: No, no, I will only give what I like.

I: So, how do you know whether you should be giving the medicine that the MR recommends or some other one?

R: I do have approximately 20-22 years of experience. What medicines are present, what is available and how they are working- that much I have knowledge about?

I: But what about the medicines that are newer on the market? The new medicines that are coming out? [14:09]

R: You may have a new company, but where does the medicine change? The medicine is the same. The company may change, but the medicine, the composition will be the same. So it depends on the company- whichever company is giving the best result, that is the one we would go with, right?

I: What is your fee structure for the patients?

R: Nothing like that. After giving medicine, we add 5-10 rupees on top as fees.

I: I see. So do you ever find out whether patients are consuming medicines in the proper way or not?

R: Yes. We call them back after two days. Call them back to see how they ate it, whether they ate it or not-

I: Meaning you ask for the empty medicine strip?

R: Yes, for the medicine I gave.

I: I see. And if someone is incorrectly consuming medicine, then what do you do?

R: Then, I explain to him that the patient will himself not get better if he doesn’t take the medicine properly. By checking the medicine, we know [whether it has been consumed properly]. So suppose I gave medicine to a patient and he says he is not feeling better. Well, go bring me the medicine? I see that all the medicine is still there, so how will he get better?

I: So, how much time do you typically take for this process of counselling one patient? To explain to them?

R: Depends on the type of patient. 10 minutes, 20 minutes…15 minutes. **phone rings- long pause**

I: I had another question- what is your, what is your confidence level that you are giving the appropriate medicine to the patient?

R: I have 100% confidence.

I: In everything? In terms of all the illnesses being presented?

R: No, not for every illness. Only for the general illnesses- suppose a fever is coming and is not getting better. If I don’t know, I would order a blood test to see why the patient is not getting better. I would investigate- based on the way the report comes, I will do the treatment accordingly.

I: So, the report that you ask the patient to bring- do you see the report yourself or-?

R: I only look at the report.

I: I see. So, in what situation would you send a patient to a specialist?

R: I am giving medicine, and the patient is not getting better. Say, someone has a fever. I gave first aid and medicine, and the patient did not get better. He ate it for 2-4 days and did not get better. So I require a blood test in that case.

I: Do you see antibiotics count as first aid?

R: No, no.

I: No? So first you will always do first aid, or first…?

R: Yes. No, first, I will only do first aid. Then, I will give antibiotics. If the patient is not relieved after that, I will order a blood test.

I: So if there is a fever patient- I am asking again- so what would you do first? Would you give Paracetemol?

R: Yes, I would give Paracetemol.

I: I see, I see.

R: I will have to give a halka (low power) antibiotic along with that- **ampiciliin**, **amoxicillin** I will have to give.

I: I see….what other antibiotics count as “halka” antibiotics?

R: Like, you have ampicillin, amoxicillin. What is below that? **Ceftrin** and all have been stopped-

I: I see, and if the condition does not improve with all of this- then?

R: If it does not work after all this, we do NOT have the power, but even then for some people, we will give like…**Cefadoxin**, **Cefaxim**, all this can work…but we do not have the power to give.

I: Okay. A little while back, you were saying that by giving strong antibiotics, you can get some side effects as well. So, if you are giving someone an antibiotic, are you thinking about the risks or the benefits for this when giving medicines?

R: When we give, there is no side effect. As in, when it it’s given outside, what the doctor is writing when it is high dose, so the patient comes to me to show how the prescription has been written and how it is causing problems. So, I tell him it is happening from the medicine, so go and show it to the doctor. Why would we fix that?

I: So, you think that there are no side effects from the medicine you provide?

R: No, no. We never give powerful antibiotics.

I: So do you think that this resistance concept we talked about- do you think it is caused by these less powerful antibiotics or not?

R: Meaning, suppose it is a 5-7 days course. You only take for 2 days and leave it. The medicine then won’t work on you.

I: I see. So it also happens with the less powerful antibiotics?

R: Also happens with those.

I: What other reasons might there be for resistance?

R: Nothing more- what more will there be?

I: Like one reason as you mentioned is not eating the medicine properly. Is there any other reason why the medicine may not work later on?

R: No nothing like that.

I: Do you do any minor surgical procedures here, such as suturing?

R: No.

I: No? Okay. Um…would you like to say anything more? If we want to stop resistance in the community, in the country, do you know of any guideline- ?

R: **interrupting** If we want to stop it, then I would just say to reduce the proportion of high power antibiotics. In the beginning, we should not be prescribing powerful antibiotics right away to patients. Go with a low power antibiotic, but if the patient is not feeling better, then we think of something else. By directly giving a high power to patient, he will face problems later on.

I: Regarding the training you had, was there ever any mention of resistance?

R: No it was never mentioned.

I: Never mentioned? And what was said about antibiotics?

R: Antibiotics, just all of this- which kinds of antibiotics we are allowed to give: mostly **Ampicillin, Amoxicillin, Ciprofloxacin**- all of this has been given.

I: And were you told about any risks related to this? Risks and benefits, which Rina was telling you about? What negative things may happen as a result?

R: No never heard anything in terms of antibiotics.

I: You never heard, okay- one last thing- have you heard of any government guidelines related to antibiotic use? Such as- you should give this, you should not give that?

R: Yes, from the government, we have gotten a certificate and have been educated that you should not go beyond what you have been given. Whatever has been informed to you is what you should be working with.

I: Okay, okay. So, if there is ever a similar training in the future, would you want to participate?

R: Even if we participated, what could we do about it afterward? We cannot do anything. I know how to do everything. But I cannot do.

I: Why can’t you do it?

R: I don’t have the power/authority. The certificate does not give us that kind of direction or power from the government. You can only do what is within the power of an RMP.

I: Yes. **pauses** Suppose someone is coming to you- you are thinking you need to give him an antibiotic, or any other medicine, but they do not have that much money. So how would you treat them?

R: I would give it anyway. For money matters, the patient will not leave empty handed from this chamber. Whether he has money or not.

I: Would you like to say anything more related to antibiotics?

R: The only thing I would like to say with respect to antibiotics is that the more powerful antibiotics that the MBBS doctors and MBAs are prescribing- only they can control this, if they like.

I: So does that mean that the problem of resistance is mainly coming from them?

R: Yes coming from them…as soon as you leave the medicine, you will have this [resistance].

I: A little while back, you were saying that for some people you were giving only 2 days stock of medicines…so if that patient who needs it for more days does not come back to you, how would you handle this situation?

R: I move the patient onward- I tell the patient to move on.

I: Meaning?

R: **with frustration, in a loud voice** Ahead, I tell him to move ahead! If you are not going to eat medicine properly, with direction, you can go on and show someone else- go see a bigger doctor.

I: Okay- thank you.
