# Supplementary material for: “Without antibiotics, I cannot treat”: A qualitative study of antibiotic use in Paschim Bardhaman district of West Bengal, India
Source: PLoS One. 2019 Jun 27;14(6):e0219002. doi: 10.1371/journal.pone.0219002 (PMC6597109; doi:10.1371/journal.pone.0219002)
Supplement: S2 File — (ZIP) [file pone.0219002.s002.zip › S2_Transcripts/KAP 160.doc]

INTERVIEW- KAP 160

AGE- 48 years

GENDER- Male

OCCUPATION- Pharmacist

HIGHEST EDUCATION- DIPLOMA IN PHARMACY

I: How many years you are working here?

R: 9years

I: 9 years. Are you here from the beginning of your job? [cut off by respondent]

R: My job? [Asking]

I: Yes

R: No. In Kolkata

I: From the beginning in Kolkata and then here?

R: Yes. [*mumbling inaudibly*]

I: Okay. How many years you are working here?

R: Here 9 years. As a pharmacist I have 25 years of experience.

I: As you are working here as a pharmacist, which type of antibiotics do you keep in your stock or you have with you?

R: Amoxicillin is there. Amoxicillin, Cloxacillin, Ciprofloxacin, Semporn etc. But Semporn actually is not an antibiotic, it has chemotherapy agent. Sempron is actually Cotrimoxazole.

I: Which one is mostly used?

R: Mainly Amoxicillin and Ciprofloxacin.

I: In which cases these are mostly used?

R: It is mostly used in deep injury. And antibiotic is need for the viral infection that are staying for long time. I wait for 2-3 days. If it is simple allergic case like cough, cold, runny nose and if it is symptomatic then I wait for 2-3days. If it is not curing by anti-allergic drugs like cetirizine then in that case I start antibiotic.

I: Okay to [cut off by respondent]

R: For diarrheal cases norfloxacin is needed. These types of antibiotics are available here. I am the pharmacist; this is the truth [hesitation]. I can’t treat all kind of patients are coming here for treatment. Medical officers [MO] can treat. I am telling it on the basis of antibiotics use for the patients coming here for treatment.

I: Does MO is available here?

R: No.

I: Okay. Do you treat patient?

R: Yes.

I: So mainly what types of antibiotics do you prescribe? Keep in stock?

R: That I already told you before. Only those antibiotics we keep.

I: When you prescribe antibiotic to patient do you keep anything in mind? What do you think before prescribe antibiotic to a patient?

R: For any deep injury, or any ulcer not curing from long time in that case antibiotics are used like amoxiclav, amoxicillin and cloxacillin. Other than that the viral infection are staying for long time we have to give antibiotic as a guard to reduce the chances of typhoid.

I: Okay. From where do you get antibiotic?

R: It is supplied by District Reserve Store [DRS] from Burdwan. All the PHCs and BPHCs of the district receive drugs from DRS. In every district there is one District Reserve Store. Till now no DRS is available in Paschim Bardhaman. We receive drugs from Purba Bardhaman. It has to be in Asansol after division of district. In other districts there is one DRS, like in Bankura district there is one DRS is available.

I: As you told us no medical doctor is available in the PHC then do you treat the patient? So in that case how do you maintain supply chain of antibiotic? How do you disburse medicine from the store.

R: Can’t understand. [Didn’t understand the question].

I: When you are taking medicine from the DRS in which basis do you order the medicines?

R: I check the demand of medicines before giving order. Also check the expenditure of whole year. We have to give the demand list of medicine in the beginning of the year in April. According to that we get medicine quarterly. Now we have to do it online. We send our demand to DRS directly through online. There is a fix date for each centre. We are under Khandra BPHC. So for all the PHCs under Khandra BPHC has a fix date to order for medicine in DRS. We have to send our demand from PHC to the BPHC at least before 10days from the given date.

I: So you [Cutting of the interviewer off]

R: They [the BPHC] keep ready the medicine from the morning. We have to bring it according to our demand. We have to check all the medicines are supplied according to our demand or not. Then we bring the medicines to the PHC by car.

I: Which antibiotic do you prescribe more to the patient? Like broad spectrum antibiotic. Do you prescribe broad spectrum antibiotic?

R: Yes. It is mostly used.

I: What is your opinion about broad spectrum antibiotic?

R: So I have to say for any particular illness [confused]

I: Your opinion on the use of broad spectrum antibiotic.

R: Mens good or bad? It is good. It is alright. If I prescribe broad spectrum antibiotic for the disease then everything is covering [All the infections]. Nothing will be incomplete. It is very useful for killing of all types of bacteria. It [broad spectrum antibiotic] is very helpful for complete cure. To kill bacteria broad spectrum antibiotics will work effectively.

I: Why the broad spectrum antibioticds are using more in the community?

R: There are good and bad effcts. In allopathy you need to compromise. If I use it [Allopathy] to cure disease then there will be some side effects. Sometimes it kills helpful bacterias. It happens sometimes for antibiotic. The most harmful is “I don’t know do you have any question regarding this or not” people are buying from the shops for 2-3 days. Even if I prescribe they are leaving after 2days. There is a side effect. Drug resistance is very difficult, means irregular use of drugs. After taking 2days they are not taking as they are feeling better. They are prescribed for 5days but not completing the dose. The problem is there. This is happening in regular basis.

I: What is the availability of broad spectrum antibiotic in the community?

R: What type means? [Didn’t understand]

I: Means what is the availability?

R: In our DRS?

I: No in the community?

R: Means what is the demand in the community? Very much demand. Demand means first they will tell you that prescribe the medicine that can cure me quickly. The patients come here don’t know about antibiotic. They tell to give good medicine for quick recovery. This is not only the scenario of my place. This happens everywhere. Sometimes they have been treated by higher antibiotics by some doctor to cure the disease quickly. So, they can be renowned in the community. It has demand [in reference of broad spectrum antibiotic] in the community. Cure me quickly that’s all they [in reference of community] want. We can start with Cifran but Ampiclox work quickly. But why do I change high dose of antibiotic at the beginning. But nowadays, the patient does not have any patience. [Inaudiable voice] We will be cured in 3days. The demand is everywhere.

I: Did the patient ask for antibiotic from you? [*cut off by respondent*]

R: They don’t know about antibiotiuc. But actually they want that thing. They can explain by their attitude. Give the medicine that can cure me quickly. For boil or any injury they want to be cured quickly. Maximum number people don’t have knowledge about antibiotic. In community most of the people are illiterate or primary educated. I gave it to you already [he means to say in survey paper he choose the option].

I: So they say give me medicine that can cure me quickly?

R: Yes. They say give me good medicine. There 2-3 types of words. 1. Give me good medicine. 2. Give me the medicine that can cure me quickly. These types of demand are here.

I: What do you do in this case?

R: See if I think it [disease] is small then I start with Sepron. If the patients come with serious disease like ulcer then I have to start with Ampliflox. They sit at the home for 10-15days thinking that the disease will be cured. When they come to us it already became serious. That time we can’t take any risk.

I: How long the medical officer is unavailable here?

R: 4 years.

I: In that case [*cut off by respondent*]

R: Sometimes MO visits here. He was in contract basis. Again he was sent in deputation to other place. Then he left. This was happened. We don’t have MO for 3-4 years. Dr. Saha was here but after that no one [silent]. We already told if someone is arranged in contract basis for 2-3 days in a week would be good for us. We can tell patient. We can’t take risk if the patient has pressure, sugar. If the patent is old then we can ask him to come on the date doctor will be here. Rest of the day we can see normal diseases like noral fever, cut, wound etc. In that case we can also tell patient to come on the doctor’s day after giving primary treatment. If he come for2-3days would be helpful for us.

I: So how much you are confident when prescribe antibiotic to a patient? Or when prescribing antibiotic to patient [*cut off by respondent*]

R: Confident means if you tell me about curing rate of disease in that case very good result. The patient comes to me; they also know that [showing finger to his collegues] telling that I heard from someone that you give good medicine. The patient has been cured. Prescribe me also the good medicine. This is truethere are some diseases that only cured by antibiotic. You have to giveantibiotic. But we can wait for somedays before giving antibiotic. Like sometimes reason of cold and cough is allergic in that case we should wait for somedays. In some cases it can be cured by cetirizine, paracetamol. No need of antibiotic then. This is my thinking. What I have learned during survey in my study on pharmacy. [*smiling*]

I: How much do you keep combination drugs?

R: No combination drug is available. We have to do the combination. There 1-2 combination drugs like Pantapazole with domperidom. Other than that I don’t have any combination drug. We have to give it separately. Means I have domperidom and other antacid like Omiprazole. So i give both sepeartely. These are same thing. I don’t think combination is needed. But people are get addicted by this. We have demandof Pantop-D. 50% of patient come here take the medicine. They think without the medicine they can’t digest food. We explain them many times that the medicine is not for regular use. Take the medicine only when you have acidity. But they don’t understand. We have to give 1 strip means 10 tablets [smiling]. They think the medicine is helpful for digestation. This is a problem. Sometimes they don’t understand anything.

I: You are saying people are getting addicted of combination drug?

R: No I didn’t mean that. Not only combination drug. For some medicines that are good for digestion. They [patient] think that if they take the medicine then only they can digest otherwise not. For 2days there was problem but at the 3^rd^ day there was no problem but they still continue the medicine. It is difficult to manage conventional thinking of the people. It is also true for educated people. Give me that medicine. Without that medicine disease is not curing. It’s like the medicine of sleeping pill. This is true that Pantaprazole and Omeprazole combination is working well. As because of Domperidone combination it is working well. That’s why people come to take that medicine. No one is taking Omeprazole. They say it will not work give me that medicine. I have 2 combination drugs. One is Coxacillin with Amoxicillin.

I: That you keep, in your stock [*cut off by respondent*]

R: I make the combination for rest of the drugs. Paracetamol is light painkiller. I can’t charge Brufen for light pain. But idf there is any severe njury then I have to charge Combiflam or other combination drugs that are available in the market like paracetamol with brufen combination. For me in severe injury I give both patracetamol and brufen separately to make a combination and an antacid with that oaky.

I: Antibiotic prescription depends upon 3 people, Doctor, pharmacist and a patient. In that case could you please explain me scenario of your PHC?

R: Means MO is not here. I have to do everything. So That is not applicable here.

I: So in that case coud you tell me the relation between you and a patient at the time of prescribing antibiotic?

R: Patient doesn’t understand about the antibiotic. After hearing of the symptoms from patient I have to decide about the medicine. I already told you in the beginning. Suppose for simple viral I could not understand that is it viral or allergic? If there is runny nose with other symptoms then I wait for somedays. I start with Cetrizine and prescribe Paracetamol if headache is present. I tell them to come after 2-3 days. If I see it is staying after 2-3 days and not curing then I have to start antibiotic. In some cases it cures without antibiotic. Actually antibiotic has no role in viral infection but we have to give. If it is staying more than 3-4 or 6 days then our WBC [White Blood Cell] will be busy to kill that viral infection. That time other infections like typhoid, dysentery attack the body and no one will be there to protect the infections. In that case we have to give antibiotic. If I see patient is not curing after 5-7 days, it can turn into typhoid so; we have to give antibiotic as guard. We need some soldiers to kill the infections. In that fear I give antibiotic. This is the rule. But I think antibiotic shoudn’t start at the beginning. What is the benefit to prescribe antibiotic at the beginning for simple cold and cough? It has a side effect. What is the need to give antibiotic without reason? This is my view what I have learned from my professors during my study diploma in pharmacy. Now the experience is be using in the practical field.

I: The antibiotics you are stocking do you check once in a year [cut off by respondent]

R: No. We do the quarterly indane for the medicines. Once in a quarter. We have to submit whole year demand list in April. They want all the expenditure list of medicines like what is the use Amoxcillin or what would be need in future. We have to submit the need of paracetamol, septorn in a year. During quarterly indane again we submit the demand if there is any need. Suppose any medicine is already is in stock. We don’t need to indane that medicine in the next quarter or if anyting in shortage then we have to order for more. We submit our demand in DRS [District Reserve Store] and send it to our center by car. The system is properly working. Here is no problem in the system. All the mediciens are available. The only problem is absence of MO who prescribes the medicine. Why the patient will come here? He needs a MO. This is also true that if the higher authority gives us permission for prescription then everything would be solved. But we don’t have a written permission for writing prescription. If the authorities think that we are capable to treat the illness then they can give us permission. Then we can practice more effectively. What I told you already that surgery I can’t take any risk for elderly people who has heart problem or come with ECG or need to do bypass. In that case MO is needed. The patient is telling me he has sugar, pressure. I don’t know howto treat those patients.

I: As you told that there is no written permission for doing prescription then how do you manage?

R: I can do the treatment for small illness. Like field staffs are working in the field. Our ANM staffs are giving medicine in the field. In that case we don’t need permission. We are also giving the same medicines and we are also treating that kind of illness. For severe illness we tell them to go Khandra PHC or go to Ballavpur RH. Ballavpur is near by. There doctors are present. I am handling as much I can do. For street accident or for any cut antibiotic is not needed [smiling]. During dressing of wounds I have to do everything. I also prescribe some medicines. These are okay. For stomach pain I tell them to wait for 1-2 days. After that I prescribe medicine. As field staffs are giving ORS in the field same as I have to do. If anything getting severe then I refer them to ballavpur hospital or khandra hospital.

I: Then patient [cut off by respondent]

R: The problem will be solved in future. It will not continue for long time. MO will come in future and that will be helpful for everyone. For me, for patient all will be benefited. Previously we worked with doctor [keep sentence incomplete]. I worked with doctor at the time of my joining in the PHC. We worked jointly. I was in Kolkata at the beginning in Sagar Dutta State General Hospital. Didn’t face any problem there. That was a very big hospital with 131 beds. We were 4 pharmacists working over there. In some prescription if we found the child is needed half dose but was given full dose then we spoke with doctor to change it. We all worked together. We co-operate each other in the work setting. In that big hospital sometimes we bought medicine from outside if government had the crisis of medicine. We did survey that which medicine are using more or which is needed more. This is still continues. I have been transferred here [Smiling]. Definetly everyone [in reference of patients] receive the medicine. According to me system is okay. There is no problem.

I: What does it mean by big hpspital?

R: Near by ballavpur rural hospital. This is not far. It is situated in Raniganj. Also our Khandra BPHC, Durgapur sub divisional hospital and bardhaman medical college.

I: As you have indane the drugs quartertely then how do you manage the nearly expiry drugs?

R: If I find that then give it to BPHC. If I find these medicines are not distributing and expiry date is nearly 2-3 months then I send it to BPHC. There patient load higher than my PHC. More than 200 patients visit there in daily basis. In my PHC only 30 patients pay visit in a day. Naturally demand will more there.

I: Where do you give?

R: In BPHC. That is the rule. When I was posted in big hospital there medicine store was adjacent to the hospital. We returned the extra stock after taking consent from hospital superintendent. They used from there otherwise it go back to the DRS. This is the rules.

I: Means you telling about nearly expiry drugs.

R: Atleast we have to check 2-3 months before of expiry date [smiling]. They [in reference of BPHC] have to spend it also.

I: What about expired medicine?

R: Till now it doesn’t happened in my PHC. I told you we don’t have that much patent as no MO is sitting here now. Here I indane only small amount of medicine. What should I indane so much medicine unnecessarily. I don’t need to take large quantity of medicine. Suppose I orderd any medicine for 3 months and within 3 months the stock has finished then I have option to bring it from BPHC. I don’t have any problem. If I indane in BPHC then they will give us. It has been toldby DRS also. DRS only supply medicine to BPHC if there is any shortage but they will not give to PHC directly. So we get from the BPHC. No problem is there.

I: Do you think patient demand for antibiotic contribute to overuse?

R: Absolutely. Till now as per my experience I am saying that it is happening.

I: Could you explain the scenario of your PHC on the basis of previous comment?

R: Here patient only tell us that give me medicine that can cure me quickly. Maximum number patient coming here with cut, ulcer, boil. Cure me quickly means they want to say give high dose of antibiotic. I can’t cure the illness quickly by giving paracetamol. [Phone ringing]

I: So patient demand for antibiotic.

R: Yes.

I: You prescribe by yourself

R: No I don’t have right to do prescription. I just maintain the stock and keep track how many medicines are given in daily basis. The pharmacist has to know how many has been spent. We have to maintain the stock in store. How many spend and present stock of medicine. I just maintain the expenditure of medicine with the name of the patient in the copy. I write the name of individual drug and what is the expenditure of the drug.

I: How do you expalain patient about the proper useof antibiotic whren antibiotic are given to them?

R: We have to explain the dose. The process of taking we explain. Also tell them not to drop single dose. Take the medicine regularly. We have to explain in their language that this is antibiotic and has to continue as prescribed. Don’t drop the course. We explain them the rules of taking antibiotic. They don’t understand antibiotic. They only understand that if they discontinue then the illness will not cure. This is the easy way to explain them.

I: Do they understand?

R: In this case two types of patients are there. One type patient is concerned that I have to cure the disease. They obey what I say. Another type of patients take medicine for 3days. They think he said normally, doesn’t need to take for 5 days. Why to take for 5 days as I have cured in 3 days. Two types of patients are present.

I: What is the failure rate?

R: Many [Smiling loudly]. There are patient who has been treated in velor but cured by my medicine. This is very much mental thing.

I: What percentages of patient don’t complete the course?

R: It is difficult to understand. Patient don’t come back and give report all the time. But still my idea is 70-30%. 70% take the medicine. They take correctly. 30-40% patient don’t take regularly. As I have spoken with the patients. [Phone ringing]

I: What do you do in that case?

R: We have to explain them again that you have take the dose regularly. Otherwise the medicine will not work in future.

I: Is there any case like that? Patient takes antibiotic and didn’t cure.

R: Yes they come. We have to change the medicine then. Suppose I have prescribed Septron at the beginning but don’t cure then I give amoxicillin or ampilox at the next step. The percentage is rare. More than 80% patient got cured by the medicine given from here.

I: All?

R: They got cured. The patients come to me when there is need or there is any acute problem.

I: As you told me before that you asked patient about symptoms before giving medicine right. Is there any facility to do culture test?

R: If there is need then we suggest them to do culture test. Now the dangeruious thing is dengue. If some one comes with fever, body pain, joint pain in that case I refer them directly to ballavpur hospital. Doctor is present there. Doctor will suggest you if test is needed. I don’t have MO. We give the suggestion. The test is needed in this case then we start the treatment. Otherwise tell them to go to khandra BPHC as this is under Khandra. Ballavpur is nearby. Doctor will tell you if test is needed. On that basis your treatment will start there.

I: Do you perform any test before giving antibiotic?

R: In what matter [didn’t undertand]

I: Do you perform any test if someone is needed to prescribe antibiotic?

R: I can’t give that kind of antibiotic from the PHC. I don’t have that [in reference of high dose] types of antibiotic. In some cases I ask patients like some patients have allergy in septron. I know the patient by name and for others I ask them do they have any allergy if prescribing frist time. Sometimes they tell themselves that they have allergy in that case we don’t prescribe. Test is not needed as he knows about that. In some cases we take the precaution. We tell them to report me after 2 days. In sulfar someone might have allergy but it is nothing. In septron chances of allergy is rare. Don’t need to perform test for the medicines are available in my PHC.

I: What do you do in that case?

R: I refer patients to khandra If I find the case isn’t curing by me, higher antibiotic is needed. Tell them to go for tratment under doctor’s supervision.

I: Do you know about antibiotic resistance?

R: Yes.

I: If you explain me about that.

R: It has been seen after many researches that the dose of the medicines is fixed according to the illness. In 7days all infection has been killed. In that the dose of the medicine is fixed for 7 days. Now you stop it after taking 3 days. In that case some bacteria still remain in your body and they create the protection against that drug. Next time the drug can’t kill the bacteria. In that case the drug has been resistant to that antibiotic for that individual. This is called resistance. This is the problem. Now this thing has become a big issue. This is the tendency of maximum people in our community. They don’t want to listen our opinion. They think that we say uselessly to take the drug for 5-7 days. It is only needed for 3days. The illness has cured then what is the need to take for long. This is the problem of the patient now. We had a seminar on Antibiotic Resistance in Asansol few days before. It is organised by pharmacy association.We had discussed lot on the topic of Antibiotic Resistance in the community.

I: What is the effect of antibiotic resistance in the community?

R: The effect is eual in the village and urban areas. More or less everyone is effecting from antibiotic resistance. In urban area if the percentage is 50 then in village area it is 70%. Urban people understand little more about the issue as they are eduacate. In village people say we came to the hospital to cure the illness. Now we have cured and why are you giving me more medicines? Actually awareness is needed. But it is difficult aware people individually. It will be good if mass of people are called for any seminar or program. Then if you explain them the issue broadly that will be effective. If you explain them broadly then it will be useful than individual counselling. This is needed everywhere. Not only in the viallage areas but also in urban areas the awareness is very much needed.

I: What arethe reason of antibiotic resistance?

R: I only know little thing. People are abusing. They go to the medicine shop and take medicine for 3-4 days. Also shop keepers are giving the medicine without prescription. Why they are giving medicine without prescription? It should be given according to doctor’s prescription. Why they sell less number of medicines than the prescription? I saw that. Doctors wrote to take medicines for 20days but the patient is buying for 5-6 days of medicine. They say [in reference of patient] that “Give me medicine for now and I will come back later to buy rest of the medicine. I don’t have money now. Give me for 3days.” People will not come back if cured. Some didn’t take medicine knowingly. I gave 10 talets they didn’t take all. It is just neglegency. Awareness is needed. If you don’t take full course then the medicine will stop working later. It will become resistance. Are you thinking anything about the issue? [Asking question to the interviewer]

I: We are conducting survey to know the situation. We would take some steps to prevent antibiotic resistance after completion of survey only.

R: Definetly you should think how to aware people. You are also working like that.

I: Do you give full course of antibiotic to the patient? Or you give 3 days [cut off by respondent]

R: If I give Ampilox then I give for 10days. For symptomatic illness generaly give medicine for 3days like paracetamol. If fever doesn’t persist then no need to take medicine for long. If it [paracetamol] is needed to take for 3 times in a day then I give 10 tablets. For symptomatic treatment taking medicine for 3days is okay. Also cetirizine is needed to take 1 at night. In that case full course is not needed.

I: Did you give the full course once at a time or call the patient after somedays when antibiotic are prescribed for their illness?

R: No for Ampilox I give full course. If someone comes with severe illness then also I give full course. It is risky to let them go with half dose in this situation. Now for nearby people whom I know closely give antibiotic for 3 days and then tell them to come after 3days. I started with septorn in that case [didn’t complete the sentence]. Usually they come. I tell them to take medicine for 3 more days. Sometimes it also happens for Ampilox or Amoxicillin. Suppose I have a supply 250 medicines. I give them medicine for 2days then tell them to come after that. Sometoimes tell them to come after 3-4 days also. I have to do that becaue sometimes some medicine doesn’t suit to someone. They are needed to check after 2-3 days. Mainly I tell them to near by patients. They come because they know me. Otherwise we give them full course.

I: Now I am telling you some name or symptoms of diseases. Like cough, cold, runny nose. Plese tell what are treatments you give for these diseases?

R: I told you already [in reference of survey] that I wait for 2-3 days and give only anti allergic medicines. After that if I see it is not allergic but viral infection then I strart antibiotic. Otherwise it can turn into typhoid. That time the diffence mechanism of the body will be busy to kill viral infections and can’t prevent the attack of any bacterial infection at a time. It is their work. In the absence of them [WBC] the bacteria can attack the body. In that case we have to use antibiotic as a gurad. It is not needed to start at the beginning if it cures by anti allergic medicine. Why to give antibiotic if it is not viral?

I: In case of fever.

R: There are two reasons of fever. One is cold, allergic. Not theviral all the time. We have to wait for 2-3 days for all types of fever. If it stays more than 2-3 days then it is viral. In that case we have to give antibiotic. Antibiotic can’t kill viral infection. Our sir [in reference of doctor] told us “that it will go after 7 days if take medicine or it will go in one week if you don’t take medicine.” Reason is that. We have to give it as a gurad to prevent other illnesses.

I: If watery loose stooi, without vomiting

R: Antibiotic is needed to start. How can you stop it with ORS? We can wait for sometimes. If the frequency increases then we have to start. Suppose we vcan wait for half day. You can give ORS. According to the rules it [ORS] should be given immediately. After that if the frequency increases then saline needs to start with antibiotic. We refer this type of patient to the BPHC.

I: For stomach pain

R: We ask them the symptoms. Is there any gas or not, pain in chest or not, exact place of pain etc. In that case we give antacid. If he feels burning then I give antacid. It has two types of work do you know that. One type of antacid works to netural the acid in the liver and another type of antacid is like Pantop-D. It works as a blocker. They prevent the secretion of acid. The acid stored in mass cell. It is HCL. The antacid blocked the secretion of HCL. It has to take early morning before taking tea. In another case where seceretion has started in that case we have to give Pantaprazole, Omeprazole. If it became more serious then I can’t treat the patient [smiling]. I have to refer means I need to ask any doctor please check the problem.

I: If come with rashes?

R: Sometimes rashes come out in dengue. Can’t take any risk. If it is simple rash, itching mostly common in this winter then I can give anti allergic. If they take it 2 tablet at a time then also it doesn’t have any harm. If the patient comes with fever, rash and other complications then that is different case. I can’t treat.

I: Do you prescribe any antibiotic?

R: No.

I: Tropical antibiotic?

R: No.

I: What are steps we have to take to prevent antibiotic resistance?

R: I am starting from the beginning. I have objection about the pharmacy shop. They give medicine without prescription. Also they use old prescription to give medicine. They don’t check when the doctor has prescribed the medicine. The patient is coming with old prescription. That time he [patient] had been cured with that medicine, next time patient also coming with the same prescription to buy medicine. May be this time the reason of illness is different. He doesn’t need antibiotic this time. He needs the same antibiotic for present illness as hehas been cured previously with the antibiotic. Give me these medicines. In medicine shop you will find the shop keepers are mainly 8^th^ to 10^th^ standard literate. They give the medicine. It should be stoped. They [patient] ask for 3 days or 6days of medicine. They have to give full course of medicine if they are giving medicine to patient. Doctor prescribes 6 tablets then I can’t give you less than that. Take medicine according to the prescription. Also it is needed to improve general awareness to complete full course. In this case there is minor difference between literate and illiterate people. It is not that educated people understand more about the problem. Those are the illiterate people they don’t take and the literate people thought it is useless to finish the course. I am not blaming anyone. Public are not aware about the matter. If some drugs become resistant then only we can use higher dose. That only we can do. If some drugs are not working in 70-80% cases then survey is needed. If any drug not working in 70-80% cases then the drug should be banned in the market. In this case alternative drugs should be introduced in the market.

I: Do you think prevention is needed?

R: Means from patient side [didn’t understand the question]

I: Everyone. Means what are preventive measures are needed to prevent antibiotic resistance?

R: I have already covered all of the pints. Prevention means you have to obey the prescription of doctors. I [in reference of patient] have to accepet that though I am highly educated but also I have less knowledge in this matter. If I go to a software engineer for a work then in that case I have to obey the instruction of that person [instructor] because I am less knowledge about software. Here is the problem. If I am going to a doctor or health center to cure my disease then I have obey each and every instruction of them. I have to follow what they have asked me to do. Everyone is a doctor now [smiling]. I told them [patients] sometimes to sit in my chair. They ask me to give medicine by name. Give me paracetamol or give me azithro or amoxicillin. Give me that medicine. It will cure my cough and cold. They [pointing finger to his colleagues] know everything. Give me 20 Amoxicillin. They are above the doctor. If I try to explain something then their attitude is like give me whatever I told you. They are creating problems. If we can manage them then the problem will reduce. Everyone has to take medicine according to the doctor’s prescription. Medicine sell without prescription should be banned immideately. It is actually difficult part. People come to buy medicine like give me that medicine which had cured me 6 months ago. Even for DOTS medicine there is so many defaulter patients. It is same for them. They don’t continue the medicine. Agin they have to revise the medicine. It is a very tough treatment. They have to takeso many medicines.

I: Is there high resistance for DOTS patients?

R: It is not that. For DOTS it is a problem. Maximum resistance occur in our normal medicine. But that has severe effect on our community. The resistant person can spread the disease to others. That’s why government is taking strong action against that [in reference of DOTS]. The cause is less awareness in the community.

I: Why most of the people buying medicine from outside store?

R: There might be two reasons. First, to visit doctor they have pay fees. It is better to buy medicine from shop. Why to pay Rs. 200 to doctor. Another thing is they don’t want to stay in line in the government health centers. Also distance is a factor for them. Patient has to travel long to reach to hospital. I also get that type of patient. They took medicine for 2-3 days from medicine shop then come to me. I always tell them come to me at the beginning of illness.
