# Supplementary material for: “Without antibiotics, I cannot treat”: A qualitative study of antibiotic use in Paschim Bardhaman district of West Bengal, India
Source: PLoS One. 2019 Jun 27;14(6):e0219002. doi: 10.1371/journal.pone.0219002 (PMC6597109; doi:10.1371/journal.pone.0219002)
Supplement: S2 File — (ZIP) [file pone.0219002.s002.zip › S2_Transcripts/KAP 201.doc]

**KAP 201**

Gender: Female

Age: 50

Occupation: Nurse

Highest education: GNM degree

I: Okay sister so can you tell me a little about how your OPD setting is over here? Meaning what, what all happens, what kinds of patients come? Can you tell me a bit?

R: In our OPD…doctor- doctors come. And all kinds of patients come here. Patients with loose motion, fever…fever, stomach ache, body pain, like that all kinds of patients come. Mostly general patients come.

I: Mhm mhm mhm- So in a day, approximately how many patients must come to you?

R: Here in the outdoors, patients come- sometimes…50…50 patients.

I: 50 patients?

R: 50-60 patients come.

I: I see, I see. And the doctor who sees the patient, or you, meaning, how much…meaning, how much time do you spend with the patient? With one patient?

R: Until they keep talking to me- they tell us about their illness, I listen. More than that, I don-

I: *[interrupting]* Yes, but if approximately 50 patients are coming, so meaning how much time would you get per patient?

R: Mmhm…….*[pausing, hesitating]* 10…like 10 minutes.

I: 10 minute, I see. And in those 10 minutes, meaning what kinds of things do you talk about? What do you ask the patient?

R: Patients as they- as they come with their problems, after listening to those problems, I tell them do like this, don’t do like this, do like this, stay like this- I explain these things to the patient. However much time it takes to do that- that’s it.

I: Mhm mhm mhm mhm mhm. So in this- uh- can you give me an example? Like suppose a patient comes- okay?- with some illness. So first- what would you ask first?

R: *[interrupting]* Now suppose a patient comes with loose motion, so we tell them that you should keep things clean, you should boil water and drink, wash hands, all this – all this- you should stay clean. This is the kind of advice I give.

I: I see, I see. So you just mentioned loose motion. So typically what medicine do you give for loose motion over here? If a patient comes?

R: Mhm- we give Metrozil, we give Normetrozil, all this we give- we give ORS.

I: I see. Everything together?

R: Yes. And in that, what do you tell the patient…about the medicine? Like you have to eat it TDS- eat it three times in a day. And ORS- you have to mix a 500 mg packet, 1 packet in the water before drinking it. I give advice in this manner.

I: Do many patients with skin conditions come here? The ones who-

R: *[interrupting]* Yes, skin patients come, but there is no skin doctor here, which is why they return back.

I: So you refer them-

R: Yes we refer them- don’t keep…

I: Oh district hospital?

R: Yes- district hospital. We send to district hospital or send them to a skin doctor for advice.

I: I see, I see okay. And what kinds- I mean, what kind of skin problems do you see over here?

R: Mostly, patients with rashes.

I: Rashes? And you do not give them anything here as first aid?

R: No. No halka-phulka [light/low power] medicine, nothing?

I: Okay. And if a patient comes with a fever. What would you do in this situation? What?

R: For patients with fever, we check the fever. And then according to that, whatever antibiotic the doctor prescribes. We give that. For fever- uh- we give Paracetemol and an antibiotic.

I: In- what do you give in antibiotics?

R: *[long pause]* umm Amoxicillin, something like that- whatever is in supply, that is what is given.

I: Okay. So if a patient comes and he has a fever, so first what is given? First, what- what would- ?

R: *[interrupting]* Paracetemol. We would give Paracetemol, so the fever gets like this [reduced]. Do a little bit of sponge.

I: And do you give antibiotics right then or afterward…?

R: Afterward, the doctor prescribes it when they go home and eat it.

I: I see. But you give it right when the patient comes? In this, do you give the full course or…how do you give out antibiotics? Suppose you gave Amoxicillin-

R: We give it for 2-3 days first, otherwise we give it for 5 days.

I: And for 3 days, what will you say [to the patient], meaning you uh…?

R: Then the doctor- when he comes back, if the fever is okay then good, otherwise if the fever has not reduced or if there is a problem, then he comes a second time to show the doctor.

I: Suppose you gave an antibiotic after three days. For three days. If he got better after 3 days, I mean. It reduced. Then, would you give anything that is remaining, or?

R: If it gets better, then uh- we do not give.

I: You don’t give, I see.

R: The 3 day course, I think the doctors must be prescribing it for 3 days, mhm.

I: Okay.

R: But uh- *[hesitates]* it is the doctors who give advice, right? Meaning for how many days, etc., nurses do not give. *[laughs]*

I: If a patient has…loose motion, with or without vomiting, what would you give for that? What does a patient typically get?

R: *[long pause]* We give saline, etc. We will run a drip first. Because the patient is going into dehydration, which is why we give, and then we give, uh, for vomiting we give- Zofar, etc, Ibumin tablets that are there, injection, etc…Mostly, we give an injection only because if we give a tablet, he would vomit right? So we give a proper injection in IV. And give the IV fluid. We give ORS, etc.

I: *[pauses]* You give ORS along with it?

R: Mhm mhm- *pauses-* when it’s time to uh drink water, the patient is only given ORS water.

I: Okay. And is it common to give antibiotics here?

R: Mmmm no, like that no…

I: Not for loose motion? Do you see whether they are mostly giving it or not giving?

R: Some people are given, some people are not given. That normetrozil, norfloxacin, that is what we give here.

I: Okay why do you give that in this case? When you give-?

R: *[interrupting]* The infection like- so the infection does not get worse- that’s why we give.

I: So can you tell me- if typically 10 patients are coming, right? – among them, how many, uh, among the ones who have loose motion, would you give antibiotics to? How many get antibiotics?

R: According to that, however the patient is, depending on the kind of vomiting or loose motion that happens- some people have light, some people have a lot. The ones who have only a little we give accordingly, and we give antibiotics, etc, to the ones who have a lot.

I: Okay- who has a lot, they get anti-antibiotics?

R: Yess [*hesitant]*

I: Even then, if there are 10 patients are coming in a day, then how many of them will be given antibiotics? You can tell me an average. Approximately. *[shaking her head]* No? Okay no problem. *[respondent laughs nervously]*

Okay, if it’s a patient with stomach ache?

R: If it is a stomach patient…ummmm…he will get stomach ache medicine.

I: Okay.

R: And we don’t give any antibiotic or anything like that.

I: What medicine would you give?

R: And after that, then, the doctor will ask what kind of stomach ache do you have…gall bladder pain, appendicitis pain, only afterward- when- *[hesitating]* you will only find out after tests are done. In order to stop it…we would only give stomach pain medicine.

I: Okay- and the first time when he comes- what medicine would you give for stomach pain?

R: *[pause]* Drotin, etc. we give- Dicolic, Drotin-

I: I see- analgesic or something?

R: Analgesic.

I: Do you do- minor surgical procedures here like suturing, stitching? *[shaking her head]* Nothing?

R: Oh yes! Like mhmmmm…if someone comes with a cut/injury, we gave them stitches- that happens.

I: What all do you see here? What kinds of injuries do you see?

R: Something tore or got cut after a fall- if it’s a deep cut, it gets sutured- if not, we do the dressing and leave it.

I: In this- giving antibiotics is…?

R: *[quickly interrupting]* Yes you have to give. If it’s a deep cut, you have to give antibiotics, otherwise how will the wound get better? In order to get better, like to prevent infection, you have to give antibiotic.

I: So if any patient comes- meaning, who, has a cut here*,* so you…give him antibiotics?

R: Yes.

I: You give? I see….*[pauses]* okay. Is there a testing facility here or anything? [*nodding*] Lab, etc. is there?

R: Yes, yes, it is there.

I: Inside? In here?

R: Mhm mhm [yes].

I: So what kinds of tests do you do there?

R: Blood test is done.

I: What kinds of facilities are there in that? What all can you test, in the patient I mean?

R: There is a sputum test…blood test, sugar, umm mhm and…*[long pause]* HBTCCHR everything- everything like that is there.

I: Culture, sensitivity, everything?

R: Yes, cultu- *mumbling-*

I: So in this, is this used before prescribing antibiotics or is it not done?

R: It is done first.

I: Every time? [*mumbling in agreement]* Before giving antibiotics? I see-

R: It should happen that way.

I: Yes. But what happens in reality?

R: *[long pause]* If it’s a big thing, in order to control it, the doctors just give it [antibiotics]. He prescribes a test, etc, the medicine starts, or he does the test after-

I: [*interrupting]* Okay, so the test and antibiotics is done together? *[nodding]* But mostly what are you seeing? Does he prescribe the test first or give the antibiotic first?

R: Test should happen first.

I: Test should happen first? And it happens like that here? *[pausing, semi-nodding]* I see. And if a patient comes who has an upper RTI, a respiratory tract infection, so what is given in that case?

R: *[long pause]* Here, patients like that do not come- if there are some like that, then he is given a Nemolizer, etc, which respiration…it’s there no? Someone with respiratory distress, that’s what you said no? [*interviewer nodding]* Yes, so we give him Nemolizer, etc., Decadone, Dailyphyline, all this is given. So breathing difficulties are improved a little.

I: Are antibiotics common here?

R: I don’t know [*loud noise in the background makes it hard to hear]*

I: I see. Okay. In this do you ever…if a patient comes who has an upper RTI, do you ever do a sputum test or stool routine culture- do you do it first? Before giving medicine?

R: *[long pause]*…No the medicine starts, and after that, in the morning all the tests are done- it will only happen in the morning [*laughing; she is talking about IPD patients here]*. First the patient admission happens, the medicines are started, after that all this…tests, tests happen.

I: Mhm mhm. What do you think, meaning, which which illnesses are antibiotics given? Over here? *Pauses—*what kind of illnesses?

R: *Long pause.*

I: Please tell us an example, if something- some disease you know about-

R: Antibiotics- the doctors see the level of infection, it is more…spread in this [person], then we give antibiotics.

I: *[interrupting]* Can you tell us some examples of diseases? The diseases that you give antibiotics for…

R: *[pausing]* See, there is typhoid, etc- you have to give [antibiotics] with that. Or if there is a viral infection-

I: Say something more- something you see in the OPD or if you know something-

R: *[long pause]* Hmm…if there is a deep cut, and if that has also gotten worse, then also we give antibiotic, so in order for the wound to get better…like that…

I: I see…in this, does the patient ever directly say “give me an antibiotic”?

R: No nothing like that-

I: Never?

R: No.

I: *[pausing]* How frequently are antibiotics given? Like I had asked you before, that if 10 patients are coming, approximately how many of them would you give antibiotics to?

R: I give it as per the doctor’s prescription as he understands the patient- just like that. I give it to someone who needs it. I cannot say otherwise-

I: What do you think? Like, you must know an average, right? Sometimes you must be in the OPD, right? So in your estimation, how many take antibiotics, or for every 10 patients, how many does the doctor prescribe antibiotics to? Just an average like-?

R: 10 patients meaning, the doctor prescribes antibiotics to at least 6-7 of them.

I: Mhm he writes for 6-7 of them. I see. And when- when you are thinking whether you should give an antibiotic or not, what all factors should you consider?

R: *[long pause]* whether to give antibiotics or not?

I: Yes yes. Meaning you are deciding- what all things should you think about…

R: *[pausing, looking unsure, shaking her head]*

I: No? *[smiling reassuringly]* okay. SO, uh….hmm…what other things, meaning, are there some things that the doctor definitely considers before giving [antibiotics]?

R: Mhm [yes].

I: Like what things?

R: I cannot tell you; only the doctor knows. *[nervous laughter]*

I: OK, does it ever happen that you give antibiotics?

R: No, the nurse does not give.

I: And if the doctor doesn’t come, then? And if the patient needs it, then?

R: No, the nurse only acts based on the doctor’s prescription. The nurse never writes her own prescription. Nurses do not have that right. Following the doctor’s advice is the nurse’s…this.

I: And if the doctor does not come one day, then what happens?

R: Uh…usually, we give out something or the other- not antibiotics, but to bring the patient under control, all this…little bit we do, but then we wait for the doctor only. Until the doctor advises/prescribes, we do not act.

I: So antibiotics- anything- meaning you have your Amoxicillin, anything- have you ever given any such antibiotic?

R: No no, I have never given out of my own accord. Only with the doctor’s prescription –no, no, no. *[sniffling nose]*

I: So if a patient comes, and asks you, you meaning the doctor, for an antibiotic, and you think it is not necessary, so in this situation, how, what would you do?

R: I, that…I would say whatever the doctor thinks, he thinks there isn’t a reason, that is why he did not give. There is no reason.

I: I see, I see.

R: It is not required. That is why the doctor did not give it. This is how I would explain to the patient.

I: Okay, okay- and does the patient ever say something like that?

R: *[quickly]* No.

I: Does the patient ever demand? Wants an antibiotic?

R: No. No, no, no. No they do not say like that.

I: Assume…suppose that someone does demand. He comes and he says, nurse, give me Amoxicillin. So in that case, what do you do? You or the doctor…what do you do? The medicine?

R: Whatever the doctor thinks is right, he will do. The doctor will not proceed according to the patient’s advice right? Like what he wants, and doesn’t want. Yes, whatever the doctor thinks he should give or should not give-

I: I see. By doing an examination, whatever the decision the doctor makes, that is what is given.

R: That is what is given.

I: Okay, you were previously saying which antibiotics are available over here. So you said Amoxicillin- what else is available here?

R: *[pauses and brings out a chart of all available medicines from underneath the desk]*

I: Okay wow- *[everybody laughing]* There is a lot. Okay- there is Metronidazole, Ciprofloxacin, Norfloxacin, Ofloxacin, Septron, Amoxicillin, Linazolid- okay thank you. These drugs are always available here? It doesn’t happen that the drugs are not available?

R: Yes this sometimes happens.

I: It sometimes happens?

R: It is out of stock.

I: So then?

R: Then we write and order.

I: So if you have to give someone an antibiotic right at that time, then what do you do?

R: We prescribe from outside if it is not there. But it’s not like that. Something or the other is always there.

I: I see.

R: All of it doesn’t finish at once. And whatever gets finished, we write it down and request it from the storekeeper.

I: I see. Where do you call it from?

R: From the office.

I: From the office?

R: Hmm. We get it from the office.

I: Okay tell me one thing- the drugs that are nearing expiration. What is the process for them? What happens to them?

R: We throw them away.

I: Where? And when do you throw them? After they expire or- ?

R: We throw them after….they expire only. We put it in the chimney.

I: Suppose it is set to expire in January. Meaning it is about to expire on January 18. So…when do you throw that?

R: *[long pause]* we throw it before only.

I: Meaning- how many days prior?

R: *[pausing]* The date gets over, and the medicine is over.

I: I see and you will use it till then?

R: Yes.

I: How often do you check that- what is the status of the-?

R: *[interrupting]* we always check. Whenever we give medicine to the patient, we only give after checking the expiration date.

I: Suppose a patient comes to you on the 25^th^. The doctor prescribes an antibiotic, okay? That is an antibiotic- suppose it is Amoxicillin. The expiry date is written as January 31^st^. So you- will you give that to the patient?

*[pause]*

They come to you on the 25^th^. The doctor, he….prescribed the medicine. So you- the expiry date is on January 31^st^. That medicine- will you give it?

R: We can give it, right? [*asking us*] Because till now, the expiry date is far away [*laughing*].

I: Okay.

R: We shouldn’t give?

I: No no. We are asking you haha-

R: We give it.

I: You give it? I see, okay.

R: When it crosses that date, then we do not give anymore.

I: And what would the course be like for that? Like if we said, you are giving it on the 25^th^, and it is expiring on the 31^st^, how many days would the course be for? For Amoxciillin?

R: If it is a 5 day course, then we cannot give, because it would reach- it would reach the expiry date.

I: I see. If – If?

R: So that would not be given.

I: I see. If it will finish by that date, then you will give?

R: Yes.

I: Okay. And suppose you find out that a lot of patients are coming on the 25^th^, okay? And it is also the situation that you have to give antibiotics. But a lot of your drugs are expiring on the 31^st^. So what is normally done? If you have a lot of Amoxicillin in stock, and all of it is expiring on the 31^st^…so typically what will be done? [*pause*] If you are thinking patients are coming, and there is an illness, but you don’t know whether you should give antibiotics or not, and then a large part of the stock is also expiring, so what would be done in this situation?

R: We would not give. We would not do anything wrong. *[very confidently, without hesitation]*.

I: I see. I see.

R: Yes, we would not do anything wrong. If it’s expiring, it’s expiring.

I: If it’s expiring, it’s expiring. I see. [*pause]* Okay, while giving antibiotics, there are risks and there are benefits, right? The patient has benefits, and there is a risk also. How do you calculate- how do you think about this? What the benefits are, what the risks are for each patient?

*[silence]*

You know the side effects? What are your thoughts about that? There is good also, and there are also some side effects for some medicines. So what is your opinion about this? Please tell us.

R: *[pondering]* I cannot say anything.

I: You cannot say anything? Okay, fine. Anything you else would like to say, in terms of the doctor’s observation? Like you must notice what the doctor is doing, how is he doing this. How things work here- would you like to say anything related to antibiotics?

R: *[sniffling nose*] no it’s all okay, our doctors think before prescribing medicines. Everything is okay hahah *[chuckling]*.

I: What are your opinions about the patient? Suppose you give antibiotics, or whatever, for 5 days. Or like you explained that you have to eat this 3 times in a day (morning, afternoon, and night). And the patients…what? Do they eat the full course or…some, some people eat half the course and go away or do some people eat for 2-3 doses and then leave it? What, what is your opinion, about this, about overall patient behaviour?

R: *[interrupting*] Now see, they take medicine from here and go away. Now if they go home and use it or not, that we cannot say.

I: But some patients must be coming back to you too, right? Like I was saying, as we were discussing earlier, 3…

R: [*interrupting*] Yes, sometimes people come and say *didi we ate all the medicines but we are still not better*, and they say like that also.

I: But this must also happen- you gave for 5 days, and they ate for 2 days, got better, left it…?

R: Yes, they say like that also.

I: They say like that also? I see. They do not complete the full course?

R: Yes. Then, I scold them. You are going above the doctors. If the doctor told you 5 days, why did you not eat it for 5 days? You should eat it for 5 days only- if it’s 3 days, you should eat for 3 days. You…the bacteria for that, they happen like that then when getting finished- *[mumbling]*

I: So here what does the patient say? When you-?

R: He doesn’t say anything.

I: So do mostly children come here, or adults?

R: Children, elderly, everyone comes.

I: Children, elderly, everyone comes? The children that come….suppose someone has 2 kids, so do you ever think that if one child is sick, he takes the medicine, then when the other kid gets sick, he gives him the same medicine too?

R: Yes.

I: Does this happen- here?

R: It happens here.

I: It happens here? Meaning, he shares the medicine with the other child too? *[respondent nodding]* I see. How many do you see like this?

R: There are some- they take medicine for the elder child for a fever, and if this one also gets a fever, then they give it to him also.

I: I see. So, the antibiotics etc. that they prescribe, do they give like that also? Paracetemol, suppose it’s a syrup, they just give like that. Antibiotics also do they give like that?

R: That I do not know. I don’t know if they give or not-

I: You don’t know about that, I see.

R: I do not know.

I: What do you think- why don’t the patients complete the course? Among the people who do not complete? Is there a reason for that?

R: No, no I cannot say *[no hesitation*].

I: I see…has the patient ever said anything?

R: No, no [*quick answers*]

I: Okay- earlier you had mentioned about antibiotic resistance, what had become resistant. What do you know about this? What would you be able to say about this?

R: *[long pause; looks shy and unsure]*

I: If you know something, then please tell us.

R: No, no I don’t know.

I: Okay- have you seen that a patient is coming- on whom the medicine is not working.

R: Hmm [yes].

I: How many times must this happen?

R: I have heard about this. That they ate the antibiotic, the one that was given, but nothing happened. I have heard like this too.

I: What do you think- what reason must there be for that not working?

R: *[quick response]* They must not have eaten it properly.

I: Meaning they ate half of it and then left it?

R: Mhm, mhm, mhm [yes, yes, yes].

I: I see. Any other reason?

R: Anything else – I don’t know.

I: Okay, okay ji…how often do you see like this? Do you see it always or sometimes?

R: Sometimes.

I: Sometimes you see? Suppose you give for 5 days, and it doesn’t work.

R: Mhm mhm.

I: So this thing about antibiotic resistance, what do you think we can do to stop- what can be done?

*[pause]*

What can the government do, what can we do?

R: *[pause; looking unsure]* That I cannot say.

I: Okay. Have you ever heard of any guideline? Any guideline from the government- how antibiotics should be used, etc. How they should be used?

R: *[shaking head]*

I: No. There’s nothing here about that -?

R: Mhm.

I: Okay. *[pause]* Okay. The medicine that the doctor gives- what is your role in this generally? The medicine that the doctor gave- how do you, meaning at what level do you get involved?

R: As the doctor prescribes medicine for the patient, as he orders it, the nurse administers it to the patient [referring to the inpatient system]. Gives it from time to time.

I: Mhm mhm mhm. The patients over here- do they all come to the doctor or do they go to the uh- like, quacks, as they say?

R: Yeah they go to them as well over here.

I: They go to them as well?

R: Mhm [yes].

I: Okay, so the patients that come here, do they come after having had some antibiotics, etc., or they come just like that?

R: Just like that.

I: Meaning without eating them or after eating them?

R: Hmm…he doesn’t come after eating, he doesn’t tell us that he ate medicine from an external doctor before coming here. The patient just comes here and tell us their problems, and show the doctor.

I: I see, but, you know that they go outside as well?

R: Mhm [yes]…*[pauses]*…I will go upstairs now.

I: What you had said earlier- that if there isn’t medicine here, then you will prescribe from outside- if there, if there is no test available here, then you will get from outside. So, does the patient do that test? Or do they come back after that?

R: Yes they do it.

I: Do they have the money to get it done or to get the medicines?

R: Yes [*confidently*] some are there who get it done, some do not get it done.

I: Don’t get it done because of money?

R: Yes, they do not get it done because of money.

I: Don’t get it done- I see…[*pause*]

R: [*whispering*] I will go upstairs- I have my duty, no?

I: Sure, okay, okay. You have to go now? Thank you, thank you very much!

R: No problem at all [*smiling*]

I: Thank you, ji.
