# Supplementary material for: “Without antibiotics, I cannot treat”: A qualitative study of antibiotic use in Paschim Bardhaman district of West Bengal, India
Source: PLoS One. 2019 Jun 27;14(6):e0219002. doi: 10.1371/journal.pone.0219002 (PMC6597109; doi:10.1371/journal.pone.0219002)
Supplement: S2 File — (ZIP) [file pone.0219002.s002.zip › S2_Transcripts/KAP 207.doc]

**KAP 207**

Gender: Male

Age: 51

Occupation: allopathic doctor

Highest education: MBBS

I: So what does your typical OPD setting look like, meaning how many patients come? In one day?

R: 70 to 80 person. Minimum.

I: Per day?

R: Per day.

I: Okay, and how- uh how many hours do you spend during the OPD time?

R: 3 hour maximum.

I: 3 hours. OK, OK, so given 70-80 patients, what is the time per consultation? How much time per patient?

R: 3-4 minutes.

I: 3-4 minutes, OK. And are you typically the only doctor here? [*nodding*] Yeah okay okay. So how would you describe your prescription patterns, meaning what are the most common illnesses you see?

R: In this area, we see mostly…chest infection, cough and cold, skin infection like scabies, and uh G- worm infection, that you know worm infection- Albendazole we give.

I: Albendazole- yes yes yes. Deworming?

R: Yes yes, we for deworming.

I: OK so for cough and cold for example…

R: [*interrupting*] Cough and cold, GI upset like gastric upset, what we call [a type of gas- inaudible]. PU syndrome you can say in English, PU syndrome- Peptic Ulcer syndrome- the folks who have gastric-gastritis. For skin infections, there is like scabies, there is tinea, and this…especially like…I just saw psoriasis. I’ve seen psoriasis and refer the case to skin specialist. These are the chronic things, na, so won’t get better in one day, this has a long treatment. This is why we send to the specialist for this.

I: OK OK, so skin conditions you typically refer?

R: Yes we refer. Whoever can be treated here, I do it here, like scabies can be done here, tinea infection can be done here, what we call ringworm, all that can be done here.

I: So what do you do for scabies normally- over here?

R: There is…BB lotion for this. Benzyl…peroxide or something lotion is there. We call it BB lotion for short- do take a look. Okay? I give that for scabies, and also…for tinea, there is an ointment of Clotrimazole available, there is anti-fungal Flucanozole- I give all that.

I: Mhm mhm- are antibiotics typically used here? For skin infections.

R: In skin infections…antibiotics…within skin, you have for tinea infection. In the skin, the…the…ringworm that you have, it is called ringworm. Antibiotic is used for that. You get Flucanozole.

I: Okay, but if you have rashes, normally is a topical antibiotic used?

R: No it is not used for rashes. For that, we prescribe an Aloe vera cream or something, and if the skin is…dry, then I give a cream to keep it moist. Antibiotic is commonly not used in that case.

I: And for cough/cold normally…uh?

R: [*interrupting*] For cough cold, like a patient just came to me right now, I have seen this thing. I give him a normal…first of all, for chest uh…[hesitating] for cough, I gave a Paracetemol type of thing. I prescribed a cough syrup or advised it. If he doesn’t get better and says he still has a cough, then I prescribe antibiotic.

I: OK. In that case, what antibiotic would you give?

R: Given…uh…commonly, we try to prescribe the antibiotics which are available here. Like Amoxicillin…we get here. We get Azithromycin here. Cotrimoxazole you get over here. That’s why we see- and we get Doxycycline. I see what kind of patient it is. If I’m thinking…that he’s going toward TB, then we have to think whether we should give Cotrimoxazole or Amoxicillin. We do not give Azithromycin to a TB patient very quickly.

I: Hmm hmm hmm- why so?

R: Because…it does…it produces resistance. So we first think that, first, we should give a simple antibiotic, and if he doesn’t get better, then we give him- what would we give?

I: So you give first line first?

R: First line, yes. After that, cough, sputum- we would do a sputum test. He comes for a sputum test, someone comes from Raniganj.

I: Is this a private person or- ?

R: No, he is from the government only.

I: Government only, I see.

R: After that, if something shows up in that, then we…give it, otherwise he would get better from common anti- he’ll get better.

I: Do you normally get tests done before giving first line antibiotics or not?

R: No, normally, we do not think about that. Not possible. It isn’t possible to do also. Practically, not possible. Otherwise, you would think, no give it by calling someone- we simply first give you simple treatment, like I give you Paracetemol if you are getting a fever, gave you Salbutamol for a cough, gave you a cough syrup this- this Levocetirizine, this which is combined with…um, what do you say, Salbutamol plus Ambroxil, all of that is mixed we give, the syrup one time. After that, I say to gargle, and if he doesn’t get better even then, then I give antibiotics. And in that antibiotic, Amoxicillin is first. Amoxicillin, Ampicillin, Cloxacillin.

I: What was second?

R: Amoxicillin is a separate one. Called by like Mox.

I: Of course, of course, Amoxicillin.

R: Yes. Then Amex- Ampicillin-Cloxacillin. Both come combined. If it doesn’t get better after that also, then we go for Azithromycin. After all this, there will be a test. Give a course, if it doesn’t work, then do a test. When the result comes negative, then we go for Azithromycin.

I: So normally, when you say the patient did not get better, does the patient come back to you?

R: Yes he comes. He comes. Over here, we only give 3 days worth of medicines. Basically, it is outdoor. We prescribe medicines for three days only.

I: OK not full course?

R: Not full course. On purpose. Because they will waste the medicine, na? They are also local patient. People come to me again after next visit. After eating for two days, they think again they will come back. Then he will come and show me. If I gave him 7 days of medicine, then it’s not necessary that he will eat it all. I am giving medicine free-of-cost, but it also has some cost, na? Then also, I…in the beginning, I used to prescribe for 5 days, 5-7 days. Someone gets better, they stopped eating after 3 days. [**5:20** Bengali] Then the sister is saying that “sir you are prescribing for 7days, our stock is finishing but the other patients are suffering.” The other patient did not get it, right? You won’t get another one from your stock.

I: Yes, so you were saying…suppose a patient comes with a fever, and there is no other symptom. So what would you normally do for this?

R: Normally, if he’s an adult- an adult, I will first give him a Paracetemol, I will tell him to do cold sponging. Okay what are the other symptoms? Mostly in a fever, it can be a viral fever. So in viral, there is no role for an antibiotic. Okay? But even then, we sometimes give an antibiotic even in a viral. Ask me why. Because I had also asked your junior this answer, but she doesn’t know, but let me explain to you- because in a viral infection, the antibodies are prepared in the body. But the viral is damaging your tissues, somewhere or the other. After it damages you, there is a secondary infection. When there is a viral infection…suppose someone gets chicken pox. It is a viral fever. It is a viral infection. But why do we give antibiotics for it? To reduce the severity of the infection. So when the viral damages your tissues, then that…when it damages the tissue, it will become dead tissue. After dead tissue, there can be a secondary infection. There can be a bacterial infection. So to combat this secondary infection, we add antibiotic.

I: Like a prophylaxis almost to prevent?

R: Prophylaxis, prophylaxis.

I: OK OK. So how do you know it’s going to develop? Do you always take, uh, do you never take the chance? You always give antibiotic?

R: No, not always. Most of the time.

I: I see. So like, which viral illnesses do you give it for, which one do you not give it for? Like, how do you decide when to give and when not to give?

R: In viral fever, there is a high-grade fever, okay? That uh…there may be a few rashes type things on his skin. There are symptoms of rashes. Cold cough, meaning cough, eyes red from the cold, high grade fever, so this can be viral. And if someone else has gotten it in the home, then it can be diagnosed from that too, that there was a cross-infection in some houses. And if it looks like only he has gotten it- please stop this [recording] for a second [he is busy with patients].

As you can see, I am working and I work very fast. Because I have some chamber in my house, I also have a chamber [private practice]. So people come here and think this is all there is. If I did this much work in my chamber, I would get 250 rupees. Per patient, okay? But my- everyone has their own nature of working. There are 10 patients coming, and you are seeing them all day. 30 minutes for each. I see patients very quickly. You are seeing how many patients came and went, came and went. I talk to them, got the diagnosis, and prescribed the treatment. Now the BP machine here does not work- I said, I could have made an excuse saying my BP machine is not working, I won’t see. But no even then I said no I’ll get the BP machine from my chamber and see him right away. Someone is going to do an operation- he’ll stop for some reason, did not check the BP. So I saw his. They are not understanding the importance of this. He is seeing the report, and it is normal. Even then, he is asking: you don’t have a sugar problem do you? Now see how much time he is taking! Meaning this matter, the problem in the government system is that people do not understand the importance of it [referring to the importance of the doctor’s time]. This- if today, I was sitting in Anandalok or mission hospital then people will say we went to mission’s doctors or anadalok’s doctors[8:31-8:37]. I would give the same details. There would be more fame. But here they [patients] say we went to government doctor, didn’t understand what they [govt. doctors] have prescribed. [8:39-8:43]. This is the mentality, okay?

I: So how confident are you in your diagnosis? If you assume you only have 2 minutes, then how confident do you feel?

R: See, meaning basically the patients who are coming to me, however history taking is very important. When you are talking to me, go right then and ask what the problem is. Sometimes patients came and complaining of vertigo but he was simply roaming in the market [9:00-9:05] So meaning test his BP, and we find out how much truth he is lying or telling the truth. And you will see any illness that is present too. That he is actually in pain. Like someone just came, had a small wound over here, and his wound increased this much [incoherent stuttering] so then I prescribed a sugar test. With high blood sugar, the wound does not heal. I prescribed the best antibiotic available to us over here, Amoxicillin-Clavulonic Acid. And I even gave Ciprofloxacin with that. He is an old age patient, and if there is blood sugar also, then it will take care of him until he gets the test. There is no room to wait and watch in this case. That now you test again, then prescribe- [patient interrupts]

I: Yes, so you were saying that there is no room to wait in this case.

R: Yes there is no room to wait.

I: So how many days was that course for?

R: I gave him a 3 day course only.

I: 3 day course? Everyone gets a 3 day course?

R: 3 day course. Then he will come to me, and I will understand about the recovery. If it’s looking like it’s going well, I will continue it. If I think the response isn’t right, sugar and things are like this, then I will change the antibiotic accordingly.

I: So by your estimate, out of 10 patients, how many come back to you for follow up?

R: They come back over here. This is a local area, so 60-70% of patients come back, especially in this area.

I: Earlier you were talking about illnesses. The illnesses you were talking about earlier- so in diarrheal illness, what do you normally give? Loose motion…

R: Simple diarrhea happens because of food poisoning, which is a water-borne disease in and around this area. First, see, I always write “use boiled water.” Totally. I give ORS. #2. Third thing, if he thinks that the problem is diarrhea meaning going to latrine 3-5 times, then I add Metronidazole. Without asking any other thing. So that is an anaerobic infection…I give Metronidazole. Metronidazole, ORS, boiled water. And I give advice that you boil water before you drink, cover the water, eat smashed rice, etc.

I: So normally if 10 patients come with diarrhea, how many of them do you give Metronidazole to?

R: [*long pause*] Most of them.

I: Most of them, okay. One…uh, antibiotic- why is antibiotic needed in this?

R: See, the difference between viral diarrhea and bacterial diarrhea is that viral diarrhea has more severity. The stool comes out like water, especially in children. Okay? Doesn’t happen in the aged. So here, the maximum patients who come here, 70-80%, are adult patients. The kids that come, they also come with diarrhea, so I give Metronidazole to them also. Why- see I am telling you- if you give medicine based on diagnosis, na? Then things will get worse. If you think practically…theoretically, you wrote a lot of books, bound them, I am a Gold medallist, enough…today I’ll give you an example about a doctor. Suppose a patient- if a lady delivers a baby. You prescribed a medicine and it’s not getting better. I prescribed Reglan, the name you’ve heard, it’s a medicine for vomiting. Metoclopramide- it’s composition is metoclopramide. I prescribed it for someone- and he went to the shop. And the chemist shop asked- what happened to you? Are you vomiting? Then patient say no my wife has never been vomited. Then say don’t know what medicine the doctor has prescribed to me.[12:00-12:05] You know Metronidazole- uhh Metoclopramide, what is known as Reglan, the Reglan tablet which comes…the way it works is that it increases prolactin which also increases milk secretion. So what I did is- art right? The medicine is Metoclopramide only, what you call Reglan. What did it work for? It’s not just working to reduce vomiting. Side by side, for breastfeeing, it is also working to produce more milk. So this you have to know, how much you know and how much you prescribe. The shop keeper influence him [the patient] is your wife vomiting? No. She is not vomiting. He [patient] didn’t buy the medicine. Second day he came and complaining my wife’s breastfeeding didn’t improve but you wrote her medicine for vomiting. I asked who told you that. The shop keeper told me. [12:30-12:40] Then I called him and I scolded him- do you know why I prescribed it? He says for vomiting and I said it is not a medicine for vomiting! He says we give it for vomiting. It’s used for other things also. So medicines have many different uses. So here I gave an example- that medicine practice is a art. How you prescribe- shut this down- there is a patient coming.

I: [Resuming recording] Yeah so earlier you had mentioned combination drugs, so here what do you prefer- monotherapy or combination drug?

R: What kind of problem is there? What kind of problem?

I: If, suppose, you have to give an antibiotic, would you prefer monotherapy or combination?

R: Simple pr- single therapy- meaning I try to give single antibiotic.

I: Why?

R: Because antibiotic- if someone has a bacterial infection, so…you don’t know if someone is resistant or anything like that. But in both ways, if both become resistant, then you suffer a loss, no? That’s why you should give single first. Mono-monotherapy. When it doesn’t work, then we give combined.

I: Mhm mhm- and what is the availability of combination drugs in the market right now?

R: Now it has increased a lot.

I: Increased a lot.

R: Cefaxim, Ornidazole, like this, O2. Its name…Ornidazole and uh…it has Ornidazole and…I prescribe O2 from the bazaar. Here, it’s single therapy mostly. We mix very rarely. Like we give Ciprofloxacin with Metronidazole. You get a lot in the bazaar. You get AmoxiClav- you get Clavulonic acid and Amoxicillin mixed together. Along with that- now, you get Oratil LZ- Cefuroxime and Linezolid. Okay and then if you write from Cipla then you have maybe Cefuroxime plus Ornidazole.

I: OK OK. In this, do you prefer generic or brand name drugs?

R: See…generic should be written by people- definitely. But all things will not be taken care of by generic.

I: Like what won’t happen? Can you give an example? What things do you find difficult?

R: Combined antibiotic will pose a problem. Now if in generic, you prescribe a hepatitis…if you prescribe something for hunger, a lot of things are combined in that. There is Lysine, B complex, some other enzymes are also mixed in- how will you prescribe it? Everything…it is possible, but it is not 100% possible. Suppose someone has- some- has male infertility, you can give him such a medicine that, many are Ayurvedic and many are mixed with Allopathic too. So how will you prescribe it? Lycopine is mixed in that, Cocufort [15:22] is mixed in, so how will you prescribe it? Now the government is telling you that you have to prescribe generic- whatever happens to the medicine manufacturers, our situation will worsen- we won’t be able to prescribe! Now some people say that you only prescribe generic over here [15:30]- so people are educated over there, right? So you can explain it to them, but you people won’t understand over here. Oh this…what kind of prescription is this, brother? I will write four medicine names and I will write this…do you know Leeprufid syrup comes. That is a medicine- it is a liver enzyme. There’s a lot of things mixed in that, Sorbuline, etc. So how will you prescribe it? If it’s antibiotic, you can prescribe, but like this in generic, you can’t prescribe.

I: Okay so if it’s generic, you have to prescribe many medicines?

R: You have to prescribe many medicines.

I: Okay. And then the patient…meaning, doesn’t take?

R: How will he take? If you write 4 medicines, he will say ‘what medicine did you prescribe?’ Think about yourself- suppose you have gone to a doctor and he has prescribed B1, B2, B6, Lysine is mixed in, and take a syrup too- will you be able to take all that? It’s there some places, but it’s not in the general market.

I: Mhm mhm. And this, broad spectrum antibiotic…that you know work for many illnesses…which is not specific for a particular illness? How is this used?

R: See in Hindi, we call this ‘gerao therapy’ [therapy to cover everything]. Gerav therapy- meaning what? You have put everything. Aminogylside—you’ve put Gentamicin also. Monotex SB Applied, Ceftriaxone and sulbactum applied, and you’ve put Metronidazole also. Suppose we do some OT…like doing an operation, no? Operation like you operate on the stomach or do anything…so in that, we don’t give just one antibiotic. You maybe…I don’t know if you know or not. [Phone ringing and a patient come in].

[“Doctor to patient: how is your stool? Patient: I only go once in the morning for latrine. Doctor: Anything else? Patient: No. If sometimes stomach pain occurred then I go again to latrine.”]

See this person – I gave her B complex, ORS, and she has gas and stomach burning so I gave her omeprazole. Belladonna is not working for her. [17:25-17:29]. There is no role for antibiotic here. [Doctor to patient: I am prescribing one syrup to increase hunger. Buy from outside. Patient: Give me two medicines for allergy”. Doctor: What happened? Patient: Sometimes it’s swelling. Doctor: Nothing happened go now.] **[Bengali until 17:50]**

[A new patient comes in] I gave Rantac…

I: You give deworming to everyone? The dosage for deworming?

R: 400. 400.

I: OK it’s written on that?

R: Yes. Repeat after 15 days. Go on, go on [ushering patients out quickly] If I don’t see patients quickly like this, then I’ll be here all day.

[Seeing another patient] In oral- oral infection is anaerobic, okay? So I gave her this…diagnosis. No one must be doing it so quickly. So I have worked in a Marwari school. In one day, I used to see over 200 patients. I used to also do OT and other things along with that, okay? I gave Brufen and Rentac [referring to the current patient]- why did I give Rentac? Because she is having gas, okay? [“Doctor to patient: Do gurgle with luke warm water and take the medicines. If it is not cured after that then come to the dental OPD. Go to Raniganj in Monday, Wednesday or Friday.”] [18:34-18:48 in Bengali]

I don’t know many 250-rupee patients I’m losing right now [laughter ensues as the doctor laments how many private practice patients he is losing by being here in the PHC].

[18:50 to 19:10 in Bengali as he examines a patient]. [“Doctor to patient: What happened cough? Did you take alcohol? Open your mouth, stretch your mouth” OK now I will give him Paracetemol. “Cough and what? Patient: Can’t eat anything. Doctor: What do you eat? Do the gurgle and don’t take alcohol. Okay. Go”] [19:15-19:35 in Bengali]

I: yes so you were telling me about broad spectrum antibiotics.

R: See broad spectrum antibiotics are used for a lot of things, like I’ll give you an example. Like I told you, broad spectrum means gerao therapy, meaning giving all kinds of antibiotics. If I did a stomach operation on someone, then I give him Septazol-Sulbactum…gram positive, gram negative bacteria…for gram negative, I especially give Gentamicin. And I gave Metronidazole for anaerobic. I can’t let him get any kind of infection. This is called gerao therapy. And broad spectrum antibiotics are those that work in many different areas. Now as an example- most antibiotics are broad spectrum antibiotics. Most of them. What used to happen before…first they used to give an antibiotic which has a specific purpose, but now have you heard that a patient got pneumonia and he died from pneumonia? No. Why? If there are some symptoms, patients will go and eat medicines on their own. So any antibiotic, in whatever way, will work on him. It will work somehow on him. That’s why nowadays…first it was lower pneumonia, and he- he died from an infection…that doesn’t happen anymore. Nowadays, people eat antibiotics on their own also, and whatever antibiotic they eat, some or the other will work. Very few people are such that they are in pain and he is not eating anything, okay? That’s why nowadays…whenever a patient comes…so you assume that he has taken 2-4 medicines before coming to see you. For sure.

I: But how do you know whether he has eaten or not? Do you ask or…

R: Yes I mean he definitely takes. Even if you went, you will say- I will have to pay the fees to the doctor, better I try to see if I can eat 1 or 2 on my own first and then I will see and maybe go. Practically, this is what happens. People have to be practical.

I: So what is the risk in this, with this uh…use of broad spectrum antibiotics?

R: Yes, antibiotics when they are…misused you know, [doctor is interrupted by his support staff]

[21:15-21:30 in Bengali].[“Support staff to doctor: Patient has completed the course of Azithral. We don’t have Cefotiam (CTM). Can you give me something alternative? Doctor: I don’t give him Azithral. Support staff: We don’t have CTM. Doctor: Then don’t need to give anything. You can give amoxicillin but tell him to do sputum test if not cured.”]

I: You were telling me about the risk.

R: I was telling you about the risk. Whenever a bacteria will take the antibiotic, any person, his chances of resistance will keep increasing. You are consuming antibiotics for no reason, so the bacteria mutates and changes. Changes and develops antibiotic resistance. Whatever antibiotics we have at present- whichever ones are available in the market- few are being researched, and more are being misused, so resistance is naturally increasing. The antibiotics available to you are decreasing. So what happens with resistance? If we do not do public awareness…someone is eating antibiotics on their own- eating for 5 days, or for 2 days and then leaving it…so there will definitely be changes in the bacteria. And the next time, that antibiotic will be useless for him. This is it- this is the main drawback.

Now see this patient. She says she has a fever, so I gave her Paracetemol. I will have to give antibiotic along with that, okay? And she…[long pause]…[asking the father of a child patient who is complaining of headaches whether she has done wet sponging on forehead. Tells her that both medicines are available here in the Government pharmacy and that she should try and take it. If it doesn’t get better, then she should get her eyes checked. Because sometimes, even poor eyesight can cause headaches. Now go and they will tell you further].

[22:55 to 23:40] [Patient comes in][Doctor to patient: What happened? Patient: At night it’s paining [Showing some part of his body]. No problem at all in the morning. Doctor: At night it’s paining. What do you think? [Pointing toward the interviewer]. At night he suffers from tettni [used some local name of disease]. Shelcal hd. I gave him calcium. Do you have any other problem? Patient: No. Only chest and leg pain nothing else. Doctor: I have written paracetamol and medicine for gas. You have to by one medicine. Okay. Also gave him albendazole. The medicine [Shelcal hd] have to buy from outside and took once after lunch. Rest of the medicines will be explained by pharmacist. Patient: Which medicine is this? Doctor: It’s calcium. You go now.”]

I: OK, do you do any minor surgical procedures of any kind over here?

R: See, I don’t cut or do anything over here. The setup is lacking. I actually come here on deputation. There is no M.O. here- I was told to come for 6 months, and I have been doing it continuously for the past 2 years. Actually, my posting is in [location removed]. Have you been there ever? Try to go from Raniganj and see what it’s like. Now you know why I was late? The road there is bad, so the silencer in my car broke. And I was trying to get a new silencer. It cost me 2500-3000 rupees- that’s how bad the roads are. The government tells us that…I am very…that doctor come on time, do this, do that, but for that, they will have to see the other structures too, right? There’s the roads, the other factors like politics which have to be reduced. Now I’ll tell you- a patient dies and the patients say look the doctor did it, the doctor did it! Particularly, in our area, this is becoming more frequent.

I: Blaming the doctor?

R: Blaming him. Now, my friend—I was very busy yesterday—my friend’s father was doing something on the gas, etc. He was admitted to the ICU. There was nothing out of the ordinary- I also saw that all reports are normal. Everything normal, everything okay. After two days, he was told to go home. He came home two days ago, and he was brought in from the car in the afternoon, and then he was on the way to coming here from the home, and boom- he had a heart attack. Now if this had happened in the hospital, what will they say? The doctor killed him! Look, anyone can get a heart attack! Second example…I have a close friend- her kids stay in Bombay. His daughter had a son, so they went home to see them. They had to go for an operation at Jaslok Hospital, very famous hospital. There was a patient- he was walking around and talking just fine. He went home, and at 9 pm, he had a heart attack. So it won’t work by blaming us doctors, right? Anyone can get a heart attack. Third example- I have a friend living in Durgapur. He called yesterday that his friend’s brother had this happen to him, and- can you do this and this? I told him I’m here myself. What happened? All of a sudden, he got a heart attack. So this kind of blaming- the doctor killed him- no one will want that our profession looks bad. But the nature has become such that- politician folks are changing the public’s nature, especially people’s aggressiveness has increased towards doctors. But all doctors are not like this.

I: So after seeing this situation, what effect does this have on your prescription patterns? Meaning the prescriptions that you give.

R: Yes, so we start writing, na? That ‘refer to higher center, or refer to skin OPD’ or, or I refer to another department. I want- I think that if he gets better, then it is a very good thing. Then, tomorrow, he cannot say that you did not prescribe this for me.

I: And the medicine that you give – is there any difference in that? After seeing that the doctor gave this, or did not give that? Does the patient demand anything directly like antibiotics?

R: See what did she [referring to previous patient] just say? [inaudible] I understood there is nothing in the skin, so I didn’t give anything allergy-related. Normally, I give Levocetirizine, but I did not give it to her. Some people do demand, but you have to explain to them sometimes, or sometimes you have to prescribe.

I: And do people ever demand antibiotics by name?

R: No they never demand. Because they are not so literate, no? You’ve seen the patient- what kind of patients there are. You can see that they…are from a very…meaning poor class. They are innocent people. The people who motivate them are other people. Yesterday, a reporter was talking to me, saying that see brother, no one from the house would want someone to die, causing a commotion or something if someone dies. No one will want a doctor to kill them. But there are 2-4 mischievous people who make the matter worse. Hey look what the doctor did! They will bring 10 people and stand here. They create the chaos/disorder that doctor has killed someone. Why he killed the patient.[27:10-27:13]. Now you know professional people do not want to send their children to the medical line. This is a very problematic thing. We work to do some good…no one give a 100% guarantee for anything. Does a lawyer tell you that you will win in court? If he wins in lower court, he loses in higher court. So that means he should leave his job? Someone should sue him as well. Tha t doesn’t happen, so why blame the doctor huh? Here, 2 + 2 should always be four, which is not possible. 2 + 2 can be 5 and it can be 3 also. This is a big factor- understanding this…antibiotic is okay- it’s a good thing you’re doing something about antibiotics. But public awareness also needs to be increased that you shouldn’t always doubt the doctor. Now the problem is that the medicine in the corporate hospitals…whatever treatment I can do here…I am not doing that either. Why? Out of fear. When it gets worse, it will be seen. Getting worse means Vivekananda hospital, Mission hospital,and other medical colleges…[28:00-28:04]. If he goes there, what, will he get this much care? His money will also be spent. And he will face trouble as well. Now here- the local people from the party who practice here- I know we have to think very carefully before doing even a small operation, whether we should do or not. Why? If there’s a problem, then they’ll climb on my head [blame me for it]. And if something happens over there [corporate hospital], he won’t say anything. He will also spend money there, and then stay quiet as well. Go see in the Mission Hospital- people have to spend 50-100 lakhs, saying whatever the doctor says will be good. And they don’t know who is doing what inside. And here we talk to them face-to-face. The public will have to understand that the doctor does not make a mistake [deliberately]…anyone can make a mistake, but no one does it deliberately. To make the patient worse. Okay? So this antibiotic awareness is very good, but this is also a very big disease.

I: That public…?

R: Public awareness! That doctor is always not wrong.

I: Okay, so this antibiotic resistance that you’d mentioned earlier- what kinds of things increase antibiotic resistance?

R: See…antibiotic resistance could be if you are not taking the antibiotic properly. Second is if you are not taking the dose properly. You are not taking the proper duration. Dose and duration…is very important. Prescribed for 7 days- you ate it for 3 days. You’re told to eat it 2 times- 3 times a day, you are only eating it twice. These are the factors. Along with that uh, you are using it when it is not needed. Where you have no use. [*stuttering inaudibly*] They’re taking it down, eating antibiotics for 2 days, 3 days, eating it, getting better. And it may be that you did not even need it. All this.

I: I see, I see. So you were saying that you call them for 2 days and then you call again, meaning,

R: [*interrupting*] Over here.

I: Yes over here. So what effect does that happen on antibiotic resistance? If the patient is not taking the full course, so in this meaning, the practical thing I am understanding…but…what effect does this have on antibiotic resistance?

R: No, by calling him after 3 days, the benefit for us is that then we can have a follow-up. That how the patient’s response was. And how much his need is. If he’s really in pain, then he will definitely come. And if he’s just faking it, then he won’t come again. [*pauses*] Suppose that he is not having any real pain…then he won’t come? So we…you will have to do an assessment. Whether we should call him in 3 days or 7 days. We give 3 days medicine, and then tell them again to come back after 3 days and show us. He remembers that I have to go and show. The patient comes.

I: So what is the risk of resistance in this? If you have only given for 3 days, and then you are following again, so then some patients- the- they are not getting the rest of the course that is there. What is the risk in that?

R: [*pauses*] Incomplete treatment always has a risk. It will never have a benefit for the patient. It will benefit him now, and then it could cause him a loss later on. It can come back, then there is a loss- if he develops resistance, then the antibiotic will not work on him.

I: Okay okay. And suppose there are 10 patients coming to you, how many of them do you see with resistance? That the antibiotic, meaning, is not working? What percentage…do you see?

R: It’s hard to say exactly…

I: Approximately?

R: 3-4 patients must be coming, like whom we treated but did not get better.

I: In that then, what do you give if…?

R: Then I give him the market medicine. Whatever medicine is available here, I write as well at first. If that doesn’t work, then I prescribe from outside, which is more expensive.

I: In addition to first line, meaning…more expensive…

R: In terms of availability, the one outside is more expensive, and they get better from that.

I: Mhm mhm. Do some patients also come, who…meaning, are resistant to all medicines? Some patients?

R: In this OPD, such cases do not come.

I: They don’t come, okay.

R: They go somewhere else. It’s a small setup, right? If it’s a big setup, then they’re many kinds of folks that come. The people who come here are just these kinds- like I just saw a patient in front of you. Even while talking to you, I must have seen 30-40 patients. It’s going so fast…the question, you…can see the name.

I: Okay, to distribute antibiotics, a lot of people involved, such as MBBS doctors, RMPs, nurses, and also pharmacists. What is everyone’s role in giving antibiotics, meaning what do you think about the other…players, the other people?

R: See, the misuse of antibiotics has increased. Everyone gives antibiotics. Why? Because they think that if they give antibiotics, the patient will recover faster, right? The public demand is also there that “I had body temperature last night. Doctor: No you don’t have fever now. Patient: No no I had fever last night.” [31:55-32:03 in Bengali] now by giving him Paracetemol, we just force Paracetemol. You must have seen I write B-complex, I write BC. You must have seen. BC- Albendazole- Paracetemol. This is a common prescription of mine. Whatever…there won’t be any loss from B-complex. There won’t be a loss from Paracetemol. There won’t be any loss from gas medicine. Where antibiotics are involved, I need to think a little bit whether to give or not. And some people also come where I think first let me give this…and otherwise what can you do? If the pressure is normal, so anyway, you can’t do anything. So I give Paracetemol, some ORS, B complex, Rentac, Albendzole. He thought he ate some medicine. Psychological…and after that, when he comes again with some problem, then I’ll tell him you go get your glasses checked. Like this girl that came…she has a fever, but why does she need an antibiotic? There can also be a problem of glasses. I have seen many people like that. We should also work with common sense- it’s not necessary that in every case, medicine does the trick. You must have seen that I asked whether she goes to school or not. History taking.

I: The way you questioned, yes.

R: Yes, and then I asked to see if she may need glasses. When kids are playing around, it also happens. They’re watching TV, playing with mobiles nowadays. The problem is increasing. Here, antibiotic is not required. Do some counselling! Right?

I: of course, of course.

R: I don’t know you must be thinking what is the doctor saying, I don’t know.

I: So how is counselling of the patient done, meaning what do…doctors normally do to counsel?

R: See, all of this in the OPD…those kind of patients do not come as much. But in private practice, we have to talk about many different kinds of things, like patient’s…confidence, because doctor gives more time there. I get patients from Dhanbad, from Jamuria, from very far away. So they think that doctor Sir gives us more time. And by going to him…meaning…everyone gets treatment, but only by properly counselling the patient and treating will you keep the patient forever. Wherever he will stay, he will come and seek you. And if you do not counsel and write the medicine, he’ll say the doctor didn’t ask anything. He didn’t talk at all, just prescribed medicine, got his bill of 300 rupees. Now how to eat this medicine…sometimes, what happens- I operated on a patient, okay? And I was going away for a bit so I went. So the doctor went on vacation. Prescribed medicine. When he came to me, his scar had swollen up. Got scared, the poor guy, and said how did the wound get so big? When I told him to bring all his medicine, then? She was a layman, the lady. Ate all her medicine, except the antibiotic…[*pausing*] she had to suffer. Now she’s coming to do the dressing, and I’ll give her a secondary suture. She’ll get better and come again tomorrow for dressing. I am not taking a single paisa from her. I know the elderly lady [derogatory term used: *budiya*] is also at fault and my staff is also at fault. At least they should have said you had a operation, make sure you eat the medicine. The wound has to get better. Otherwise, if you don’t want to eat food, don’t eat. What did she do? She ate everything [rattling off incoherent medicine names], ate everything for pain, but did not eat the antibiotic. She suffered and the wound got worse.

I: Where did this happen? Oh, here? Private?

R: No no, in private! [*laughing*] Impossible that it would happen here. I am giving you an example of what kind of problem can happen if you don’t counsel people.

I: But how does all this counselling, meaning, why doesn’t this kind of counselling take place over here? Proper counselling of patient?

R: This is a basic primary health center, na? What happens in the primary health center is that…meaning, simple patient comes…simple means what? Cough, cold, diarrhea, vomiting- these are the patients who come. When you go a little above this, then you’ll get the subdivision hospital. No no, it would be the BPHC- Raniganj. If you go above that, it’ll be that. And above that will be that. In these kinds of centers, there isn’t as much of a need. I’m telling you if the government…the government…I’m not blaming…I’m also from the government job [*laughing*] wants to do things honestly, then honesty or charity begins from your home. You know that right? Charity begins from where? From the home. So honesty begins from…yourself. That I come on time, care for the patient, talk to him- if I go to my chamber, I come here too. But I told you what the patients here think of me, right? They think that doctor is just like that- he’s there, there’s no importance. So then I also think- you went to my other government place right? How nice and clean I’ve kept it! Because I’m in charge of the place! And here I’m on deputation, but even then there is coordination with all of them. So what needs to be done? The government, all of the basic things, that we also have staff…come properly, education, training, and the people like, I mean what happens? The medicine that you give here…suppose it is not being dispensed properly, then it will expire. You go and see over there, I didn’t show you- I go in my own car from the BPHC and keep the iron syrup over there, without any indent or anything but then it will expire over here. At least there I can give iron to the poor people. You must have seen many people have gotten it. You go to Raniganj and ask people. I keep Iron tablet and syrup myself, that at least the poor should get it, on whose name the medicine has come here. Over here, it will expire and we’ll throw it in the sand. You need this awareness. When you get this awareness, you will think that you’re doing something.

I: So what other things are there that the government is not providing, by which your work can be improved?

R: There is a huge staff requirement- the staff are very limited.

I: Staff? Everyone, meaning your nurses and doctors and all?

R: All, staff are very limited. Postings are not happening for many days. At all. And then a building was built over there. Lakhs were spent on it. What should the government do? If they had asked me, what would I have said? Make Raniganj BPHC a first class center, give first class service in that. So people do not even have to go. And put a lift there, and like a private, make it like a private center. And increased the staffing of doctors. Increase the staff. And they all should come on time. Like in private, they all work, right? Like that, they’ll work here also. You’re giving him lakhs, and your input is not happening, why? Even if the government is wanting it, maybe the local politician is not letting it happen. Some reason is there. Until today, you what- our area- what drawback does it have, tell me? I am from the area- should I tell you what the drawback is? Where are you from?

I: Bakhura. Bombay.

[38:25 onwards needs to be transcribed]

R: I see. I’ll tell you what the problem is. If you have to develop a hospital in this area, then Ranigaj Alugoria hospital should be developed. Building should be developed in this hospital. Government has made the political decision and make Raniganj as referral hospital. As Ballavpur was the area of CPM, they made a memorial in the name of Binay Choudhury in that corner where no one wants to go. Is this wastage of money or not? If you have to prepare then made it good. You can keep a small outdoor here [in reference of Ballavpur RH] and for big one make it in Raniganj. Till now the structure of Raniganj is same, a one storeyed building with 10 bedded hospital. How much load it would take? You should build Raniganj as it can take load. But you made a hospital in that corner [in reference of Ballavpur RH] due to political interest. People will give us vote as we have prepared a hospital over there.

I: Tell me one thing what are the measures are needed to take from your side as well as by govt. to prevent antibiotic resistance in National level as well as at local level by govt. and ?

R: Public awareness is needed to increase. Make them aware that only use antibiotic when it is needed. Don’t take antibiotic by yourself. Also education is needed for doctors and nurses that explain patient about the need of antibiotic when prescribed. If you think it is needed then give otherwise consult with your senior. These are very simple things but you have to do.

I: Does nurses give antibiotic in sub-centres or in these areas?

R: They don’t give that much. Give co-trimoxazole .

I: What is the training they get for this means what is needed to give or not to give?

R: See actually nurses are not authorised to give antibiotic. But our structure is like that. They have to do the work. If I don’t go to Tirat today then who will do the work there. The centre will be open. Don’t get me in trouble.

I: No absolutely not. The identity will not be disclosed in the interview. Also I will not identify any PHC.

R: In Tirat , there is no pharmacist only sister and me are working and another one group d staff. Only 3 staffs are working there. Another staff comes in daily basis for cleaning. There is minimum need of two sisters, two doctors and another pharmacist is needed then the work will go one. Slowly people will be interested that doctor is available, medicine is available. Also supply of medicine should be proper. Then we can treat properly. When we are giving health facilities then that should be better quality. It’s okay if you are not giving 100%. 100% is not possible to give by any government to dispense medicine. But as I know the qualities of medicines are given in the health centres by West Bengal government is better than any other states. I didn’t go to other state but it is better than the nearby states like Bihar and Jharkhand. Medicines are properly dispensed here also the staffs are very honest. They come and go on time. I come little late. You know that doctor has some bounding. I can be late sometimes. But I instructed them to call me if there is any need. Even if I am in any work trap I will come early. Otherwise I shuffle things around and get there. It is big thing to manage 2 places. It’s going on. In government list a doctor is posted in the centre.

I: In government list. Did they tell you someone is posted here?

R: Yes. Recently posted. But they are working in the other centre.

I: Why there is shortage of doctors? What are the barriers?

R: Barrier [Asking]

I: Yes.

R: Do you want to know why doctor is not coming?

I: Yes.

R: If any doctor comes after study they need some basic facility. They need room, road, electricity, safety. If they have kids then need education facility. If government give all the facility then no doctor will be disagree to do social work. We are doing duty and we want also our family will survive with us. If family staying in Kolkata and I am here then how do you concentrate in work. They will leave ultimately. Do you know why I am surviving here? Because my home is nearby in Raniganj. That’s why I can come here. That is not a problem that I am in deputation. I feel that I am working and also I have worked in many places so I have experience of how to work. If I want to waste time then I sit here from 9am to 2pm. I will see limited number of patient. But I think I have to give real service. See time is very important. For a doctor it is difficult to maintain 100% time. From government side other facilities should be available. Road, electricity, safety, politics. Some people disturbed doctors and forced them to go for home visit. These are the part of politics, part of nasty politics. [smiling]

I: What do you think where RMPs are prescribing in absence of doctors which type of medicine they give?

R: They give very worst medicine. They give the medicine also perform delivery. Also do the wash. If something happened they refer patient. They are very dangerous. Their practice should be banned.

I: What do you think as nurses are getting training, is it also possible to train RMPs as no one is there?

R: 100% is not possible. They will exist. It’s better if they refer timely. After doing harm to patient, half of the antibiotics were used. Patient’s conditions are not improving. Public awareness is very important. You can’t do anything until public get educated. When you tell me doesn’t go to RMP, I understand because I am educated and I know it is harmful to visit RMPs. But the poor people they are not educated. They don’t know that in health centre doctor and medicine are available. They will go to RMP. RMP take the money as well as give the wrong medicine to him. But still they will go to RMPs because they are not educated. So medicine of all treatment is development as Modi ji said. The treatment is education. They are needed to be educated. Proper schooling is needed as they understand good and bad. They only it is possible. Today I know what is good or bad because I am educated. If they ask you to do home delivery, do you agree on that? After marriage if your mother in law asks you to do home delivery, do you agree on that? [Pointing to the interviewer]

I: No.

R: Why? Because you are educated. Don’t want any risk. So ultimately it is education. Education is needed to give.

I: Do you think they can they give First Aid?

R: Yes. Everyone can give first aid.

I: Does MR visit you with literature?

R: yes. Lots of.

I: Usually what they bring to you?

R: They bring their product monogram; explain about their product as their product is best. No medicine in the world other than them.

I: Did you write that medicine?

R: They give the idea that you read in Google. They do the marketing from there. They also told the benefits of old medicines. That is the marketing strategy. Okay.

I don’t know what all you’ve written- stop it now.

I: Okay.
