# Supplementary material for: “Without antibiotics, I cannot treat”: A qualitative study of antibiotic use in Paschim Bardhaman district of West Bengal, India
Source: PLoS One. 2019 Jun 27;14(6):e0219002. doi: 10.1371/journal.pone.0219002 (PMC6597109; doi:10.1371/journal.pone.0219002)
Supplement: S2 File — (ZIP) [file pone.0219002.s002.zip › S2_Transcripts/KAP 238.doc]

Interview: KAP 238

Age: 42

Place: Asansol

Highest education status: B.Com

Occupation: Pharmacist

How long: 5 years

I: So you keep antibiotic in your pharmacy. Which type of antibiotics do you keep?

R: We keep all type of antibiotics. Like fever or other injuries, we have to check first then we give antibiotic.

I: Okay. The antibiotics you keep, what are the reasons to keep these antibiotics.

R: Doctors prescribe these type of medicines so we have to keep. We [hesitating] don’t prescribe antibiotic by ourselves. We only keep and give medicines which are prescribed by doctors.

I: So [*cut off by respondent*]

R: According to doctor’s prescription.

I: Yes yes. Does it ever happen that patient came to you and asked for medicine by themselves?

R: Yes they ask for but we don’t give all type of medicines. We give for fever **mumbling inaudibly** like Calpol, Crocin, paracetemol*.* Then we give medicine for latrine but don’t give antibiotic.

I: You don’t give antibiotic

R: No. Without doctors no antibiotic is allowed.

I: Not allowed. Okay, so what does a typical patient encounter look like, meaning the patient comes to you…how, meaning?

R: They [means patient] told us to give antibiotic then we [patients] don’t have to visit the doctor. But we [pharmacist] don’t give. Why do we take so much risk? Doctors are allowed to give antibiotic not the pharmacist. This is the rule. According to the rule this is the regulation.

I: Then why they [patient] ask for medicine by themselves?

R: Yes to be well. To heal their body/treat their illness. What else.

I: Okay. What is your opinion about antibiotic?

R: My opinion if the patient taking antibiotic then has to fulfil the course. If they don’t complete the course then it would become resistant. Then later if we charge the antibiotic means if doctor charge the antibiotic then it will not work. So it is important to complete the course not to have the resistance. But patient doesn’t think about that. They just keep the medicine. It is just sitting there. But doctor will give full course of medicine. Who is needed to take for 5 days doctor gives them for 5 days. Who is needed to take for 3 days, doctors give them for 3 days. All the antibiotics are available in the market are stacked according to the dosage. If some medicine has the course of 5 days then 5 tablets will be there in the strip. Means public don’t think about that. They take 3 tablets and left rest 2 tablets. Then later become resistant. It doesn’t work, that is the fact. Ask more. Also antibiotic is important to give. It is not that without antibiotic the pain will not get cured …**stuttering*.* But it [antibiotic] is important to give to cure pain. If we don’t give antibiotic then he has to suffer from pain. And for other things, we have to go. Or get admitted- anything.

I: The patient comes to you for antibiotic; you give them after checking prescription. Do you tell them anything? Antibiotic **cut off by respondent**

*R:* **interrupting** Yes! Total! Whenever we serve antibiotic we tell them to take like this and when to take this and has to complete the full course. Don’t leave the course. After finishing the course there is no more medicine. For 5 days of course we give medicine for 5 days. Don’t give medicine for more than course. So you have to take for 5 days. We tell this. Now it varies man to man.

I: Okay- so, uh, in this, can you say a little about the average antibiotics you keep- the supply chain in this, like you bring it, you use it, so what…meaning, on what factors are the supply chain based on?

R: *[pausing]* Based on, meaning?

I: Like the supply chain- suppose you brought this antibiotic-

R: **cutting him off** We get antibiotics from the market only, according to the companies. Whatever antibiotics the companies make, whatever distributors they have, we get antibiotics from them.

I: I see.

R: So we get it from them. And there- to get it, we have to take all the documents- for example, we get antibiotics- for other medicines also we have had to give all our documents beforehand. However many distributors there are, everyone already has all of my total documents. From my driver’s license to the shop name- everything is already given. Uhh- GST number, etc, everything. Address, etc. everything. Then only they make the bill. Then, after making the bill, we get it. So we have to go to every distributor- whatever kinds of antibiotics they all have- eh? *[stuttering]* we get antibiotics accordingly. Like we get all the other medicines, we get antibiotics the same way as well.

I: In this- do you procure more medicines based on which one sells more? Meaning, what does the distribution look like? With respect to your antibiotics?

R: Yes, however it gets sold, we keep the stock accordingly. And anyway, we-uh- give. So the doctor as he- uh – prescribes, his- heavy antibiotics for instance- those would not be written by just any general physician. Only a specialized doctor would be writing that. Meaning one who understands it well. So then he **stuttering** he writes it, and we have to keep his medicine too. Like that, I have a lot of doctors, approximately…many many doctors who prescribe all this- once they prescribe, we have to give.

I: Hmm you just mentioned “bhari” [heavy-duty] antibiotics, so what kind of antibiotics are these?

R: What do you mean bhari antibiotics?

I: For example-

R: Did I say bhari antibiotics?

I: You said “like bhari antibiotics we give-“

R: Heavy heavy antibiotic! I, meaning- for instance, like Cefaxim. With Lisolid. Right? All this- these are “bhari barkam” [heavy-duty] antibiotics. 500 Cefolaxim with 600 Lisolid. These are all bhaari antibiotics. They are also more expensive. So these- meaning- are useful when someone is heavily injured. Right? If it’s not healing completely. If the entire wound is not healing completely. So then we have to give for this. We have to give then- bhaari antibiotic and everything. Meaning- doctors write the prescription, so we have to give.

I: So you must see a lot of prescriptions, right?

R: Absolutely.

I: In this, what is the most commonly used antibiotic? Which one do you see most frequently?

R: Yes- Amoxicillin with Potassium Clavonate is the most common antibiotic. Meaning- your day by day- as it is going day by day- for people- the resistance power will increase. Resistance power. First I give one Azithro to get the work done. Azithromycin. Complete. It all gets taken care of with that. By giving Azithromycin, it gets taken care of. Now there’s two- Amoxicillin. Potassium Clavonate. Now this is also not working. So we are having to give Livofloxacin. So, it’s Amoxicillin-Potassium Clavonate- with Livofloxacin. Three antibiotics are being given- together- in one person. So this is the situation. Resistance power is increasing- after you’re doing this day after day.

I: Okay- what you just mentioned- that three antibiotics you said are being prescribed together- so is this referred to as combination antibiotic, right?

R: No- this isn’t a combination. Combination has not come into the market yet. The three-combination drugs have not come yet. Whatever has come has been banned. Those people, I mean, this wasn’t done. So then, what we do, we have Livofloxacin separately and Amoxicillin with Potassium Clavonate . We give these two [Amoxicillin with Potassium Clavonate] together and give Livofloxacin separately to make it into 3.

I: Okay.

R: So then, the right thing happens for the patient. Otherwise, it won’t work. What to do? Now the doctors are also helpless- doctors are also helpless and have to give. Because the patient comes to the doctor to consult the doctor and get better. If the patient…does not get the right treatment by the doctor, then why would the patient come to the doctor?

I: Right. So then the combination antibiotics that we have- what kind of demand is there for them? Meaning, what is the demand for them in the community?

R: Nah, in the community, it is according to what the doctor prescribes. That’s not about demand- demand meaning the public doesn’t ask for it directly. But uh…even if the public asks, we do not give. We tell them to go and see a doctor. Go consult a doctor and then come. So they come to us with a prescription after consulting a doctor, and then we serve them the drugs.

I: But approximately how many patients must you see in a day? Meaning how many patients must come to the shop?

R: To me?

I: Yes, over here.

R: Approximately 30-40.

I: 30-40? So among these, how many ask for medicine by name directly? It’s not about you giving it to them. Meaning you don’t give that I understood.

R: *cutting interviewer off as phone starts to ring—*let’s say 2 [out of 30-40 patients].

I: So how many of the patients who come here, 40 patients who come as you said, how many ask you directly for medicine?

R: Antibiotics? Yeah I mean 2-4 ask for it. It’s not like that. 2-4 or 5 ask us…give us antibiotics so we can…get better.

I: Among these, are most generic or brand name drugs?

R: No no, there are no brand name. They leave that to us. At most, they would say we have…this condition, give me an antibiotic, I will get better. We don’t even know any brand names- nothing. They just leave it to us. Meaning, the people who are patients- the people who come here.

I: Okay uh- tell me one thing, the broad spectrum antibiotics…that you just said about broad spectrum antibiotics, so what is the…in the community, what is the demand in the doctor? Meaning how many doctors give it and how many…?

R: See, as the patients come, the doctors prescribe accordingly. As the patient uh…the type of patient, the doctor only prescribes antibiotics after seeing what problem the patient has. Otherwise, they do not prescribe antibiotics. Even the doctors, meaning, prescribe antibiotics after seeing the patient, what problems the patient is having. It’s not like they are just prescribing antibiotics to anyone…doctors also think carefully before prescribing antibiotics…all doctors, some doctors don’t even prescribe antibiotics. It’s not necessary that they will give. They see what problems the patient has, what it is, then okay, let’s give them this to give them some relief. They do not need to prescribe antibiotics. Simple.

I: But the…the broad spectrum antibiotics- mostly, do you see that people are giving broad spectrum antibiotics or is it narrow spectrum for one disease?

R: No, no, no, broad sp- ehh—spectrum also they give.

I: Yes.

R: And for narrow…for narrow spectrum, meaning mostly the doctors sparingly use antibiotics. They have to see all the medicines and try to treat the patient.

I: By giving other medicines?

R: By giving other medicines. Not antibiotics.

I: So meaning what if there’s cough and cold?

R: Yes, yes, that the doctors look at other medicines- they, they…if antibiotic is not required, they [doctors] do not give antibiotic to them. And where…where it’s required, you have to give it to them, then they give.

I: So what do you think that these broad spectrum antibiotics, how are they- are they used appropriately or…?

R: No, it’s used appropriately because the problems that the patients have, if after seeing the problems we do not give, then the problem doesn’t go away. And the problems that are happening or not happening in the body…that’s secondary. That’s the next step. First, if after the person has taken…*stuttering…*antibiotics, if they are about 40 years old, he takes antibiotics, and all the antibiotics that he has taken have an effect at the age of 60 years, means any problem on the body, inside the body, any problem, then at least the person lived for 20 years healthily? After that at the age of 60, a person goes on anyway. That’s why for now whatever problem there is, the doctors have a responsibility to fix that problem right now.

I: So these broad spectrum antibiotics- meaning in your opinion, how is this related to antibiotic resistance?

R: It is related to antibiotic resistance. If he doesn’t eat antibiotics properly, or doesn’t complete the course, then resistance will develop. And after resistance has developed, then the second time or next time, it will not work. And after resistance develops, there will be a problem. So you will have to give a high dose. And by giving a high dose, there will be a higher effect on the body- meaning, there will be side effects. That’s why developing resistance is very bad. This is why patients should always complete the full course of antibiotics.

I: So…and do you also say this to them? Meaning to the patient?

R: Yes, all of them uh…anytime. Whenever I give antibiotics…*stuttering…*meaning uh after seeing the doctor has prescribed them, I tell them right away that see, you have to take this for 5 days and complete it for 5 days, so that if the antibiotic…if you do not complete this course, then this will become resistant and then it’ll be very, it won’t work the second time, this antibiotic. Then you will get a higher dose. Then it will be a problem. We also explain this way, even in such a crowded setting. Even when it’s crowded, we tell [the patient], we tell them. This is important to eat. Like that-

I: And this resistance which you mentioned, what other things can increase resistance?

R: Resistance, well, other than antibiotics, you do not see resistance in anything.

I: No, meaning, within antibiotics, you talked about antibiotic resistance, meaning the dose that they have, they eat it incorrectly.¨

R: If they take the dosage incorrectly, then antibiotic resistance happens. Antibiotics…other than that, we do not see antibiotic resistance anywhere. In my view, we aren’t seeing it. Uhhh doctors are giving antibiotics properly. No one else that I can think of.

I: So if a patient comes to you, and you told him to take the full course, but he did not continue taking the medicine after 2-3 times. Then he came to you and you noticed that he did not eat. Then, what do you do?

R: Then I tell him that the antibiotic course that is there- if you do not complete it, there is a chance of resistance. There is a chance of developing resistance. Resistance doesn’t happen anytime. It depends on the person’s body. As in…meaning how it happens. For some, it happens, for others, it does not. Often times, it does not happen. But this…is not a very, I mean, “bhaari bharkam antibiotic” like Amoxicillin Potassium Clavonate. These are not that “bhaari” [heavy duty/powerful], which is why resistance does not happen. So we make it work.

I: So then only the powerful antibiotics-?

R: **cutting him off** There is a higher chance with that. A higher possibility. According to the means see…it can increase. Then after getting resistance, then I mean problems develop. Doctors have to think about what they should give to make the patient better. Then they have to think about that.

I: The patients that come to you…mostly suppose if you want to give antibiotics, so do you give, I mean, the full 5-6 day course, whatever the course is, do you give the full course at once or do you…?

R: **cutting him off** No, I give the full course at once.

I: You give it at once. And if they come- how often does it happen that they come back, and tell you that the medicine is not working, does anything like this happen generally?

R: Yes, it happens, it happens.

I: How often, meaning what percentage of the time must this happen?

R: That, meaning, that is also not very frequent. But it happens. Now, we have to see whether after eating antibiotics, he has eaten the complete antibiotic and then also his illness has not gotten better, then the doctors have to conduct tests, etc. Now, whatever comes in the test…whatever comes from the test, accordingly.

I: Mhm mhm mhm.

R: Why this antibiotic did not work. What did not go correctly. This will be go for testing. After going for testing, however the report comes, the doctors will then prescribe accordingly.

I: So this medicine…uh…given- given by the doctor, so there are three members involved in giving the medicine. There is the doctor, the patient, and the pharmacist, so the- how much, meaning, what kind of dynamic involvement is there, between them? The dynamic between these three, how- what kind of relation is there when it comes to giving antibiotics?

R: To give antibiotics- the doctor gives them. [**shuttering sound, cutting off by the respondent*]* These doctors [**asking for permission to start again]*

I: Yes, tell me tell me.

R: The antibiotics that the doctors give, according to the doctor’s prescription, according to, according to that only, we give to the patient. And what we give, the patient also eats. Simple. Doctors gave. We did not give. Doctors gave. [**raised voice, firmly]* What the doctor gives, they eat. [*quiet voice*]. After that, if it doesn’t work, for example sometimes 2 patients have not gotten better till now, there was a simple fever, cough and cold. So the doctor has already prescribed an antibiotic. So then they came and signed up for the doctor for tomorrow to ask why this has not gotten better. So then they are given a test. Then you have to do a test tomorrow, he will bring the results again after doing that, the doctor will see it tomorrow. And after that, however the results come, the doctor will see accordingly.

I: So I would like to know, let’s assume the doctor gave one antibiotic, he prescribed one and you do not have that antibiotic, so then what do you do?

R: If we do not have, then we say that it will be available tomorrow so come take it then. Show the prescription and take it. I do not have it. Right now, I do not have it, if you can, come tomorrow and show us and we will see how the doctor has prescribed the medicine for them to take. So, if we see that it will also work if you take it in the morning, then we take the risk otherwise we don’t. We tell them [the patient] directly that take the prescription elsewhere to any pharmacy and take it from there.

I: Okay. So whatever the doctor prescribes, do they mostly write generic names or brand name…?

R: [*interrupting*] No, brand name.

I: Why do you think this is the case, mostly brand name?

R: They should not prescribe brand name. But they write brand name. Now the government has said not to prescribe brand name medicines.

I: Why?

R: Why, because they must think the doctors take some money from the company representatives or something to do this. So that’s why they think this must be some kind of advertisement. Then the doctors are taking some gifts or something and prescribing. That’s why they said to prescribe brand name instead of generic name. But the doctors had started with generic medicines, and then shifted to brand name.

I: Why?

R: This…[*hesitating*] I do not know, even the government did not provide such a tight schedule [means: regulation]. When they do, then generic medicines will show up. But the thing is, whatever antibiotics etc. whatever medicine we procure, what you get in the government hospital, that does not work. They say 50% discount, 60% discount. By selling the medicine at a discount, will he bring the medicine from home or something? No he won’t give. He will not give from his own pocket. He will sell whatever all the government’s generic medicines. Their 3 CALPOL tablets are equivalent to 1 of our CALPOL tablets. After eating one of our CALPOL tablets, the fever will be gone. After eating 3 of their CALPOL tablets, the fever does not go away. This is the trick with 60% discount. So this, we, like you have this brand name, whatever brand name medicines we sell, the molecule etc. is always up to mark. I don’t know what the other situation is. We do not sell that medicine, so I do not know.

I: Meaning in that medicine, what…?

R: [*cutting off interviewer*] I don’t think the molecule is correct, which is why this happens. It’s not about that- brand name or anything like that, theirs is also brand name. Whatever brand there is, such as Galaxosan, you also get the generic medicine of that. But this is very different, why I do not know. Even the molecule that they show does not stay. That’s why it does not work as well. Our medicine works right away. We see that their medicine, whatever medicine you take, even after taking 5,6,7 of them, it shows little effect. The power of one of our medicines is the power of 6-7 of theirs. I see some difference in the power.

I: What is this molecule- can you explain this a bit further?

R: [*Laughing*] Molecule, this molecule thing, this medicine that you are seeing, it is what is inside the medicine, which makes it work. That is, means, this drug or this medicine, that is what works in the drug [*Incomplete*]. That is a chemical. Oh it’s a chemical. Different types of chemicals are there in any tablet. There are chemicals. They are chemicals. It is the chemical that works in the body.

I: Okay, the patients that come without prescription, when you take a decision to give them antibiotics- what is the reason behind this?

R: The reason behind this, meaning, the problem of the patient, we have to treat the problem. First of all, this is our duty. Meaning, every pharmacist, every doctor has this duty to treat the problem. However the patient is- reduce their problem. In any how and any condition. Whatever we have to do to reduce the problem, we do. Even if we have to give an antibiotic, or some other medicine. Meaning we will treat the problem. If we need operation theatre, then we will take him to the operation theatre. If we have to do an operation, we have to do an operation. The doctors think like this.

I: No, what I meant to ask was if you wanted to give an antibiotic to a patient after examining them…

R: [*cutting off interviewer*] You do not give to everyone like this.

I: No no not like that. Meaning, if there is a need to give, so what illnesses do you give it for? For which illnesses do you think, alright, let’s see for 2-3 days?

R: No we, whatever illness there is, for example your, meaning direct antibiotic…why do we give direct antibiotic? The doctors who give direct antibiotic means in order to treat the problems. But we see, doctors see what the [patient’s] problem is. According to the problem, meaning if you have a fever, we will not give you an antibiotic right away. You have to take Paracetemol and try it out first. We see whether you are getting better after 1-2 days of eating Paracetemol- if it works, it works, otherwise antibiotics. And the people who are, the public has become over-smart now- they go directly to the chemist instead of the doctor and they keep Paracetemol in the home. Whenever they need it, body ache, or a fever, right away they pop 1 Paracetemol tablet. After taking Paracetemol for 2 days, if it doesn’t get better, then they go to the doctor, otherwise they do not go.

I: So there are some patients who come and say give me for 2 days or 3 days.

R: [*cutting off interviewer*] Antibiotic

I: Antibiotic.

R: No.

I: Then what do you do?

R: No. We do not give. We directly tell them doctors [*incomplete sentence*]. After 2-3 days or afterward…if they give now, then they’ll give for 1.5 days, 2 days, or 5 days. They won’t give. We will directly tell them to wait for the doctor- after waiting for the doctor. Whatever the doctor prescribes, whatever antibiotic he prescribes, we will give. Without doctor’s prescription, antibiotics are not allowed to give the patient [*emphatically*].

I: Absolutely no patient?

R: [*interrupting*] no patient!

I: So I would like to know that the drugs which are nearing the expiration date- how does the distribution of those drugs work?

R: Expiry drugs? [*didn’t understand the question*]

I: Near-to-expiry drugs.

R: Near to expiry. We see what means the patient has, suppose a drug expiring in December comes to me in November. Comes to me in November. After coming in November, if it is expiring in December, that the patient is…for how many days…[*hesitating*] how many days they would need the course for. If they need the course for 10 days, and it’s approximately Nov 7^th^, then we give them that drug. If he will eat it for 10 days, it means the course will complete on the 17^th^ [of November]. So we can give, and we do give. If we see that it’s the 25^th^, and the drug is expiring in December, then we do not give the drug. We keep it separately and it goes back to the stockist. Right now, the position is such that no stockist is taking that drug, and we are having to throw all the drugs. The drugs that are expired, we are having to throw them. They go in the drain.

I: So how do you discard it- meaning it goes in the drain?

R: We burn them or throw it in the drain [*inaudible*]. Otherwise, we burn it, means we find some space and we burn it there. We destroy it.

I: And if there is a fever?

R: if there is fever, then you have to give Paracetemol.

I: But in either situation, you will not give antibiotics if there are no other symptoms?

R: No. And even if there are symptoms, so then go to the doctor and he will prescribe and we will give.

I: And if someone has diarrhea?

R: [*cutting off interviewer*] Yes then we give them plain medicine. To stop diarrhea, no antibiotic. If it stops with that, drink ORS. ORS, because if you have diarrhea, the body loses salt. So it’s important to make them drink ORS. If there’s also vomiting, it’s important to give them Electrol. So we give everything. And we give medicine to stop diarrhea. No antibiotics.

I: And if he has stomach pain?

R: For stomach pain, we give a pain tablet. Then we give a tablet.

I: What is a pain tablet?

R: No antibiotic. Plain pain tablet.

I: And if someone develops rashes?

R: If someone gets rashes, then we tell them to take Lacto Calamine etc. We give some Cetirizine, that’s it.

I: Is that a peripheral antibiotic that you give to put on top?

R: No no no. There is no antibiotic in that.

I: Can you tell me a little about…if a patient comes and asks for 2-3 days of medicine, what is the reason for this?

R: Money. People don’t want everything [*incomplete sentence*]. Yes there is one reason for not eating medicine- it’s an allopathic medicine- you should not take a lot. This thought is in their head. And the second thing is that we, India, is a very poor country. If they can make do with little medicine, then they take little, simple.

I: So if someone comes and says they do not have money, I only have money to buy medicine for 2-3 days, what do you do in this situation?

R: Then, we give 2-3 days of medicine, but antibiotics do not come like that. It won’t come in that. Then we, the patients who ask for 2-3 days of medicine, then we only give them the medicines that are required. But when we look at antibiotics, if they are required, then we tell them to see a doctor. Then the doctor sees. Whatever medicine he prescribes after seeing the patient, whatever antibiotic he prescribes, we give it to them. If the patient happens to be short on money, then they can give it afterward, but take it with you- by eating this, make your problem go away. This much, we do. [*laughing*]

I: And this antibiotic resistance you have mentioned before as well- so one thing is the patient doesn’t take the medicine properly- what other reasons are there for this?

R: Resistance?

I: Yes, for resistance.

R: *Long pause.* I can’t think of any reasons. This depends on bad weather, etc. everything else. This is the case. This much I know. Something related to weather too, which leads to antibiotic resistance.

I: Okay so other than medicines, other than medicines, what other reason is there? Weather is okay, maybe it’s a reason, but any other reason other than medicines?

R: Other than medicines, there is no other reason. There is only one reason for antibiotic resistance. And other than medicines, only the doctors can tell you. I cannot tell you. [*in low voice*]

I: So that means if we want to stop antibiotic resistance, so what you said before, there is the doctor, there is the pharmacist, and there are the community members as well. If we look at all of these people, all together meaning how can we stop resistance? What solutions are there to stop it in your view?

R: The first thing with doctors is that you can tell the doctors to stop giving antibiotics. And if the doctors don’t give antibiotics, then we will not give antibiotics, sell antibiotics. So doctors are the main way to stop antibiotics for you. But the thing is if the doctors stop antibiotics, the problem will not go away. The problem will remain. And if it remains, then the patient won’t go to the doctor. So how will you stop this? Right? If doctors cannot treat patients, cannot make the problems go away, then why would the patient come to the doctor? Then what’s the purpose of the doctor? So then it’s also important to give antibiotics. It is needed to give antibiotic. Without antibiotics, nothing can be done. It’ll only cause you trouble…[*trailing off sentence*]. If there is a wound, if there are a lot of rashes, and if you do not get antibiotics, then your wound will not heal completely no matter what you do. You will have to give them antibiotics in order to get rid of it. Otherwise, you will have to tolerate the problem. Simple.

I: I had one question, which is- how involved are you in giving antibiotics?

R: Doctor prescribes and we give. Simple. On our own volition [*saying firmly*], we, we meaning any pharamacist, do not have the courage to give antibiotics [without prescription]. Means means- we do not have the courage because we are not doctors. Doctors know what will happen after prescribing antibiotics. We do not know all this. Because he is a doctor. We are not doctors like that. I am a pharmacist- their job is to see the patient, and give them antibiotic to treat them. What is the function of the antibiotic, what molecule is inside this, knowing these two things is my job. What is his job? That’s an antibiotic for stomach or for pain, what kinds of antibiotics there are, an antibiotic for fever, knowing this is the pharmacist’s job. And next you have to know what molecule is in it. How much, what is it…that’s it. These two things are important for a pharmacist to know.

I: In your view, do most pharmacists think that they cannot give antibiotics?

R: I do not know. Pharmacists do not think about all this. Some pharmacists who know about this, know about medicines, they think about this. And the people who do not know, who do not know anything about medicine, they do not think about it. If they have to sell, they sell it.

I: So here you are the pharmacist. And the other people-

R: [*interrupting*] they are staff.

I: They’re staff. So do they also give out medicines without you at times?

R: No. You saw yourselves that you had to wait outside. I came with the key for the shop and opened the shop, then the shop opened. The key isn’t with anybody, they do not have the key to open the shop. You waited one hour for me. Then I came, gave the key, and opened the shop. So how will they prescribe antibiotics? [*smiling*]

I: Okay so this antibiotic resistance- how dangerous is it in your opinion? How dangerous is it? Can you tell me a bit about this…

R: Resistance? Resistance is very dangerous. It gives you so much trouble afterward, means to the doctors, means you have to go for testing. After going for testing, which antibiotics are working for which things, which are resistant, we have to see. After seeing that, then they select whichever antibiotic will relieve their problem. Then they give. Then they have to go for the test [*repeating his sentence*]. Some are resistant to Levofloxacin, this one is resistant to Ofloxacin, that comes in the blood test. Then after seeing it, meaning they …antibiotic [*trailing off*], then they prescribe antibiotics. Developing resistance creates a lot of problems. A person’s illness does not get better.

I: Is there another name for this test?

R: Yes. That, I mean, I don’t know. The doctors give the test, that much we see. Yeah, right now I don’t remember, what’s the name of test…[*trying to remember and trailing off*]

I: And one more, you are a pharmacist. You also run a shop. So as a pharmacist, what do you think, what should the role of a pharmacist be in controlling antibiotic resistance?

R: Pharmacist’s role? [*confused*] The pharmacist’s role should be that whenever a doctor gives an antibiotic to a patient, whatever antibiotic you are serving to a patient- you should explain everything to the patient when giving the antibiotic to them. What they have to eat, how many, for how many days, then resistance won’t occur. Then resistance will not happen. If the pharmacists do not play this role, then resistance will happen 100% of the time. If pharmacists, any pharmacist, takes on that role properly, and tells the patients this is how it is, you have to take this and for 5 days or you have to take it for 3 days. And if you do not take it, then there is a chance you will get resistance afterward. If resistance happens, then you are the one who will face problems. That’s why it is important that every pharmacist has to tell the patients. This is a vital role of the pharmacist.

I: Okay. Would you like to say anything else about this? Antibiotic resistance, whatever your opinions may be, anything.

R: Antibiotics, see, antibiotics which are required for every patient, means it is required to give to patients who need it. Because we see a lot of the problems that patients face. So after seeing these problems, we ourselves do not understand how the patient will get better. So then the antibiotic is required. When we see the patient crying, we also feel like crying [*getting emotional*]. Then you do not think whether antibiotic resistance will happen or not happen, we do not think of this. Then, the doctor gives and we serve it right away. We tell them that they have to eat like this. Explained all the details about the antibiotics that you have to eat it like this, you have to complete this, and the patients eat it too – it’s not like that. Often, patients eat it, means approximately 90-95% patients eat all the antibiotics that the doctor gives them. 5-7% of people are a bit clever, too clever, extra cleverness that they have, which is why they eat it for 2-3 days and stop it after they start feeling better. This means- very little [not many people].

I: How do you know that very few people do this?

R: Very few people do this because we find out when the patients come to us. Next time, when they come, we ask them if they completed the course. Yes we did. We also ask.

I: And do you find out from symptoms [*cut off by respondent*]

R: No no, finding out symptoms means…*[hesitates]* you will not find out anything from symptoms. For such symptoms, you do not find out so quickly for patients. If we see afterward, long after. Approximately 10 years, 15 years, or 20 years later, you find out that resistance has developed.

I: Alright. Okay ji- thank you so much. Thank you.
