# Supplementary material for: “Without antibiotics, I cannot treat”: A qualitative study of antibiotic use in Paschim Bardhaman district of West Bengal, India
Source: PLoS One. 2019 Jun 27;14(6):e0219002. doi: 10.1371/journal.pone.0219002 (PMC6597109; doi:10.1371/journal.pone.0219002)
Supplement: S2 File — (ZIP) [file pone.0219002.s002.zip › S2_Transcripts/KAP 239.doc]

**KAP 239**

Gender: Male

Age: 31

Occupation: allopathic doctor

Highest education: MBBS

I: So doctor, for starters can you describe your typical OPD setup? How many people come per day?

R: Uhh in our OPD at Raniganj BPHC, on an average, 300-400 per day…patient come…different uh- with different types of diseases. [*pauses*]

I: Such as?

R: Such as, uh, common, such as fever, diabetes, hypertension, uhh injury…[*long pause*]. And uh, diarrhea, vomiting, pain abdomen, etc.

I: OK. So let’s take these one by one. If you had your um let’s say just a cough and cold- what would you typically treat them with?

R: Firstly, we go…firstly we take total history. From how many days he is suffering, and…is there any fever, or uhh…is there any past history of several type of illness? If it is 1-2 days…we, uh, give…such…painkiller such as Paracetamol, and anti-histaminics. And we follow after 3 days. If symptoms persist, then we go for further examination such as laboratory test consult, such as sputum, uhh…or if necessary, x-ray, and if fever persist or go on high temperature, then we prescribe antibiotic. If we think that there is absolute need of antibiotic…only at that time, we’ll prescribe antibiotic.

I: OK so you don’t start with antibiotics?

R: No no no no.

I: OK. And is there a lab setup available here…in the BPHC?

R: We have, uh, we have a lab, but uh there is primary few…uh uh test are being done, such as uhhh…malaria test…uhh..TCDC ASR, that is CBC…and uh, uh, we have also facility of platelet count and sputum also. But uh, we uh have one extra facility that is PPP model testing facility that includes various types of test- blood test. Huh? That includes most of the test which are regularly needed on common basis for follow-up or exclude any type of illness or fever or any disease. That is- that facility is also available here.

I: So do you do culture and sensitivity?

R: No. We don’t have…that facility of culture and sensitivity.

I: So how do you know when you- an antibiotic will be required?

R: [*long pause*] Suppose…uh one patient came to me- come to- one patient comes to me. [*pause*] uh with a fever…firstly I take total history, uhhh, then we first uh, send the patient, send the patient for malaria test. If it is negative, we uh, uh prescribe them this anti-pyretic and advise him or her to come after 3 days. If fever persist, then we umm advice for CBC- that is complete blood count. And on that basis, if we uh think that there is any high WBC count or low WBC count or CRP is high, depending on that report available, we…often uh, prescribe antibiotic.

I: CRP is C-reactive protein?

R: Yeah.

I: OK. Umm so the PPP model that you mentioned for the facility- where is this facility?

R: Actually, it is…uh, it is a private setup. The agreement is- has been done from district level, and representative of that facility [*phone rings* *and interview is paused].*

I: You were telling me about that lab…PPP.

R: Yes, PPP model lab uhh…facility, that is a private setup and agreement is from district level authority, and their representative come here regularly and they collect blood as doctor’s advice. And uhh…upper…the test result they submit reports to the patient, and patient come to us with report.

I: Does the patient usually come back with report? I mean, what does it usually cost the patient?

R: Cost?

I: Yeah.

R: 50%, meaning total uhh…total test value that is 50% supplied by government and 50% patient…

I: Out of pocket?

R: Yeah.

I: OK, OK. And do most patients who you tell to go and get the test- do they come back with the test result?

R: All patient come.

I: All patient?

R: All patient.

I: OK, that’s great, OK. So coming back to the diseases that you mentioned…you said fever, you mentioned cough and cold- what if somebody comes with watery loose stool with or without vomiting? What would you typically do…in that case?

R: Um…first of all, we have to…consider the age, that is ummm the patient is below 1 year, or 1 year to 5, or 5 to 12, or 12…more. If a patient come with stool…meaning, stool frequency or loose stool below 1 year, we just prescribe ORS and uh, lactobacillus. That may be [inaudible] or solution that is consumed orally…and uh patients 2-5 years, depending on the age and type of stool uh, we firstly go for ORS. We…actually, we maintain, we try to maintain that fluid level when treat the diarrhea patient. First of all, we give [*inaudible at 7:43*] that is fluid level in the body is decreased or normal. Huh, depending on that situation, we go for O- this fluid supplementation and we follow. Actually we follow. If uhh, it worse, the situation of the patient get worse, then we definitely go for…uhhh, stool- sometimes stool cultu- sometimes stool culture from outside. And if there is any infective cause, then we start…antibiotic. Uhh actually…uhh in Raniganj and Asansol, there is…I think there is no facility for sensitivity… sensitivity. This test only…actually available in medical colleges, Bakura or…Bardhaman Medical College. So…we…just…see whether there is any infective cause…

I: By taking a history?

R: Taking history or a stool examination. Examination, not sensitivity and culture. Then, we go for antibiotic. And sometimes, we need culture, sensitivity- then, we refer patient for higher center for that purpose.

I: Where would that be?

R: That uh, Bardhaman Medical College.

I: OK, OK. Alright…

R: And uh, if the patient is umm…more than 12 years, then…we…prescribe Metrozil, firstly, and follow for 3 days. If, uh, it subside then it’s okay. And if not, we go for…fluroquinolone antibiotic.

I: OK, but when a patient first comes to you, do you typically give an antibiotic then and there with the ORS, or not?

R: ORS.

I: Just ORS?

R: Just ORS.

I: No antibiotic?

R: ORS and…PPI…

I: Which is?

R: PPI which is Pantoprazole. Sometimes, we give Famotidine…and…sometime, whichever is supply at our end.

I: OK, OK, since you brought up what is supplied at your end, can you tell me a little bit about what is involved in stocking of antibiotics? What that process looks like?

R: Yes. We have various types of antibiotics, such as…uhhh, Metrozil, Metronidazole, Ofloxacin, Ciprofloxacin, Amoxicillin, Cefuroxim, Levofloxacin, Cefalaxim…[*long pause*] uhh, Sulfamethoxazole-trimethoprim. These are oral, and injectable are…Ceftraxone, injection Ceftraxone. Ceputazim. Metronidazole infusion, Ciprofloxacin infusion.

I: OK, and are most generic or brand name?

R: All are generic, because these are all DRS supplied.

I: OK, and do you have a preference for generic or brand name for your own- this thing?

R: Personally, I uh, don’t. I um…most of the patient, I prescribe which medicine is available at our end. I uh-ye- most of the time…when patient come…someone else, who uh…gone for another doctor outside then come to us and uh…when I check what medicines he have taken…uh, most of the time, most of the time I maintain my level that is government is supplying medicines for those people or those persons, so whatever is available, I prescribe them. And uhh…usually I don’t prescribe from outside or any random.

I: OK, OK. And do you detect any difference between generic and brand name medicines?

R: [*long pause*] At our facility?

I: Or in general from what you have seen? Is there a difference between brand name and generic drugs…in quality, efficacy?

R: I don’t have any idea.

I: You don’t have any idea, okay great great. What about if we come to uh, combination drugs vs. monotherapy? What do you prefer and why?

R: Combination- this monotherapy is better than combination therapy obviously.

I: Why is that?

R: Uh, because, uh suppose one patient come to you…with a cold. Common cold. And you prescribe him or her a medicine that contains 4 or 5 drugs, no 4 or 5 medicines. But actually, he needs 1 or 2 medicines. That is sufficient for her symptoms to subside. So, it is- it is much more better, I think, that you go for…this monotherap- this generic medicine depending on patient’s symptoms. Unnecessary other medicines should not be prescribed, I think. It is my opinion. Others what do I- not comment.

I: What is the effect uh…if any, of monotherapy vs. combination drugs on antibiotic resistance?

R: Definitely there is.

I: Can you explain that a bit?

R: Actually…suppose, uhhh…one patient…uhhh…coming to you your or me- with fever, or with other symptoms, which and…on that basis, he need antibiotics. Antibiotics. He need antibiotics. I don’t know the organism that is causing this symptoms, or this disease, is…sensitive to this antibiotic or these- or these etc. I don’t know. I…uhhh…if the, uh, culture and sensitivity test is available, then it is easier to me to choose one, okay? Which will add to my- better, better. But, as I don’t have, I…depending on my medical knowledge, what the- I think that the organism is gram positive or gram negative. What the organism…huh, what type of organism is inside the body causing this symptoms…depending on that, we go for that, this drug will be better for him as organism may be gram positive. Or this may be better as this may be gram negative. So first of all, I have to decide- I have to decide what type of causative agent is inside him. Depending on that, I will go for one antibiotic. If unnecessarily I prescribe more than one, that maybe two or three, okay? Actually that is not necessary for the patient. That will make…become resistant in him in future. So, it is better to think and decide…on the situation of the patient what antibiotic…uhh you will prescribe, only one or two or more…that is depends on the patient coming.

I: I see. And what about the availability of combination antibiotics in the market?

R: [*long pause*] Mark- in the market?

I: Or in general, among physicians? What do you see?

R: There are…lots of.

I: Do you…feel like it is prescribed more than monotherapy or what is the preference that…people give?

R: Uhh…if you…take my opinion or consider my opinion, I think…uhh from today…uhhh…3-4 years back, there was a tendency to use combination antibiotic- combination therapy, but now, most of the doctor I think, the government- the doctor working at government setup…go for the monotherapy.

I: Ok. In the government setup?

R: The government…setup.

I: And what about the use of broad spectrum antibiotics?

R: Broad spectrum antibiotic…actually, antibiotic…there is misuse antibiotic- there is obviously misuse of antibiotic is happening…around us. And most of these misuse is being done by the quacks or…medicine shop- owner of medicine shop- who don’t have degree of pharmacy and they don’t have knowledge on medicine. They sometime give patient 2 or 3 or even more antibiotic for a particular disease.

I: [*pause*] Hm.

R: [pause] And you asking me what is broad spectrum antibiotic?

I: Yeah yeah yeah. And what are- what is the use, availability, and impact of broad spectrum antibiotics?

R: Actually, broad spectrum antibiotics are those antibiotics which covers…more than, more causative agent uhhh…therapeutic index is more!

I: Okay, okay.

R: I think those are the broad spectrum antibiotic, which can be…prescribed…uh a- uhhh- a huge number- more and more number of – in case of more and more number of infection and diseases. And uh, obviously, there is impact of broad- broad- broad spectrum antibiotic in prospect of thinking that uh…the future…the future is actually not secured uhhh what is going on and it will uh…cause resistance to most of the broad spectrum as well as other antibiotics in near future which will create a great problem in our medical as well as health problem. So, we have to…think which is- what is necessary and what is not, and ummm we have to follow the rules that is…made by government, or WHO, or big NGOs who are uh, um, making and highlighting consciousness about- on use of antibiotics. We have to follow, but…if we don’t take any steps or government or other don’t take any steps on those particular quacks or medicine shop owner, ti will be really cause a. huge problem in health in near future.

I: Mhm mhm. Mhm mhm mhm mhm. What are the drivers for antibiotic use or misuse in the government setup…around doctors?

R: Actually, there- there are different types of uhhh doctors. And they have personal view and thinking on antibiotic use. So, uhh it is…[*pauses*] so it is not possible to highlight all of the thinkings of doctors…uh, from me in this regard…

I: How about the doctors here under your in charge? What happens in the BPHC?

R: [*quickly answers*] Most of the doctors um…follow antibiotic policy.

I: So there is a strong antibiotic policy from the government that says…do this, do that?

R: Definitely. Definitely government- uh, definitely um um…have a uhh…antibiotic policy, and I think most of the government doctors follow. Few…may not.

I: Does the patient ever demand antibiotics? When they come?

R: Huh! It’s a good question. Sometimes, patient come…this antibiotic- I need this antibiotic!

I: They say by name?

R: Yes?

I: They say by name?

R: Yeah.

I: Like give me Ciprofloxacin?

R: Ciprofloxacin. Give me AmoxiClav tablet! I don’t…actually, most of the patient come…our OPD, they are the…lowes-local, general people and they, I think they don’t have enough idea on that antibiotics, whether it will…good for him, or absolutely- or necessary or not they don’t have. Sometimes, they checked- they are checked by someone else by outside and he- tha- that doctor prescribe a antibiotic. He call him 2-3 days purchasing outside, and after that he or she will be coming at us, and told…kindly prescribe me this tablet. This antibiotic. Ummm…sometimes, it is...meaning, just…we astonished that patient is coming and asking for prescribing this type of medicines as they don’t have any idea whether it si for this…whether it is for- uh- it is necessary or not! Patient come. There are…suppose…uhh…in 30, 300 patient, you will find 3-4 patient at least…they…come and…tell you give me this medicine.

I: Around 300, 3-4?

R: 3-4.

I: Around 1% of your patients.

R: Around 1% of patients.

I: OK, OK. Mhm mhm mhm mhm. Wow. And how much time do you take to counsel the patients? If you have 300 patients coming, how much time do you get per patient?

R: Actually…300 we…uh…we have sanctioned post…6…at our BPHC, and we have already…at now 4. So, we are going at…shortage. We are running hospital in shortage, so when two doctors run the OPD, uhhh…around 2:30 pm, we start 9:30 am and we…closing OPD 2:30 or 3 pm. And think there are 300 patients. And per doctor, 150 patients. And we getting how much time? [*long pause*] 5 hours to…5 hours 30 minutes. So, so you can calculate what is the time we are able to give patient. [*laughing*]

I: Sure, sure sure.

R: Most of the time, it is not possible for us to take total history, and sometimes we…we have to…treat patient…on the basis of whatever they complaining at presently.

I: Mhm mhm mhm. What is the perception of the patient…of the doctor in the government set up specifically?

R: [*long pause*] It is a…different type of question. So…uh…whatever I feel, when I was…school student…I have enough- I had enough faith on doctor and respect to them. We sometime told that this person is Doctor, I am going- we actually give some respect on that time. But, today, there is little…change in thinking of public- common people, so most of the patient respectfully come and honor us. And few of them…they are…they think…what can I…[*phone rings*] they think they are…they are all along- all [*trailing off*]

I: They know everything?

R: They know everything, and they have all right to talk any type of…uhh languages, any type of behave.

I: Out of 10 patients, how many are like that?

R: Out of 100…6-7 at least.

I: Out of 100, 6-7?

R: 6-7.

I: Ok, Ok. so still not a majority though? Small number of your patients?

R: No, no. Small number yes.

I: Okay, okay and do you-

R: But the overall view is being change-

I: is changing. I see I see. Do you feel this affects your prescription patterns at all?

R: No no no. It doesn’t and I think it never.

I: Mhm mhm mhm. And what about patient perceptions, in your opinion, of private vs. government- where people go. We know in India, out of pocket spending is very high- many people prefer private. Why do you think that is?

R: I don’t think so.

I: You don’t think that’s true?

R: No.

I: OK, mhm mhm.

R: I think most of the people uhhh…take facility from government setup. And uh…some- some who are…capable of bearing cost, sometime they take facility from outside or private setup, but now, if you see, at all everybody, you will see- you will find people from higher level umm…they are also coming at our facility.

I: Really? Interesting, interesting. Okay, coming back to something we were going through- illnesses- how commonly do you see common skin problems in this area…rashes and all?

R: Common skin problems are…the…patients uhh…out of 100 ummm…10-15 patient come with skin problems, and most of them are scabies. And few are fungal infection, few are dermatitis.

I: How do you treat these problems? You can take one and give me an example.

R: Think- a patient coming with scabies. We have Benzyl-Benzoate lotion.

I: BB lotion?

R: BB lotion. That is government supplied. We give BB lotion along with one anti-histaminic. If it is- if we think that there is secondary infection, we sometime prescribe antibiotic also.

I: Are topical antibiotic normally applied? In the beginning?

R: Topical antibiotic we give only on- depending on the skin lesions. If you think one patient come with abrasion, and he actually not…ummm…uhh meaning, actually it not necessary for systemic antibiotic. We prescribe, that is, cream or lotion or ointment. [*pause*] And uh…if it is…if we think that it can’t be managed on…ointment or cream or lotion, then we prescribe systemic antibiotic also.

I: OK, OK. What about if it’s a respiratory tract infection?

R: Respiratory tract infection…hmmm out of 100 patient, uhhh…it will be around 10- 10 person- 10 people.

I: And how is it treated typically?

R: Typically, depending on the present complaint- if patient come with…cough, then we prescribe cough syrup, and we take history. If it is more than 7 days or more than 10 days, we do sputum. If there is nothing in sputum, we go for chest x-ray. And…or…if we see in the chest x-ray, there may be something…which is not detectable in the x-ray, we go for CBNAAT, that is cartridge-based nucleic acid amplification test. Uh- for mycobacterium tuberculosis, and…depending on the report, we treat. If patient come with distress, maybe asthma, COPD, bronchiectasis…depending on wha-what patient is complaining, we go for medicine. If…patient is complaining only distress, she is not able to take enough air inside, we give bronchodilator. And…on clinical generation, if we find that there may be sign of infection, we take preventive measure…sometimes with antibiotics. And uh…if patient come- if known patient of COPD or tuberculosis come, we follow them- how they maintaining our advice, and following our advice- whether he or she consuming regularly medicine or not. All we check.

I: Mhm mhm mhm mhm- so typically do you provide antibiotics for uh… preventing secondary infections?

R: Secondary infections? No no no.

I: So you don’t provide okay? So there’s no use of antibiotics as a precautionary measure?

R: No no no, never.

I: Okay and that’s not recommended also?

R: Yes.

I: Okay okay. So, if- if 10 patients are coming, again, on average, how many would you give antibiotics to? Taken altogether, huh, not for a particular illness or anything.

R: 10 patients?

I: Yes. Out of 10 patients or out of 100 patients, how many get antibiotics? [*pause*] at this facility?

R: Uhh…10-15% patients get antibiotic.

I: 10-15%?

R: 10-15%.

I: What are the risks and benefits of antibiotic use? In your opinion? And how do you weigh the risks and benefits?

R: [*clears throat*] unnecessary…this uh…unnecessary…unnecessary antibiotic use is not recommended. I think- I think if you need, you prescribe. If don’t, you don’t do that. And you have to…um, think uh…the present situation and also the past, that is near future. So, we have to preserve some antibiotics for our next generation. I think…unnecessarily antibiotic is…use must be condemned.

I: What are the drivers of antibiotic resistance…mainly?

R: Mainly unnecessary and uhhh…illogical use of antibiotics.

I: OK. Any other causes?

R: [*pauses* and hesitates] sometimes, patient…take medicine from directly from medicine shop. Um uhh if you- check, few people or a few household, whatever they do on- if they get uh, ill- sick- some- most of them will tell you that they take medicine from outside and that even antibiotic is not necessary at that stage, they take medicine- they are taking antibiotic. And that also causing antibiotic resistance. Overuse of antibiotic- that is uh- uh- one patient need 5-7 days antibiotic therapy. And if you think, after that, this antibiotic- there is no role of this antibiotic, and some prescribe antibiotic for 2 weeks, 3 weeks. Even some patient come that they are taking antibiotics for 4 weeks…or more! That will also…cause resistance.

I: Hmm hmm hmm. Any other causes… at all?

R: [*long pause*] Uhh…if you thinking, one patient with gram positive bacterial infection and…you giving one antibiotic, that may cure the disease. On that situation- on that case- another case, meaning same type of another case…when another doctor or another person, they giving 2-3 antibiotics. That also causes resistance.

I: And what are strategies to combat antibiotic resistance? How can we tackle it?

R: We have to strictly follow the guideline, and have to strictly maintain the antibiotic policy.

I: Antibiotic policy. Provided by?

R: Provided by the government or international NGOs.

I: International NGOs, OK, OK. And uh, any other strategies that we can use?

R: [*pauses*] Ummm…I mainly follow government protocols.

I: What are your attitudes toward say…continued medical education (CME)? What are your attitudes- what do you think about it? Do you access such programs?

[*looking unsure, shaking his head*]

I: Not particularly, no. Do you know if antibiotics are- are involved- are explained in CME programs or no- any idea?

R: I attended when I was…intern- intern. I attended a workshop on antibiotic use at our medical college. Uhh…

I: Okay. How are the CME sessions organized? Meaning who organizes them? Which body? And where are they set? Generally?

R: Generally, who organize them? [*pauses*] I don’t have enough idea on that. You- meaning…I attended that one or…?

I: No generally. And yours was organized by the college right, your medical college?

R: Yeah.

I: Okay okay. Is that the case normally- do you know?

R: No [shaking his head indicating he doesn’t know]

I: What can be the role of a doctor, pharmacist, a nurse- those involved in the medical setup- to combat antibiotic resistance?

R: *[long pause]* Uhh…obviously doctor is the main pillar…for particular disease, prescribing- what medicines he is requiring, doctor is prescribing. And role of pharmacist and nursing staff in that situation is only- follow their doctor. [*pauses*]

I: What to do if a doctor is not available? If you go to rural areas, at the subcenter level maybe, there is no doctor. Right, sometimes? So what to do there?

R: Actually, there is one instruction from block level that uh…do not use antibiotic…do not use antibiotic. If use- consult with our doctor…and do only after that. After consultation with doctor.

I: Does that happen in reality?

R: No no no no.

I: So what is the reality?

R: Actually…subcenter. Subcenter level- mainly the national programs, those national programs which are organized by our government are being followed up as per guideline, and they give, at subcenter level, immunizations- immunizations as per government guideline are also given. And they have few halogen tablets if there is any diarrheal outbreak or waterborne disease or something- then they distribute those tablets among those areas, and usually they don’t use antibiotic, without consulting our doctor.

I: Okay, okay. Hmm and do any medical representatives come to this office? Talk to you- or?

R: Not particularly in this office. They come- they come. At OPD.

I: OPD? So what is that conversation like? What do they talk about?

R: Conversation are…they actually uh, come and…sometime they…ask to prescribe those medicines which are not supplied by government. If absolutely they are required. Otherwise, usually, medicines are given from our end.

I: Do they provide scientific evidence…for the medicines they are talking about?

R: Actually, we are handling lots of patients…and we don’t have enough time to converse…in that situation, so we actually don’t uhhh…give enough time to them.

I: Yeah, makes sense, makes sense. Okay, how do you manage patients if they take the improper dosage? If you tell them a particular dosage and they don’t follow, how do you manage patients who do that?

R: Actually, we…calculate dose…depending on age or umm weight. Sometimes, uh, patients from outside come that they are taking or consuming medicines or syrup or something that is improper dosage, that is low or less or more. We actually tell them that this, for this medicine, the dose should be this, so follow this dose. Actually, we can’t take any step [*phone ringing*] actually we cannot take such steps- outsider who are doing this types of improper dosing- we don’t have guideline to follow, so…if you come, you will find some patient they are taking medicine with improper doses. Most of them are from outsider- outside. They uh, most of them are from quacks. They don’t have medical knowledge or…

I: What is your perception of the quacks?

R: Quacks are those who don’t have medical degree. They don’t have medical knowledge. They working in past under a doctor…and after 3-4 years…under working doctor, they open up a chamber and uhh…they write on board Dr. this. We check this, this, this type of patients. I think actually, they are 10^th^ pass or HS pass- most of them. So, what will be the impact of…their thinking or their work in that service they are providing to the beneficiaries?

I: What do you think? What is the impact they are having?

R: Definitely bad impact. Because if I am a student- uh if I am a- I have completed only 10- class 10, and I worked under you- you are a doctor- I worked under you 2-3 years. I acquired some knowledge- this antibiotic for this disease, this medicine for this disease- and I…open up a chamber and I am checking patient and advising them, so what will be my ummm…meaning, what will be I mean…actually I want to say meaning…

I: You can speak in Bengali.

R: Bengali means…they don’t have the knowledge, the thing I don’t know…meaning, how to do this thing.

I: How to treat?

R: How to treat.

I: If a medicine is near to expiry, does it affect your prescription pattern?

R: Actually, we don’t use expired medicines. I…we use only those medicines which are within expiry date.

I: How often do you check?

R: 2-3 months before expiry.

I: you check? Okay. Before prescribing each medicine, do you check the expiry date?

R: No.

I: Okay.

R: The, uh, pharmacist checks…actually, our medicines come from DRS quarterly- that is every three months. So, uhhh…those medicines [that] came in previous quarter, we use them first, and uhh…medicines coming after that, we keep for next use.

I: And the ones that are expired- how are they disposed of?

R: We have SembRamky Medicare. Those expired medicines, we hand over them and they treat according to guideline.

I: So, and, and have you heard of delayed prescription practice?

R: What?

I: Delayed prescription practice?

R: Delayed?

I: Yeah. Like okay what if you do if you feel an antibiotic is not required immediately, right now, but say a patient lives far away and you are unsure of follow up. What would you do in that situation?

R: We don’t have any scope.

I: Any scope. So what would you do? Let’s say a patient is coming from very far away.

R: If a patient came from UP- Uttar Pradesh- you give medicine. And if he return back, then we don’t have any scope or any type of communication to make to this patient- have follow-up.

I: And out of 10 patients, what is your follow up like? I mean, can you give me a rate or something of how good follow-up is?

R: Almost 70-80% patients are coming.

I: 70-80% is good follow up. You have follow-up?

R: Yes.

I: Okay. For how many days you can give…medicine to a patient?

R: We generally give uh…three days.

I: three days?

R: Three days.

I: Antibiotic also?

R: Antibiotic also?

I: Why is that the case? Why 3 days and not the full course?

R: That is…ummm…thinking that all patient come, coming for facility and all are able to take medicines, thinking on the demand. Huh? Actually, we give 3 days because we- there is various criteria. One of the criteria is there is shortage of staff. One of it. Second, if you give three days medicine, the follow up will be better. And it will be also a better thinking that whatever you prescribed in previous visit, if at all those medicines are working or not- it will also…will be maintained good. There are different types of thinking, so we decided give three days for better public service.

I: Is this recommended practice? As per guideline?

R: Ahhhhh…as such, no guideline. And we give uh, 30 days medicine for hypertension, diabetes…

I: What about specific to antibiotics? So, if you have a full course, you don’t give the full course at one time?

R: No.

I: So you tell them what? What do you tell them? 3 days and then come back?

R: I’m…uh uh I tell them I’m giving 3 days medicine. You come after 3 days. And if I think there is – this medicine is working good, then advise full course.

I: And if it’s not working?

R: If it’s not working, then we have think to alternate-

I: Meaning do you then go for a higher antibiotic or do anything else?

R: Ummm…we sometimes, we change antibiotic to another group.

I: And then would you give for another 3 days? How would you test that?

R: Then we give for another 3 days.

I: So approximately patients, out of a 100, complete the full course of antibiotics?

R: Out of 100…50 to 50% patients complete full course. Some patients…we prescribing 3 days…if they are feeling good, or the symptoms subside, some of them don’t come in the next visit.

I: How do you cope with that?

R: We don’t have any scope.

I: [*chuckling]* Anything else you want to say as we wrap up? About antibiotic resistance or antibiotic use? Anything at all?

R: No that is [*smiling*]

I: Alright, well, doctor thank you so much. This is very helpful. Thank you!
