# Supplementary material for: “Without antibiotics, I cannot treat”: A qualitative study of antibiotic use in Paschim Bardhaman district of West Bengal, India
Source: PLoS One. 2019 Jun 27;14(6):e0219002. doi: 10.1371/journal.pone.0219002 (PMC6597109; doi:10.1371/journal.pone.0219002)
Supplement: S2 File — (ZIP) [file pone.0219002.s002.zip › S2_Transcripts/KAP 248.doc]

**KAP 248**

Gender: Male

Age: 31

Occupation: allopathic doctor

Highest education: MBBS and dental degree

I: So Doctor XX how long you have been here in this capacity?

R: 5 and half years. 5 years 9 month.

I: Ok. Ok and what is your typical OPD sitting like?

R: Typical OPD setting like is just a basic set up with basic things and we also provide basic medicines. .(quick pause) and we deal with emergencies but that is up to a certain capacity and not beyond that

I: Ok

R: we got to refer that as we don’t have much of sterilization set up or operation set up like that so much we can do with the medicines we deal here, rest we refer to outside

I: So what kind of emergencies do you see?

R: Road traffic accident mainly, but particular that I deal with mainly road traffic accident and few comes are the deliver emergencies, the labour emergencies so that’s well dealt here because we have that set up. But mostly these road side accidents…these are the thing we cannot manage here because of the infrastructural inability so we have to refer it somewhere where the sterilization is proper.

I: And why do you refer to those patients?

R: Asansol district hospital because that is the first referral unit we have over here and so…(pause)

I: Ok. And are there typically patients need care beyond that point?

R: Yea…you do get few patients which are manageable by medicines like AGV patients, fever patients,

I: Listening deeply by making nodding sound

R: Like acute abdominal pain patients….(mobile rings) those patients are typical patients and we do manage them as we have the infrastructural set up over here (quick pause)..thats not an issue. But mostly the problems with the patients over here and related to your topic ; they go for self-medication.

I: I see. .you tell more about that.

R: They …….to the situation in such an extent…they go to pharmaceutical shops, take medicines from there, have it by themselves and by the time they comes back to us, its really gets difficult for us to diagnose first what is the problem because they have some medicines of the other ..before that which is masking the original effect and then they are coming and approaching us, so sometimes it really get very difficult situation to diagnose in most the cases..(sigh) Well then fine, GOD´s grace nothing happened yet.

I: Ok. Ok. How do you handle such cases when that happens?

R: We try to take history first. But unfortunately the patient we deal here, 90% of the patients are illiterate. Typical illiterate patients so they couldn’t give proper history also.

I: Listening deeply by making nodding sound

R: And more we face the situation with quacks. Raniganj is full of quacks. Everywhere you go you will find quacks. (Quick pose) They comes and says (indicate patient) “Ek bari kaayeka aaya” (had one tablet^[1]^) “Do bari kaayeka aaya” (had two tablets), ¨Paani charake aaya”..they gives us prescription then asking for treatments. .. ¨Paani chara dijiyea tho thik ho jaayega”..So this is the problem…(Quick pause) We are basically trying to get the history which is not proper every time. In most of the cases we get a wrong history. .. Now when the patient is getting cured or we are managing the situation in a successful way, slowly slowly we come to know over a period of time, and with a long interaction, suppose a day or two, what is the exact scenario. Initially they doesn’t exactly reveal what’s the scenario. So that’s a big issue over ere. Few times, prejudice also attached to that.

I: What do you mean?

R: Like ..(mum for a second) they have got some Godly prejudice . Specially let me tell you with the menstruation thing, they have got few prejudices…so if somebody comes with the pain abdomen, if you ask them about their menstrual cycle thing, and the gynaecological set up they are going through every month..they will pretend to hide the thing..out of the prejudice or out of the shame; I don’t know but they don’t want to reveal this. This is one easiest example, I can cite now and there are lot more. Like in case of chicken pox, they call it with a local name like ¨Maayer Ashirvaad¨(Mother´s^[2]^ blessing) or something. You know Bengali or? (Pointed to the interviewers)

I: NO. I (responded affirmatively the other interviewer)

R: ¨Maayer Ashirvaad¨ or ¨Maa Dekha Diyeache (Mother has come) , it is like that like GOD has come given this to them. So they just reluctant to bring the patient out of their home so most of the time, when the localites are bit literate, bit aware about of the health situation, they gives us a call, we go there, we practically insist them, force them, to get into the house, see the patient, and inform them what to do what not to do. Their (indicate patient´s party) basic thing is ¨Kuch maat khilaao, kuch maat paraao, ek jaaga me side karke rakh do tho thik ho jaayega”..thats Godly thing, that’s come on its own and go on its own. So I think basically it is right. It comes on its own and go on its own. But we have to maintain our physical status to throw it out of body without harming ourselves So that thing, few these things are difficult to handle in Raniganj. Not only in Raniganj, I think most of the rural places of India, it is the same scenario.

I: Hmm. So what is the patient´s perspective on the medicines that you give?

R: (thinking a bit, making sound) Most of the patients that I said, they think antibiotics the main treatment. If they come simply for a dental pain, they will ask for antibiotics first without letting know what is the exact scenario or if they come for simple if you see a road traffic accidents, having pain in their arm or feet, anywhere, they wi ask for antibiotics first. “Antibitic de du, thik ho jaagega). So they go for self-medication, so..even before coming to us…that day, I dealt with a patient with a knee injury and severe laceration. It was severe laceration with patella are exposed literally. We skin the muscle ..tendon was exposed very badly. Part of the tendon was cut off

I: Listening deeply by making nodding sound

R: That patient met up the accident the day before he came to us at night. The whole night he was under the treatment of a pharmaceutical shop and quack. Next day morning he came to us for stitching. So I told him “Bhai hum tho stitch nehi kar paunga” but I don’t know about what the others will do…I mean the bigger set ups because that is the problem after 12 hours the patent is attending. We have almost lost the vitality of the part..of the soft tissue, we lost the vitality. Re-suturing is more dangerous over here….we can kill that patient in that scenario. Right?

I: Listening deeply by making nodding sound

R: So that goes under the plastic surgery then again we have to cut out the C-section, and XXX and then go for de-suturing …So I referred him. This is the thing that is really the big shortfall in our health set up. Even we can do it, we are unable because of patients perspective. They moreover prefer to go for the quacks and the local medicine shop. That’s a shortcut, coming to the hospital, getting an OPD ticket, getting the consultation of the doctor will take an hour, but going to the quack beside their house will take 5, 10 minutes. (Sigh) so that is the problem here in Raniganj.

I: What are the other drivers besides time that get patient go out to the quacks?

R: See, if you ask me on behalf of the patient, I can say without insulting or hurting anybody ; may be sometimes our behaviour from our point of view is bit rude or what but if you ask me about myself, we can guarantee this I deal each and every patient in a way he or she is my friend. Because I believe when a patient comes to you, until and unless that patient gets relief from you, mentally he won’t allow you to work on him.(interviewer nodding sound) He needs a mental security first , the person I am dealing with is a good person ….he will deal me with the ok ok thing. So behaviour is a think you should take care upon. Specially in the government sector and on the Government side we have less staff, so you have worked with us, you know (pointed towards Rina, the other interviewer) we have go severe staff crisis and severe patient load so staff is to patient ratio is so less, it is almost….(fumble for a moment) majority of our staff are aged one, more than 40, 45 years. So for them to keep patience and deal with each individual patient, that to be illiterate who doesn’t even know how to talk to others. So it gets difficult for them to keep patience…sometimes they get rude that I know. If something like that happen in front of me I do try to stop it and doesn’t linger further because if I tell you something wrong, you will also tell me something wrong. (nodding sound by the interviewer) so that will linger the thing. But it happens sometimes I know.

I: So per day what would your patient load be?

R: OPD patient or emergency patient?

I: I guess you have done both.

R: OPD patient tho (a Bengali affirmative) , you came over here (pointing to Rina), 350 to 400 OPD patient we deal. And we have at least one or two doctor to deal with that (smiling with a sense of irony) , one pharmacist, one nursing staff max to max 2 nursing staff in a day..and nursing staff got to deal with the indoor, (Mane) I mean the indoor patients as well as EPD patients also. So…(Murmuring a bit) emergency patient (tho) ..there is actually no calculation for that sometimes if there is a RTA bus accident then at a time 5o patients will come. And I have seen days, like no single emergency has come to us. (rIght said the interviewer) in 24 hours. So that’s pretty unpredictable for us

I: And how many patient in the IPD?

R: IPD, we have a 25 bedded hospital, this is so sometimes it is 40 sometimes it is 15 sometimes 5 sometimes one depending on actually the…the environment, depending on the climate also. Because in the winter, not much patient we get to admit. .but specially during the monsoon in summer mostly we get more patients so during that period of time. In this winter we get deliver patients mostly. Deliver patient, labour patient come and..(murmur for a moment)..this is another hindrance that there between patient is to Government hospital ratio, patient thinks (ki) if we go to the government hospital we will not get a bad, we have to lie on the ground. And the scenario is not clean enough so …It is my personal opinion of hospitals are not clean because of the staff only because of the patients also. Well in your state if you have to go to any hospital (referring to Mohit), you won’t spit anywhere. Forget about the State (USA), if you have to go to a corporate hospital even, we won’t spit. But when we come to Government hospital, we feel like spitting there, isn´t it? (smiling together); You have staff crisis to clear it out.

I: Yea

R: Well, last 3-4 years back, I started a rule actually, unofficial rule. If patient spits in front of me, I will force a the patient, I used to force that patient to bring something from outside, either leaf or paper and clean it out. So.

I: How did that work out? (Smiling together)

R: That worked out pretty well but actually, but this mentality should be practiced by all the staff

I: Yea (collectively)

R: Tho (Bengali), I alone cannot change the whole scenario. Ultimately ***ek time ke baad*** (after some time) I also got fade up ***chor de*** (leave it) (Smiling together) So..

I: So typically how much time you take per consultation?

R: (smiling), Should I tell honestly or dishonestly?

I: Honestly, always honestly (smiling together)

R: one to two seconds. Before the patient enters, we almost write down the prescription. …because we don’t have any other options. Our OPD time is actually from 9am to 2pm. So due to our (pause and fumble for a moment) or whatever we start OPD 10, 10.30

I: (Yea)

R: Madam knows (pointing to other MSF interviewer). And we end up sometimes 2.30, sometimes 3.00, sometimes 3.30, handling up 340, 400 patients and unfortunately the OPD patients, most of the OPD patients come for nothing. As because, the hospital is within the locality, so while going to the market, while going to the shop, anywhere they just come here to peep. Is there any doctor? And a normal complain. ***Do din se khujli ho raha hai.*** (having itching for 2 days) O kaal thora do baar potty huatha (yesterday had 2 episodes of lose motion)…these are the basic complains who come over here.

I: Nodding in agreement (sound)

R: And if you tell them, and there is no need of your medicine, and you say go, they will insist you where there is no medicine. (Pause)

I: Nodding in agreement (sound)

R: When your organization was with us^[3]^..so 50% of the patients we got to deal with the patients who go to you why there is no medicine (smiling) ***Is me tho koi daawi diha hi nehi***. (No medicine is prescribed). Yea kaise hoga? ***Do din se bukher hai, ek daawi deke chor diyaa***. (having fever for 2 days and have been prescribed only one one medicine!)^[4]^ Remember that? (Smiling) (Asking MSF interviewer)

I: Yea

R: This is a thing, we actually we can provide much time to the patients but when we feel his patients really needs care, so what time exactly do we give beyond that patient, we don’t know. That may be 2 hours, that may be 5 minutes that may be that may be 10 minutes…until and unless we get to diagnose the case, until and unless we start the treatment or decide what to do, we go for that patient. But most of the cases 1 to 2 seconds, maximum 5 seconds because if you divide time, 5 hours with 400 patients (nodding sound made by the interviewers) , you will get that statistics itself.

I: So how does that affect your prescription pattern at all?

R: I mean, hugely. Most of the patients when I write a prescription I doesn’t even know what I am writing, matching with the exact disease process or not. That really affects the treatment procedure and the worst thing in heath service in last 5 and half years with me is I forgot half of the name of the medicines. When I get a serious patient like that I have to devote time on it. I got to bite my pen over 5, 10 minutes to remember what medicines to be given on this particular treatment. Even in case of antibiotic selections also sometimes we really fumble because we have got specific few antibiotics supply over here. So patient coming to us, having a serious complications, selecting the best antibiotic and empirical the basic one for them is really difficult. ***Matlab*** (Meaning) after working here ceptram DSN, Amoxicillin, for 5 and half years you really fumbled to think about what is the generation, (nodding sound) what is the efficacy of that antibiotic. So really that of matters

I: So what is the other kind of antibiotics you work with, what other typical..?

R: Over here or?

I: Yea.

R: Over here, In our Govt. supply we have this clotamaxizol, we have this penicillin, recently sefioroxim is coming in and metronidazole is there, cetnidazole is recently available with us. ofoxacin is plenty available. Ciprofloxacin, Levofloxacin. ….these are the basic quinolones, basic penicillin and cephalosporin are available.

I: Ok, Ok. Is there preferences generic vs branded drugs?

R: I personally prefer generic medicines.

I: Ok

R: Because I have a concept, I don’t know whether you or others will agreeing with me or not, generic drugs are low priced actually not because of their quality but because they are not marketed to the others in front of the world. (Nodding sound). Ina Brand as far as I know, in a brand a company has to invest at least 200% of the making chares of the drug for marketing but generic doesn’t have that situation. (Nodding sound) Along with another thing I heard from one of pharmacist’s friend if brand medicines get expired, company takes it back. There is no damage for the shopkeeper. But for a generic medicine, if it gets expired it is the shopkeeper’s loss. So they keep the price of the medicine less. That’s why I prefer generic medicines because if I am for example I am prescribing telmicerton to my patient, it is costing 8 to 8.30 a brand piece, 40 mg, 8 to 8.30 brand, in brand it gets. So if he or she is a poor patient, for how many days he or she can buy it and have it (nodding sound) but if I am going for a generic one, it will come around 2-2.30 per piece. So the patient is 4 times more duration he can continue with the drug. That my theory beyond generic medicines over branded medicines. (nodding sound)

I: Is there a difference between the efficacy of the two at all?

R: I never found. Believe me. People says, Govt medicines not so good and all. Believe me I last 5 and half years, personally I am using Govt medicines for my any sort of diseases (nodding sound) and I became dengue struck in 2015 and Trust I got admitted 2to 3 days in Kolkata AMRI in Salt lake. But before that until and unless it was diagnosed to be a dengue, I was only having paracetamol from Govt supply and it worked really perfect. (nodding sound) I was having 1000 mg paracetamol, TDS so it worked really well. So subsiding dengue fever along with the pain (tho), so it is quite effective. Tho, I didn’t find the efficacy of the drug are bad in generic. Now I don’t know, if a particular lot of drug is bad or not, that I don’t know because in our Govt supply also few drugs are sometimes we get the order to pull back those whole lot of whole batch because of the quality. We pull back that drug and get the newer ones from the supply. So can be company has messed up a particular batch Over all the generic drugs are quite good.

I: What is the supply process? Supply chain?

R: Supply chain? Govt supply?

I: Yea

R: In Govt supply, we put on the indent, in Bardhaman, now recently at Asansol (nodding sound), so they send us the medicines, we send our car..so they send us the medicines whenever required. Quarterly, every 3 months, they send their own vehicle, logistics to supply the medicines. That’s the quarterly indent.

I: ok, if drugs are nearly expiring, what happens? What’s the process?

R: There is a rule to intimate the government 3 months prior to the expiring (mane) meaning expiry of the medicines

I: Ok

R: Ok? Suppose we have got thousand pieces of paracetamol, expiring in next 3 months and we have calculation for last so many years, the patient load and all that we will be requiring max to max 300 or 400 pieces of paracetamol. So we can notify that, that we are having thousand pieces of paractamol to be expired on this month and our requirement will be 300 to 400 plus 100 for the extra safe purpose. So this 500 is surplus with us expiring in 3 months. So Government will take that back and supply somewhere where there is crisis but in case of emergency medicines like atropine sulphate or any decadrone, in case of those medicines there is no such guideline. Govt says like ARV also ARV AVs; Govt says if it is expiring , let it expire but you always keep your stock afresh. Matlab (meaning), we are having 10 atropines, but we know max to max 2 or 3 will be required, next 3 months, then also we do not notify there is no harm in that. We can keep it with us because, suddenly we got to need more of atropines than need, so if Govt pulls back that 7 from us then when we need, there is nobody to give us.

I: (Ya; making a sound of an agreement)

R: So that is the system we worked upon. Only for emergency medicines, if it is expiring we do not notify anybody rather we try to sit back and wait if it gets used up, later on we destroy that. In case of other medicines, we notify and it goes back.

I: Do you feel like, in what ways if at all, the expiry of medication affects prescription pattern?

R: Expiry of medication have no relation with prescription pattern over here. (Ok, said I). Because we have drugs here in plenty but what happens specially, during the winter like in winter we, get to have more COPD, patients or more asthmatic patients. Because, because of the climate. So, during that time we may, run short of salbutamol thing. So that to we have an emergency indent system, we can bring it in 1-2 days. And we have got another system also, to purchase medicines from the generic store. Now to purchase medicines in generic prices, that has been given by the govt, a guideline is given by the government that should be below the MRP. .we do that, in case of severe..(Nodding sound by the interviewer)

I: Do you prefer mono-therapy or sort of combination drugs? Can you walk me through that process a little bit

R: Depending on the case

I: Ok

R: How can I say now, that I will go for mono therapy or combination drugs…depending on the patient attending us..and the diagnosis we are coming close to, depending on the case we decide, particularly I decide (emphasized)

I: Ok. Risk and benefits of both?

R: Risk and benefits of both? Like benefits of mono-therapy and combination therapy and the difference I cannot tell you right now sitting in this situation because until and unless I get a case to study and work upon, how can I tell you the benefits but there are always risks with allopathic drug.

I: Ok.

R: that was taught to us on the first day orientation class in pharmacology. (Phone ringing). Drug without a side effect is not at all a drug. So the effects we are getting from a drug are also the side effects. Paracetamol is killing both the pain and the fever. So which one is the effect and which one is the side effect? Until and unless, there is any harmful effect, I considered drugs, these allopathic drugs, have two type of effects. One is effect and one is harmful effect. Side effect is the treatment and harmful effect is the other way round (smiling together). So, anyway it is very difficult to tell you right now the benefits of mono-therapy or combination therapy, separately. Definitely one benefit we have, that we are treating the patients and they are getting well. So that’s a social benefit. ..and risk, we definitely have risk, if we are using irrationally, any drug we are using irrationally either be it mono-therapy or combination therapy or anything , that’s a potential risk to the society overall not only to the patients. Potentiality of getting resistance especially in case of antibiotics. So, and definitely with other complications, GI tract complications and all so , kidney complications, so ther is risk to everything.

I: And what about when it comes to broad spectrum antibiotic?

R: broad spectrum antibiotic, I personally doesn’t prefer much.

I: Ok. Why is that?

R: Because if I know the treatment, if I know the disease actually, what is it? I can very well go for something which is directly hitting that particular organism. Why to go and waste my spectrum basis and create resistance to other bacterias and all. Because that may create resistance to the society overall. Like in “Mejhia”, the block beside Raniganj;

I: Ok

R: So there the main problem with the patient is antibiotic resistance. They go to the shop, this is there at Raniganj also. They go to the medicine shop, for example, if I take about penicillin the shopkeeper gives them 3 penicillin, 3 pieces of penicillin, table or capsule or whatever form, and ask them ¨Aaj raat me kha lena, kal dopor and shyam and kaam khatm**¨( take one at night, one tomorrow afternoon and one at tomorrow night. Job done. )** So that creates resistance not only to the individual but o the society also. That particular organism will definitely change through mutation and attack the other. That will not wait for you or me to find out what is the change. So that is a problem. And another problem is us. That I was telling you before also, I told it yesterday also. In case of penicillin, amoxiclav has been, I have seen doctors, 90% doctors, 99% doctors of Raniganj prescribing amoxiclav in BD dose. 25mg to a adult patient. That’s believe me, that’s a crime. If you don’t know the use of antibiotic, you don’t have any right to misuse it. So that creates resistance.

I: So what conditions are broad spectrum antibiotic preferred?

R: I personally, if you ask me, (Yea, nodding sound by the interviewer) my school of thought, my professor´s school of thought, I personally go for broad-spectrum antibiotic to something which is really a very serious situation, serious scenario and until and unless the culture results come, we are not able to handle the situation with something else. So to start with, if you are sure it is a bacterial origin, start with, try with broad spectrum antibiotic and then wait for the culture reports to come to select best antibiotic for your use.

I: I see.

R: And another thing I sometimes prefer, not always, in case of RTAs, Road traffic accidents, post repair, because I don’t have any much of sterilization support here, basically I work on surgical field, here also, outside also so as because we don’t have much of sterilization support here, I prefer to give some broad spectrum antibiotic in few cases where I know there is a chance of mixed infection. Post repair, might be, that may be nosocomial also, that may be due to the environment outside of the hospital also. So there I give but usually when patients come, I try to select out the antibiotic depending on the case. So..

I: So what about, when you see patient in 1-2 seconds, how does that affect?

R: Actually I told you when I am seeing patient in 1-2 seconds (Yea, nodding sound by the interviewer) I know that patient really doesn’t need any medicines

I: Ok

R: So moreover I go, for vitamin B complex and few XXXX like. (Smiling together). That time I really don’t write ..that medicine that can effect in a way that is harmful. So writing down ppis to patient for 3 days, I don’t think it will be of much harm. And specially in our tropical climate where gastritis is a common problem, with the water we use over here. That’s a common problem, gastritis is a common problem, we can very will go for ppi for hyperacidity and thing. So that doesn’t affect much me when I am treating patient in 1-2 seconds.

I: And how often you encounter common skin problems? What do you prescribe for those?

R: Very often

I: Ok, What Kinds?

R: Every day we at least go through 30 to 40% patient in OPD with skin problems. And what do we do, actually what do we do, we just give them BB lotion. BB lotion

I: Ok.

R: Bejyle Benjoid…and, because now it has been granted out here, most of these patients here are scabies patients due to their lifestyle, the way they stay and it is very contagious in terms of their lifestyle. Ok? Even, in spite of if you ask them to isolate themselves, to isolate their clothes and lifestyle, they don’t do that. (Nodding sound by the interviewer). Once they are out of the hospital, they tend to forget everything, go into their regular lifestyle, normal schedule and (Nodding sound by the interviewer). So mostly we use BB lotion until and unless according to our knowledge if we find something else. If it’s a just a , khujliwala thing (itching) BB lotion is enough, until and unless lea son is visible or any proper history given to us. So in case of proper history, we find it to be, most I personally go for the ointment based treatment initially, just to check out the response. Because over skin blood vessels and capillary are that much don’t help you to get the oral medicines. Oral medicines, it needs a particular plasma concentration to reach that particular area. ..that too so above the internal organs. So I prefer to for the ointments depending on the situation. If I feel it to be mixed infection or and there are chances of having mixed infection due to life style, I go for a mixed ointment having an anti-fungal or anti-bacterial thing, and if I find it to be just a bacteria thing or anti-fungal, I use, actually I hate to use steroid thing. Because that another issue, I have been fighting with my colleagues and outside colleagues also. Why do you use steroid ointments so much? That preresponse can any time turn into severe outcome.

I: Ok

R: Anyway so we deal with ointments mainly but if there is a severe thing patient is a having a generalized skin issues, then we do try to prescribe some oral medications depending on our capacity definitely. (Nodding sound by the interviewer). Because we are not dermatologist, we are not specialist so depending on our capacity, our level of knowledge, our viewpoints, we do it.

I: What kind of medicines in that case?

R: Flucanozole is the basic medicine. And many doctors I have seen preferring flucanozole giving in 150 mg once a week dose. But somehow, my school of thought says, to start with loading dose of 200 mg and then maintaining it to 150 mg weekly. That’s my school of thought. Totally. So I do it in that way. And until and unless I find any mixed source there, I don’t use any antibiotic on skin infections.

I: In general though across the hospitals that you have seen, how often would you say topical antibiotics are prescribed?

R: In our hospital, we use highly topical antibiotics. Topical ointments in antifungal range...few days back we had anti-viral tropical ointment also. So..but those are out of stock right now. They were really helpful in viral infections like your herpes and all. So, I personally use them for two kids, so it really worked like a magic. Without any antiviral therapy orally, it really worked like a magic.

I: Do you perform any minor surgical procedures?

R: Over here, we need to. Few things, that’s within our infrastructure, within our capacity without harming the patient in terms of any infection, acquired infection or not, we do it.

I: And for that, do you prescribe antibiotics? Are they indicated often?

R: I do prescribe antibiotics sitting here at Raniganj BPHC. Honestly speaking. Because as I told you, I know the situation of the sterilization out here. So, going for a surgical intervention, I’m putting into more infection inside the wound, than getting it cured, so I not only prefer oral antibiotics in such cases, I give topical antibiotics also during the surgical procedure. What I do is I use one anaerobic antibiotic like your Metronidazole and one quinolone. Those liquid, infusion liquid, I pour them in the wound, along with Betadin, to keep the area sterile for the time I am working. And after that, while dressing, I use Gentamicin injection. I just break them and apply over it with a sterile gauge for dressing purpose, along with Mupirocin ointment and others which we have over here. So I definitely use- I use hugely over here antibiotic during my repairs, but when I work in the OTs, like in few nursing homes I worked in surgeries, so I don’t prefer to give antibiotics then and per-operative we give- pre-operative, par-operative we give, we give prophylaxis- prophylactically- but post-operative, in most of my cases, antibiotics are not required. If the patient is kept in an isolated way. OK? But in our government setup, you don’t have any option other than that.

I: What about the availability of microbiological testing?

R: Uhhh, for what purpose? For the patients or the institution?

I: For the patients.

R: For the patients, we have lab out here. We have got government rates to that, that functions in public-private partnership mode, so we send there, but authentication of the reports, sometimes I doubt.

I: Why is that ?

R: Because few instances I found different reports from them. Uh proved diabetic patient having no medications, and the other place showing a huge difference in the diabetic profile, they gave the normal report. And many a times, it happens with the haemoglobin thing, especially with the delivery mothers, so…clinically we are finding that cannot be above 6, the symptoms, but they are giving 9, 9.5, 10 results. So authentication, and one more thing- [*chuckling*] but it is B-positive, negative, there was also confusion with that one time with two patients, so one patient came we dealt with it, BMOH dealt with it, but later we found out yeah, they were hepatitis B positive. That’s a huge risk factor for not only the patient and patient party, but also for the operators. Because we are working directly with the patient contact, so…but okay. Treatment is happening, patients are getting reports, they are happy with it, and as I said, 90% of the patients here are with no disease. So whatever reports come, they get better only.

I: So specifically, do you use culture and sensitivity-?

R: [*interrupting*] Yeah I personally use it- I told you before also, I use culture and sensitivity excessively because for selecting my drug, for selecting the drug of choice, you got to go for culture-sensitivity. And that I usually request patient to do it somewhere else, according to their feasibility, their convenience, other than this place. Because I personally won’t trust the report of the culture-sensitivity over here.

I: Is it available here? Culture and sensitivity?

R: Yeah yeah. Yes yes, they take money and they do it. Okay, actually this is unit of one of the lab at Asansol. They have two types of rates- one is the Government rate for few fixed tests, regular tests that we give them- other than that, they perform all tests at Asansol. They send the blood samples from here, and another thing that- because I feel that…they give wrong reports not only because of money, but they tend to preserve the blood for longer time. They draw the blood at 10, 10:30, 11 in the morning, they send it in the afternoon, and the test is performed in the evening or the next day. So vitality of the blood during this time, though it is kept in anticoagulant or whatever, the vitality of the parameters, the organisms won’t stay there or live there for so much time. So…that’s why…I don’t believe I- not believe, I can’t say that sitting in the Government scenario- but I tend to trust outsiders more in this context than others.

I: Hmm hmm hmm- how do you typically treat upper respiratory tract infections and diarrheal illnesses?

R: Upper respiratory tract infections- history is the prior thing. What is the patient exactly suffering from? Is it just a viral thing or a bacterial thing or nothing, but a allergic thing, because here at Raniganj, 70% of the upper respiratory tract infection comes out of uh, allergy. So then we try to give some vasodilators and your bronchodilators along with some anti-allergics and leave it to see if there is any outcome or not. Then, if it is not so, swab testing and all are not available to us. And I personally don’t go for a swab test for a patient coming with a…asthmatic condition or a upper respiratory tract problem, because uh, spending 500 rupees of 2000 rupees for a swab test, they won’t come back to me any further. And I’ll lose the patient, not because- I don’t have any monetary issue over here for losing the patient, but I’ll lose the experience of mine to see what type of patients are over here. Mentally, I go for a bit research thing while treating patients. That’s the nature I imbibed actually from my microbiology teacher- she once told me- each and every patient you go through, try to record it, so that later on in life, when you’re 20 years ahead from your practicing, you know what type of patients you have dealt with at which places. So everything- I’ve got my photographs over here to patients, the prescriptions, and everything. Anyways- so depending on the case, we treat upper respiratory tract infections.

I: OK, and how often would you use antibiotics for the treatment?

R: Uh, that I said, depending on the case. If it is just an allergic case, then- first of all well, what I do is I just give an anti-allergic and a bronchodilator either in a syrup form or a tablet form. So, if the patient doesn’t come back to me anymore, then it can be two possibility- either it has not cured and gone to some other doctor, or it has been cured and he or she is not bothered to come back to me. So, if the patient comes back again, uhh, that it hasn’t gotten better, then we try to go for the diagnosis, that exactly the history taking and all, is there a migration history or not? Does anyone in the family have a history or not? Has there recently been an exposure or not to the…unusual environment and all? Then we go for antibiotics definitely. And my personal choice of antibiotic for upper respiratory tract bacterial infection is Azithromycin initially, a bacteriostatic one. If it fails, then Levofloxacin. But initially I go with Azithromycin 500 mg only.

I: And you mentioned several times about patients coming back with test results- does that happen often or do you lose patients?

R: No no no that happens often, that- you can say it’s my good luck- once a patient is mine, it’s my patient forever. That may be any sort of patient I treated, so that’s a good luck to me. Honestly.

I: Have you- do you usually confirm with sputum or stool cultures before prescribing?

R: Yes yes yes. Definitely.

I: Always?

R: For sure.

I: And what about other doctors that you see?

R: They also do it, because that comes under the protocol. We have few government protocols- in our hospital, uh, we do it- everybody- but outside, the private clinicians I don’t know.

I: Mhm mhm and in these circumstances, under what circumstances would antibiotics be required?

R: [*pauses to think*] If we find a definite general bacterial inoculation or bacterial infection.

I: Hmm hmm. Otherwise no?

R: Otherwise no.

I: Uhh, what is your sort of decision making process when prescribing antibiotics? What factors are driving your decision- would you say?

R: Depending on the signs and symptoms definitely. Duration of the disease. And…patient’s history and above that, at last, if we cannot come to any decision with it, uh the culture and sensitivity test.

I: Mhm mhm mhm- does diagnostic uncertainty ever factor in?

R: Diagnostic uncertainty is always there with each and every cases. Because if we go for a trauma also, if you send patient to the x-ray and all, sometimes you get to get so much of confusions with the x-ray plate, so that’s a bigger thing I’m telling you. A bigger perspective- that we’re confused if there’s a fracture, a soft tissue fracture of anything. So that comes- this microbiological thing, that comes in a way, everytime. I mean when you’re not sure with the reports. When you cannot rely on the reports 100%, so you have that confusion every time.

I: Hmm hmm hmm- you did mention that patients often demand antibiotics, so how does that affect the prescription patterns?

R: I personally don’t give them. I personally try to make them understand what is the use of antibiotic in a simpler way.

I: Do they know what is antibiotic or what is simple- ?

R: [*interrupting*] No. How do they know- if he doesn’t have any-

I: Yeah- means [*interrupting*] if you write a Paracetemol, then they will also not be able to recognize what it is?

R: [*interrupting*] No, few patients know. That’s and they know- actually we have a term in Bengali- if you know in a half way, that is the most dangerous thing. [*chuckling*] Either you know full, or you know nothing. So, people out here knows the half thing. So, Paracetemol they know that’s a fever drug. So if you’re using it as a pain killer also, they will shout at you that- why you have to take fever drug for a pain! And I personally prefer Paracetemol as a painkiller over any other drug. I personally prescribe 1000 mg Paracetemol over anything- [*inaudible drug name*] Diclophenac anything. So that’s a huge issue with, uh, them. They know half of the thing. And antibiotics, they are aware of antibiotics from the medicine shops. If it is a traveller’s diarrhoea, not traveller’s diarrhoea, if it is a diarrhoea of viral origin even, the shopkeeper will give them antibiotic. And the thing is, Metronidazole, they know it’s a…diarrheal drug. It is an antibiotic. It took me 4 years at least to make them understand it is antibiotic. Last 1.5 years, 2 years, I’m a bit relaxed with the patients- if I’m prescribing, they’re not asking that no my stomach is not having a problem. Else, they come and say! This is a drug for an upset stomach- why would we take this?

I: [*pause*] Hm. Uh, do you have an in-house pharmacy?

R: Yes, we have in-house pharmacy.

I: Ok, so what kind of drugs are available here? And do you prescribe- do you prefer to prescribe drugs from the in-house pharmacy?

R: We do prescribe drugs from in-house pharmacy itself. Until and unless it is required, we don’t prescribe drugs from outside. Because the prescription cost is zero, but the drug cost if it comes to be 1000 rupees, how can a patient bear?

I: Right. Right, right, right. Hmm…you mentioned sort of retaining your patients, but at the same time, you’re saying that if the patient demands antibiotics, you don’t uh-

R: [*interrupting*] Actually, I make them understand. For me, the patient:doctor ratio is very friendly. My patient feels free to call me anytime of the day, anytime of the night. I never misbehave with them. If they ask me a single question 10,000 times, I tend to answer them very politely, so that they knows the thing. For me, asking them about the antibiotics and making them aware of the antibiotics is in a very simpler way, not related to any pharmacology or like that. What I usually tell them…that consider your house has been attacked by few goons. So you need to protect yourself? So if you just shout at them or hit them slowly, they will go away and come back in a way- very soon- with more powers. But if you kill them totally, they won’t come back- rather others will also be afraid to come to your house for this particular thing, because your punishment is severe. So if I’m prescribing antibiotic, if there is no goons, if you are throwing bomb at your own house to kill the goons and there is no goons, then you’re getting your house. That means you’re breaking your body with antibiotic for no reason. But now if there is a goon, if there is a…bad person in your house, if you are charging a bomb, charging a bullet- at them- your house is getting harmed, but you have things to recover. But at time, your housing is getting saved from those things also. Those people also. So antibiotic is like that way, and if you’re killing it in a half way, they will come back with more power. So if you use antibiotic like for one day, two day, irrational dose, irrational combinations, irrational thing, so it will act in that way. The goons will go away, come back with more power, and then your power won’t be active with them. Then, you will be having those medicines, but no work. So it’s better you complete the course- so that the wound dies, and never comes back to you again in the same capacity. Might be other goons can come, but they won’t come back to you. This is the way I try to make them understand in their language even in Bengali or Hindi so that they understand. Because whenever I write antibiotic over here, we have a system of writing medicines for three days at a time. We actually prescribe for three days. We’ve got two intentions for that- one is after three days, the patient will come back to get the medicine, and we will know whether the medicine is responding or not. Another thing is- giving lot of medicine together- patient thinks that what will happen with so much medicine- they throw it and go away. So that’s a very difficult thing we face. So, most of the cases, patient takes that medicine, goes, and doesn’t come back in the next three days to take the medicine, but after 17 days- ah I didn’t get any benefit from this medicine, prescribe another medicine to me- so…it happens. But my patients- they definitely come after 3 days- whenever I write prescription, I ask them to take it for 6 days. I ask them- take for 3 days, come after 3 days to show me, and then take for another 3 days. Initially, when I came here at Raniganj five years back, it was a difficult scenario for me also to motivate them to take the medicines. But now they have understood- even if I’m not there at the outdoor…not performing the duty, they come, they go to the pharmacy, even they tell the pharmacist that- you have to give the medicine, doctor sir has said to eat the medicine. So it’s an achievement for me you can tell, and I’m really happy to have this type of patients out here, who listens to me.

I: Hmm hmm- how do you manage that with the number of patients though? Even if the vast majority don’t have any illness?

R: See- one second also, I can speak politely. It doesn’t mean I’m providing one second to a patient, I have to shout. If I- present myself politely, the patient will definitely- uh, will be mine. Mentally, he or she will be mine. So, that’s more important. I have seen other thing- that actually helps you to reduce the number of patients this uh…uh…the patients which don’t have any disease, they come back, but on my days of OPD, that is reduced. Because they know- that if I really have a problem, Doctor will definitely take care of me. But if I’m not having any problem and going to him, he will just talk to me and let me go.

I: Mhm mhm mhm- what are your thoughts on delayed prescription practice?

R: Delayed prescription…I’m not getting the concept.

I: Uh, just basically, waiting till you’re absolutely sure before-

R: Oh before prescribing the thing? Yeah, that should be the original mode of treatment, but here at Raniganj, you cannot afford to do that. The patients will not allow you to do that. OK? See, in treatment, there is always the risk of getting something wrong. OK? If you are delaying with the prescription, you are waiting for the reports to come. The patient party will say- the doctor did not do treatment for us. If something wrong happens. So, if you cannot give anything, just give a Vitamin B complex or a Pantoprazole, to make them happy that we are doing something. Many a times, it happened- instead of tetanus toxoid, I pushed distilled water to the patient, because I know there is no use of a toxoid to the patient. But they are forcing that you have to give Tetanus. So I pushed distilled water, so that they get the pain- distilled water IM is very painful- they get the pain, and they know that- yes some medicine went inside, that is why it is hurting. That’s the type of treatment sometimes we have to perform.

I: Placebo sort of-

R: Yes yes placebo sort of things.

I: OK, OK. But what happens, say, if you know that an antibiotic is not required immediately, but a patient lives far away and you’re unsure of follow up? What would you do in that case?

R: Uh, we try to refer it to somewhere, where the setup is there, and I personally do- I write out the prescription in a way, give it to the patient, and tell them to show it to any doctor they go and see. What is my thought process? That is also important for the doctor to know, because the first examination, I made it. The doctor is seeing after few hours, or after few days. He or she must know what was the first symptom the patient appeared with. So I do that- I write out the detailed description of the whole case, I found out the history and my examination- everything- and I then asked the patient to go. It’s quite often- many a times, for these RTI cases only, we have to do it. Like, the highway is just beside. So person coming from Purulia, or Jharkhand, or anywhere else, meets some accident over here- definitely, he or she won’t wait here for 7-10 days for my follow up. So, initial treatment I give. I try to give it in the best way, so that the next doctor does not have to interfere on it anymore, but I write the detailed history and my work, everything. So it’s fortunate- most of the time, the doctors from there gives me a call and praises me that you know, it’s a good thing, thank you, you work really well. A patient few days back came up, he came from Bodhpur, he had an accident at Mangalpur over here. So he said the doctor he showed over there, he praised about the government setup that in such a small government hospital, such big things are done? He had an accident and this particular thing went off [*pointing to the lip*]. The lip part? Yeah, only one side of the lip was attached. Rest was hanging. So I made a surgery over here within my capacity and I let him go. I asked him that it’s a request to please not touch the stitches. Tell any doctor not to touch it. Wait 7 days and then do what you want. So he went off well- he went to a doctor, definitely his house physician, his surgeon, whoever- so that person was full of praise about the government setup. So that’s an achievement. Anyway-

I: What do you- how do you counsel patients who self-medicate with over-the-counter medicines?

R: First of all, well I shout at them. [*chuckling*] That’s a common scenario, and my patients are so loyal to me- even if I shout at them, they won’t mind. Because their concept is doctor sir is so polite, if he scolds me once in a while, it must be for my own good. So, then I try to correct the situation if it’s possible.

I: Ok, Ok. How do you weigh the risks and benefits of prescribing antibiotics? Can you describe the process a bit?

R: Uh, definitely as I said, initially a sulfa-allopathic drug without a side effect is not a [*inaudible*] drug, so always I have to keep in mind the side effects of it, but again for better benefit of the individual, we have to select something which is, even riskier, we have to select that. So, during that, I keep in mind the adverse effects, but I keep in mind the benefits first. So if I find it is much more beneficial than it is harmful, I go for it. Because the harm, later on we can tackle.

I: OK. So, coming to drug resistance a little bit, so how often do you encounter patients with antibiotic resistance?

R: This major part [*pointing to a map*] the neighbour part of Raniganj, the neighbour district of Raniganj rather- uh, 70-80% of the patients are resistant to antibiotics.

I: To first line antibiotics? They are coming here?

R: Yes, the first line antibiotics. No, no, no- I actually go there for a chamber [*private practice*] once a week. So there, I was shocked to see the type of medications they have from the medicine shops and all.

I: Like?

R: Like, uh, the first line- penicillin! – the most empiricial antibiotic we go for infections commonly, so that’s there highly resistant- a patient came to me with dental infection. I’m basically a dental surgeon. Then, I perform head-neck surgery, because I’m trained with it, I’m trying for the post-grads and all. So…she had a dental infection, a severe dental infection with a, with a abscess formation in the buccal space and all. So, I prescribed 7 days Amoxi-Clav, with TDS- after seven days, she came back to me with the same swelling, same pain, and same symptoms.

I: My god.

R: Then, I took out the pus from there, I sent for the culture. I said now for 3-4 days, no drugs, nothing. Just stay on a painkiller. Then result came that Cefuroxim was perfectly sensitive to that patient. I then prescribed Cefuroxim 500 and then, uh, she recovered from it. So I was stunned to see after even 7 days of antibiotic therapy with regular dosing, the patient did not respond a single percent. So we encounter quite often over there. Here also, there are few [instances of] antibiotic resistance, but actually the thing is, mostly in government setup, doctors use broad-spectrum Clotrimoxazole. So, that is known for least resistance.

I: Least resistance?

R: Least resistance. Clotrimoxazole Septra DS- that creates least resistance. So that’s the good thing about the drug. And the health services are alive because of that particular drug itself. OK? Anything and everything starting from cholera to…everything you can think upon, start with Septra DS, until and unless you are sure with the drug what to give. You’ll find yeah it is responding, initially it is responding.

I: So what do you think drives antibiotic resistance? What are the different factors?

R: First of all, well, irrational use of antibiotics- self-medications. Secondly, irrational use of antibiotics by us [doctors]. The iatrogenic cause- definitely the iatrogenic cause is a major cause in India specially. Because most of us do not even know the dose of empirical antibiotics. That’s a…huge issue of concern. That’s why I’m ready to give you this interview, because yesterday only I was talking- that we should know- I tried at Raniganj itself to conduct seminars regarding that, then I found that all the doctors will take it in ego. Anyway, they have this feeling now that the past 5.5 years, their patient load has decreased- after I have come here, I’m pulling their patients in, in the government setup. So nowadays, many patients are coming from good families, they are having good financial backup. They are also availing the hospital. So they [other doctors] are a bit angry over me. Now, if I conduct these type of seminars letting them know of antibiotic use, they will kill me off [*chuckling*]. So yesterday, I found a good platform to share my views over antibiotic therapies. So, this irrational use of antibiotics- doctors are also creating resistance definitely- and third, the most important thing is our modernization.

I: Meaning?

R: Our lifestyle is getting modern every single day. We are using much more gadgets, we are using much more foods which are refined foods, rather than the original empirical foods, so we are lacking in our immunity also to fight out the infections. That’s why you are using antibiotics…more often. And this is helping the bacteria to change their own types. So, this lifestyle is also a big issue, modernization is a big issue for this antibiotic resistance.

I: OK, OK. Doctor, tell me something- so you have your doctors, nurses, pharmacists, RMPs- this whole dynamic, right, of people who interact with antibiotics. How does that dynamic affect antibiotic resistance? I mean, the dynamic between these-

R: [*interrupting*] Highly, highly, highly! This RMPs, this shopkeepers, medicine shopkeepers, sometimes nurses, sometimes seniors from house without having any medical background also, they tend to prescribe antibiotics now and then, and worst thing in India is selling of antibiotics over the counter. You go to a shop, you ask for any antibiotic, they will give. Unfortunately, you go to a shop, you ask for any oral contraceptive pill, they will ask for a prescription. Which is much more required in India! To control population, that is getting with prescription. Without prescription, that is scheduled drug, they are not giving it, but for an antibiotic, they are giving it. Any antibiotic you ask for, they will give you over the counter. So, definitely, this is a big issue. This should be stopped. I mean, without prescriptions, no antibiotics should be given.

I: And, uh, what steps do you think can be taken…to control antibiotic resistance at all the levels we talked about?

R: First of all, DCI/MCI, Council for Medicine and Dental in India, they should be strict about use of antibiotics by the professionals. Because there is no clear-cut guideline- how to use it or not. I have read in my pharmacology, I am accordingly going through it. And my school of thought- my professor- I am driven by my professor actually! Whatever I’m doing today, I’m driven by my professor, his school of thought, which he taught me in my student life. Most of the doctors are going in that way. Above that, few are company-oriented doctors, that I everytime see. Like, uh, after seeing 100 patients a day, he or she doesn’t even bother to look back to the whole day, what type of patients he or she has treated. What is the trend of the disease? What should be treated? Consult with the books- there are e-books available- so much of e-books available, you need not to open the thick book and go over the leaves. You can go to the computer itself- e-books are highly available. But, they…listen to the companies! A company comes and says I’ll give you a…whatsoever I should not say this sitting in the professional chair, but company says- most of the companies, I just fought with a company 2-3 years back with the same situation. That- that’s a renowned company I won’t name. He came and told me that AmoxiClav is sufficient if I give BD. And that’s the biggest issue, which plays in my mind, because Amoxicillin is the empirical drug we can think of! I asked him why…625 BD. So what is the function of 125 mg in AmoxiClav? He doesn’t even know the use of Clavulonic acid, what is the use of Clavulonic acid, but he dictates the doctor- practically dictates the doctor- because a reputed company [*pauses*] over the years, 100 years old multinational company- he has got the right to dictate the doctor! To write it in a BD dose. Many doctors follow that.

I: Uhh, so you mentioned the MRs- can you tell me a little bit about the role they play? How often do you interact with them?

R: Uhh- not much MRs visit me, because I have an easy policy- I’m not a broker for any company. OK? You come to me, definitely your company is selling medicine, my patients require medicine. When and where required, I’ll definitely think of your company, if you come regularly, I’ll have in my mind your company has this product and this is required for the patient not available in the hospital, I’ll write. But if you consider me to be a broke who will write and you’ll be giving me something, you’re wrong. You ask your company to offer me a 300,000 rupee package per month, I’ll leave my job, I’ll work for your company and I’ll write only your prescription. [*chuckling*] So that’s why not much of MRs like me, because I go straight away with the thing, because patients are much more prior to me than anything else. I’m getting paid for that. But, a few are there, who, with whom I’ve got personal relationships, personal relationships like friendships. After coming back to Raniganj, I’m not a boy from here- I’m from…Kolkata, so this was a new place to me initially, so I had a few friends, among them a few are MRs also. They never insist me of writing any medicines. They never bother to show the literature even to me. If I ask something, that this particular composition, do you have in your company? If they say yes, if it is required to the patient, I just write it down. But neither they show me any product, nor I ask them for any product. And that is another reason I’m tending to forget most of the medicines sitting in the government setup. Because I don’t like go through the new, upcoming products to the- in few seminars, I do get upgraded- a few seminars through journals and all- but that doesn’t help in the brand thing.

I: You said about journals, so can you name some journals you follow?

R: Yeah JIME- Journal of Indian Medical Education. That is really a good journal because uh…JIME and JIDA also- the journal for the dental association as well- that gives a very vast knowledge of the…Indian scenario. Like, reading out international journals might upgrade your knowledge, but that will go when you are sitting in the rural area of India and practicing. The disease that we are facing- in US, doctor does not know how to treat malaria, believe me. They know how to give malaria vaccine, but if malaria is infecting a patient, doctors do not know ho to deal with malaria, because there is no malaria there at States. But in India, we know how to deal with it! Yellow Fever we don’t know how to deal with it. I’ve read it once in medicine book, and now I’ve forgotten it, because- never saw it. But in States, doctors know how to deal with Yellow Fever, because they work with it. So if I read too much of international journals, that will upgrade my knowledge, I can give you 10 names of international journals, you will know oh wow look at what kind of doctor he is, see how upgraded he is. But that won’t help me in treating my patients. So Indian journals, uh, and I don’t- I’m not a book worm, so I read as much as I require, so JIME and JIDA are sufficient for me, along with the- sometimes, few journals come with the medical companies- they come and give to you, some journals. So whatever I get, I see one time what upgradation there is or not. Then, there are seminars and conferences, that helps us.

I: What are your attitudes with respect to continued medical education, especially in relation to antibiotic resistance?

R: It’s very important! Very important! This is very important, not only for antibiotic resistance, but also if you can touch other topics, specially the basic topics. Because uh, in the field of practice, most of us tend to forget the basic things, okay? We, if you ask a doctor- can you tell me what is a nerve? I bet 90% cannot tell you. He or she can explain you, but he or she cannot define you what is a nerve, because we don’t deal with a nerve, we deal with a neurological disorder. If you ask a patient what is a stomach, what is the build of a stomach, he or she won’t be able to say, but you ask for a GI tract infection or GI tract complication, he will give you 5 page of description. So the basic things we need to revise every moment, so that we get upgraded. Until and unless your basics are clear, your treatment is all in vain. So, if I’m not clear with my base, I’ll write AmoxiClav in a…BD dose twice a day. If I’m clear with my course, I’ll write it thrice a day. So that’s the thing- continued medical education is really important. And definitely antibiotic use well- there should be a protocol and guideline for antibiotic use. That’s a major issue India is facing now.

I: Are you familiar with any government guidelines on antibiotic use?

R: There is no government guideline on antibiotic use. As far as my knowledge goes, there is no government guideline.

I: Hmm hmm hmm- have you attended any CME programs?

R: Yeah, since my college days, I have been attending CME programs.

I: Regularly?

R: I have done, yeah, ACA uh- this BLS and ACLS at least 10 times. So, people ask me why you’re going for Basic Life Support and Acute…life support, why you’re going for it? So I tell them that basic life support, the concept is changing every single year. So, if I’m not upgraded with it- I work in a surgical field- forget about my work, my field, I go out to the world, I go out to the society. If a patient in front of me goes in syncopes [*fainting*], so at least if I know the thing, I can save that patient at that particular point of time. So getting upgraded with it, and BLS is one of my favourite topic. So whenever I find a BLS course, I tend to go there, along with other topics also, I go, I attend this, uh, CME for pain management and all. Pain management is a huge topic now. And definitely antibiotic therapy, if there is a CME anywhere, I try to attend. And the disease process in a surgical CME, surgical workshops- surgical workshops, especially head-neck region, I go, because others are not in my domain. At Raniganj, I have to work because there is no other person to work in the surgical field. No one is oriented in that way. So, tip to toe, I do. But, beyond that, my capacity is head-neck. Restricted to there. So, I try to attend those conferences- those seminars.

I: What strategies have been tried to combat AMR- anti-microbial resistance?

R: Strategies adopted by whom- me or the society?

I: No, society at large.

R: How do I know? Nobody is taking up any strategy, but I found you- you are taking a strategy to combat this, this is a very good thing. Go ahead, I’m there with you.

I: Okay, that’s great! That’s great! [*chuckling*] Do you have any suggestions or recommendations for how we can tackle it at a local level and a national level?

R: Without hurting the doctors’ ego, the clinicians’ and practitioners’ ego, try to give them CMEs on the use of antibiotics, without letting them know that you don’t know nothing or you’ve forgotten the thing. Try to…involve them in a way, if…I hammer you something every day, if I tell you something every day, one day or the other, you tend to follow that. So, if there are repeated CMEs, repeated journals on use of antibiotic and all, definitely one day or the another, I’ll listen to it, I’ll keep that in mind and do accordingly. There should be a guideline and all. First of all, this thing- second thing, there should be clear guideline, protocol, by the MCI and DCI to work on it, to make it an effective step. Otherwise, no one will listen to it. And third is…to tackle the population and make them aware of the thing. That is another important thing. If I know antibiotic abuse is bad, I won’t go to a medicine shop and ask for antibiotic, or even if he or she provides me with antibiotic, I won’t take it without consulting a doctor. So you cannot stop medicine stores giving antibiotics, because they will do their business. That’s their own nature. But we cannot- we can stop the population by making them afraid of the thing, making them understand about the situation- what can happen. So, that is a huge issue- and that only you people can do it. It is difficult for us to reach there. Because yeah, I know I meet 400 person per day in my OPD, but I get very less time to make them aware of the fact. But when you face 400 person standing in a platform, you get time to make aware those 400 people at a time. So, you can conduct CMEs at…different areas also- community also- community awareness programs can be conducted. So that’s my- actually, I had a NGO initially to work on these things- and later on, I found that it’s nothing but- in India, if you want to work with an NGO, or you have an NGO, you have to have political power, and then, bad mentality to earn. Because my mentality was to…devote things for the marginalized people. But the society did not allow me to work. I was working on this particular things itself.

I: Where was this?

R: It is in Kolkata. The registration is in Kolkata, but we worked at the Sunderbans, we worked at [*inaudible*], where it is required actually. We found out few places, and then we went there- within Kolkata also, there are few slum areas. There also we worked. But, then I found that no, a huge political pressure is coming and then we lack funds- the main problem beyond stopping that is we lacked fund. Because, working on this, creating a awareness program or holding up a awareness program in a single day, you, if you go and ask a slum area people that tomorrow you, 400 people come and I’ll tell you- nobody will come, until and unless you show them something profitable to them. You give a food packet, you give a t-shirt, anything- they will come for that. Rest, you have to force it into their mind. So, it got stopped. That was uh, very…bad for me. I felt really very bad. Three of us, three of my friends, started that. So fine, you are now doing it- I’m really very happy. And I’m with you. I know I have a platform now to work on it.

I: Well, thank you so much, doctor. We really appreciate it.

R: Thank you!

1. From Quack
2. Here it implies GOD.
3. Referring MSF
4. Referring Patient
