# Supplementary material for: “Without antibiotics, I cannot treat”: A qualitative study of antibiotic use in Paschim Bardhaman district of West Bengal, India
Source: PLoS One. 2019 Jun 27;14(6):e0219002. doi: 10.1371/journal.pone.0219002 (PMC6597109; doi:10.1371/journal.pone.0219002)
Supplement: S2 File — (ZIP) [file pone.0219002.s002.zip › S2_Transcripts/KAP 260.doc]

**KAP 260**

Gender: Male

Age: 31

Occupation: allopathic doctor

Highest education: DNB paediatrics

I: So doctor can you tell us a little bit about yourself? Sort of… your educational background and how long you’ve been here?

R: Actually, I’m a…paediatrician. I’ve been working here for past 3 years...and basically, I am concerned with the pediatric department, and I’m seeing the pediatric patients only.

I: Mhm mhm- only pediatric patients?

R: Only pediatric patients.

I: So what does your typical OPD setting look like?

R: Uhhh—as—about the patients? Like basically the patient coming here are from the poor family, and uh…poor background. That is, they don’t have higher income or they don’t belong to the higher income group. They come here for free treatment, and free medicines, and most of the illnesses that we find here are belonging to the lower socioeconomic group. And-

I: [*interrupting*] so what would that be? What kind of illnesses?

R: Uh- like, basically concerned with malnutrition, and associated diarrhea. Other respiratory illnesses like bronchiolitis, pneumonia, and worm infestations, and tuberculosis of course, and typhoid and other and most of the bacterial infections and those associated with malnutrition and other concerned problems. And we also get other diseases like thalassemia and other diseases also…there, but most of the diseases are problems concerned with malnutrition and…other issues.

I: What is the typical treatment procedure that you follow, like what- can you walk me through a typical conversation with a patient?

R: Sorr-y?

I: So like can we walk me through a typical conversation with a patient? And your treatment decisions?

R: Yeah basically when a patient comes, uhh, it is very hard initially to make them understand about the illnesses. Because they are basically coming from a…poor socioeconomic background and not much of education, soo first of all, it is very tough to make them understand about the…illnesses. First of all. So, it takes a…good amount of time for that. And- once that is done, we have standard protocols for most of the disease-s…from the government’s side. Which has been set by the India government, and Indian Academy of Pediatrics has also set so may standard protocols. We follow those protocols and we…follow those protocols strictly, and do the…needful for the patient.

I: So, what do those protocols say about antibiotic use?

R: Like, antibiotic use it depends on the diseases. Basically, for diseases like diarrhea, as per the protocol, strictly we don’t have to give any antibiotics. So when a patient comes with diarrhea, we have to treat first the dehydration part, that’s the major- main part, and…uh we do that. By fluids, either if the patient is able to take it orally, or...other means. We give the fluids and rehydrate the patient as much as possible, and antibiotic has no role in…diseases like diarrhea. And other issues like…bronchiolitis, which is a viral infection, also doesn’t need antibiotic. And only those diseases like pneumonia, and other bacterial infections…antibiotics are needed. And for viral illnesses, antibiotics are not needed at all. And as per the protocol also, strictly, we don’t have to give uh…antibiotics [*quieter voice*].

I: Now, in the survey questionnaire, you were mentioning something about when- it’s not necessary that viral infections do not always not need antibiotics. Can you explain that a little bit more in depth?

R: Yeah, because viral infections if you see in an OPD setting, the patient comes to…the, I mean the first…uh, place you see the patient and you don’t give any antibiotic, you just gave, you treat it for a viral infection, but you need to get the patient on a follow-up after third or fourth day. But most of the patient won’t come back to you after that third or fourth day. Because of the lack of knowledge, or uh….they are influenced by some other…issues. They go to some other practitioners, or…they are missed, actually. And uh, if you take 10 patients, only about 2 patients will be coming back to you for a proper follow-up. So, in that case, you are missing lot of things like you don’t know whether the patient is responding or patient is getting well. You have no clue on that, so…that’s a big disadvantage. And we see the OPD crowd is so much…that…patient gets lost, and proper follow up is also not there. And…even if you say patient strictly to come back on third or fourth day, they don’t turn out, so…they go to another place, or another hospital, or another private practitioner, and in between, they may show another doctor, and he may give an antibiotic. And patient may come back- some patient come back again after a week, saying that…uh…hiding those treatment. And they’ll say that they’ve not taken anything, so…uh, you won’t get a proper…history of that patient also. So…in that place, in that case, we are getting a lot of handicap that way. And about the lab support also there are so many lacunaes here, so uh if we have a proper…lab support uh, it will be better like 24 hours around the clock lab support, and other things. And it will be more easy for us to decide on whether to…um…give or not. But basically, as per the protocol, and I personally also feel that…there is no need for giving antibiotic for…uh, in viral illnesses like flu. For that, uh, we need a proper follow-up and a proper lab support.

I: So uh, you mentioned lab support. What is the availability of microbiological testing…right now?

R: Uhhh, right now, we have…uh, we don’t have…cultures per se. It’s better to…easy to say what is not there. So, we don’t have culture-s are not available here, and uh, first of all 24 hour lab support is not there. It’s limited time, and around the clock it is not available. And um, means….some things…may take time also, maybe due to uh…proper…uh…resources are not available. Like, uh…people, as well as other resources, are not available. Because of that also, some gets delayed, and that also affects the treatment, like if a test is delayed, that also is a big problem. Like, and basically the other…lab uhh, like cultures are not done here. Only things are done are, uhh, counts- total counts, then…uh…and urine. Urine also culture is not done which is a basic need…for…pediatric practice is concerned. It is not done. Then…sputum, nothing is done. And…so basically, there are a lot of limitations here at present.

I: So, sens-sensitivity?

R: Not done.

I: Not done. If you really want to do some test, like suppose if you apply for private, from outside, patient will agree to do or is there- there is any proper opportunity that present-

R: Like uh [*interrupting*] uhh as I said, most of the patients are belonging to the lower socioeconomic group, and uh…at least if you take 10 patients, only 2 or 3 may allow, or maybe be really willing to do the test from outside. That is around 30% around they’ll be doing from outside, and you cannot also compel the patient so much to do from the private, that is not…uh done here also, because that should not be done, because you can’t compel the patient to do from outside.

I: What’s the patient workload in OPD?

R: Per day, we get around 180 to 190 patients.

I: And you’re working in the IPD as well, so…IPD?

R: Yeah. Uh…IPD there is about 30 to 40 admissions.

I: Can you tell me the time per consultation in OPD setup? Like, suppose 100 patients in a day, so how much time you are getting for per patient?

R: Like actually this is a government setup so the crowd is…huge. They come for, uh, free treatment of course, so crowd load is too much. So per patient, an average you get only 3-4 minutes. Actually, that’s not enough.

I: you’re working alone or you’re working together with any other doctors?

R: Other doctors are also there.

I: Also there?

R: At least three doctors will be there...umm, in pediatric OPD.

I: So did you ever discuss with other doctors…in terms of prescribing antibiotics or any…a discussion is going on there- ?

R: [*interrupting*] In OPD setup, uhhh, due to lack of time and due to other issues, during the consultation time…

I: [interrupting] It’s not possible.

R: [continuing] It’s not possible. We discuss it after once the consultation or this is over, we get to…discuss with other uh- some of the main cases we take and we discuss. [*mumbling*] otherwise it’s…

I: mhm mhm mhm. So what would you describe your prescription patterns like? What are they like?

R: [*long pause…looking unsure*] Like…?

I: More- typical medicines given…sort of, you know, what kinds of medicines you are prescribing?

R: Like, as far as we are concerned, we have…to give free medicines as I said. So, patients can’t…most of the patients can’t afford from outside. But we have support from fair price shop, which gives some of the medicines uh…for…relaxed prices…but even those…have some…limitations, because only those medicines available in the hospital pharmacy or fair price shop we can give, because…uh other…medicine patient will have to buy from outside, so we can’t- most of the patient won’t buy, and they will not take also, so we have limitation in that also, like…umm, most of the medicines are available, but at times, due to lack of stock, or uh…lack of this thing, lack of availability also, we are forced to give them limited medications.

I: In the medications means, do you have a preference for generic names vs. brand names?

R: Yeah…at present, the…doctors are only allowed to write the…uh, generic names. We are not…bound to write the brand names, because legally it is- not…[*trailing off*]

I: Okay- why is that the case?

R: Why is that the case? [*snickering*] actually, that should be asked with the authorities, because uhhh basically I am of the opinion that uhh, this issue comes because of the price difference of course. Because…brand uh medicines are of huge price compared to the generic medicines, and…actually uh…my personal opinion is that it should not be happening in a country because two types of medicines. Government should take…and authorities should take actions for that. [*very confident*] Basically. Rather than compelling the doctor to take generic medicine, it should be on the other way around that uh medicines should be…priced…in an…like, all the medicines should have a price control by the government. And government should decide which price and [*chuckling*] that should be done and it should be affordable for the community and people also. That should be done from the authority’s side, and uh…[*chuckling*] doctors should not be blamed for that also. He should be free to write any medicine as far as I feel like because…the quality control or the price control, it doesn’t depend upon the doctor, it’s purely on the authority’s side or the government’s side. So, government should take note of that…they should make a…useful thing on that.

I: Is there a difference in quality between generic and uh brand name…at all?

R: Uhhh…basically, uhhh…as far as I know…umm…[*looking unsure*] most of the patient as I said, we don’t have a proper follow-up, so we can’t strictly say that…this is right, or this is having a good…umm, that is branded medicines are having good effect, and generic is not having good effect. That we can’t say. But, I say that it should be done in a proper setting lab, like and uhh the pharmacy control unit or the authority should take a note on that, whether it is useful, whether it is effective, whether it is…not, doctor should not- doctor should be free to write the medicine, and he should be free, and he should be concentrating on the treatment side, and other issues of effect of the drug, that should be done by research side, and uh…pharmacy side or any other control drugs side. It should not be in the hands of doctors. If umm…that’s it.

I: mhm mhm mhm. You were saying earlier about problems that doctors face…uh, in the community, getting blamed for certain things. Can you tell me a little bit about that?

R: Community…the doctors face most of the problem is, the crowd…that…uhhhh lot of people are coming, lot of crowd are coming, so the load…the workload is the main problem, and if you have to give…a proper treatment, and a proper consultation for a patient, it takes at least 20 to 25 minutes for a patient…and on that hand, we are doing it for…3 minutes to 4 minutes, means obviously it will affect the quality of service also. So…more doctors should be available, and uhh…we have to sort out some other way to combat this. India is already a populated country, so, and uhh…the…settings available in government setup is also very limited, so…there are issues with that.

I: Mhm mhm- did you ever face such a problem like in your life, in your practice?

R: Regarding?

I: Regarding the security ethics.

R: Yes, security things…a lot of time I’ve also had issues…because doctors are…now getting beaten up everywhere, each and every place, so umm…you don’t have proper security for a doctor because any person, or anyone can come and beat you at any time. And, uh…as…the days progresses, even the authority’s side or proper concerned authorities are also not giving much proper security for the doctors. Because...actually one thing is uh…one of the fault is also with media and other side also. They…create such a notion, or they make a big picture like doctors are someone who is looting the patient, or they are only for earning money from the patient, they are extracting so much from the patient, and not giving- such an illusion is created all over the country. The patient is also getting debated and they think like doctors are only for extracting money from the people, rather than giving- but as far as I’m concerned, even if a doctor ge- like charges you- uh, I’m talking about the private service- if a specialist or super specialist may charge you as a consultation fee around 500 to 600 [rupees] in a medium city, and in a…super city, they may charge around 700 rupees. But I don’t say that that is a huuuge consultation fees. Because, mmm…if you go to a beauty parlor or somewhere, for just a simple thing, you may spend around…1000-2000-3000 rupees- without any- like, for, and you say that for a health issue, you can’t even spend like 600-700 rupees, which is very meagre amount actually. Like, as far as other- your daily living cost or something is concerned, even if you go to a Multiplex and see a movie, it will cost around…uhh…200-300 rupees for a single individual, even in a minimal Multiplex, so…for your health, if you can’t spend around 600 rupees, that is- I don’t think that is- not too much. And doctors are actually…charging for the…brain, for the knowledge they are imparting. Patient thinks that they are simply sitting there and writing on a piece of paper and just extracting money. Actually, it’s not the case. They have to diagnose, and for diagnosing it takes so much skill that is earned through years. To become a super specialist doctor, it takes around 6+3+2, at least minimum of…

I: 11 years.

R: [*continuing*] 12 to 13 years, it takes…to become a super specialist doctor. And…that is the time he has earned all his skills, his knowledge, everthing so, for that 13 years, only he is…charging this much, so patient doesn’t think. Patient thinks that only for that 15 minutes, or 20 minutes, he is charging around that much. But actually, the case is he is charging for those skills that he has earned. And, in any other profession, like, even if we take- I don’t blame about the profession, they have to charge I’m saying, even if it is a advocate, lawyer, or any other profession, like engineer, or any other profession. They also charges. Some of the super advocates and biggest setup- they charge around lakhs, 15-20 lakhs for 5-10 minutes. Not telling it’s bad- they have to charge, because it’s their skill they are charging. So, and nobody has no problem with that. And actually it should not be, but that’s the case. That’s for the skill that they have earned through years. For that they are charging, and…when it comes to doctors, it turns everything down. Even the superstars who have been blaming these doctors through shows and through other things, they create a illusion or such a big drama that doctors are only for looting. Actually, that’s not the case. And their charges…crores…for just an appearance of about…3 hours. Actually, they are not charging for that three hours. They are charging for those experience that they have earned and their acting skills that they have been acquiring through…years. That’s what they are charging. Actually, they are also…doing their job. And, people doesn’t have- don’t have any problem for that- how much they are charging and instead, they create an illusion that doctors are…looting them. But, actually, that’s not the case. It should be- a patient should be- made aware of that, and…[*nervous laughter*]

I: Yeah, hmm, so this level of trust- can you tell me a little bit more about how, if at all, it affects your prescription patterns?

R: Yeahhh, level of trust means- see, if a patient comes to me and, with a viral infection, and I don’t…uhhh…basically, I strongly disagree on prescribing antibiotic to that patient, and I don’t like to prescribe also – for such illnesses…an antibiotic. So but, the patient…is made…uh, to believe…uh, that antibiotics only cures the disease. It’s uh…patients are made, forced [*emphasis added*] to believe actually- because they are getting so much inputs from…media. Uhh some of the programs in the television, some of the big screen programs, and through other people in and around the society like other non-medical people, basically, like- uhh- those concerned the…uh medicals, but actually they are non-medicos. Like, I don’t want to specify the job of the people- such people…discuss with the patients about the things, and patients are made to believe- that only antibiotics can cure diseases. And they are of the- and basically, they are made to think and they are made to believe- even- at- it happens, not only in the uneducated or uhhh lower socioeconomic people, but with the…higher groups…also. Such a notion is…uh, made that only antibiotics can cure umm diseases. That may be due to the increased influence of ads, or…these influence of other people who have been discussing, like some of the patients may discuss with their family, and the family members say the- uhh my…hmm…patients were having such a disease and it was cured immediately after giving an antibiotic. Maybe that disease is another disease and which may require an antibiotic. But, [*laughter*]

I: So, in this case, do you give an antibiotic then- with this…?

R: No basically I don’t prefer to give, even if how much pressure I have, I don’t give antibiotic, even if I lose the patient, I don’t mind because basically practice is one that you get…over the years. It is not in single patient or- you have to build the trust and it takes time, also, like patient may compel you also so much, but it’s uh…better not to give the antibiotic because…instead, I believe that even if you don’t do any good, don’t do any harm to the patient.

I: Mhm mhm mhm. Yeah I understand your personal opinion, but do you think that that demand for antibiotic- that’s one of the factor for more antibiotic resistance?

R: [*starts talking before interviewer is finished*] Yeahhhh- maybe for uhh upcoming doctors and new doctors, that may be a little pressure…to give antibiotics because of the pressure from the doctors.

I: And you previously mentioned that patients not come back to you, or whether they are not followed properly, so how many patients are- come back to you, like out of 10, like- ?

R: [*interrupting*] Like I said…previously also, out of 10, only…3 or 4…basically, that is around 30-40%.

I: So, did you ever ask whether they take the full course of antibiotics or uhh- what their feedback?

R: Uhh- most of the patients in that 40% also they don’t take the course of medicine prescribed or actually advised to them. Even if you take time and advise them, they don’t follow- your advice properly [*chuckling*]. Maybe, uh, that should be the first thing that should be done- education of the patient. Basically uhh...[*trailing off*]

I: So, in this case, do you prefer to give a full course of antibiotics right when you see the patient, or…you will call back after 2 days or 3 days or…?

R: No, if I start an antibiotic…at present, I will give the full course.

I: Full course.

R: Rather than giving half course or something. I [*inaudible*] give the full course at present, but I don’t know about the research studies that’s going on…and some researches are going on…at present, which…is bringing out some results concerning like there is no need to give full course of antibiotics. But as far as I’m concerned, as far as I’m concerned, at present, there is no published things yet, which tells you that you should not give, or there is no protocols at present which tells you that you should not give half course of antibiotics.

I: So you previously mentioned that there is…the limited facilities available for the test and all. So suppose the patients come back to you- like you already prescribed antibiotics in the first visit, and the patient come back to you after 5 days. And it’s not cured. So what are the steps…what are the things you do for the second time?

R: So…after giving an antibiotic?

I: Yeah. You already…prescribed for one full course of antibiotic, but patient not cured. Patient come back to you again…after 5 days. So what’s your steps?

R: I do detailing…in- vestigations. And with a limited lab support…available, we do detailed work up of the patient, and…based on the results only, we look for further procedures [*trailing off*].

I: Further procedures. Mhm mhm…

I want to come back to drugs for a brief minute. So, do you prefer monotherapy or combination drugs and why or why not?

R: Uh…basically, I prefer monotherapy. Erm…uh…polytherapy is not needed at the present, and we don’t have any supporting evidence uh…with lab support or something to go for a polytherapy at present, so we would…go with a monotherapy and look whether a patient is responding or not, and how the patient’s response is…happening, and….we will stick to that. And with those therapies, and from this year’s even we have to come know about the basic disease pattern and basic problems- how the things respond in our setup. So each and every question we know about the things- I am talking this because we don’t have a proper laboratory setup, or backup at present…so we stick to monotherapy and see the response of the patient.

I: What are examples of combination drugs that are on the market?

R: Combination as far as…uh, this thing…[*pause*] um drugs, combination drugs…so many combination drugs are available, like them, so many uh…which is undesirable combinations are also available. So I mean they mix 2-3 antibiotics, which are not needed actually, which can be cured by single also. But I don’t look into that…I don’t want to- comment also on that.

I: Okay…what about the availability and use of broad-spectrum antibiotics?

R:…availability?

I: and use. Of broad-spectrum antibiotics. In what circumstances are broad-spectrum antibiotics indicated?

R: [*confidently*] Broad-spectrum antibiotics- basically, our setup is a primary setup, so initially, we get the patient who comes to us in the first instance itself. So, basically we will be the persons who will be prescribing the first treatment, so for us, broad-spectrum antibiotics for us…are…not much needed. Because…we treat with- starting, as per the protocol also, we start with the uh...basic drugs, antibiotics, and then we go for broad spectrum and up. But, at times, in cases we get some…which have been treated from outside, how- which is not having much response, and coming to us. Or, they come with a valid sensitivity report, which requires a broad-spectrum…uh, thing, and other things are…when the patient is immunocompromised, or in other uh circumstances, they may need broad spectrum antibiotic.

I: How often you are seeing the skin diseases or skin problems? What’s your prescription pattern when you are prescribing for them?

R: Basically, most of the skin…uh, diseases that we see, it’d be either scabies- as far as pediatric patients are concerned, it’d be either scabies or staphylococcal infections or **pyroderma** or something like that. So, for scabies it’s basically, treatment for scabies- no antibiotic- and we treat for scabies. And…for other staphylococcal infections, of course we have to prescribe Amoxicillin or Co-Amoxiclav for staphylococcal infections.

I: What about topical antibiotics? Did you ever prescribe- ?

R: [*interrupting*] Yeah. Topical antibiotics we are also prescribing.

I: Such as?

R: Such as Nepirosin, or something.

I: And all drugs are available here in the - ?

R: Yeah. All such basic drugs are available in here.

I: Do you perform minor surgical procedures at all?

R: [*quickly*] No. Basically, I am not a surgeon, so…surgical procedures are performed from the surgery side, so-

I: When- when they are performed, I mean, do you know if antibiotics are typically indicated for minor surgical procedures?

R: That’s…we don’t perform…so I’m not…

I: So you’re not- that’s okay- totally fine. If I, uh, listed a few common illnesses, can you describe what the typical treatment procedure would be, as per protocol and as per your practice?

R: Ok sure.

I: So, suppose a patient proje- uh- presents with just a cough, cold and runny nose. What would you do? Nothing else.

R: Uh, we will treat for the fever, of course, and for a running nose, by either say…if a child, we will prescribe an anti-histamine. And we’ll cover up for that only, basically for the running nose and fever. Beyond that, we won’t do anything.

I: Okay. If a patient is coming with a fever, then?

R: If a patient is…coming with a fever, uhh…it depends. Like, uh, for small children like uhhh…less than you may say 3 months or small children 2 months. Those children may be having some other illnesses also, which we would require some other investigation to be done. And, we will wait for those results to- basically, in the first instance, we don’t prescribe antibiotic.

I: What about if there’s watery diarrhea, with or without antibiotics?

R: Watery diarrhea, as far as paediatrics is concerned, there is no scope for antibiotics. It’s basically rehydration.

I: Rehydration- so ORS?

R: Yes.

I: Okay. Stomach pain?

R: Stomach pain…uh we look for the cause. Basically, most of the patient who comes here…the cause will be uh…either a worm infestation, or…it can be uh, something like gastritis. These are the most…common things we see here, so we don’t need any antibiotic. If at all we go for uhh….some smaller children may complain with stomach pain for urinary tract infection also so…we screen for that UTI also.

I: Mhm mhm- and you mentioned skin problems earlier, so for rashes, what is the typical treatment?

R: Rashes, as such, there is…umm, we come across most of the diseases of skin that is scabies. So that may be present with uh…a problem, with a rash, and itching. Basically, we treat itching, then we give treatment for scabies. If it is a skin infection with bacterial, like Staphylococcal or Streptococcus, then we will go for topical antibiotic…and treat it accordingly [*trailing off*]. And if we have much more things coming up, then we will consult with our dermatology support…and then we will make it [*trailing off*]

I: Mhm mhm. Uh you already mentioned- did you send the culture before starting the antibiotics?

R: Yeah of course we send the culture before starting the antibiotics.

I: Like in which- blood or what?

R: It depends like if you say urine culture we need, we uh…do a urine culture. If it is a bacterial culture, system infection, we do blood culture.

I: [*interrupting*] But where you send like- ?

R: We send to private. Again, that’s an issue. Like most of our patients won’t be willing to-

I: So this you will do for all patients before starting the antibiotics, or or—some specific?

R: No it’s specific patients, because of the uh lack of infrastructure and lack of support that we have, we cannot, uh, compel all the patients at present. Because actually it should be done for all the patients, but we cannot do at present because of the lack of [*trailing off*].

I: What are common antibiotics used in this- used in your daily practice?

R: For my? For-

I: Yeah. For your prescribe-uh-

R: OPD basis?

I: OPD basis.

R: OPD basis, common antibiotics prescribed are Amoxicillin, CoAmoxiclav, then Azithromycin, Cefrodoxim, then Cotramoxisol, Trimotheperim Cotramoxisol, then…uhhh Azithromycin of course. Then…Nitroferentine, then…Cefaxim, Cefuroxim.

I: Okay- so what’s the percentage? Like suppose out of 10 patient, uh, like uh for how many patients you are giving antibiotics on a daily basis? Average.

R: Out of 10, we may give 4…3-4 patients.

I: 3-4 patients. Uh…how do you treat upper RTIs normally?

R: Uhh…RTI we treat…again, it depends on the age group- basically. Like um, a smaller child who is uh like months old…or 2-3 months, uh, we have, we don’t have that back up or support. Actually, we should basically be like to investigate those patient more. And because small children may get other infections, coinfections, very easily. So we would like to have a…thorough investigation before giving antibiotics, but umm…we are, at present, lacking in that. So, for very small children…basically- we’ll start with an antibiotic. But for…uhhh, older age groups…and, as far as I’m concerned, basically for all children, you should wait- and see whether it is responding or not. And then go for an antibiotic, but most of the things we can’t do as per the this thing.

I: This thing being the? Protocol, or?

R: Uh- because of the lack of support. Other sup- yeah.

I: Okay. [*pause*] So what are the factors, comes to your mind, before- uh- before prescribing an antibiotic?

R: Uh the first thing basically is I see is uhh- whether it is needed or not for the child. Whether such child really needs it…or not. So, then, we have to assess the child clinically. Completely. They we may, we’ll get an idea. And then we look into the protocol. Whether it is needed or not. And we should stick to that protocol…strictly whether it is needed or not. And, as my guide said, whenever you have a doubt, you think whether it is your child or not. What you will do? Then, you will get the answer that…will be right.

I: And from patient’s side? Like suppose the patient who comes from far or any other things that comes to your mind?

R: No I don’t…uhh think about what, whether the patient comes from far or near, or whether he will not consult-

I: [*interrupting*] Patient demands.

R: [*continuing*] Yes, he will consult me again or not- what he demands. That, and all issues I do not consider at all, because basically we are treating the patient. Basically…patient should get cured. We don’t see about the other factors- I don’t see about the other factors, whether they uh demand- if they say, if they demand more, I say this is the treatment I know, and which I will do. If you want, you can consult another doctor. You are free to consult…I have no, uh, issues with that.

I: Mhm mhm –do you think cost is a factor. Like suppose if you write- if there is a prescript- not available here in any and you prescribe from outside so-

R: [*interrupting*] Yeah as far as we are concerned, cost is of course a factor because most of the patients belong to the lower group, so they may not- even if you write- they will carry those um…paper with them, and they will roam around. They will not take the medicine. So, cost is of course an issue.

I: Do you frequently encounter patients who save the strips and- for later use?

R: Yeah…[*chuckling*] even though they belong to uh very low group, most of the patients, around 90% of the patient, carry the previous slips, and almost all the...things which they have shown, at least 5 years to 6 years back, all the papers they have with them. And almost all the doctor’s prescriptions- everything they keep in a big file and they bring it. [*chuckling*] I don’t know why- in a big cover they bring it- most of the patients.

I: How much time- you are using to counsel the patient? Like in OPD basis, I know it’s very difficult. Means both scenario- OPD and IPD.

R: Like, I use at least… 5 minutes to consult a patient. And it goes along with the…[*pause*]

I: Consultation.

R: …consultation, everything should go together, but at least I make sure that everything I say, but actually…after counselling, you have to re-check the patient, whether they are understanding, whether they are able to reproduce what you have said. There are so many…uh, methods to check that also, so you have to make sure that they have understood. But there is limited time for us to make sure that they have understand. But uh basically…I do counsel them, but the other point like we checking and making them strong with…what they have understood or not, that is…not possible.

I: So suppose if you prescribe antibiotic to one patient, so what are- can you explain the main 3-4 points you’re explaining to the patient?

R: Uh, main, I basically I try to make them understand that first of all, why they are taking this antibiotic. What is the need for this antibiotic. And uh, they have to take the full course. They have to- what is the mode of taking. And the quantity that is also important- how much they should take. Most of the patient don’t have…or the lab or…I mean, the pharmacy won’t supply a proper measuring, uh…equipment. Whether a filler or…uh whether a…this...measuring scoop they are not supplying, so patients are basically depending on spoon. This much spoon, half spoon, full spoon, and…first of all, I say that they should not do that. You should have a proper measuring equipment for that.

I: And especially in pediatric, this is very important…

R: Very important as far as way- where treatment is concerned, you have to be strict with the dosage, so-

I: Proper use- dosages-?

R: Proper dosages. Use of the antibiotic. And some of the antibiotics…can have some…side-effects or other effects. That also you have to make them understand. And, I will also tell them to come back for follow-up after…taking the antibiotic, full course.

I: Mhm mhm- how do you- how frequently do you come across patients who self medicate…with over the counter medications?

R: Actually…I feel like about 98 to 99% of patients are trea-taking over the counter medicines.

I: So what do you tell them in this case?

R: Uh—[*chuckling*] there is not much to say. Even if you say- again and again- the same patient will come back to you with- for another disease after taking a medicine. It is very hard to make them understand I don’t know and mostly the thing is- basically again with the ads and other medias going around in the country at present. Because they have so much of advertisement and so much of branded advertisement, patient thinks that those are the things that they should take and they try those things first before coming and- uh- as far as, another factor is the pharmacy side also…are…more into…actually they should not give [*defiantly*]. Why they should give an over-the-counter medicine? If the patient demands to a pharmacy shop also, they should not give. They know they are banned- they should not give. First of all, why they give? [*chuckling incredulously*] That’s the main thing- and so many hands are involved in that- the whole issue with the authority’s side and government’s side they have to take care of that. And…those are- this is one of the issues also- leading to this- uh- drug resistance also. I strongly believe in that.

I: So before we come to drug resistance, you raised an interesting point- of the multiple players in this. So you have the doctor- you have your nurses- you have the uh, pharmacist, you have your uh RMP- so how do all of these people play a role in antibiotic-

R: [*interrupting*] All are really involved in this and they are the people actually who should stop this from occurring. They have a big role to play. Like, if they know their responsibilities well, how to…do their job well, then this can be stopped.

I: So can you be a bit more specific? So, in terms of let’s say pharmacists- and RMPs-

R: [*interrupting*] Pharmacists basically- even if the patient demands for a…antibiotic, they should not give without a prescription. Why should they give without a prescription? They should say that we are not bound to give. Hmm…you should not look…only about the uh…monetary side or money side. You have to be concerned about the patient and the society also. You should have little [some] responsibilities towards the patient and some society so- they should not give. And even if you have, uhh, so much pressure from the patient, RMPs or other practitioners should also not give if it is not indicated. You give only if it is indicated, because ultimately what we are looking for is…cure for the patient. It’s not that- basically, [*chuckling*] money is not everything. People don’t understand that- they believe money is the main thing, but actually, money is not the main thing- they will not recognize now. They will recognize only after living for 40 to 50 years, they will recognize that that was not the main thing. [*mumbling*]

I: So how do you manage the patient come to you- the patient already that they are getting the proper dosage and they can’t afford the treatment and cost- they already self-medicated from the over-the-counter medication or- they already take advice from other uh, local doctors- so in these categories, patient comes to you, then how you are managing?

R: If a patient comes with that, I assess the patient for that moment, like…uhh whether he has come uhhh I don’t worry about whether he has taken or not. I assess the patient at that moment, and whether…he needs, or what is his disease, and if he needs medications or not, I look and asses that patient at that moment, and then I write. I don’t…uh, see for what has happened previously. Assess the patient at that moment, and then, according to that I decide…whether it is truly needed for the patient. If it is needed, then I prescribe.

I: Suppose the patient has already taken half dose of antibiotic, then what’s your next step?

R: If he has taken half dose and come to me, after uh 3-4 days, I see the patient at that moment, whether he is…uhmm…having a problem at that moment. If he is having a real need for antibiotic, then I’ll prescribe a full course for that patient. If he doesn’t need, I don’t prescribe also for that patient. Like, basically…it depends on- the disease of the patient when he comes to me at that moment. According to that.

I: Mhm mhm mhm. How do you weigh the risks and benefits of giving antibiotics? Can you describe that process?

R: It depends on the uh disease- if it is a real umm bacterial infection, umm, which is having life-threatening problems, we have to give antibiotics. And…there are some diseases, bacterial infections, for which you have to give…umm…antibiotics and you cannot omit. For such, you have to…give antibiotics anyway, because other than that, it cannot be cured. So you have to give. And risk and benefits, like, of course, if it is a life threatening condition, you have to give the antibiotic and if it is not, you may not give antibiotic. Depends on that [*murmuring*].

I: Mhm mhm mhm mhm mhm. How frequently do you come across patients who have- who are resistant?

R: [*long pause*] uhhh….basically for checking that, we don’t have support at present, so umm…my practice very rarely with reports from other places they come. Other than that, we don’t have much support here.

I: Mhm mhm mhm- so, what do you think- what are the different factors that drive antibiotic resistance?

R: As I said, so many factors come into play, starting from the doctors [*chuckling*]…like so many are pressurized to give antibiotics, and…people take advice from other non-medical people- patients-

I: Uh huh who do you mean here?

R: Like they may take advice from their family members, or other close assistants or other pharmacy people, or other unqualified…uh practitioners- who are basically not qualified for practicing. Such people. And, uhhh, other group is basically the pharmacy people itself who are giving the antibiotics, and…actually there should be a…strict legal action to block this, the availability of these things…to the patient. That should be blocked in the first instance. Then only, you can do some real thing.

I: mhm mhm- how do you evaluate the infection control facilities here in the- in your working facility?

R: Uhh, to be frank, it’s very poor.

I: In the sense? Can you explain little bit?

R: Like, proper umm, infection control methods are not done actually. Like, basic thing is handwashing and all health staff should be properly trained to do- how the handwashing should be done in the first place. Like what are the steps for handwashing and how…frequently it should be done. Those are not practiced properly like, even seeing, every patient after seeing every patient and before seeing…touching the patient, you have to handwash. And, before giving any IV medication or IV access, you have to do handwashing. Those things are not properly done for each and every patient. That should be the basic thing to start with. Then, mmmhm, after that uh, proper this thing should be done- surveillance should be done. What are the uhh- microorganisms at present in each and every setup like operational theatre or in ward or SNCO or ICO setup, which are the things. That’s also not taken- taking place. And frequent fumigations, and uhh [*chuckling*] those things are also not done…on a regular basis I mean. Uhh, there are, due to lack of infrastructure, or lack of supporting staff or lack of other features, it’s not done I don’t know.

I: Do you think improving the- what effect does improving the public health sort of practices have on antibiotic resistance…if any?

R: Sorry?

I: Wha-what effect do these sort of public health practices, like you’re saying handwashing…infection control, what effect does that have on antibiotic use and resistance?

R: Uhh with that, you can control a lot of diseases, if you properly have these like…measures. And then, most of the diseases will, naturally will come down. So, when the diseases come down, will- the use of antibiotics also will come down. In that way, we won’t be using- we won’t be made to use much of antibiotics, so if you use less of antibiotics, of course your resistance will…come down. So basically, health measures should be improved a lot.

I: And we talked about causes of ABR- what are- of antibiotic resistance- what are some strategies to combat antibiotic resistance- in your view?

R: Like, basically, umm first of all, the availability of free antibiotics to the patient should be stopped…in whichever they get, that should be stopped. Like over the counter medication. There’d be strict legal action…against…um, patient getting those free antibiotics. Like without any, basically they do this…[chuckling] only to cover up the consultation that they give to the doctors, which is basically a meagre amount. That, for saving that, they are doing such a big…uhhh issue to their own health. So basically that should be cut down like getting all that free source of antibiotics, that should be cut down. Then, proper education or umm…regular, frequent counselling- I mean, not counselling- um campaigns against antibiotic or antibiotic usage should be done. That should be on a regular basis initially- it should not be once in a year or twice in a year- it should not be done like that. It should be regular and frequent. And all the fraternities should be made to participate in that…and either by legal action or strict action- they should be made to forcefully participate in that. They should participate in that. All the groups, like health facilities and other people, non-medico people should be strictly made under strict vigilance and they should be- there should be strict rules against- and strict punishment should be made for those who are propagating such things.

I: So I want to push you a little bit on that. So you have your unqualified medical practitioners or rural…registered medical practitioners…sometimes they are servicing areas that are not accessed- at all by, you know, MBBS doctors. They’re far away. So, what role do they play? I mean, what role do they play in you know both antibiotic resistance, but also service to the community? How do you weigh this?

R: Actually…they…prescribe things which are not…umm under any protocol. They do as per their wish. They mix antibiotics, they mix steroid, so lot of drugs they mix. They do it as…without any control. They should be…banned actually, and there should be strict imprisonment and action should be done with them. Authorities should strict…take strict actions against them. They have a big role in this. Like they prescribe…without any…actually they do not have any knowledge about this- drugs basically, so they prescribe left and right- whatever comes to them. And they take prescriptions of…other doctors which have been prescribing- and they don’t know for what it is prescribed, and why it is prescribed. They’re unqualified…people basically, so they do whatever they want. And few such…antibiotics left and right, then it will…of course goes to resistance in the long run.

I: Do you think they have a role in providing first aid…or do you think there is more harm done than- good?

R: [*interrupting*] There is more harm done than…uhh, this thing. Actually, uhh, training can be done, and…they should- not they- that can be given…like some, in uhh, PHCs we have sisters [nurses] who have been treating basic pneumonias…who have been given permission from the government’s side to treat pneumonia before they access uhh…the doctor. So…they see and they uh assess the case, and they can be, when there are so many protocols and guidelines given to those sisters and other people, and there are- in rural areas- sisters who take delivers, who are trained to- such things can be done more frequently, rather than giving it in the hands of these unqualified practitioners- who have no knowledge about- [*trailing off*]

I: Mhm mhm mhm- are there any strategies that have been tried at the national level to combat AMR?

R: Uhh….at present, I don’t have much.

I: Okay. Okay- uh, one thing I wanted to ask you- what are your attitudes toward continued medical education? How useful is it?

R: Yeah it’s really useful- we come to know about most of the recent updates. Whatever recent protocols and so much of things. And once you are into full practice, you get- you don’t have much time for uh- actually reading books, and so…there is limited time actually…so medical edu-continued medical education is really fru umm…

I: Fruitful for that.

R: Fruitful for that. Plays a really good role in that.

I: Is antibiotic use and antibiotic resistance covered?

R: Uhhh…at some places.

I: At some places- okay.

R: Uhhh…

I: And do you attend regularly?

R: Yeah I attend- almost- almost all places uhhh in my radius I go- like if it is accessible, I go. Yeah, regularly I go.

I: Mhm mhm mhm- one last thing that we have to cover is…level of- your level of interaction with medical representatives. I mean- do MRs come? What is that interaction like?

R: Yeah, MRs come- um, at regular intervals…and we meet them also. But, at the meeting, just I listen…to the new release of medicines- and what are the- plans coming up- what are the new medicines- new combinations…that are coming up. And for everyone, I ask them for that…uh…published journals from which they have been quoting, so I ask…from all of them- where it has been published, and what are the studies done- I, get all those details from, each one of them, and I try to read into them, and I just- I go back and I look into those medicines whether it is really needed- whether the study is done- regarding those new medicines…and what are the effects. I do little bit of study myself before umm…going into that. I, you…just listen to them, to medical representative, just to know that such a medicine has been released. Basically, that’s my- as far as I’m concerned.

I: Okay- so do they normally provide evidence, or…I mean, what what, how do they interact with you?

R: Uhh- not all. At least, only about 40 to 45% provides evidence- real evidence. Even if you ask…repeatedly, only about 50% will come up with a…either that or they don’t turn up next time or they don’t show the real- uh…

I: And, in your experience, what incentives do they normally provide doctors to prescribe?

R: I don’t…basically take- I don’t ask from them and I don’t take.

I: Okay- uhh- do you- can you say a little about what impact, if any, they may have on uh…antibiotic resistance?

R: Yeah they also have lot of impact, because uh they may…provide…some big incentives, or big…things, if you look into- them- their products, they may try to, but [*chuckling*] I don’t know much about that because I don’t basically take. Because, before prescribing a pediatric medicine, you should actually look everything about that medicine, like not only about…the thing whether the patient will take that medicine also…whether it is palatable, starting from whether it is palatable to the patient…whether the patient compliance, because for an adult patient, it may be easy like…you can tell that patient to take that medicine, but for a pediatric patient, there are so many other factors also. Like, it should be palatable for the child. A child may not like the smell, a child may not like the taste- it starts from basic things for a pediatric patient, so…you have to look into everything, then the quality, then…umm, price, everything you have to look into a medicine, so basically for a, as far as pediatric practice is concerned, you cannot fall into a…MR, or even medical representative, you cannot…from the, their this thing, you cannot…prescribe medicine. Actually, we I taste most of the medicines myself, before prescribing also because then only you will come to know about…umm…the…the thing with the medicine, because whether a child may take. Some smells are very- really offensive smell that nobody will take such medicines. There are so many factors [*indistinct mumbling*].

I: Uh, is there anything else you want to say about antibiotic use, antibiotic you know, awareness…?

R: Actually, antibiotic awareness should be done at a regular basis all over the country. Big campaign should be run…and…so many, uh, people are coming up like…Doctors Without Borders are also coming up- so ermmm…so many are coming, but I say it should more frequent and more regular, and in an organized way like all the…fraternities should be invited and it should be done on a frequent basis, and scientific studies should also be presented in that. It should be a two-way interaction. Then, it will be good for the society and for the future.

I: Thank you so much doctor. Thank you so much for your time. We appreciate it.
