# Supplementary material for: “Without antibiotics, I cannot treat”: A qualitative study of antibiotic use in Paschim Bardhaman district of West Bengal, India
Source: PLoS One. 2019 Jun 27;14(6):e0219002. doi: 10.1371/journal.pone.0219002 (PMC6597109; doi:10.1371/journal.pone.0219002)
Supplement: S2 File — (ZIP) [file pone.0219002.s002.zip › S2_Transcripts/KAP 323.doc]

**KAP 323**

Gender: Male

Age: 28

Occupation: allopathic doctor

Highest degree: Masters degree

I-So uh doctor can you tell me uh what your typical OPD set up looks like? So is there…what´s your typical patient load?

[*not understanding the* question]

I: What’s your patient load like?

R- Ya its around 80-100 patients everyday.

I-Everyday. Ok ok

R- I- I- actually sit over Monday to Friday over here, from 10/10:15 to 1:15. That’s my usual duration. And on Saturdays, I have to work in the block hospital. That’s usually a night duty, from 5 pm to 10 am next morning.

I- OK, OK. And uh, how much time then, given the 80-100 patients, how much time per consultation?

R- Aaa 2-3 minutes per patient.

I- 2-3 Mintutes. And what does that- I mean what are you typically looking for- what do you ask- what are the general questions you ask?

R- basic signs and symptoms are there , the physical appearance, and general history- whatever past history, present history, the general look of the patient.

I-Sure sure. Are you typically

the only patient? I mean sorry the only doctor?

R-Ya I am the only doctor. Any…in the rural set up, there are very few- so suppose if you take the average, 3 lakh doctors are there. 3000 doctors should be posted in the rural area. They won’t take up such things. Since I am close to- I live in Asansol proper, the city itself- my home is around 9-10 km from here. Thats why I have taken this. If they would have posted me somewhere around 100-200 km from my home, it would have been difficult for me, because the gov’t won’t provide you any communication or anything.

I-How would you describe your typical prescription patterns?

R-My prescription patterns- mainly, the basic antibiotics or the pain inflammation because those are the usual cases that come and… that’s it. On…whatever symptoms usually preside- on that basis, the rationality of the treatment comes out.

I-So when you say typical antibiotics, what would that entail?

R: The typical antibiotics which the government provide, because one rule- one law- one rule is there. You have to provide the medicines as much as possible from this counter, which the government gives. Which the government provides. And they provide very less also, because they have a grading system for- it’s a PHC. And there’s a block hospital. There’s the district hospital [*phone ringing*], so…60…60 kind of medicines for PHC, 80 kind of- and it goes upgrading like that. So whatever medicines are available, whatever you get it from…the indent, usually you have to prescribe from that.

I: In this, do you mostly prefer generic medicine or brand name?

R: These are all generic medicines.

I: All generic medicines? Okay.

R: Eh, but if- any- chronic cases come, then I have to prescribe brand medicine.

I: Oh OK OK…

R: But, but that’s very less- very less. I- around 2-5% of the patients, usually chronic cases come, but I have to write because I have to go by the ra-law. Otherwise, they can sue me. Because whatever you HAVE to prescribe…whatever medications you’ve been given.

I: And what uh- what is your- I mean- perception of the difference between generic and brand name- in terms of?

R: Perceptions… the quality is definitely there.

I: Okay- like? Can you describe that?

R: The quality in the sense- uh…see, a generic drug comes at 1-2 rupees per tablet. OK? And a brand drug, a lot of…besides the branding and the marketing, the quality of drug is also quite high. So in that ways the quality and the branding itself provides you. Suppose- uh- you have a cold and cough. You come to a government hospital. You take the generic medicine. Maybe you are getting cured in 3-5 days. But…the quality of tha- those medications is, in this, brand- so called brand, gives you a better treatment and better quality also. You are able to- you can feel the cure!- you are getting cured within…hours or something. That’s the difference!

I: OK, and so, earlier you’d mentioned typical illnesses that you see-

R: Yeah- yeah- resp-respiratory tract illness. The diarrhea, dysentery, the fever with rash, particularly in the infants in the neonatal period of…that are the usual cases which come. And the different skin related disorders- dermatological problems, like scabies, and the pyoria is there. These are the usual cases which you, which I encounter over here.

I: So for this, what would you typically prescribe?

R: [*makes a sound*]

I: For the- for the, I mean, different illnesses you listed?

R: [*incredulously*] Depending upon the disease! Every…disease has a treatment guideline. I follow that, that’s it. Here, in my OPD, I follow standard treatment guidelines and ensure rational use of medicine.

I: So uh, suppose a patient was coming with a cough and cold.

R: Yeah for-

I: What is a typical course for that?

R: See, I have to check- fo-uh- depending upon the present, past history, and everything, I have to see. Depending upon the duration of the disease. And so depending upon- so whether to use an antibiotic or an anti-allergic- whether it’s allergy or a specifically…lung disorder, whether it’s a COPD. Chronic Obstructive Pulmonary Disorder, it’s a lung respiratory infection- depending on that.

I: Mm-okay. So among 10 patients, how many would you say you prescribe antibiotics to? Like on average?

R: [*pauses*] See, on an average, 3 to 5 [patients].

I: 3-5. OK, OK. Umm…do you prefer monotherapy or combination drugs?

R: Combination, obviously. Anytime, combination. Because, it will give you a better result- that’s it.

I: OK- can you tell me a bit more?

R: One is the result, one is the quality of medication, but over here…the generic medicines is all comes in monotherapy. So, I have to combine a lot of things. 3 to 4 drugs at the same time- to get the cure, to get the result! Because…here the people will see the result. They won’t take the entire duration of the treatment. Suppose…the, uh, treatment goes for 5-7 days- they will usually take it for 2 or 3 days, then stop the medicines. So, they want the cure within that stipulated period of time.

I: So, you up the-

R: So the combination comes. Suppose if you have a patient with chronic disease like tuberculosis, malaria- it’s a fixed-dose combination treatment, which is going on. OK? Then, suppose in case of tuberculosis- so…they have to take the drug daily. Previously, it was…three times a week. Now, the government has proposed a new rule regarding daily dose. Daily DOTS program. So, for BETTER CURE, and for better result, that’s it. Efficacy- increases. [*mumbles*: nothing else].

I: You mentioned that patients don’t take the full course. How often would you say patients don’t take the full course? How many patients?

R: Uhh…around…5-10 of the patients. If you take 5 patients out of 10 people.

I: Ok- will- will complete the?

R: will complete the course and 5 will be defaulters.

I: OK, OK, uh huh. So typically, do you typically give the full-

R: [*interrupting*] Because of the literacy! Obviously, I will always- I told you na- rational use of medicine and standard treatment guidelines. I always follow that. Strictly.

I: So what happens, say, if a patient lives far away- you know they can’t afford- I mean sorry not can’t afford, but you know that they cannot come back for the- so you just give the full course at one time?

R: Yeah.

I: OK…OK. What would you say- ?

R: [*interrupting*] Because I can’t trust the patient whether they are taking or not, because I can’t see what are they- what they are doing at their homes. So I have to give them the thing.

I: And for follow up? Do you ever check if they’ve-?

R: [*interrupting*] Definitely, because there is always a thing called- suppose if a patient is suffering from hypertension. He has to come every month to take the medicines, to get the BP checked, and that’s it.

I: Mhm mhm- okay. Uh how about…broad spectrum antibiotics?

R: There’s no such broad spectrum antibiotics over here.

I: Not here? OK.

R: All basic generic drugs are there. Suppose if a patient needs that, then I have to prescribe all brand things. Then, broad spectrum comes into the picture. SO, resistance…has already been developed, okay? A patient…takes drug from everywhere. He…not only comes over here, he will see a private, he will go to a private clinic. Already resistance is developing. So, I have to look for the patient, I have to analyze myself that- okay, spectrum is limited over here. Whatever…drugs are given. When to increase it? See the patient is suffering from 10-14 days he’s not taking drugs. Then, I give a broad spectrum drug. That also hardly 1 or 2 cases out of 10 people.

I: Mhm mhm mhm- so we mentioned broad spectrum drugs, combination drugs- what is the impact of all of this on antibiotic resistance? I mean in terms of-

R: It has a HUGE…it’s huge because the more you prescribe antibiotics, the more the resistance develop. OK suppose you start, you give one antibiotic- see I have around 6-7 types of antibiotics over here- okay, I prescribe, I try to prescribe at least one, so the resistance doesn’t develop. I try to remember correctively from the picture of the patient that I’ve given this- given this patient this drug! Maybe… to prevent that resistance to occur that early, then…okay secondary I have to…but uh, that…drug is not working at the same time. Then, I have to go for another drug. Then, somehow or the other- [*emphatically*] you can’t prevent the resistance! You can’t totally ignore it, but you have to prevent it to a certain extent that’s it.

I: Mhm mhm, mhm mhm mhm mhm. How often do you encounter…uh sort of, common skin problems, among patients?

R: Very very very common, over here very common. Because…the health, the hygiene is totally bashed over here. They don’t take usual measures in spite of repeatedly telling them that do the all that proper sanitation is a very- HUGE problem, huge problem over here. That’s why skin disease usually coming, because they usually take bath in the local things only, the local outdoor pools which are available over there, which are hardly clean. And the gram panchayat- they usually don’t take steps or measures to clean up them properly. That’s why.

I: Mhm mhm- so in that case, what would you typically give- for the kinds of illnesses you see?

R: Usually, there are no antibiotics as such. Uh…the…the usual treatment that usually come, the Flucanozol, usually Flucanozol 1, 1 Beebee lotion is available- whatever is available from here, we have to prepare that.

I: But no antibiotics typically?

R: No.

I: Okay okay.

R: No and basically no antibiotics for- [*gesturing to patients to be a bit quieter*] I usually don’t start with antibiotics. See, I have any respiratory tract infection, I first see whether it’s an allergy or not, because dust allergy is very common, okay? First, treat that allergy. Then, if the patient is not getting cured within two or three days, you give an antibiotic.

I: Mhm mhm- yeah, so do you perform minor surgical procedures over here?

R: No no no- minor…suturing…very rare, but yeah I do perform. But in a block hospital, I have to do that, because all the setup and all the infrastructure is available over there.

I: Okay. What kind of procedures?

R: Like basic suture- basic cuts and wounds. So I have to suture the wound- ligation, tubal- tubal vasectomy- these are the things. Basic.

I: Among these, in what situations would antibiotics be indicated?

R: See, any wound greater than 2 cm depth, you have to specif- you have to give an antibiotic…because they have to heal the thing- wound, so antibiotics are prescribed 90% of the time in those cases. If the wound is around more than 2 cm depth.

I: So and, we see that you have this rational use of antibiotics as you were saying- to what extent, if at all, does the patient drive the antibiotic prescription process? Like, do they ever demand it by themselves?

R: [*quickly*] No they never demand it. They usually- they lit- they totally believe the doctors over here. They have to believe, because- and when they are seen, because the word of mouth is a very important thing for doctors, and if they are getting cured, they will come again and again. That’s the thing.

I: Mhm mhm. Mhm mhm. So, but- they- do you ever feel the pressure to prescribe something that will get them cured faster?

R: Never never.

I: Doesn’t happen? Okay. Mhm mhm- do you- do you have a microbiological anywhere in your- ?

R: No no usually, any testing facility, we have to send it to the district hospital.

I: Okay. So, how often do you do that? In wha- how many cases let’s say?

R: 4-5 per 10 cases. Usually need- whichever is needed.

I: Mhm mhm- and you do culture and sensitivity and everything?

R: Yeah everything in the block. Some- some tests are specified for block, some are there from the district hospital…the patient has to travel on their own to get the test done. And if the patient feels cumbersome enough to get it done, because it’s very rushed over there, almo- 1000 to…1200 patients a day. Okay? Then, they will take resource from private setups.

I: Mhm mhm. So, in this setup right here, I mean you don’t do the…testing?

R: No no no.

I: So, how do you feel about delayed prescription practice? What do you do when you’re unsure of a diagnosis?

R: Mhm…see, we have a probational diagnosis for every patient…okay? Every patient has a probational diagnosis, because based on which, I’ll prescribe some medicines. Then, if the patient is not getting cured, then I’ll take help from uh…investigations.

I: Okay so in that- in the basic medications typically, are antibiotics included in that?

R: Uh- to an extent.

I: To an extent? Okay.

R: Not always.

I: Okay. And what are the- sort of the- first line antibiotics that you would give?

R: I usually use Amoxicillin…is one of them. Ciprofloxacin…uh, and Azithromycin. These are the three top most antibiotics that I usually use over here.

I: OK, if we come down to the illnesses, when you look at your upper RTIs and diarrheal illnesses, what is the typical course of treatment…for these uh?

R: See- upper respiratory illnesses we have to first rule out allergy. If allergy is there, no need for giving antibiotics. If allergy is there and patient is having breathlessness, then you have to prescribe an antibiotic. And cough for a longer duration of time, then antibiotics come into the picture with…cough syrups and anti-allergic. So antibiotics, anti-allergic, and a cough syrup. And uh…yeah these are the thing.

I: So before prescribing antibiotic, uh, do you confirm with sputum or stool routine? I mean how common is that?

R: [*interrupting*] That happens when a patient is suffering from cough for more than two weeks. Then, the TB picture comes into the picture. Less than 14 days is basically upper RTI or lower RTI that’s it.

I: Okay- okay- umm…let’s see, so what is your decision making process like when you’re prescribing antibiotics? What goes through your mind? What do you think about?

R: Step-by-step management of everything. Every step- sign and symptom, break it up, and then…

I: Could you break that apart for like a typical case?

R: Uhh suppose like for I- I just told you for upper respiratory tract infection- it’s a allergy. Okay? Cough. Fever, cough, and there’s breathlessness. So depending upon the thing, we’ve to give treatment for fever, cough, and the allergy. So an antibiotic, anti-allergy, and a cough syrup.

I: OK. Is there an in-house pharmacy here at all?

R: No. This [*pointing to his stache of medicines*] this is whatever in house pharmacy we stow.

I: So what happens if drugs are expiring…I mean what is that procedure like?

R: Oh okay if the drugs are expiring, we take note of it. We have a stock register. OK? We mention that- at first, take the- whenever we are doing the indent, indent of drug is usually done from the Bardhaman Medical College. OK? The drugs come, we note the expiry date, manufacturing date. We keep the- in the stock register only, we mention it, OK? Once uh…the pharmacist over here, who comes over here- he’s on a leave now actually- OK? He will take the note. He will inform me, then we either dispose it…proper measures…or send it to the block hospital. They also dispose it in the proper way.

I: What is the disposal mechanism?

R: Just burning and- deep burying and…or…burying and burning.

I: OK, OK. What are you- what affects the supply chain for the, I mean, what drives the supply chain for the antibiotics?

R: Whenever it is finished, I do the indent. Usually done 3 months- 3 uh once in 3 months. 4 times a year, it is usually done.

I: Ok. How do you manage patients when they say, uh, take improper dosage? How do you find out about it? How do you manage it?

R: Counseling. That’s totally counselling.

I: But with two minutes per patient?

R: No, no. I can…see, they have to have faith in me. That’s why they’re coming once in a- more than once. So they listen to me. OK fine. It’s not getting cured? Then they will go take help from another doctor. OK? Once they getting cured, I have to counsel it. 2-3 minutes is sufficient. If you can…that’s your skill and art. That’s it.

I: Yeah. Uh- what happens I mean- how frequently would you encounter patients who are self-medicating with over-the-counter medicines?

R: Uh…it’s very less…because most of them are very poor. So they…usually come over here, take the medicines, and go back. They don’t have money as- to get it from over-the-counter drugs.

I: OK, so if a patient comes, and he doesn’t need an antibiotic right away, but they live far away, you typically- what would you typically do? You…

R: Depending, depending. [*Telling other patients to wait outside for a minute*]. Whenever chronic case comes, like your [*inaudible*], I have to see it. That uh- what drugs did he take before…depending upon that, I have to manage it.

I: OK- OK. How do you weight the risks and benefits of prescribing antibiotics…with each patient?

R: [*pauses*] See, antibiotics have always a benefit role. OK? Overuse of antibiotics, and overuse of same antibiotics…will bring resistance and will harm your health. OK? So, I usually stick to one medicines per month. And when there is the resistance developing na…you’ll get an outbreak- we have a rumor register. Any outbreak in the locality will be informed to the doctor. That uh- some form of resistance is developing the- the…drug is not working. Then, I have to prescribe another medicines. So I’m trying to delay the resistance to a certain extent. In that way…the quality of medicines are pres- are…retained to a certain extent, and the resistance is prevented to a certain extent. So the benefit is always there, there’s no harm from medicines- antibiotics. It will always benefit you- it won’t harm you. But, if you overuse the same drug over and over again, resistance will develop and then the harm factor comes into play.

I: So how widespread is resistance in this community? I mean…

R: it’s not there.

I: Not there?

R: Because people don’t take the medicine properly. So resistance develop when…you’re taking it for long time. I’m taking it myself- my father is also a doctor- OK? I have eaten every form of antibiotic since my childhood. Some form of [*stuttering*]- I have developed resistance to most of the antibiotics. OK? Then I have to go to a generic- I have to have faith in my generic drugs, which I’ve not taken. OK? Then those works for me. So resistance already developed in a- 8 to 10 kinds of antibiotics? I have to have faith in those generic drugs, so that it can help me. That’s what resistance…you take the same drug over and over again, you develop resistance over that drug.

I: Mhm mhm mhm…[*long pause*]…Uh- what other things drive antibiotic resistance? So you mentioned you know-

R: [*interrupting*] Overuse is one, OK? Intolerance to drug is one. Poor compliance is one. Uh…taking drugs at the wrong time is also another factor.

I: Meaning?

R: Meaning suppose the dose is BD. You have to take the dose…after lunch and after dinner. The patient thinks…why to take the drug? Let’s take it once only. The proper duration is not maintained. So there’s 4-5…

I: Are you awa-uh- are you aware of any general guidelines related to antibiotic use?

R: Yeah. I told you na, I follow that book. It’s a bible of medicine- Harrison’s Principles of Internal Medicine. All statement guidelines are given there. Even in during the medical course, we’re…we’re supposed to follow that. Then…uh dat dat dat.

I: Hmm what about at the national level? In terms of the government putting out any guidelines.

R: Yes, guidelines are there to be then…WHO site is followed.

I: OK, OK. What do you think the utility is? Of such guidelines? I mean…are they followed by most people who are practicing or…?

R: [*looks hesitant*] I don’t know much- I usually follow it [*more confidently*], because all the health program related disease which WHO prescribes, you have to follow that- those guidelines.

I: Mhm mhm- what are your attitudes toward continued medical education and training programs?

R: Definitely. It has an impact, and I feel every doctor, till death, should go for CMEs…because…such a profession where there is updates every day. Every hour. You have to get that update, get that knowled- otherwise, how will you treat it? Look once resistance develops, you have to get to a source of…alternate system of medicine. Not the Ayurveda- in the allopathic only- other systems- how you have to change it, why to, how to cure the patient. Because at the end of the day, the patient comes to the doctor to get cured. So once resistance develops- in any medication, be it inflammation, be it pain, be it antibiotics, OK?

I: Mhm mhm- so do you regularly attend such programs?

R: Yeah uh, once in 3-4 months.

I: Ok, OK. And uh, coming to sort of your practice again, what is your level of interaction with- ?

R: [*interrupting*] See, I don’t conduct any private practice, OK? First of all, I have that thing NPP. Non-practicing allowance I’m taking that. So I haven’t started practice- private practicing at this moment. So I come over here, sit for three hours, whatever government duty I do…yeah that’s it.

I: No that’s fine! And so in this practice so to speak, so uh what is your interaction with pharmaceutical reps? The MRs?

R: Very less. Because…all the top brands won’t come into the…locality, they won’t be able to sell their brands. So, 3-4 people usually come over here. [*mumbling*] Very few.

I: So…and how often do they come would you say?

R: Once a month.

I: Once a month? OK. Ok. What impact do you think…MRs have, if at all, on antibiotic resistance?

R: Definitely. They have a…huge picture. Meaning, they have a role- that…to prevent the resistance, but at the end of the day, they only tell- marketing the brand. It’s up to the doctors to prescribe it. And how to control resistance. They don’t have much, but they’ll continuously pressure you to prescribe there there there there there. But it’s a- at the end of the day, it’s doctors who have to prescribe…I think doctors should not fall prey to the demands and the whims…the…what do you call that…atrocities of the different pharmaceutical companies.

I: When you look at antibiotic prescription, there’s a whole spectrum right- you have doctors who are prescribing, you have nurses, pharmacists, RMPs [*phone ringing*], and uh…community members who are taking them. So if you look at the full spectrum, what is each person’s role and how, you know, what do you think about each person’s role in this?

R: Ask her regarding that [*pointing jokingly to the nurse*]. Ok. See, first of all, the patient comes to the doctor. The nurse and the pharmacist works under the doctor, so they have to rely…totally on the doctor to prescribe medicines, so I don’t think they have much role, but definitely they have a role. If they have- they feel that one drug has to be uh—suppose they give very less- 6-60 different types of medicines? They give very less amount of medicines…OK? First and foremost. So the pharmacist or the sister working over there, the GNM working over there…they can give less medicines, so that lot of people can be catered to at the same time. So that’s a…huge role, they play a role.

I: And what happens in areas where there isn’t a doctor? The uh…top most professional is a ANM/GNM.

R: I can’t say anything about that…really, in most of the villages in…this district, there is a doctor.

I: There is a doctor, OK. Um…just…last thing really, uh, what recommendations do you have for reducing the threat of antibiotic resistance? At a national level and at a local level?

R: Local level, first, you have to prescribe the generic medicines. I’ll tell all the doctors- it’s my view- point of view. Give generic- start with generic! Don’t go for…higher spectrum at the initial stage. And counsel…

I: [*interrupting*] Why would you say that?

R: Because the lesser drugs have not become resistant at this point. The higher drugs have already become- because outside, you’ll go for better drug…to get the cure earliest. So, go for generic drugs- counsel the patient properly. And tell them to take the drug at the right time, with or without food, and all these things. That’s it.

I: What strategies have already been tried at a national level that you’re aware of?

R: Uh- strategies for antibiotic resistance…I think generic is already preferred- that’s why in all the government hospitals, you…have the generic kinds of medicines. One is that. And one’s…and another thing which government has prescribed in the PHC…60 different-80…the less amount of drugs, the less amount you’ll be able to prescribe. That’s it.

I: OK…thank you!
