# Supplementary material for: “Without antibiotics, I cannot treat”: A qualitative study of antibiotic use in Paschim Bardhaman district of West Bengal, India
Source: PLoS One. 2019 Jun 27;14(6):e0219002. doi: 10.1371/journal.pone.0219002 (PMC6597109; doi:10.1371/journal.pone.0219002)
Supplement: S2 File — (ZIP) [file pone.0219002.s002.zip › S2_Transcripts/KAP 324.doc]

**KAP 324**

Gender: Male
Age: 60
Occupation: allopathic doctor
Highest degree: MBBS

I: Doctor, so I was saying can you uh, describe your OPD setting a little bit? Like how patients come here, what kinds of patients come here?

R: Here?

I: Yes.

R: This, uh, general patients usually come here. And then specialist-special like ENT, surgery, uh…gyne- patients also are coming here.

I: So what kind of illnesses do you see?

R: Usually, uh…viral fever. Mostly viral fever. Some uh, diarrhea, dysentery. Some uh skin infections, like uh, what do you call it…you have itching, right?...skin skin infections. Besides these, tuberculosis patient because it is a dots center. Leprosy patients. These are the ones coming here.

I: Okay. And what is your typical patient load…in a day?

R: Per day, approximately…in the whole year, approximately 150 per day. [*talking to another patient*].

I: Yes, so you were saying 150 patients per day.

R: Appro-approximately.

I: Approximately. So, what is the time per patient that you take…for each?

R: Uhhh this depends upon the quality of the patient. Sometimes it takes only one minute, sometimes it takes 10 minutes. So it cannot be said how much time it takes.

I: So what type of things do you talk about with the patient when they come? What sort of things do you talk about? What things are discussed?

R: First- first of all, I want the history of the patient. As I’m practicing for 30- or more than 35 years, usually I can understand mhmm…what type of illness they may come to me. Particularly in case of viral fever, I think I should not spend much time, because there is no question of investigations here. We’ll have to…have this, to see this patient only by uh, seeing, the pulse, seeing the pulse, pressure, etc.

I: If you need an investigation sometime, what will you- ?

R: [*interrupting*] Yes, so I’ll have to prescribe them to do it in the outside, or I’ll have to send them to District Hospital in Asansol- referral centre.

I: If you send them to the private, do people go and get the private lab or no?

R: Noo! Uh, we have no attachment with the private lab. Usually, I send it to government hospitals, but sometimes- sometimes, it may be- uhhhhh who tho-tho-those patients who are telling it’s not possible to go to District Hospital, that is time consuming etc. etc. Then, I tell them to do it from outside.

I: Okay, okay. And do you normally- before prescribing antibiotics, I mean, do you normally do an investigation or get lab support before that or…?

R: Nooo- usually, I-I believe on my clinical findings simply. If I see that there is some infection, I will usually give the empirical antibiotic, but when it is not…relieving their symptoms, symptoms and signs, then I’ll have to have the…definitely antibiotics, or I’ll have to send this patient- refer this patient to higher centers.

I: Mhm mhm- what do you mean ‘empirical’ antibiotic?

R: Empirical antibiotic, suppose…broad spectrum antibiotics like.

I: Okay, okay- so what are the types of antibiotics available here?

R: Here, we are getting sha- all sort- mostly…uh, sulphur drugs. We are also getting um Cephalosporin, meaning Cephalosporin sulphur drugs, fluroquinolone derivatives- all this sorts.

I: Your –oxocillins and everything, okay. Uhh, so you mentioned broad spectrum drugs. In what cases do you use broad spectrum antibiotics?

R: Particularly in case of LRTI [lower respiratory tract infection]. When I’m taking, after uh [inaudible] I’m thinking that there is no case of pneumonitis, but LRTI is not responding by giving Paracetemol for 3 or 4 days. And there is- sputum is sticky-sputum is [inaudible] and thinking that there is- there is chance of having secondary infection, in case of viral fever, I’ll have to prescribe the…mostly Amoxicillin-Clavulonic Acid, or sometimes I have to give Lomifloxacin Moxifloxacin that’s it.

I: Mhm mhm mhm mhm. So, and if you think about sort of monotherapy vs. combination drug, which do you prefer?

R: I- it depends upon the quality of the patient.

I: Okay. Can you tell me a bit more?

R: Uh- regarding?

I: Just about this. When you use monotherapy, when you use-

R: [*interrupting*] Nah, usually I use monotherapy. But when I uh, think that the patient is not able to have the investigations…number 2, I have to recovery- they want the recovery. Huh in that case, when they are not able to have the investigations, when monotherapy is not working- in that case, I’ll have to give the dual therapy. Suppose I’ve given Amoxicillin one patient. Not responding. I have to give Amoxicillin-Clavulonic acid. Not responding. I have to give the fluroquinolone antibiotic…okay?

I: Mhm mhm mhm mhm. And how often does this happen that a patient doesn’t respond? Out of 10 patients, how many patients?

R: Uhhh actually problem is this- in this area, quack is there. So they’re using antibiotics. So I think there is antibiotic resistance. So in this cases, I’ll have to give antibiotics mostly, because they are- they come to me that I have taken this medicine. In that case, I’ll have to use antibiotic or higher derivatives. Otherwise, the patient will not respond- because they are resistant nah– actually.

I: Yes yes yes. How often are patients resistant? How many patients?

R: Uh this cannot be- actual, exact data I cannot give you…but mostly I can get it.

I: So meaning, are most of the patients who come to you resistant?

R: No, most not mostly, but at least 30-40% patients we are getting uhh getting antibiotic outside- they are also coming too.

I: Okay okay- and you’re saying- earlier you mentioned that patients are self-medicating. Is that true?

R: Yeah uh sometimes they are self-medicated or they are getting medication from…quack doctors.

I: Quack doctors- can you tell me a bit more about quack doctors? I mean, what-

R: No, I should not say it.

I: Okay, okay. Sure, sure sure. Ummm, so how- let’s- if I name some particular illness, can you walk me through what the treatment procedure would be like? So if someone comes to you…with a cough, cold, uh, and sore throat? Nothing else.

R: Yes [*interrupting*] but uh in that case, I ask them to use gargle, to give the uh, plenty of water, I also give them Paracetemol for 2 or 3 days. When I see that it is not responding, and in all my consultation I am getting bronchi [*inaudible*], then I start with antibiotic. Empirical antibiotic.

I: Okay okay. And what antibiotic would you start with?

R: Oh, the Amoxicillin-Clavulonic acid usually I start. If it does not work, I go to higher derivative like Cephalosporins, meaning third-generation, second-generation, or Amoxici- I mean floxacin, I mean this fluroquinolone derivatives like Moxifloxacin, etc. etc…okay? Sometimes I also prescribe some cough syrup.

I: Cough syrup…okay, okay. And do patients normally come back? I mean the-

R: [*interrupting*] Oh, usually do not, usually do not. Within 5 days-7 days, they’re all right.

I: They’re alright? Okay, so you- you don’t find the need to use antibiotic okay okay. When you give an antibiotic, do you normally give the full course or do you give- ?

R: Yeah, I’ll have to give the full course…for 5- at least for 5 days.

I: At least 5 days. Do patients take the full course in your opinion?

R: I cannot- I do not know because they do not give me the feedback. I think they’re alright- they will not come to me. And whoever is coming to me, I’ll have to refer them for investigations. Okay?

I: Mhm mhm. Okay let’s say a patient comes to you with just a general fever. What would you do in that case?

R: Nothing. Only to take plenty of water, cold uh- cold hot sponging, and I’m giving them uh Paracetemol. No antibiotic for sure.

I: Paracetemol okay. In this case, does the patient ever demand an antibiotic on their own?

R: No, no! I’ll not give.

I: Okay, but do they-

R: [*interrupting*] At least not for 2 or 3 days. Let them ask! Patient will ask so many things- why I will give it? 2 or 3 days definitely I’ll have to wait.

I: Okay. Alright what if it’s watery loose stools with or without vomiting?

R: Without- uh with vomiting, I give Ondosetron, or Domperidon, which is available here number 1…ORS…and just wait…for a day. If there is problem, then I’ll have to give Lomotil, though I know- that lomotil is now banned practically. I’ll have to give Norfloxacin-Tindaxole or Norfloxacin- Ofloxacin OZ in an offensive stool- in the case of offensive stool, with fever, etc. That is actually infection in the stomach- then I’ll have to give this antibiotic. Otherwise, I’ll just give ORS, uh plenty of water, that’s it.

I: [*pause*] Is there a patient waiting? We can pause.

R: No no no.

I: Okay. What is the- what about skin problems? Do patients come with-

R: Skin problems. Actually, usually this poor- they are dwelling in poor area. They really use the water of these ponds. They’re actually getting this short of infection which is itching, the itching is happening because they are…taking their bath in the ponds or rivers, hm? So usually they get this type of infection. We’ll have to give the BB lotion. Meaning Benzyl Benzoate lotion we’ll have to use, and some Cetrazil, or SOMETIMES…where it is not recoverable, we’ll have to give Flucanozol. Because it- it may be a mixed infection.

I: In what circumstances would you use a topical antibiotic?

R: No we do not use actually- topical antibiotic. But when uh…there is a case of ringworm, which is not recover by this Benzyl Benzoate etc., in that case, I’ll apply topical antibiotic. Not uh- normal we are not using it.

I: Do you perform any minor surgical procedures over here?

R: Yeah I- actually I did my [training] in surgery, and I stood first in surgery also in my college. Uh- I did surgery, but after my- after getting a heart attack, I’m not doing surgery. Not minor surgery- I was also in district hospital- as a surgeon- for 3 years, when I did [inaudible], I did appendectomy, I did hernia, everything- I did. But uh, recently, 2 or 3 years back, I had a heart attack, I didn’t- I mean operation except some small incision and drainage, like abc ess- that’s it. Cyst- small abscesses.

I: Okay okay. For that, is an antibiotic normally required?

R: Yeah, I- I give antibiotic because I think- in my hospital status, this is not so much sterile, so I must have to give antibiotic, otherwise there is chances of an infection.

I: Infection. Okay. And in that, what is a typical course like?

R: Yeah 5- 5-10 days. Usually I give Ciprofloxacin or uh Cefaxim or Cefudoxim.

I: Okay. And you give the full course at one time? You don’t ask the patient to-

R: I give the…full dose. At one time! And tell to come back to me to tell whether they’re alright or not, in cases of surgery [*trailing off*]

I: So when you are giving an antibiotic as a prevention measure, is that recommended in any guidelines or I mean what is the typical-

R: [*interrupting*] Actually…why I am giving as a preventive measure, because there is a chance of having infection. We are telling nah- we are not in a setup, which is fully sterile, etc. etc., but they are so much dirty in their room. They will- meaning, even they do not use uh, soap- chances of infection is there. I have seen this- from my experience of what I have seen that in this case suppose…she may have diabetes or not. That has not been uh, tested. So, if I give this uh, antibiotic, chances of infection is less. Otherwise it may uh, suppose she’s got diabetes- and from this, there’s chances of [*inaudible*]. So thinking on this thing, from my experience I give this…antibiotic. Particularly in this case.

I: What are the risks and benefits of giving antibiotics? How do you weigh the risks?

R: See, risk is there! Because uhhh….unnecessary use of antibiotic may lead to resistance…may lead to meaning, what do I say…[*stuttering*] it is also expensive. The third one is Don’t- don’t think- I think that there should not be use of antibiotic unnecessarily. When that is there- when there is need of antibiotic, we must use this antibiotic, we cannot think…suppose we are using Kanamycin, or Gentamicin, we are sometimes using Gentamicin- see, in that case, those who have some kidney problems- if I use this, kidney failure- chances of kidney failure is there. So some antibiotics- fluoroquinolone suppose derivatives- fluoroquinolone we are using. There is chances of some- uh- skin problems. Kidney problem, liver problem is there particularly Ciprofloxacin and other Ofloxacin. Chances of liver problems are there, so there are risks- of antibiotics- unnecessary use of antibiotics…so I´ll have to give Amlodipine **in** n this case, because we are using Amlodipine. We are also, sometimes we have to give Enalapril, and sometimes we have to give Telmisartan.

I: Mhm mhm. What um, what is the availability of uh, testing in the area then? You always send to district hospital- microbiological testing- ?

R: No no no. I have said- that those people who are not able to have the…uh, usually we refer it…but some patient says that it is not possible to go to the district hospital. Okay? So in that case, I tell them you can have here in free access to have your investigation from outside in a good laboratory- must be good laboratory.

I: Okay. How do you treat upper RTI and diarrheal illnesses? [*did not understand*]. Upper RTI? How would you treat an-?

R: Upper respiratory patient?

I: yeah yeah.

R: In that case, if it is a…for a prolonged period, suppose- more than 4 or 5 days- I’ll have to give Azithromycin. Okay? Uhh I think Azithromycin is also effective in GI tract. So…nowadays also there are some drugs- Azithromycin Ciprofloxacin combination…in that case, we also give…it from outside. Okay? Here also we give Cefodoxim with Azithromycin- in that case, this will act as anti diarrheal and as well as for the upper respiratory infection- not for the lower respiratory. Uh it will also act the lower respiratory too…because Cefodoxim is a drug for lower respiratory.

I: Do you confirm with sputum or other tests before giving?

R: No. Because there is- the sputum test can be done here only for the DOTS patients [referring to TB program of GOI]. We do the sputum test- nowadays, the Government of India has started the program of CB-NAAT which is now set up in the district hospital. Sputum negative patient, all patient we are getting we are sending it to at CB-NAAT. Suppose sputum negative, [inaudible at 13:48] **positive, we are sending it to CB-NATT.** Nowadays, they are also telling that in case of sputum negative patient, but you are suspect- clinically suspecting tuberculosis and also sending them to CB-NAAT to district hospital.

I: OK. What is your decision making process like? A little bit more can you tell me about how you decide whether to prescribe antibiotics or not? [pause] in each case?

R: In each case, I mean, quality of the patient I have told you. Suppose the patient has come to me, okay? I have given the Paracetemol, etc. Not responding. 2 or 3 or 4 days I have seen. There is no recovery from the temperature- fever- I think there is chance of having infection, secondary infection. In that case, I will be giving- this is well, clinical practice from my ideas, from my experience I have seen it and I’m doing it. Without having any investigation, what can I do?

I: Are there any guidelines or trainings you are aware of, with respect to antibiotic use?

R: No no, no such-

I: So anything from the government?

R: I think- I think there should be some training.

I: Can you tell me more? What kind of training would you…?

R: Like uh uses of, proper uses of antibiotics, etc.

I: Mhm mhm mhm. What are your attitudes toward continued medical education (CME)?

R: I always- I think there should be continual uh…medical education. Each year or…alternate year.

I: Okay. Do you regularly attend such programs?

R: Uh I was used to attend all these programs…uhh conducted by different medical companies or…uh IMA. Huh, I used to have, but last 2 or 3 years, as I am having my heart attack and I’m also 61 years of age- at this age, I’m just- just restricted, huh, to attend this training, but usually, I would umm…attend this type of CME- regularly, done by IMA or by some medical companies.

I: I mean, uh, what about use of medical representatives? Do they come…frequently?

R: Yeah.

I: Can you tell me a bit about that? Your interaction with MRs- what that’s like?

R: They are all businessman- they will always want that I will write like this, but I think uhh…I don’t always…uhh…allow the uh [*muttering in Bengali to himself trying to recall the right word*] products. If I use this type of products, I only see my net- I want to know the pros and cons of antibiotics and another drugs. If I think this is suitable, I use it.

I: What kind of-what do they talk about with you? I mean, do they provide scientific evidence or?

R: Yeah, they give scientific evidence. Sometimes, they also give me a CD, okay? Their papers, etc. they give me.

I: Okay okay okay. Do they ever host conferences or anything like that and invite you?

R: [*interrupting*] Yeah they do the conferen-conferences, okay, at different year. But I, for last three years, I am not attending all sorts of…specially, 2 or 4 per year, I go.

I: Mhm mhm mhm. So…you mentioned antibiotic resistance. What are the other factors that drive antibiotic resistance? What causes it?

R: #1 Misuse of antibiotic. #2 Low doses of antibiotic. #3 Inadequate intake of antibiotic. Okay? Uh…I think these are the things.

I: When you say inadequate intake, what do you mean?

R: Inadequate intake means, the patient, suppose I’m giving antibiotic for 5 days. Okay? It needs 5 days antibiotic- they are using only 2 or 3 days. When they’re alright, they’re not using it.

I: So full course sort of not-

R: [*interrupting*] Full course.

I: Okay okay okay. And are there any strategies we can take to control antibiotic resistance?

R: Uh, I think…uh, public…[*pause*]teachings are needed. What you’re- whatever thing that we need. But, how can I do- give it? I, personally, I cannot give it, because I can…tell them 1 or 2 people do it like this or what. But uh, this cannot be possible…public teachings, meaning [*stuttering incoherently*] laypeople why they will go? And how they will be understanding that this can cause antibiotic resistance. They will hear in one ear and out another ear. This is not possible in India, I think!

I: So you don’t think there can be much that can be done?

R: Yeah!

I: What- so are there other strategies that we can take, or?

R: What can I- you can…give some placards or- I think there’ll be no beneficial effect…because the public is not so much concerned about it. They are taking one day, two medicine, and they’re alright! They just stop it- finished! This is the study of the people- 90% people. Those who are educated, there is no problem. How much people is educated? Class 4 education is not education I think. [*pause*]

I: Hm. So, there are multiple actors in this- there’s the doctor, there’s the pharmacist, you have your ANM/GNM, you have RMPs, right? And you have your patients. So what do you- what are your views on each of the different actors? How do they all come together…to give antibiotics?

[*long pause*]

R: In my…health center, what I can do…is to teach my, uh- what I am doing, they are also doing this. They’re giving 5 days antibiotic, because they know these things. But I cannot tell the RMPs to do this. This is the part of the government! To teach them…that…ask the people- take the medicine for at least 5 days, or take the people [*stuttering incoherently*]. This is the part of the government, not for myself. For my citizen, I can say there should not be such type of lapse. They should know all these things. And usually I’ll sit with them- my sister, my pharmacist, I’ve sometimes…discussion- that I’ll have to give such medicine for this one. Ehh- this I tell.

I: And do they- do they do that in your absence, for example, do they…

R: I don’t know, I don’t know…because uh…I have not asked them.

I: Okay, okay. What do you think a solution can be for areas where there´s not enough doctors? Maybe more remote areas where there aren’t enough doctors. What is the role that can be played in terms of access to antibiotics you know, and knowledge about antibiotics- what can be done?

R: I cannot tell you that.

I: Okay…okay. Any other recommendations that you have? Maybe to curb resistance or anything else you want to say?

R: One thing…teachings for this RMPs is very much needed. Because what we are…getting the resistance is actually not from our side. From the, meaning, basic level it’s the RMPs. There are uh, thousands of RMPs. If they know the exact use of the…as they are from grassroot level, they can teach the people! That you should have to take this medicine…otherwise there will be harm…there will be umm…this is hazardous for your health, so I think starting from the RMP…RMP should be first, meaning, taught the exact amount by what kind of antibiotic they should use, or this should be banned by the…government that you’ll not be allowed to use this uh, antibiotic. Go straight away if you give uh Paracetemol for uh, fever, for 2 or 3 days- if it’s not relief, you go to doctor.

I: Right now, is it banned for them to use antibiotics?

R: No no no.

I: They can use?

R: They can use. They are using it! I don’t know whether there is any rule to uh, to guide them.

I: Ahh okay okay. Hmm…so how do you handle patients when they take the improper dosage?

R: This is a great problem, because whoever is in the habit of taking improper doses, they are always in the habit of taking improperly…doses- what I can do? When I’m seeing they are taking improper doses of medicine, I just teach them- I tell them…don’t do so, if you do like this, this will be harmful for your health. I don’t know they are taking or not.

I: Okay. Do you normally do this kind of counselling?

R: Oh yeah.

I: Even in the 1-2 minutes per patient?

R: Yeah oh that’s not the problem! I’ve said that I can take seconds or minutes or even 10-15 minutes also, according to- meaning quality of the patient.

I: Okay okay okay.

R: Suppose this patient having a paronychia, I take only one second time- 1 or 2 seconds time, because this is sufficient to diagnose the paranychia. I’ll-uh- meaning I did…surgery, so I can know that I’ve done surgery- I know that this is a paranychia. And this will be incised.

I: So and what about patients who are self-medicating with over-the-counter medications? How do you counsel them?

R: Uhhh…usually those patients who are coming to me, it’s not self-medicated. They’re medicated by the RMPs, not self-medicated! Those who are not getting cured by the RMPs…they’re- either they’re coming directly to me suppose 150 patient, 120 is coming directly to me. 30 is coming through RMP. That RMP is not recommending! But as they are not cured, they are coming to me. [*long pause*]

I: Uhh do patients normally go to several other doctors for their needs as you are saying or…is that common?

R: Uh uh…it is a…character of the patient, meaning in my long life of practice, that when they’re not being recovered by me, usually they go to another doctor. When he’s not available, only 2 or 3 days they wait. Then they’re going to another doctor. This is the practice of the patient, most patients! But uh…here is also some problem. Not problem. As I’m practicing for last 30-34 years, I have seen that some patients- suppose 50% of the patients at least- has a good faith on me…so they can wait for 10- 15 days even, till I’m saying not to go to another doctor, meaning who is a post- meaning specialist. They can wait. Meaning this type of…um I would get it.

I: Mhm mhm mhm- so…this habit of…changing doctors and moving on-

R: Yeah! [*interrupting*]

I: [*continuing on*] Does this affect prescription patterns at all?

R: Definitely, definitely!

I: Can you tell me more about that?

R: Uh, we the doctors are very bad in this sense, because uh, it is the habit of the doctors- suppose I’m writing Stamlo for hypertension, some doctor is writing Betacard. This is the same! But as he is a doctor, ego is…problem. He’s just changing the name of the…number one. Number 2, they are also changing the antibiotic. Suppose I’m giving Levofloxacins or, they are giving some another antibiotic. This is the problem!

I: Okay. Do they give higher antibiotic or- or- what?

R: Uhhh…that is not the factor, just- just changing the name is the factor. Ego etc. Number 2! Meaning they are just not waiting for…not telling! That you take this antibiotic at least for five days. Instead of this, what they’re doing, suppose they’ve taken 2 days of Levofloxacin, huh, they are changing it…to…Cephalosporin for five days. So this makes also antibiotics resistance. It’s a very important point. Suppose I’m giving Levofloxacin- in need of antibiotic- I’m giving Levofloxacin…and temperature has not recovered. They have not recovered the temperature. Another doctor has said ‘oh! This is not right. You just take…this type of antibiotic.’ So change of antibiotics! He has taken two days of antibiotic- again, another doctor has said- given 5 days. Instead of that he is going another doctor- he has also changing the antibiotic. This is a- this is another way by which antibiotic resistance is developed. No one is actually saying just wait and take it! I should say no doctor! No, most of the doctors are not saying that uh- just take this!

I: Mhm mhm- so uh, do you have a preference between generic and brand name medication?

R: Uhhh…I have not used generic medicine because- good question- meaning question of generic medicine, because uh what we are getting it, I am not writing any medicine outside. So I do not know the- what is the role. What is supplied by the Government of West Bengal, we are using it here. Okay?

I: Okay okay. So do you know any differences in quality at all- or any?

R: Somebody says that generic medicine is cheap, so it cannot work. I don’t know such type of- I cannot- I should not comment, because I’ve no- I’ve got no experience there…

I: But, when you give medicine to your patients, do you see a cure rate or indicative…do you see any?

R: [*gesturing that he does not know about this*] Generic medicine and this medicine I cannot say.

[Doctor was seeing a patient, and asked her if she had just come from the RMP.

Doctor- To whom you have visited?

Patient- At my neighbourhood, in my village.

Doctor-RMP, to RMP, you did not visit any passed doctor [Referring to MBBS].

Patient-No

Doctor-You consulted RMP. Nothing will happen; there is nothing to be afraid. But fever is not cured?

R: Now you [towards us]tell me what I should do. You being the doctor, tell me.

I: I’m not a doctor.

R: I should start antibiotics? Then? Without the investigation, I’ll not start antibiotic. Usually I would start this- Cipro- Ciprofloxacin, because at present, I have no Amoxicillin-Clavulonic Acid. Otherwise, I would start with Amoxicillin-Clavulonic Acid.

A patient came he was diagnosing the disease and discussing with us

Rina to the patient- Previous paper? Prescription or medicine from previous doctor?

Patient- he did not give me prescription, he is a compounder.

Doctor- Do you understand, compounder means RMP, and you understand the meaning of compounder? Those who passed [referring MBBS] are called doctor and those who are not [Referring to RMP] called compounder. In this case [Referring to the patient] I can give malaria test, I can do malaria, dengue, now it’s not dengue season so I don’t give. Usually I want means malaria testing is here, I tell them to have the malaria test, MP.

[Another patient came and the doctor is busy with him asking about the signs and symptoms.]

I-What happens to the drugs which are nearing expiry?

R-Ya

I-What is the disposal mechanism what drugs that are nearing expiry?

R- Disposal mechanism no usually

[*A patient came and we paused]*

*Continue*

I-Aaa so you are saying the disposal practices, expiry mechanism.

R-Suppose near expiry in this institution what we have done I have asked my pharmacist to see to give me a chart like which will be expired within three months, I use all these drugs within three months.

I-Ok so

R-There is no expiry, question of expiry here, in this institution there is no question of expiry here

I-So meaning that what are nearing expiry you

R-Na I asked my pharmacist to give a chart which is nearing expiry within three months so I use all these medicines within three months.

I-Ok ok ok, and what if

R-And if it has found that it will be not used within three months I send it to District Hospital Asansol for their use. So no question of expiry. Drug abuse is not there meaning all drugs are used here.

I-All drugs are used, ok. Is it is there ever pressure to prescribe antibiotics at all? When it’s not

R- No pressure, who will give pressure to me?

I-I don’t know that is what I am asking.

R-No no nobody will give me pressure, if they say also like give some antibiotics I think I am not in a position to hear such type of order from others, you give me antibiotic, I don’t think so. Because I am a senior doctor I don’t think so they will tell me like give me antibiotic, what I will think I will give it.

I- Alright doctor thank you so much, thank you.
