# Supplementary material for: “Without antibiotics, I cannot treat”: A qualitative study of antibiotic use in Paschim Bardhaman district of West Bengal, India
Source: PLoS One. 2019 Jun 27;14(6):e0219002. doi: 10.1371/journal.pone.0219002 (PMC6597109; doi:10.1371/journal.pone.0219002)
Supplement: S2 File — (ZIP) [file pone.0219002.s002.zip › S2_Transcripts/KAP 47.doc]

Interview 2: KAP 47

Age: 56 years

Education: H.S pass

Gender: Male

Occupation: IHP

Work place: Private clinic

I: So as we were talking to you, approximately how many patients come per day every day?

R: Not a set amount. Approximately 4 go 5 go- like that, we see.

I: And uh, the patients that come- among them, what kinds of illnesses do you see most?

R: Like the one that came just now- cold, fever, and after that you have headaches, body aches, the ones you have to give a pain killer for, eh, these are the kinds of illnesses we see.

I: And, these are the normal ones…in addition to these, do you think there are other illnesses that people come for?

R: They come, but I don’t see for those [illnesses]. If I don’t understand it, I don’t see them. We send them elsewhere.

I: Like, you mean…how?

R: Cold, fever, you know all the light illnesses that we can understand- those are the ones I see.

I: I see, and how much time does it take you to see each patient?

R: At least 20 minutes…[*short pause*]…per patient.

I: I see, and, I mean, what does the process of examining patients and prescribing medicines look like? Could you tell me a little about that?

R: Meaning, after I see a patient, I give medicines or I make a prescription. Hm?

I: And, I mean, what do you ask them?

R: I just ask- how are you feeling? What has happened to you? Hm? If they tell me this is what is wrong, whether it’s a cold, a fever, or a stomach ache, then I write medicines accordingly. If I do not have the medicine, then I write the prescription for the medicine.

I: And, if someone wants to get a prescription, as you are saying for example, how- how do you write that prescription? Meaning, the pattern- what all do you mention in [the prescription]?

R: Meaning, as in, I have it write here- the RMP one [*pulling out prescription note*]. When we write a prescription, we look at the illness, and then indicate medicines based on our understanding. Many people get better from this. If they don’t get better, they have to go elsewhere. I send them to LM hospital(District hospital is also known as LM hospital) [

I: So, uh, over here…meaning, uh…do you keep medicines here with you?

R: Yes we also keep a few medicines. You get medicines, but right now you can’t find it. Earlier you could find it, now you can’t anymore. I had to submit my license, which I did, but I’m still not getting the medicines I require. If we have it, we give it. If the medicine isn’t in stock, we write [a prescription]. Eh? Just like this.

I: So the medicines that you have…

R: Then we give it.

I: These medicines- where do you get them from?

R: We, like your uh…the wholesaler does not give us medicines. So we take it from the retail shops. Gives it to us at a discount rate.(Print dam me dete hai hum logo ko) So then, we disburse it for 5-10 rupees.

I: I see, very good.

R: The wholesaler doesn’t give us any medicines. Hahah, he gives it to the chemist, but he doesn’t give it to us practitioners. We take the medicines from retail. After taking it from them…suppose the cost is 50 rupees [*phone ringing*]…we charge 55 rupees. 5 rupees for us.

I: Okay, okay. And…among all the medicines you have over here, what kinds of antibiotics do you keep in stock?

R: See, the antibiotics we keep are Monosafe (200 mg), (for kids)…of Monosafe only (150 mg). Just these- I keep Monosafe. I don’t keep anything else.

I: And what about tablets or syrups?

R: Yes, syrups like if you have a cold, then we use Coscopin, Benadryl. Hm? And…bachake (kids)Coscopin or Pediatric we use, Monosafe syrup we use, all of these we use.

I: And there are a lot of antibiotics which work for a lot of illnesses, meaning what you would call broad-spectrum antibiotics…

R: Yes, yes [*interjecting*]

I: Do you keep those?

R: No.

I: Any combination ones?

R: No, no, we do not keep. Here in (It means in villages) [Parakhan?!] no one takes these. Whichever medicines are expensive, people do not take. Here, we have to give 50-100 rupee medicines. You know the situation in Parakhan (In villages). If I charge more than that, people won’t even come to me. They will go elsewhere.

I: So, when you are giving medicine or writing a prescription, what sort of things do you keep in mind? Or, what things do you have to think about?

R: Look, we have to think about his [the patient’s] illness.

I: This is, this is…

R: Yes, it is important. And, I have to think about what medicine I should give which would suit him. Which will be good for him. If he doesn’t get better today, he will not come tomorrow. That’s why I think carefully before giving medicines. If I don’t have the medicines, I write a prescription.

I: I see.

R: Yes.

I: So, this…what you were telling me right now that, uh, about money. So do these factors come into your mind as well?

R: Yes, it does. Look, here it’s in such a way that sometimes no one comes. If someone comes, they come for 2-3 days. For two days there isn’t even a sale. I just have to keep sitting here. And in Parakhan, you know it is very difficult.

I: Someone comes to you with an illness and asks for medicine- can you share an incident like this with me?

R: Meaning?

I: Meaning…a patient comes, a typical patient comes and asks you for medicine or told you about an illness. Can you share an incident like thi-?

R: [*interrupting*] No, without seeing, it’s very hard to give medicine.

I: Yes, after seeing the patient only.

R: Yes, after seeing the patient, we give medicine.

I: Does the patient directly come and ask for medicine?

R: When the patient comes, then only we give medicine.

I: No- I’m asking, does the patient directly say the name of the medicine or-

R: No, he does not directly say the name of the medicine. He tells us about his illness and then we give the medicine accordingly.

I: So, no one comes directly to-?

R: [*interrupting*] no no.

I: For example to give this kind of medicine.

R: No, all of this they don’t do- they go to another shop and ask for Dispirin or to give something else or pain killer medicine or Paracetemol. If he comes to us, we don’t give medicine. Meaning if there is a problem after taking medicine randomly, that will cause problem for us. Right?

I: I see.

R: That’s why I never give.

I: I see. Suppose you are giving someone an antibiotic- what do you think of during this? Particularly when you have to give an antibiotic?

R: Yes.

I: What are you thinking about then? Or we have to give this antibiotic- to take this decision, what things do you have to think about?

R: I have to think about- before taking a decision, I have to look at his illness. If I give it [antibiotic] to him, will he get better or not? Or will it suit him or not? That’s why we have to be careful when giving medicines.

I: I see- whomever you are giving antibiotics to, do you give them a full course?

R: Yes, I give them a full course. I give him a 5-day course- I give 10 tablets. He has to eat in the morning and evening- antibiotics are only given in the morning and evening. In the morning and evening, he has to eat for 5 days. After 5 days, he feels better. After he takes it for 2-3 days, he feels better, but if he leaves it- it’s better to take the full course.

I: So, this full course that you give- as you described regarding money before- do people take it for 5 days?

R: Very few people take the medicines for 5 days. Some people take it. Some people and say give full- for example, you have cold, or a fever, or if something like this happens. So, they take for full 5 days. And, many people don’t take it [for 5 days]. They tell me give me two tablets. So from two tablets, the illness does not go away. But even then, we have no choice- we have to give it.

I: So how do you explain this to the patient?

R: I explain- at least take the 5 day course. After this, your illness- you will see a difference- you will feel better. Without taking medicine, this won’t happen. But they don’t eat [the medicine]. 2 days- 4 days- over two days, if they eat a lot, they will take 2 tablets or 4 tablets. They just don’t listen.

I: So, what do you think- why don’t they won’t take a complete 5-day course?

R: See- money constraints or they just don’t want to take it- it is difficult to say.

I: Yes.

R: Yes?

I: Yes.

R: It is difficult to say why they don’t take it or they don’t even come. Even if you feel a little better, they should still come. To get more for another 1-2 days. They do not even come. It is very difficult here. Here- medicine- meaning, it just difficult to do anything [*chuckling*].

I: I see [*long pause*]. Do you get minor surgical cases?

R: Yes they do come, but I don’t do surgical cases.

I: So if someone gets a cut or wound…?

R: We bandage the wound. If there is need for any stitching, I don’t do it.

I: You don’t stitch?

R: Yes, yes, yes. For example, someone comes with a minor cut- we bandage the cut and that’s it. Nothing else.

I: I see. [*Pause*]. And, uh, any illnesses such as respiratory illnesses or diarrheal illnesses, what kind of medicines do you give for such cases?

R: See, patients come for diarrhea. For dysentery. But I don’t take a risk- I don’t give medicine because I don’t do stitching saline, so it’s not right to take a risk. So I send to the LM hospital-

I: So if?

R: SD hospital (ADH is called so) [*utters name of a hospital inaudibly while Rina is talking*]

I: If the case is not that serious, it’s a small case…

R: See, then, if there is a small case, I give Cifran CT tablet or Dep- this, Dependal-M I give. Right? Others I do not keep.

I: I see.

R: Yes.

I: Do you give antibiotics for such a case?

R: Look, if the party would take it, then we would give it, right? 2-4 tablets- they will come bringing 10 rupees to ask for 2-4 tablets. So then it becomes difficult to give.

I: I see. So how do you handle such cases?

R: We don’t do anything. I leave it.

I: So you send them to LM hospital?

R: Yes, we send them to LM hospital. There they can do stitching Saline and there are facilities for everything. And it’s important to send [such cases] there. It’s not right to keep such a patient.

I: Yes, yes, yes.

R: Yes, yes.

I: Do people come with skin illnesses?

R: Yes, people with skin conditions come. “Halka phulka khao phunsi aate hain.” So there we give Supramycin or another cream. If there is a tablet, Deroxid roxid 150 mg, I give that as well for older people. If it’s a child who is coming, then I give him “Kit.” Yes, that’s all.

I: So, if people come with some skin problems, do you give antibiotics for this?

R: No, we do not give.

I: No antibiotic to put on it [the skin]?

R: *[cutting interviewer off]* No, no. I don’t give high antibiotic. Make it work with 1-2 tablets.

I: So what do you do with such cases? After giving a tablet?

R: Suppose there is a wound- you give Ethrocyin. You give an ethrocyin kit- if a kid comes, there is this kit we keep and I give it. So, they [the patient] take 2-4 tablets with them. After eating 2-4 tablets, he takes it, the kit. If he feels better after this, he comes back and I tell them there are more tablets and ointments you can take. There is a skin cream you can take. He tells me ‘No, I don’t have money, etc.’ So that’s why they do not take.

I: I see.

R: Yes.

I: So, uh, you can’t do anything for, for this.

R: No.

I: I see. So, uh, uh, do you have a testing facility here?

R: No, no.

I: Do you tell anyone to go get a test?

R: I tell them to get a test, Botherin (spelling) test, all these tests, blood test- a lot is written down. The party also comes and sits down with me, but no one does it. They say, for example there is malaria, the patient comes with chills and a fever, so I tell them to get a blood test first. So they tell me ‘no I won’t get a blood test- will just eat a tablet.’ I tell them what will the point of just eating a tablet like this? I don’t give.

I: I see. So, uh, if you’re telling someone to get tested and he is not going for it, what do you for this patient afterward?

R: I don’t give him anything. He goes away. *[pause]*

I: *[mumbling:* I see]. Without a test, for him?

R: Yes. No no, I don’t give medicine. If I have to give Dicogyn for malaria or something else, it is difficult [to decide].

I: And for what things do you think you give tests? Meaning, if you could give me a few examples…as you said, malaria, and…

R: Like there is malaria. With fever, there is typhoid. In these cases, a test is required. Or there is sugar: it is also important to test for it. Yes? But people do not do it.

I: I see. So you think these are problems?

R: *[cutting interviewer off*] Yes, yes, yes.

I: Uh, how many patients, on average, do you give antibiotics to, among those that come to you?

R: Very few.

I: Very few?

R: Hm. They don’t come, as in if they have come- suppose they’ll come with 10 rupees and ask me to give them Dubocalpone. Will you give Chalocapone? What antibiotic can you give?

I: So they take the name of the antibiotic and ask you?

R: Yes. They come and tell me: give me CALPOL. And we tell them ‘no we don’t have it.’ We work with something else. I work with Paramed. Hmm? *[looking for affirmation]* All this… so then he says ‘no, it is written as Paracetemol.’ Well, but Paracetemol is an ingredient in that as well! But they just don’t take it.

I: So how do you handle such a case?

R: I don’t give it. He goes and gets it from a chemist shop.

I: [*cutting him off*] If you don’t give him what he asks for, then…

R: Yes- no. No we do not give.

I: Okay, okay. [*long pause*] Uh *[pause*] uh, so uh, you know the medical representatives that come, do they come to you?

R: They come.

I: Really?

R: They come. Sometimes, they come once a month, sometimes they come, but they come and take and then don’t come again [for a long time].

I: I see.

R: If I ask for a sample, they do not give it. [I tell them] at least give me a sample to at least get introduced to it. I can give a free sample [to the patient]. Then also, they don’t come. Once they had come, 2 people, from Kolkata and Asansol. They sat down and then did not come back.

I: So did you have another meeting with them, regarding medicines or….?

R: No. It doesn’t happen.

I: *[keeps talking}*

R: If they have come one time, then they don’t come again. They don’t come here at all- very few come here.

I: So…you don’t get information about medicines from them?

R: No…

I: [*long pause*] When you are prescribing medicines, in that…*[loud whirring sound in the background…long pause*]

R: Yes.

I: When you are prescribing medicines, how much of a role does a patient have in this? How much does the patient…encourage you?

R: A lot. Look, very few people get prescriptions. Suppose I do not have a medicine. There is some tablet- they come and ask for 4 tablets or 2 tablets, and I say no, I will give it according to my perceptions. But they do not take. Some people come and ask for a prescription that they can take outside. So I write [a prescription]. They give Rs. 20 as fees for this.

I: Does the patient ever tell you to only write this particular medicine?

R: No.

I: [*continues* speaking] for fever? Or they name a particular medicine?

R: They come, but we do not give medicine.

I: I see. You do not give them medicines?

R: No. *[Long pause]*.

I: Do you ever have to go and see patients in their home?

R: I do not go.

I: You do not go? And if they call you?

R: I do not go. They call me if it is serious. So if it is serious, who would go see it? That’s why I do not go…I don’t go, but a lot of people go.

I: *[pause]* Suppose you gave a full course of antibiotics to someone, and they have not completed the full course. How would you handle this case? How would you see it? What would you tell them?

R: See…I tell them if they do not complete the full course of treatment, the illness will not get better. But they…like they eat four tablets, then they do not even come back. So how can you handle this situation? I cannot go and call them, so I leave it. What happens is they go somewhere else outside. Outside they will go and give money- they do not come here. This is how it happens.

I: So do you ever do this, as in you think you should give a 5-day course, but you give for 2 days for now, and tell the patient to come back after 2 days?

R: Yes, I do that.

I: Meaning, then you will give for 3 days again. Does this happen?

R: Yes. I tell them that they have to come and take it. But they do not come.

I: I see.

R: They do not come.

I: What do you think? Are antibiotics allopathic medicines or some other type of medicines?

R: These [antibiotics] are allopathic medicines.

I: I see. [*long pause*] When you are prescribing antibiotics for someone, are you thinking about the risks and benefits for the patients? Do you think about this?

R: Yes, I do think…sometimes that there may be some harm. But then, sometimes there haven’t been many problems, so that’s why people are continuing- giving.

I: So I mean…how are you taking this decision?

R: I have to sit with the patients and then take a decision, whether I should continue with one course or not give it. Sometimes the illness is…I have to think that if it is some other illness, things could get worse. That’s why people sometimes don’t prescribe medicines, after thinking this way.

I: I am going to name a few illnesses. Please let me know what you would give for these conditions. Suppose someone has a cough and cold with a runny nose. What would you give him?

R: See, I give a syrup.

I: Which syrup?

R: Syrup like…I give Coscopin, like Benadryl if it is a very dry cough. Hm?

I: And…if there is a fever, then?

R: If there is a fever, I give a Paramed tablet and then uh…I give a choloromyestin capsule. [*pause*]

I: There is watery loose motion along with vomiting.

R: Yes. If there is vomiting, I give Domastyl or Reglan, or if [the patient has] dysentery, you can give Dependyl tablet for it. If that doesn’t work, then as a last resort, you can give Cefran CT.

I: Uh-

R: If required.

I: I see. And if there is watery loose motion but no vomiting?

R: For this, you would give Dependyl and a Acilog tablet to prevent gas, and I give ORS.

I: So suppose you have a stomach ache…

R: Yes, if there is a stomach ache, we give him Capstone, yes? Or we give him a Belargano (spelling?) tablet.

I: So all of these medicines that you named…were any of these antibiotics?

R: No.

I: So this is not antibiotic…?

R: No.

I: So in these cases, you would not prescribe an antibiotic?

R: No, no we don’t give. In uh…dysentery cases, people want to give antibiotics, but if they aren’t available, we give Gentamycin or Decadaron or an injection. [*Pause]* we give mecasin injection. Mecasin is an antibiotic, for the elders mecasin 500 mg which controls it. It kills the microbes.

I: As you said that you are having some medicines so it happens that you are having a large number of antibiotic of which expiry date is closer so how do you tackle those antibiotic?

R: Don’t give that.

I: So what do you do with that?

R: Throw them away.

I: throw them anywhere?

R: throw them, As we don’t take such then no question of apply.

I: No like every medicine will expire one day.

R: Yes so we take 10 and give that after that again we bring 10 after checking the date. We do it before expiry those which are near to expire we don’t take from shopkeeper.

I: ok so if it happens like it will expire in one month and you are have a large number of antibiotic what will you do with that?

R: So I throw them, don’t keep.

I: Ok

R: If I am having then throw them and if I don’t have then no problem.

I: So do you dispose anywhere or throw it anywhere?

R: No, I throw it in the pond or anywhere.

I: Ok. So the medicine shops don’t take it back?

R: No they don’t take that’s why we also don’t give. Suppose we took 10 and the expiry date is in this month, 5 are being sold and 5 are thrown away.

I: I see. Aa [*Long pause, sound of fan*] you must heard about antibiotic resistance?

R: Yes

I: If you say something regarding this.

R: Don’t know that much

I: It is called ‘Antibiotic Pratirodh’ in hindi, something about this?

R: Don’t know that much.

I: Ok. Did you ever feel or heard that medicine is not working on disease?

R: Yes I heard.

I: you heard?

R: Yes

I: So did this kind of case come to you?

R: Yes, came but I did not see, I don’t take up such case, I refer .

I: So you refer?

R: To Outside doctor to P.K.Banerjee to any other doctor in Asansol I refer. Wherever he prefers he goes.

I: So where do you get more information about antibiotic or any other drug?

R: See I mean I used to be trained under Dr P.K.Banerjee.

I: I see

R: Yes, after that from nowhere I train. From there I practice and got the licence from Kolkata and after that started practicing.

I: So now if you need information about new medicine?

R: I have to go to someone, don’t have knowledge about new.

I: So means you don’t know that much.

R: Ya

I: So when you are giving medicine means prescribing then do you use brand name or generic name?

R: No, I use brand name.

I: You use brand name?

R: We use generic very less. Generic does not work that much that’s why we give by brand name.

I: I see [pause*. Noise of car*] suppose a direahhoea patient came, having vomiting and loose stools so in this case do you give for stool test means do you see these things?

R: No, I don’t take p that case, I don’t see that patient. If comes to me I say that I don’t have required treatment for this, you take him to LM hospital or to some senior doctor. I don’t have the treatment, I don’t see such patients.

I: When you are giving antibiotic to one do you give information regarding the use of it?

R: Yes I do

I: Like?

R: I give like you take this [medicine] after breakfast, don’t take it in empty stomach and at night also take this [medicine] after dinner. Take this [medicine] two times, not three times. Antibiotic should be taken two times. We also write two times and if we prescribe then also we mention two times then it is in their hand, now if they take three times then it is their loss. Antibiotic should not be taken three times, it should be taken two times that’s why we tell them earlier that take it two times, morning and night. Yes

I: And if they miss a dose or don’t complete the full course then what can happen to them do you explain it?

R: About this, after this the patient don’t come, they come ones then they don’t come.

I: Yes, when they come ones.

R: Yes in the 1^st^ visit we tell them like this is the thing, take the complete dose then will be better.

I: How means how if you please explain more.

R: How means like we tell them that take full course and we also tell that if you take full course it will be better but they eat or not, after taking medicines from here they don’t come . they don’t even come to say whether they got better or not. Hey should come and tell. But they visit the outside doctor two times, three times, five times but here if they come ones they will not come for the 2^nd^ time.

I: so when you are giving medicine do you think that I have to do something so that they come for the 2^nd^ time?

R: yes they should come; if they take it will be good. They should come and say whether the medicine I gave was beneficial or harmful. They don’t come [*Sadly*]

I: Do you know any guideline or book regarding antibiotic?

R: Book is there but I don’t have.

I: I see

R: Yes

I: Can you tell the name of any such book?

R: See there was a Medical guide in which there was about antibiotic. I had but ek bar chori ho gaya tha dokan me then I lost everything. I used to keep everything here but now I don’t keep.

I: I see

R: Yes [*Pause for a long time, only sound of fan*]. Take Prasad.

I: It’s ok. [*Pause again*] so would you like to say something more about antibiotic resistance?

R: No [*few more words not clear*]

I: [*Long pause*] what is the relation between you and the patient while writing or prescribing antibiotic?

R: See relation must be very good. As long as the relation is not good he [Patient] will not feel relieve but it is difficult to say what he thinks later.

I: So as you said earlier that sometimes medicines don’t work [*yes from him*] so in this situation do you think you can do something? What do you think; can you do something at your position?

R: yes I can do but I don’t know much.

I: I see

R: Yes yes

I: [*Pause*] Assume that you did not give medicine to one but you prescribe, in this case what do you tell to the patient?

R: See we prescribe here after that they go to clinic or retail shop they make them understand means we only write that you have to take these many time, we only do that, we write medicine for three days. We write the prescription after that they go to shop and ask the shopkeeper, if they don’t say then come to me and I tell that you have to take in this way. I tell them that you have to take in morning and evening or take three times like that.

I: Do you tell them what medicine is written or for what reason?

R: Yes. Like if he is having stomach pain or fever or cold, I write medicine according to that. I tell them that take two spoons three times like that.

I: Ok so when you write antibiotic do you tell them that antibiotic is written?

R: yes I tell like this is Monocef tablet of 200mg, this is antibiotic, you have to take at morning and evening and five days. If you don’t take five days and take for two-three days then will not be cured, you will feel little better. But after that what happen I write for ten , they will take 4 or 5 after that they take or not they don’t come to me. If they come then will come to know whether he has taken or not or did not take at all.

I: I see. Did it happen ever that you prescribe medicine; he did not get it in the shop and came back to you?

R: No they don’t come.

I: They don’t come. Whether they get or not they don’t come?

R: No they don’t come, even if they don’t get then also. There [at shop] they tell them , they [patient] ask them and they say like Dr. has written like this and they are satisfied with that and go .They don’t come here, they should come here , should ask me or show me the medicine like I did not get the medicine you wrote so they give another medicine the opposite medicine. So he should come and ask should he take less medicine or not to take any. Suppose I wrote one medicine and he did not get and took another one so he should come and ask me but these people don’t come. This is very tough; to practice over here is very tough.

I: So does it happen that they come to you after visiting one doctor for two days?

R: No

I: After visiting you to another?

R: they go to other but don’t come.

I: don’t come. If they come how do you handle?

R: If they come then we see what that doctor has given and accordingly I do, if he gave such medicine he took for two days four days and did not get better and come to me then I will say to him what he [the other doctor] has written for how many days for one week, take one week after that I will do this.

I: I see [*Long Pause*] so as you said that you are having a small stock of medicine over so when you are giving medicine to one or prescribing then do you think that whatever you have you will give that only?

R: No

I: so do you manage that?

R: If I have which he [Patient] requires then I give otherwise I prescribe.

I: So it does not happen that they ask like give whatever you have.

R: No they don’t say.

I: So they don’t say that?

R: I write and say take it from outside.

I: So does that kind of patient comes who directly say name of any antibiotic or such medicine?

R: Yes they say but I

I: like?

R: those who ask I don’t give them.

I: Like what if you

R: like someone came and ask give me chloromycetin or this or that, I don’t give.

I: you don’t give?

R: Yes

I: So what do you say to him?

R: I say go and take from shop or go visit another doctor.

I: [*Long pause*] *sound of fan bike horn*

R: Take tea

I: No, thanks. When you are giving medicine to one do you say about that or something to do at home?

R: Yes i say.

I: Like what is known as health education.

R: Yes I tell them like I tell them that take this medicine to home and either eat or not.

I: With that something more need to do like if having cold cough so something home remedies?

R: Yes I said to them to take precautions, don’t eat this, don’t eat that, don’t take cold thing but whether he is doing or not that is another thing.

I: So do you tell this to every patient according to their disease?

R: Yes yes

I: So average how much time does it take for every patient?

R: It takes minimum half an hour.

I: Ok. So it takes that much time.

R: Yes and fewer patients is coming so one patient sits for half hour to an hour.

I: Ok .So it’s not problematic?

R: No, its not problematic.

I: [*Long pause*] Do you think that like what I said to you [*Sound of phone ring*] that combination antibiotic or something like that are available in the market or in environment? Means available largely?

R: It is available but I don’t get.

I: not you but if someone want can get easily?

R: [*Starts speaking before I finish*] Yes can get.

I: Or is there antibiotic resistance or drug resistance in the environment? What do you think is there anything in the environment? Anything is there?

R: no I don’t know such things.

I: you don’t know?

R: Yes

I: so if there is any training or something like that do you want to participate?

R: Yes then it will be good. Its good thing, if get knowledge then it feels good.

I: Do you want to tell anything about Antibiotic or any medicine or anything else?

R: No, now don’t want to say. [*Smiles*]

I: If you remember such incidents?

R: No, not such, not such thing.

I: Not such thing?

Ok thank you very much.
