# Supplementary material for: “Without antibiotics, I cannot treat”: A qualitative study of antibiotic use in Paschim Bardhaman district of West Bengal, India
Source: PLoS One. 2019 Jun 27;14(6):e0219002. doi: 10.1371/journal.pone.0219002 (PMC6597109; doi:10.1371/journal.pone.0219002)
Supplement: S2 File — (ZIP) [file pone.0219002.s002.zip › S2_Transcripts/KAP 60.doc]

KAP 60

Gender: Male

Age: 39

Occupation: IHP

Education: HS Pass

I: Can you tell me a little bit about yourself, a little bit only- like how, meaning, how long have you been running this shop? How did you start out?

R: This practice over here, near about 5 years. And this…I have two clinic…1 is in residence, Dharampur, and one is here. So, since 5 years I am practicing here OK. So, definitely I’m…performance is good. So, what is other question?

I: So, so what kind of training did you get?

R: training, I have training …I have 10 years experience in nursing ….

I: 10 years of experience in?

R: in nursing home, Daffodil medical center. I have experienced from 10 yrs, after that I am open clinic and practicing here.

I: So in a typical day, in one day, what all kinds of patients do you see? How many patients do you see in one day?

R: In clinic?

I: Yes.

R: several types of patients I am treating like injury, general fever, cough and cold…so many patient..that means general patients I am treating. Like..aaa…… fever ,cold, cough and injury , body injury, loose motion , vomiting.

I: so how many patients in one day in the clinic? Average?

R: average 5-10 patients I am treating here. And due to winter season patient is not coming due to dull season and just…since morning I have taken 3 patients.

I: At the clinic?

R: At the clinic in morning time.

I: Means today?

R: In morning time…in evening another…[*inaudible*]

I: do you also do home visit?

R: yes..home visit..i visit

I: how many patient for home visit?

R: mmmmmmmhh…[*pause]…*at least 2 to 3 patient daily.

I: ok 2 to 3 patient…how much time do you take per patient?

R: aaaah…mmm..according to severity of patient.

I: Okay.

R: OK?? Suppose an injury patient came and I have to give aaa.. stitch or whatever so definitely at that time I take more and more time…means aaa… half hour and one hour to the patient. Suppose a fever patient coming….. fever patient coming.then 10- 15 min is enough.so according to patient coming, which type of disease..

*(outside noise and 3^rd^ person asking something)*

I: you were saying sorry!!

R: so… as I told that as the severity I am giving the time of patient. I do know very well.so suppose the loose motion patient coming , I give 10-15 minutes only per patient. Ok? Atleast mmm…20-30 min is enough.

I:ok! So in that visit what do you..what do you ask? What kind of conversation do you have with the patient?

R:means aaa… which type of patient coming ..suppose that fever patient coming according…according to severiority and if fever patient coming I will ask to fever, cough and from from..from how many days you are suffer with fever…like this ask.

I: what about if somebody just comes in walking through the conversation so rina is the patient so what will you ask? What kind of questions do you ask?

R: first of all I will ask means aa … if I mention the patient name first or I will mention age and severiourity which disease with you come .so… … according to disease I will; give treatment.

I: aah!! So how do you decide what kind of medicine to give to patient?

R: suppose that patient come with fever an d cough ,,fever and cough.. at that time I according to my intention that should be amoxicillin or amoxicillin clavunate, in modern age this combination. So accrding to severiority I choose the antibiotic.ok?

I: Ok?

R:so fever patient so many antibiotics are available…ok

I: so antibiotic…how do you know that antibiotic is necessary?

R: because … if patient is coming with fever 2 or 3 days and he already taken paracetamol so definitely we should apply the antibiotic ..without antibiotic it cant be cured.

I:cant be cured?

R: cant be cured..

I:ok

R:so definitely I have to apply the antibiotic atleast	5 – 7 days

I: do you give 5-7 days antibiotic at one time? or do you give little bit and ask the patient to come back?

R: little bit

I: how much you give?

R: sorry?

I: so how much- what you give to patient? For how many days do you give to the patient?

R: I at least aa… means… aa.. here I am treating for 2 days only ok? I applied the medicine for 2 days , after 2 days I call the patient , come after 2 days for review what you want..ok? so after that if it necessary the medicine again applied otherwise according to economic condition of patient.lots of poor patients are coming ..that a … if the… if patient will recover within 2 days patients are not coming for review.he thinks that I am ok , what is the need to go the clinic so many patients are not coming. If patient is not recovery again come .and I apply the medicine again and I convinced the patient you have to take medicine atleast 5 to 7 days antibiotic .so if patient is satisfy then he taken medicine from my side.

I: hmm… and do most patient- do they come back? You say that you want them, meaning do the patients come back when you ask them to?

R:patient is not..means patient is…is fully care and not again come and is not cured is fully cured is come back here.i always convince to patient that you must take 5-10 days antibiotics.

I: 5-10 days.

R: but due to economic condition patient so many poor, so much patients are coming

I: so do you think most patients complete the full course or not?

R: No.no.

I:ok why not? One concern you are saying is cost. What else other reasons patient do not complete full course?

R: due to money..due to money

I: any other reason at all?

R:any other means aaa… I always convince to patient that you must come and take medicine for 5- 10 days. Actually one time patients are not taking fully course medicine due to economical condition.

I: So after that when you ask to review, why do most people not come? One reason is money.

R: hmm……money!

I: Any second or third reason why people do not come?

R: Second, third, meaning…money is the main reason…mainly it is…mainly, the thing is that…a lot of patients, any general patient…whether it is you or anyone does not pay much attention to medicine. I eat more medicine…he directly comes and tells me, give me some medicine doctor, which will give me relief in one go. Or in 1-2 doses. I do not want to eat medicine. I don’t want this, I don’t want that- despite this, I…we convince them that to get the right cure, you will have to eat the medicine. In that aa…meaning…they eat medicine for 1-2 days, get cured 75% and think they are fully cured, and don’t need to come back. This is the reason they do not come. This is the thing. No one likes to eat medicine, right?

I: Yes! And does it ever happen that you tell someone that you do not eat medicine for this particular thing, and then patient asks for medicine? Does this ever happen? Like suppose there is an illness-

R: We say…they come with a lot of diseases. A lot of acute gastritis, loose motion, because prevention is better than cure, isn’t it? So we tell just take precaution before illness…loose motion.

R: Suppose you have loose motion. In loose motion…you have to do the oily white…like…you have to eat Moong Dal and rice khichidi. Do all this and you won’t need much medicine. But even then…sorry…even then for 100% cure, you have to apply medicine *[pauses]* …this is what they do…okay? Medicine! Patient is coming to you for quick relief, so without medicine, it is can’t possible that he will be- she will be repaired.

I: Okay as you had said earlier, patient comes for like a cough and cold. What do you typically give to these patients? What medicine do you give? [*pauses*] Cough, cold, running nose is there.

R: For cough, cold and running nose, definitely meaning…are you saying antibiotics??

I: Meaning any medicine- meaning, what would you give typically?

R: Yes…so in that, if he is coming with a headache, fever…in that, you have to apply paracetemol in that. And if, you can assume in that we add an antibiotic.

I: I see! In what situation do you add an antibiotic? Why do you add it? That is what I am asking.

R: Patient…patient is troubled for 2-3 days.

I: I see!

R: Okay? In this age, there a lot of medicines such that patients eat them at home before coming here; for pain relief, he eats a lot of them at home before coming…and after that, if he comes to the doctor, means he hasn’t gotten better, we have to apply antibiotic to them, only then can they fully recover. So in the situation, according to severity, we, in that, we apply antibiotics.

I: Yes! okay! Okay if this happens…water diarrhea, with or without vomiting, so what would you give for this?

R: For watery diarrhea?

I: Yes. For loose motion?

R: In loose motion, there are a lot of antipyretic, such as you…ahh…Ofloxacin...you apply Ornidazole, Metrozil, and with that antibiotic comes combined. Ofloxacin and Ornidazole are the best which you give for loose motion, and for watery loose stool like Segadotril, Septriomycin- there’s a lot in capsule form, which you have to give for a fast recovery.

I: Yes yes yes.

R: For vomiting, like, Ondasetron ahhh all this you have to give.

I: What all?

R: Like…ondaseton tablet

I: Ondaseton?

R: hmmmm….domiperidone tablet

I: I see!

R:hmm…or also an injectable…you have to give an injectable.

I: I see! I see! What all…hmmm…if there’s pain in the stomach, then?

R: For stomach pain, you have to give an antispasmodic…this…there’s a lot from the Dotavarin group [of medicines]…compared to injection..

I: And if there are rashes?

R: If there are rashes, then I have to give anti allergy tablet.

I: Anti allergy tablet….you don’t give any antibiotic in this? Any topical cream?

R: You have to apply topical cream.

I: Is there antibiotic in that or not?

R: There is antibiotic in that cream.

I: Okay…hmmmm…..what, as in one or two names?

R: There is Ofloxacin …

I: Norflox???

R: Hm…Norflox well you …aahhh…[*clearing his voice*] …this cream is not from the Norfloxacin group. Ofloxacin, and in that there are many combinations.

I: I see….so how do you decide whether you have to give antibiotic for this or not?

R: If there is a lot of…you have to add antibiotic, even orally you have to add antibiotic. Depending on the type of rashes, if there’s a fungal wound, according to that you have to add an antibiotic.

I: What do you think if 10 patients come in a day, right, how many of them would you give antibiotics to? Like 10 out of 10 patients or 5 out of 10 patients, or 2 out of 10 patients…?

R: Out of 10…I give antibiotics to 10 patients.

I: You give antibiotics to everyone…I see! Does the patient ever ask for an antibiotic himself?

R: Patient doesn’t ask right, what would the patient know…patient does not know what an antibiotic is. Only the doctor chooses it and applies it.

I: They just ask for medicine, that’s it.

R: That’s it! They say, brother give me a good medicine, I want to get well soon.

I: aaaah….hmmmmm…you, meaning, confidence meaning, how sure are you that this is the right medicine for this condition? Like some are antibiotics, patient is ill, so they come to you…normally, how sure are you that this is the right antibiotic for this illness?

R: I am 70% sure that this is the right medicine for the illness.

I: I see! And if you’re not sure, what do you do for those 30% of cases?

R: If I’m not sure, I have to do an investigation or something.

I: Meaning how do you do it? Tell me a bit about it.

R: Suppose a patient comes with fever, there is a fever for 3-4 days, so there are many kinds of fevers. There is typhoid, malaria or many others in this day and age. There’s a lot of dengue fever around. So like that, we cannot just apply medicine without investigation. We give medicine for 2-4 days. If he recovers, then okay. Otherwise, I send for investigation.

I: Meaning blood, all that?

R: Blood! Blood! Blood, complete investigation, complete routine investigation needs to be done.

I: I see! Where do you send the patient?

R: I send the patient to the lab.

I: Is the lab nearby? Where?

R: lab…there are a lot of collection centers here, they collect and take it, so you know there are a lot of diagnostic center over here- we refer over to them.

I: And with him- does he then go to the doctor?

R: He shows us the report.

I: I see, he shows you the report. I see!

R: We see the report, and if it happens that this case is out of my hand, if he then doesn’t get cured by me, then I refer to a specialist. So there is a specialist for every illness, so I refer there only.

I: Yes, yes, yes. Also, what kinds of antibiotics do you typically have in stock?

R: We keep all, like amoxicillin , ciprofloxacin, norfloxacin. Then, your Azithromycin, roxythromycin. We have to keep all kinds of antibiotics. And if I do not have, then I prescribe from the medical store.

I: I see! I was just going to ask this, that where do you bring these antibiotics over here?

R: Here, we get it wholesale from Asansol.

I: You bring it from wholesale…I see! Meaning can you tell me a little bit about the price?

R: Price…whatever the medicine is, the price is according to that.

I: I see, I see! Okay…what you’re saying about the prescription, meaning do you do prescriptions for some people too?

R: hmm…I do prescriptions.

I: Everyone, meaning, whom you are giving medicine to and whom you are not giving medicine to also?

R: Means, a patient comes, a lot of patients say “doctor Sir, give me medicine from your clinic,” so whatever cheap and best medicine there is, we give it. Many patients say that “doctor, prescribe medicine to me,” so I prescribe. So according to this, they say give medicine from your counter so I give, and a lot of poor patients come, so with them, after giving a few medicines, I give without including my fees in that. It’s like this…whatever medicine gives us a little bit of benefit. Many patients also come who say, who do not have money. We do the treatment- go and give me money later. This also happens. Many poor patients come- he will come without money, take treatment and tell me he will give me money later. Whoever gives, gives, but many patients also run on credit.

I: Okay, so these antibiotics, after taking them, meaning, the doctor, the patient, the pharmacist, the RMP- what role do they have in giving it?

R: Everyone has a role. If it’s actively happening, everyone has a role. The doctor does what, he treats the patient, writes prescriptions, and the prescription goes to the pharmacist. He gives the medicine, plus the pharmacist brings the medicines from the stockist. He keeps the medicines and provides it in the market. The stockist also has a role. He brings it from the manufacturing department. So this is how the medical environment works- this is the kind of circle, right?

I: Do you ever keep combination antibiotics in your stock?

R: Combination antibiotic in today’s age definitely comes. In today’s modern age, there are a lot of combination antibiotics, like your Cefaxim-Ofloxacin comes, Amoxicillin-Clavunate. Okay? After that, Cefodoxim and Cefaxim is coming. Cefaxim and Azithromycin is coming…a moderate disease comes, where every patient will not be cured with one antibiotic. In today’s age, if a patient comes, he does not even wait 2-4 days with the doctor. He wants quick relief. If the doctor can convince him your illness will go away in 7 days, then it will go away in 7 days, but it will go when you complete a 7 day antibiotic course. So that patient is not even satisfied with that [doctor]. At a time, he changes doctors 2-3 times. If he does not give a cure in one day, he will not come back to me tomorrow, he will go to a second doctor. If the second doctor does not cure within 2 days, he will go to a third doctor. He changes doctors 4-5 times- in today’s day and age, the patient does not have any patience. Patience…no patience. He wants quick relief.

I: What are your thoughts about this- about this quick relief?

R: My opinion is that, it is that…see…my opinion is that you changed one doctor and went to another one- many diseases get recovered in 5-10 days.

I: Meaning on its own?

R: On its own! Suppose influenza…influenza will be there automatically for 3-4 or 5 days. If he changes two doctors, and he gets better by the third doctor, he has to get better in 3-4 or 5 days anyway. After 5-6 days, he goes to another doctor, automatically patient is getting recovered. After 7 days, he fully recover. Now that doctor gained fame because that doctor is the one who treated him, this is how it happens. So even after the convincing the patient, he does not listen to the doctor- he keeps changing the doctor. So before, approximately 20 years before today, patients used to have some patience. He used to get it treated- now that’s not the case. Now the patient wants a fast recovery. That’s why we have to choose a double antibiotic- many medicines, in many diseases.

I: So mostly, do you give combination antibiotics?

R: We keep both plain and combination...according to disease severity, I have to distribute the medicine. Many diseases come which don’t respond to a single antibiotic, so there I have to choose double, combination antibiotics.

I: What you said before, that meaning, the bacteria that are there, they are not responding to any antibiotics, we call that resistance, right? So have you ever heard of this term, like resistance? You just explained to me, so what other reasons may there be for this? In your opinion, one you said already- are there any other reasons for resistance? How- why is it increasing?

R: Hmmm…mmmm…that…see…that, in this age, it is the thing is that the environment has gone bad only.

I: Meaning? Bad environment meaning?

R: Meaning…environment, climate, food habits are changing, we food habits also I mean- suppose that fast food and a lot of and a lot of things- there are lots of bacteria and viruses in the water also…so the resistance happening, the resistance has to happen, it can happen…if it’s becoming resistance, so according to it, so which medicine is being resisted, if it’s not a good fit, then a blood investigation has to be done. Which medicine will suit him…for that…

I: Why does resistance happen?

R: Why does it happen? Why is resistance happening? … I don’t have knowledge about this.

I: Anything about the patient, meaning…any behaviour of theirs, because of which resistance may be increasing, or the doctor’s behaviour, or RMP’s behaviour, or pharmacist’s behaviour? Is there any reason like this?

R: There is no reason related to behaviour linked to this. In this, there is nothing about behaviour. After investigating resistance, which medicine will satisfy, that we have to do.

I: Meaning you are saying that this is natural- resistance is happening on its own?

R: It is happening on its own...

I: OK I will ask a different question. What can we do here, like you have your doctor, pharmacist, RMP, community member- the people who come to get medicine, you are there, the RMP- what can all of these people do together, to stop this, this thing?

R: To stop this thing, what can be done? In one organization, everyone has to sit for a conference. By sitting in the conference, get together- meaning, whatever individual things to talk about with the specialist, the specialist will also be there in the research center. Whoever is a good specialist in medicine, we have to take their view also. And we have to start an organization for the good health of the community. This is it, right! That’s why this happen from one person to one person. Everyone in one place- meaning sit you down and convince- what is there, what is not there, this research you have to conduct, then only it will be done. There is an expert also, no? In every thing, there is an expert. Expert advisor, expert viewer, expert specialist who is there, keep them all together, convince them, put everything in one place and decide what needs to be done. Like this…

I: Meaning everyone together…one, yes…

R: Coming together, how good treatment must be given to the patient, what is happening, what is not happening, do we have to give just treatment, meaning the view doesn’t align- that’s the thing.

I: So all of these people are in their own place- can any contribution be made separately?

R: They can do separately, but it is also important to get everyone in one place. You have 100% knowledge about this thing, he has 70%, I have 50%, and some have zero. So how will they come to know? Until you get in one place together, in one place, any specialist- any, any specialist comes and talks about this thing, how will it spread? The line that you are from, the line that I am from, the practical, I worked in a nursing home for 10 years. What they say- practice make the man perfect. In anything, theory doesn’t work, you have to do practical. So for practical, everyone has to come in one place, you people have to plan- and what- how you have to do- you have to run an organization- you have to get everyone in one place, then only something will happen.

I: So do you have any RMP association?

R: We have an association.

I: So tell me a bit about that?

R: Our association, meaning we come in one place, however many we are, once in the year.

I: Where is this association? Is this association only of Asansol or the whole district?

R: In Asansol...it is only Asansol…in Asansol once in a year, in a district library or at one place like Lamia park wherever, we…do the whole program, all opinnions are there, what treatment needs to be given, what should be done, how, in what way, should we do what to each patient, which type of patient should be taken, lots of things are done. Like that, our organization runs.

I: Who organizes this?

R: Among us…go visit one doctor, Dr. _________, you have to go. He sits outside Riverside.

So he is also there, and there is another very senior person, he runs your organization, any meeting that happens, he calls us there, we go, lot of things are discussed, many MRs are called to the conference, there are a lot of MRs for their product, they keep calling us. There we go, there is a lot of discussion on a lot of diseases and a lot of products.

I: This organization you spoke about- how many RMPs are tied to it?

R: There are a lot in it, I, I guess there are 150-200.

I: And what things are discussed in that?

R: The discussion that happens, all this commonly like…see, he says that there are a lot of RMPs, there a lot of practitioners who don’t have knowledge but they have taken a case and caused trouble. If you do not have knowledge, then refer that person. Whatever treatment you have to give, give according to your degree. By trapping the patient like this- like this, a lot of discussion happens. I refer directly to ADH. [*background noise*]. I give first preference to ADH, because there is a lot of treatment happening there, a lot of it is proper, do not give preference to nursing home, but a lof patients ask us to refer them to the nursing hom, but first preference is ADH. In ADH, there are all kinds of departments, many different kinds of facilities, many different kinds of treatment are done, and the people with money, well they brother- they get their own advice, you don’t need to ask them. This is the thing…

I: When you were in the nursing home, how were antibiotics used?

R: In antibiotics, antibiotics in the nursing home- meaning, I said right- depending on the type of patient that is coming, depending on the severity he is coming with, that kind of antibiotic. Over there, injectable is used more often, injectable is used more, and it is important to use a lot of antibiotics.

I: It’s not important?

R: It is important [chuckling] in a nursing home. If someone is admitted to a nursing home, means serious patient. Now, if you don’t give antibiotic to serious patient, then they will never get cure.

I: What types of antibiotic was used over there?

R: [pause] Over there, you’ll get just like here, but injections are used more often. Lots of antibiotics in injections.

I: I see!

I: What do you do with medicines which are expiring?

R: If it’s expiring, I let it expire.

I: How do you expire it?

R: There is a rule that you have to bury it in the ground or burn it.

I: If you have a lot of medicines which are about to expire, and a patient is coming, then what will you do? It’s not like it’s expiring right now- after one week, so how do you understand this? How- what do you do with this?

R: Whatever is near expiry, I remove it.

I: You remove it. You never give it?

R: No!

I: I see. Is there a specific place where you burn it?

R: See, we mainly- you have pharmacists- pharmacists normally have huge quantity medicines, and a lot expire at once. They have to think about it- we have only 2-3, which we just throw. So like that. We don’t have a quantity of medicine to pose problems- just put it in the dustbin and it gets dispatched on its own.

I: How often do you check whether it is expiring or not?

R: Always! Always!

I: Always! Meaning even then, how often…?

R: In a day, it goes, but once even then…now I bring the medicine and I see how many days it is expiring in, so before giving the patient, just I have to watch that is it expire or not. Before giving it, we check whether it is expired or not.

I: Would you like to say anything else about antibiotics?

R: I said a lot about antibiotics- you people know better [*laughing*]- what more can I say?

I: Whenever you give antibiotic to the patient, what do you explain to the patient about it? Meaning how to take or?

R: Yes, the antibiotic dose that is there for him, I have to explain to him accordingly. That he has to eat for this often, and for how many days he has to eat it, that I have to explain.

I: If someone does not complete a dose, then can any problem occur?

R: Lots of problems happen…many antibiotics do not have a clear dose. Patient does not get fully cured. You should definitely finish the entire antibiotic dose. Aaa…many patients do not eat at all, so they should definitely eat the full course. Many medicines, see, we are practitioners. We give medicine for two days, and say to eat it, fast…then you go to specialist and he will give you course for 7 days or 15 days, but the patient does not eat it. Eat for two days, then goes to the trash- now after that, what effect will happen or will not happen, he is not understanding, doctor is also not understanding, doctor also said for the patient’s benefit to do like this…but the patient did not eat, so his result keeps getting worse, na! Now, there are a lot of steroid medicines, like Omnacortine…in Omnacordine, it is such that if you apply it, you have to complete the course, you have it 3 times a day for 5 days, eat twice a day for 5 days, then once a day for 5 days. This is its full dose, the illness also got better and it wasn’t completely harmful either. Many patients leave after taking for 2 days, 3 days, so that can cause a problem in the future. It does not, but it can.

I: A little while ago, you were talking about medical representatives, you talk with them, so do they visit you? What interaction- how does it happen with them?

R: Yes…they tell us about their medicine. With their product, what should be given for which kinds of diseases, what the doses are, what the courses are, they come and convince. According to that, we use the product. If the product…if their product works well, if after the patient eats it, the result is good, then we use the product. But if after using it, the patient is not getting cured, then we have to go in another direction.

I: Okay if you do not know the patient’s diagnosis, then what do you do in this situation?

R: If I cannot get a diagnosis, then I go for an investigation.

I: I see, meaning, lab?

R: Lab!

I: I see…okay…look as you’re giving antibiotics, there are risks and benefits- right?- so how do you understand this issue? What the risk is and what the benefit is while you give it out. What do you think- can you tell me a bit about this process?

R: See, antibiotic should be started in low potency, okay. After starting in low potency, it won’t be as harmful. If you start in high potency, then it can have some side effect, okay. But low potency- but in low potency, it will take time for the illness to be treated. I know this, and the doctor is understanding, however many medical people are all understanding it, but the patient is not understanding. To treat the patient, we have to use the high antibiotic, so he recovers quickly…okay. So benefit and cost is in both, but there is more benefit. We choose such a medicine that it only benefits- does not cost anything, okay!

I: Do you mostly use generic or brand name?

R: We mostly use brand name over here.

I: Why?

R: Because…the patient gets good relief. By keeping the patient, okay?, there is another kind of satisfaction. A lot of brand companies like Ranbaxy, and many others- their products have 100% satisfaction that if we use this medicine, the patient will be cured. And whichever has satisfaction, we will use that medicine. Many MRs come- I take his medicine, and if the medicine is right, I use only their medicine. When the MR visits, if his medicine works well, you will give preference to him only. If you come to my clinic, you will definitely get respect. So the MR comes, and I use his medicine. Since he is coming over and over again, I will use it. When you meet someone, you have to see, so it works like this.

I: How often does it happen that a patient is coming and the medicine isn’t working? This resistance we are speaking of- how many patients come who are resistant?

R: That- well- I will only find out if he comes 4, 5, or 6 times. If there are many illnesses I don’t know about, what use will the medicine be?

I: Can you tell me a bit more about this?

R: Suppose a patient with pain abdomen is coming, okay, and, suppose that he has cancer in his stomach. So without investigation, I am not understanding and I am applying medicine- the medicine is not working, then I send him for investigation. So I don’t have this much knowledge that I will find out his illness from his investigation. Suppose that I send for USG. In USG, however much he understands, he made a report and sent it. But from the USG, he is not seeing that he has cancer, I am also not understanding. In the end, it happens that I have to refer it to the surgeon. Now if the surgeon has any experience, that his report is okay now what further investigation needs to be done- if he does it, and the illness is understood, then a new treatment will happen. This is the thing, na! And we provide investigation for the first time, that go brother, do an investigatation but no one goes. Now if he is in a lot of trouble, he goes. You find out the illness, it gets worse, so the case moves forward. He gets referred only. This is the thing.

I: Do you follow any guideline about antibiotic use? Like…or do you know if any guideline exists?

R: Guideline ahh nah…guideline- I have experience. You find experience in nursing home that which antibiotic is for what dose. The MR also gives us a guideline. This much experience I have, plus the MR gives- a lot of Drugs to Date I follow, that also has all the indication. That also I have to follow completely.

I: And from the government’s side? Do you know about any guidelines from the government’s side?

R: I don’t know about any guidelines as such from the government’s side. We have studied, did a course where we found all this from, plus from the nursing home, plus from whichever senior doctors were around, plus for internal view I followed Drug to Date. There is a medical book I have to follow. According to the experience, I have to provide the treatment.

I: If there was a program which provided information on antibiotics, would you like to attend such a program?

R: hmm…I would definitely like to participate, because, aside from the medical line, if you have learned everything at a time, this is not a big deal. I know in the practice line this is not the right thing- for one specialist conference or another, people are going here sometimes, sometimes to America. To learn something new day by day. To learn something new. If there is a new invention, then it will come to mind. So if there is such a program, then we would go too. Why wouldn’t we go? If we got the opportunity, why wouldn’t we go?

I: How much time goes in counselling each patient? A patient comes, you gave him some medicine, so how much time goes in explaining everything to him properly?

R: At least 15-20 minutes I have to give. And I told you right, that so many illnesses will be like- if he comes with a fever, I give accordingly some medicine for 10 minutes and discharge him. If he comes with a more serious illness now, it takes time to investigate him properly- at least 15-20 minutes it takes.

I: Ok thank you so much.

R: Thank you for joining us…have a nice day.
