# Supplementary material for: “Without antibiotics, I cannot treat”: A qualitative study of antibiotic use in Paschim Bardhaman district of West Bengal, India
Source: PLoS One. 2019 Jun 27;14(6):e0219002. doi: 10.1371/journal.pone.0219002 (PMC6597109; doi:10.1371/journal.pone.0219002)
Supplement: S2 File — (ZIP) [file pone.0219002.s002.zip › S2_Transcripts/KAP 64.doc]

Interview: KAP 64

Age: 45

Education: B.COM

Gender: Male

Occupation: IHP

Workplace: Private clinic

I: First, please tell us a little bit about yourself- meaning how long you have worked here, and ?

R: I have been practicing here for approximately 30 years- okay? And mostly, there are poor patients who come to me. Lots of patients are like that as you saw. Then I give a little prescription, or sample medicine which I give them. Because if we don’t cooperate with them, then who will? We have to go with the locality. I am not a pharmacist or doctor- I am an RMP doctor. I also want public support- that I can cooperate with the public. This is my thinking. Okay?

I: Do you do home visits?

R: Yes, see- those people who are unable to go to the hospital, or they have a financial problem- they don’t have money to go to the hospital- they call me and say “Doctor sir this is what is happening- will you come home with me?” Then, I go to their house and visit. And whoever is able to get treatment from me, I will provide them with some kind of first aid. How much can we do after all? I cannot play with the patient’s life, right? The way I think is that…if the patient gets better under my cooperation, then I support him. For example, suppose someone suddenly develops abdominal pain. Give a lot of painkiller or tablet **mumbling inaudible** once the patient feels better from this, I tell them to eat it twice a day, or three times a day. After that, if it is required, then you can go see a surgeon, or as per the requirement depending on the case, I refer and send them to Asansol District Hospital. Or the patient has a lot of money and he doesn’t want to go to the hospital- I tell them to straight away contact an MD first. After that, do what you need to do and contact accordingly.

I: So, uh, what is your approximate patient load every day?

R: Patient…number of patients?

I: Meaning…yes. How many patients must be coming to the clinic every day?

R: See- approximately 20-25 patients…end up coming.

I: Among these, what kind of illnesses do you see?

R: Mostly see, as you are seeing now- seasonal, with the season changing, cold, cough or fever, or someone gets a wound. Right? Suddenly, someone comes asking me: “Doctor sir please put dressing on my wound, I got hurt.” So this is the kind of patients we see.

I: How much do you take per patient? Approximately?

R: With one patient, we don’t take that much time: 5-10 minutes. Because we don’t take a full history of the patient, and it is not the kind of patient that we take a full history and then offer treatment. We just do first aid for the patient, including 2-4 doses of medicine or whatever you give, do a prescription, whatever we think is appropriate for the patient, we do.

I: I see. *Long pause.* And, these- 5-10 minutes you spend per patient…can you tell us a bit about what you ask them, what tests do you do?

R: Yes, yes. First, like, there are many patients who are like “doctor sir we have taken this medicine, we have taken this medicine from the medical store, this is what my problem is.” So I suggest to the patient, see if there is any problem, if there is any RMP doctor near you, see him and contact him rather than going directly to the medical store. What will happen then is that you will get better guidance than from a medical store. So suppose a patient tells us that he has not been feeling well for X number of days, so I will first take the history of the patient, asking them to show us the medicine they took first. So there are a lot of patients who have been taking medicine from the medical stores directly and eating for 2 days [before coming to see me]. After he doesn’t feel better, he comes to us. Before treating them, we ask: this medicine that you used to eat…how did you eat it? There are a lot of people who take it after food. But the medicine is better during fasting. There are others who are consuming antibiotics two times, three times on their own from the medical store- we tell him not to take many antibiotics- after asking the patient, we ask him if there are any side effects to medicines that he has taken, any allergy that has happened. By taking the history of the patient. Or if there is a treatment going on, whether is asthma, or sugar, or blood pressure- in this manner, we ask the patient. Then we give medicine, and if he doesn’t feel better, we tell them to go to an MD or contact a hospital (it would be better for you). Whatever we can do, we will do, but we cannot do a full treatment- I only do first aid for the patient in this manner.

I: So when you typically give the patient some medicine, do you prefer generic or brand name medicines?

R: So…in generic medicines, there are some medicines such as Paracetemol and ChareCIPLA (*inaudible)*- whatever antibiotic we give, it is by following prescription. The doctor is responsible for antibiotics. Doctors do not want to comprise anyone when it comes to antibiotics- by giving brand name medications, you will get a result! Was my diagnosis correct or was it wrong? It happens right that I’m giving it to you- suppose a patient with a wound comes to me- I give the patient a Ciprofloxacin tablet. I think to myself, okay well, I made the right diagnosis here. So, the patient gets cured. If he doesn’t get better, then I think I have a problem- after doing something and giving antibiotic, why did the patient not get better? Understand?

I: Mhm mhm. However many patients come- suppose among 10 patients, how many patients do you give antibiotics to?

R: Among 10 patients, I give some kind of antibiotics to a minimum of 6-7 patients.

I: What types of antibiotics are included in this?

R: Such as **Ciprofloxacin,** **Cefodoxim** a dry syrup for kids for cough and cold and things, you have **AmoxiClav**, **Ofloxacin** tablet, **Amoxicillin** capsule- all of these we use a bit.

I: So, let’s go according to illness- so suppose a patient comes to you with cold, cough, and a runny nose- what would you do for such a patient? That is all that has happened to them- cold and cough.

R: Cough and cold?

I: Cough and cold yes. So what would you give them?

R: **Amoxicillin** capsule, depending on body weight, 500 mg **lots of background noise**

I: And for how many days would you give this dose?

R: 5 days at a minimum. For the full course, we want patients to take for 5 days, but the patients we have that, forget about 5 days, they find it difficult to purchase for 2 days for some patients. So for two days they eat, and then as soon as their problem is relieved, they stop taking it. Then, they don’t take more and don’t come see me. They don’t finish the full course.

I: I see. But from your side, do you give the full 5 days course, or do you say that after 2 days…?

R: No, I tell the patient that see- do one thing- take Amoxicillin 500 mg according to body weight if we are giving him, along with an anti-cold tablet, I tell them to at least take 6 pieces each to take 3 times day for 2 days. After 2 days, however you are feeling, you come see me. The patient gets better and then doesn’t come see me. And if he comes, if there is some problem, I tell him to repeat for 2 more days. According to this.

I: So what would the rate be- for coming or not coming to you? Meaning, out of patients, how many come and see you and how many don’t?

R: 4-5 patients come- that’s it.

I: So mostly, they don’t come?

R: Yes yes. So then I assume either the patient has gotten better or he has gone to see another doctor. Or he has contacted the hospital. It happens like this as well.

I: Okay, if a patient has a fever and he comes to you, what would you give him?

R: See, I would give him, first, a **Paracetemol** tablet, and with that a **Chlonophenicol** capsule or something if we give, okay? After that, if the patient says he is not feeling better, there is an MD in front over there. We ask him to go show to him. Then the patient asks us to get a blood test or something done by the nurse. So, TSH HBS SR test we do- if there is a positive result in this, then we don’t touch the patient. In Asansol District Hospital, there is Dr. Sanyal, or we ask them to go to any other hospital, or go directly to ADH.

I: Meaning uh…for some things, you ask for a test?

R: Yes, for example fever- suppose patient says doctor sir my fever is not going away, can you do a test or something for me? So I contact them- there is a person I know around here who does blood tests- he comes to me from time to time. So, I tell him it’s like this, and please run this particular test. After conducting the test, we may understand some problems, then we refer the patient. Whether it is closed or not.

I: What kind of things do you understand by looking at a blood test?

R: You can understand a Widal positive test result. According to that- the full detail is written. TSH if someone has higher levels, then I suggest to the patient to go to the hospital to get treated.

I: I see, I see. Suppose a patient has diarrhea, with or without vomiting, what would you give them?

R: Firstly, I would suggest him to go to ADH. Or give a **Ondocetron** injection. 1-2 cc we push to the patient. Along with that a **Rentac** injection. Okay? If the patient has abdominal pain or anything, then **Trotaprin** tablet, or if the patient is having excessive loose motion, 1-2 **O2 tablets** or something like that as first aid. Anything else we send to the hospital. There is no better place than the hospital for treatment for such things. If you have to push saline or if there is a pulse issue, right? That’s why for these things we take no risk. Firstly, we avoid the patient and tell them we cannot do anything about this, you please go to the hospital. If the patient is requesting a lot, they are poor or it’s the night and difficult to find a auto rickshaw, so then I will give from my side- without taking any fees- to ask them to get from the nearby medical store and that we will push it [into the body] for them. If you get better, then good, otherwise take no risk and take the patient right away because you would require saline for this. And we don’t do saline.

I: You don’t give ORS?

R: Yes, ORS is a must. I give ORS. ORS is required for this.

I: And do you typically give an antibiotic or not?

R: In this case?

I: Yes.

R: Yes, as I said about **Ofloxacin-Ornidaxole O2 tablet** which you get- I give that.

I: I see. And if there is stomach pain?

R: We use a **Drotin DS** tablet- you get a Drotin DS tablet right?

I: Do you usually see a lot of skin diseases?

R: There’s a lot of rashes and all that we see- on the skin, some patients- the patients are unknown. First of all, if there is a doctor in my area, so first I will go to him. That´s why, first, as I told you, that mostly poor patients come to me. If a patient is qualified, he should go to this other doctor. He can take a proper decision, which we cannot. So if a poor patient comes to me, I give him an anti-allergic tablet or something like that, which he takes and gets some relief after having 2-4 tablets, otherwise we refer them to the hospital or a skin doctor.

I: So normally you give an anti-allergic?

R: Yes, normally. Like- **inaudible medicine name** etc. Common tablets like these.

I: How often- how often do you give a topical antibiotic, if at all?

R: See, with this, if there is a very deep wound, then I give an **Azithromycin** tablet for 5 days. I tell them to eat once daily, in the morning, fasting, empty stomach, right? For 5 days. And I also give an anti-allergic tablet. The patient gets cured with this, otherwise if not, we- just refer to anti-allergic…skin doctor or hospital, ask to contact. So some people- whoever has the money- Dr. Diptha Siris**inaudible name** in Gopalpur, right? There’s Dr. Mosur Alam, people like them, skin specialists, surgeons, whatever they may be, they go to them. Otherwise, I tell them myself that if there is a financially stable patient, I tell them, do one thing, if you want to go to the hospital, then that’s fine- or else you can go see a private skin doctor somewhere.

I: So, in this, is there any kind of lotion or anythi-?

R: *Interrupting** There is **Kelamine** lotion, etc., **Estabil** lotion, etc., something-

I: Any antibiotic to put on top?

R: No antibiotic to put on top.

I: Not there?

R: No we don’t give **mumbling**

I: **Pauses** Okay, when giving an antibiotic, meaning, there are benefits and there are also risks. So, how do you calculate this for each patient? What the risk is, what the benefit is…?

R: Like- we normally, as I told you, do not administer high antibiotics first of all-

I: **cutting in** I see, can you tell me one thing about this? What does “high antibiotic” mean? What would that be?

R: Like we get a lot of knowledge through Medical Representatives like **Cefodoxin with Clavulonic Acid** is there, **Azithran with Cefaxim tablet**, the ones with double antibiotic-

I: Combination antibiotics?

R: Combination antibiotics. **Ceftroxin injection, etc.** We never touch that. **Gentamycin** normal injection, etc, that you have- if there is a deep wound, then I use Gentamicin morning and evening. And with that as well, another single antibiotic- or if the wound persists, give an antibiotic injection ,and if the patient does not want to go for an injectable, then I give them a **Ciprofloxacin** tablet etc. **incredibly confident tone as he is listing medications- no hesitation on any medical uncertainty**

I: Yes, so, how do you weigh the risks and benefits? You were saying that you-

R: Yes, yes. Suppose that I gave somebody **Amoxicillin**. So I see if the patient experiences loose motion, etc. Right? If he has loose motion, then I give a **Metronidazole** tablet or something- after giving that, the patient feels a little relief. **wailing in the background** Lot of patients are such that they experience itching, etc, after eating any antibiotics. So with that I use a **Avil** *sp?* tablet, etc. They get relief from 1-2 tablets. After that also, if they are not relieved, then we tell them to contact the hospital. Like if we don’t use any high antibiotic, there is no tension that we gave the patient such a high dose, gave them a high antibiotic. What happened? How did it happen? I don’t work with such risks. As I was saying, I give 2-4 tablets to the patient as a first aid- what you have- otherwise patient doesn’t even come. If the problem gets worse, I tell them to contact the hospital. I won’t be able to help you. I do it according to this.

I: So as you were saying about not using combination drugs a lot-

R: I don’t use.

I: -so is there a reason for this? Meaning, why you don’t use?

R: Why I don’t use, see, little knowledge is very dangerous as you know ma’am**smiling, interviewers laughing** Okay? Dangerous to life. Whatever my capacity is, if I only practice according to that then my practice will go really well, I will be able to get dal-roti, I can run my house. And playing with someone’s life, well, that is not possible for me. That’s it. Right? Now, someone is having abdominal pain, so why is it happening? I am not an MS that I can give the patient an exact tablet or suggestion then and there, I do like a first aid- that yes, with a painkiller, I give a **Diclophenic injection**, give a **pentac injection,** after which if nothing happens, then I cannot do anything with the patient except referral.

I: Hm hm hm **pause** okay, do you do any minor surgical procedure here like suturing, or something else?

R: No, no.

I: You don’t do anything?

R: No no.

I: Then, if a patient comes, what do you tell them?

R: Yes, if a patient comes, local patient, then see I tell them- like there are a lot of garages here- right? There is a lot of work that gets done here- like labor. Suddenly, they will come to me- see this has torn **pointing to his leg**- so if it’s a lot or if it’s halka phulka rashes- then I give **Nevasal** powder and bandage it, I push a **Tedbac** [injection], gave 2-4 medicines- if it’s more than this, then after putting powder, giving first aid, and doing a dressing, after giving one **Tedbac,** I straight away send them to Asansol District Hopsital, the patient.

I: Meaning stitching you don’t do?

R: Stitching, no.

I: No. Mhm mhm mhm **pauses** so if you are thinking of giving an antibiotic, and if you don’t know really well whether- what the problem is-

R: Yes.

I: So how do you decide this- whether you should give or not give? Which one you should give? How do you take this decision?

R: In what case?

I: Uh…any case! Take a simple case- any example. A patient is coming, with some problem- how do you decide whether you need an ANTIBIOTIC **with emphasis** or not?

R: See there are a lot of- when the season changes, okay? After the season changes, automatically a person has cough and cold, things like that they have. So, immediately, I do not want to push an antibiotic, right? That see, with the patient, suppose that…there is a bronchitis patient- I know he has asthma and that he could have relief with some antibiotic- first a patient comes with cold, cough, and fever- so I give a Paracetemol tablet or something. By giving, the patient feels relief. If he says again that yes he isn’t feeling better- then with that, I give one or so antibiotics with it…that’s it.*Pausing*- suddenly, I do not use any antibiotic. Or you can go and just prescribe an antibiotic quickly- patient will buy or- without buying, how will they get better? Will he come again? But this I don’t do- whether the patient comes or not…my work I will do in such a way that the patient does not experience any side effect. Today, I have been here since 20 years, even today if a patient has not said anything good about me, he has also not said anything bad about me. I think that if a patient does not get better after coming here, he will go to some kind of doctor- doesn’t get better with good doctors- but I didn’t make a patient’s condition worse. Because if one patient gets worse, it will be highlighted more. I went there- this happened, that happened- I don’t want anything like that.

I: Mmhm- yes.

R: I just want to go on as I’m going- and serve the public.

I: So has a patient ever gotten worse in the past or any situation-?

R: Like that, until now, nobody has come for 20 years. I’ve been here as you all can see- this type of patient, you have seen 2-4 patients just now. Right?

I: Okay, you had mentioned MRs earlier. Can you tell me more about this? What type of people come, what they say?

R: See, many kinds of MRs come here. A lot of the companies that are there. Okay? There’s Mankind, NL Pharma,**inaudible**, Award company, Aristo company- these people come and do detailing like- do with the doctors.

I: Detailing meaning?

R: Like there are lots of anti-cold tablets- okay?- antibiotics are there, cough syrup is there, and lots of other things from the company. They do their detailing that this is how our medicine works, and that you should use this in this manner. So, our knowledge is increased in this process. For example, the use of antibiotics- where they should be used. We will not be able to tell you exactly. Through MR, we get experience. By getting experience, we can see patient if there is a need. Our experience improves.

I: Hm hm- so the medicine they tell you to give, do you give that medicine generally or do you decide other medicines?

R: No no, firstly like, they come to us, with some hope that if we go to the doctor, then there will be a prescription. So we see this type and keep in our memory that, brother, the company that has come here to me- I have to keep it in my knowledge. They give me some literature, keep it in the front- if some patient comes of that type, we do their prescription. By getting results, we push it further- then the patients come continuously, and we do the treatment accordingly.

I: By getting results, you mean you call the patient again and see?

R: Yes, yes, yes, yes-

I: I see. I see, I see…so just now, you said that you get a little knowledge from the MR?

R: Yes…we get.

I: What other sources like this do you have from which you would get knowledge about medicines or antibiotics?

R: See, we are- whatever antibiotics we have, we give. We cannot share that anywhere. If there is a senior doctor, we didn’t share it with him. Right? The MR that comes- when we get the result, we tell the MR that yes, we used the medicine, and there is a good result.

I: Mhm mhm- okay you just mentioned knowledge, so do you have any association of any kind? Or any place where RMPs-

R: **interrupting** Yeah, our, yes- we have an association. Probational medical practitioner association. Here you- maybe it’s every month or after two months. In this way, there is a meeting- however many doctors there are.

I: Where?

R: Number of doctors, however many doctors there are, everyone gets- in the Company. MR folks, from their side. Like there is Franco India- **inaudible**- these people like MR comes and visits. What they do- they choose 10-20 doctors that you know we will not be able to visit 10-20 doctors in a day. Do one thing- you all should….do a get-together meeting.Then, we also for example in some kind of hall, so suppose that- some companies are also like this that they say, come on, let us do a program in this hotel. After that, we want to do a program in Hindustan Residency. You doctors, please come there. So by going there, there are lots of- whatever companies there are- do a discussion about their medicines and our knowledge increases a little bit. We have all come together in a get-together- become familiar with each other as well- who has their practice where- so in this way, our knowledge is increased.

I: Mhm mhm- in this, do RMP doctors and MBBS doctors go together?

R: No, if MBBS came too, it would be much better for us as well, but only RMPs come. Now suppose, we say “ka” as “kha,” when it is actually “kha.” You will tell me no it’s kha, so someone should be there to tell us no you are saying it incorrectly. By having something like this, we get guideline, and we want that we can support the patient a little.

I: Yes, yes. Okay tell me one thing- what is the perception of RMP in the community? What do people think about RMP doctors? In comparison to MBBS doctors? If you…?

R: See, MBBS doctors are, many of them are happy with us. That, suppose that we prescribed an injection, we are the ones to give it, who else will give it? In the night, we are the ones practicing in village after village. MBBS will give his time, visit, and then leave. But the problem that the patient has, first of all check up we do, after that we refer. After that, whatever MBBS that are- they support us as well, saying “doctor saab, this is what had happened- I saw the patient who you sent, but if a patient comes like this, it would be better to send them straight to the hospital.” So sometimes, this happens as well. For instance, whichever MBBS doctors are close by, if the patient doesn’t want to go directly to the hospital (it takes one hour to get to the hospital from here). The patient will come here in 10 minutes, right? So when we send them there [to the MBBS doctor], the MBBS doctor also cooperates with us. Like, okay, however it happened, the patient comes to us, and we do whatever we need to with the patient.

I: Mhm mhm mhm.

R: Say something more, Sir.

I: **laughing** no no, yes we are continuing the discussion. Here, you are keeping some mediciens- like a small pharmacy?

R: Yeah, by something, I mean first- **inaudible** meaning your medicine- brought from medical store in Asansol. Okay? Wholesale medicine, and now the thing is, with wholesale medicines, the GST will start. We do not have a GST number that we can practice with it. Okay? Whatever chemist folks there are, wherever there is a retail counter, 2 strips or 1 strip of medicine like **Paracetemol**, *inaudible*, **Antacid tablet, Rentac tablet-** all of these we bring and we keep. And the rest as I said- that I give 2 or 4 tablets as first aid **shouting/screaming in background.** After getting some relief, if the patient comes again, they will take more medicine, otherwise we refer them.

I: So medicines- whose expiry- is set to be in a few days…in 7 days or one month…how do you use such medicines?

R: We don’t take such medicine at all. Before purchasing, when we are purchasing 1 or 2 strips, we will see the date of expiry first right? We are not taking used medicines. Right, and the number of patients also isn’t so high that we have to use it all in 5 days, if the expiry is one month. It’s not like that either.

I: And if a drug is expiring, you are seeing it, so what do you do with it?

R: I do not use it.

I: But what do you do with it? What do you do with the drug- meaning, with what you already have? You already have it. And you have bought, and it will expire in a week or in two weeks. What do you do with- ?

R: I don’t give that to the patient. I leave it. I throw it away.

I: Yes, where do you throw it?

R: There’s the dustbin. Dustbin like what we have here [pointing to his personal trash can]. People come from the corporation [referring to Municipal Corporation]- it goes in that.

I: So, does it go with the normal garbage or separately- is there another separate thing for medicine?

R: No, no- both together with everything.

I: Together?

R: Yes. Whatever dust etc we have, say if we did some cleaning, there is this dustbin. Those people come weekly, or in 10 days, in 5 days, in 4 days…and they take it with them.

I: And how often must you check whether this medicine is expiring or not expiring- the medicines that you already have?

R: See, within one year, it has maybe happened once or twice. Within one year, maybe it has happened once or twice **smiling**. This does not happen. That is why- I purchase 1-2 strips. I check the date before getting it. So, regarding short expiry- there is no question of that happening with me. You asked that’s why I’m answering you. Within one to two years, maybe it has happened once or twice that the medicine that is sitting here has expired.

I: Okay, earlier, you had responded to one of Rina’s questions- regarding first aid you were saying-

R: Yes, yes. First aid-

I: I have a question about this- in your view, do antibiotics get used as first aid? Antibiotic use?

R: Yes, yes, it happens.

I: Can you tell me more about this?

R: Yes. Suppose suddenly a person gets hurt in their leg. Okay? Gets a wound- a wound in it. With that, by giving an antibiotic, the patient will have a little recovery. Like that-

I: Mhm mhm mhm. **long pause** Uhh- and have you ever- uh- heard the term “antibiotic resistance”?

R: Yes, yes, yes.

I: Can you tell me a bit more about what you’ve heard about this?

R: There are many such antibiotics like that- that made the patient resistant. Suppose we assume that someone has…you…let’s say cough and cold? Okay? So with this, I could not decide which antibiotic should be selected for this. Instead of **Amoxicillin,** let’s say I gave **Ciprofloxacin**. Okay? So this here, resistance happening…the diagnosis was incorrect itself. Like if it’s an RTI infection, and if I give Amoxicillin, so the result is not very good. So, with Ciprofloxacin there- what result? We did not get, the patient was not cured…**mumbling** like that…

I: I see. And what things do people do by which resistance increases? Can you tell me a little bit? Why does it happen?

R: No I do not have knowledge about this.

I: No knowledge okay- tell me one thing. There are a lot of people involved in giving antibiotics, such as MBBS doctors, RMP, pharmacists- what role must they have in giving antibiotics? What, meaning, what is the responsibility?

R: Yes yes yes- antibiotics see- the situation is such that 99% doctors- okay? – practicing without antibiotics would be very difficult. Everyone in today’s date- whether it’s pharma, RMP, MD, DC, whoever it is- all have come to professional life. They are doing business. Everyone wishes that the patient will come to them more, and use more “high antibiotics.” If you use antibiotics- suppose we say the patient will not be cured, he will right away go to another practitioner. It’s better that we give antibiotics and control the disease. Without antibiotics, I cannot treat. In this way, however many doctors there are, they use it- antibiotics- for this reason…that the patient will get cured with me. Okay?

I: So what do you think? Is it being used a lot in the community or is it-?

R: **interrupting** yes yes antibiotics are used everywhere- used a lot only.

I: Used a lot?

R: Yes, yes.

I: Does it ever happen that the patient comes and directly asks you for medicine?

R: Yes, patient comes directly and asks me for medicine- that doctor, my head is aching, give me two **Paracetemol** tablets. I have stomach pain- give me **Cholimex** tablets. I tell them, do one thing brother- you go take it from the medical store. Or a good doctor- the way you are telling me, first you tell me what the problem is. If you take Paracetemol and eat it, so it’s not good- and medicines have side effects. So they say no I eat Paracetemol. So I tell them don’t use it a lot, if you use it. Why you eat it, go and ask a good doctor for advice. Whatever problem you have, go and contact a good doctor- why you take you tell the doctor and the doctor will give you the right medicine.

I: Does anyone ask for an antibiotic?

R: No, no one asks for an antibiotic- but when I just ask “did anyone give you any medicines?”[They say] “Yes, doctor Sir gave me antibiotics.” So if I ask what antibiotics they gave him, and they say that they went to the medical and see they gave Nohomox syrup (?), Amoxicillin, whatever it is- we have given to the child.

I: Sometime, just suppose, for cold and cough or whatever, you did a prescription- or you give medicines. Do they ever ask whether you gave antibiotics or why you did not give?

R: To the patient?

I: Does the patient ever ask you? That, whatever medicine you have given, whether you have given antibiotics in that or not- ?

R: Yes, there are a lot of patients which are like- antibiotic- they have only heard the name antibiotic. What antibiotics are they do not even know. If instead of an antibiotic, I gave a syrup- if I give **Trudec (?)**- they will say yes doctor sir gave me an antibiotic. But they do not know what antibiotic is. So this way the patient will ask me: doctor Sir you have given me antibiotics, right? Where I’ve given I say, yes I have given it. This way the patient is there-

I: Okay, have you heard- from the government’s side- do you know of any guideline? About antibiotic uses or meaning, how you should use it?

R: No no nothing like that.

I: Okay. What do you think like, meaning, in your practice- to stop resistance- meaning, do you have any thoughts about this?

R: See, if I have a senior, if he sits with 2-4 RMPs as a get-together- with that we will get some support with that, that this antibiotic should be used in which circumstances. What type of patient should be given- which, what kind of antibiotic. If we get some guideline, so even more guidance- patients will be relieved more. Suppose you have- any one- like you just saw a patient with mumps- we prescribe AmoxiClav syrup to them and then tell them that they meet some DCS. But suppose if that patient does not get better, he will go directly to the DCH. They will give high- in today’s date, we assume like Dr. [name removed]- #1 doctor among whoever there is in today’s date. They use a lot of antibiotics. Right? Morning, evening **inaudible** but we cannot do it like that because we are RMP doctors- that’s it.

I: Does it ever happen that someone else saw your prescription and they call you afterward to say why did you write it like this?

R: No, no no, until today…it was never required. Or it may be that it has not come into their knowledge or that the patient is not willing to give information, to whichever doctor or MD he has gone to. They saw the prescription and put it to the side- okay eat this.

I: So how do you handle the patient if he, say—takes the wrong dosage? Like you said you take this twice in a day- how do you find out whether they are taking it properly or not taking it properly?

R: Like a patient came in front of you just now saying “doctor Sir should I eat this medicine twice a day or three times a day?” The patient was confused. I asked have you taken or not, and he said no he has not taken. So I said okay you take this antibiotic morning and evening. Paracetemol and Rentac tablet that we have, you take that morning and evening.

I: And if-?

R: I give it according to this- and suppose patient takes it 3 times instead of 2 times, okay? So the medicine firstly I will give- I do not give more than 2 days of medicine to anyone. So suppose if he takes it 3 times in the first day, then the second day, whether the patient is relieved or not, the patient will come and report to me. We see that- brother, you have used the wrong way, it is an antibiotic- the way you are doing it, there can be many kinds of side effects. “So Doctor sir what should we do?” So with that I give **Trantacid syrup** etc to the patient. And I tell them not to make the same mistake again.

I: mhm mhm mhm. And if a patient cannot take the treatment, then? Meaning cannot afford the treatment or…does not have the money?

R: So there’s lots of- yes. Like in this area, in the year 2000, there was a fiasco during Muharram- Im not sure whether you know or not. So in that area, there I put my money and used to treat the locality during that time. There was not one man in the colony- meaning only older people, kids, were left- they you know there was nothing they could do- there was a curfew at the time in 2006. So, I told some people near the clinic that look, whoever needs medicine, I will give it free-of-cost if you do not have money. Because there were many people who, out of fear of the police, they left their house and belongings and ran away. The men ran away- the older folks…for a week or 10 days, how will they stay? U. Chatterjee sir had also cooperated a lot with us, a lot of counsellors or whatever there were counsellors etc at the time who would come here and distribute rations. Along with that, I would give medicines for free to everyone. And even in today’s date, I have given patients- from my side- I give medicine…free of cost. And here there is also- I should not say- I also give patients money from my pocket to buy antibiotics. Like I wrote a prescription to a patient for **Cefadoxim syrup**. It’s a kid, his fever hasn’t come down, and he has been troubled for 2-3 days. Paracetemol syrup, etc. we get here as samples. I gave him an antibiotic- and they say “Doctor Sir he won’t buy, he doesn’t have a father, has a garage and needs money for it.” I feel bad about that too. By God,I am saying, I have given patients money from my pocket to work. That’s it. Humanity is also a thing, right tell me Madam?

I: Yes, yes yes.

R: And another thing we see is that honesty is the best policy- with honesty, I have been here for 10 years. For an RMP doctor, to stay in one location like this for so long is a big deal. Okay? Like that we do. We want that whichever patient comes to us- he does not go away angry- and that we should support them as much as we can. It even happens that – like 2-3 days ago, I sent a child to ADH- he was having loose motion. Came in the morning- doctor Sir, if there is some medicine or anything, please give me some. There was a **Nonmetroxole** syrup- I gave him and said do one thing, show this to some DCH. He came in the morning and afternoon both and said “Doctor Sir if we go take the child to a doctor, first we’ll have to pay 200 rupees in fees and then he will write 200-400, 500 rupees of medicines- morning, afternoon how will we get this money together? So I said, do one thing- take a bus from here. It is 7 pm here. You can easily go to ADH. You won’t have to spend a single paisa over there. I suggested this to him, he listened to me and took his child and admitted him there for 2 days. Today, the kid is okay- yesterday morning he came to me and said “Doctor sir, I owe you a huge favour, you saved my child” and I said I did not save him. I became a guideline for you. I did cooperation with you, and you had less financial also- so come on I tried a little, and what more do I want than that? That’s it.

I: *Pauses-* Is there ever such a case, meaning uh, a patient has come- you can tell by looking that he does not need a lot of medicine. Or that he does not need an antibiotic. So if you do not, uh, give medicines, what reaction does the patient have?

R: Look, there are a lot of patients who need psychological treatment. That is a good question- you have asked a good question. There are a lot of patients who are such that –doctor Sir see please I have pain here and here, if I’m eating this, I am not getting hungry- a lot of…psychological treatment. We do psychological treatment for patient. If I do not do it, then the patient will not believe in me. For medicine, first I give him **Renatedim**. One tablet in the morning and evening before eating. And with that, I prescribe a B-complex tonic or something. The patient does not need it but the patient will request so much that we give it to them. They eat for two days and come and say now Doctor sir…better feeling. So, this is **laughing** psychological treatment. This does happen.

I: Anything else- what can we do to stop resistance in society? Any thoughts? Or do you have anything else to say?

R: Yes, I would like to say that see, however many practitioners we are, all of us practice with fear. We are scared that – as I said, little knowledge is very dangerous. We have an RMP course- what happens, how it happens- you only know and even the doctor knows what we are. Right? So I want that we are able to give treatment in the best way possible. See we are – we are here only to help, right? So there should be a senior, XYZ- who can tell us that see Doctor, the tablet you have given here antibiotic tablet- you should not be giving an antibiotic tablet here. You should do SOS- continue 2 days, 3 days, 4 days it will go away on its own. Right? So like that if there is a senior doctor who can support us, like suppose 2-3 days in a month, there is a class with 10 RMPs, so our experience increases through this process. This support, if we can have it, thanks God- **chuckling** we also be thanking you. Right? If there is support from a senior behind every junior, then their courage increases and the fear gets reduced slowly. As I said just now that a DCH doctor, Ceftroxin injection or IV, he writes for morning and evening. Us RMPs we think twice about giving a Gentamicin injection. We will go for it very rarely. What side effects may happen, what the result may be, right? So us, at least if we can get some experience that we also can give an injection. If the side effect of giving an injection is like this, then in its support, you can give this injection and this medicine. From that, what kind of support is happening? Suppose that a person is having a reaction to a medicine, like a Diclophenic tablet or something, so we have to give an antijection or something…**mumbling inaudible names of medicines**…according to this we have to give. Suppose that if we give this also, and the patient still doesn’t get better, the pulse keeps weakening- the whole day we cannot understand what we should do. So there we send the patient to ADH. And 10-15 years prior, the same incident happened with me. I was very troubled- even today that patient takes my name. One patient used to take Diclophenic medicine- after that, he started having a reaction. Diclophenic. Didn’t say anything to me- that Doctor sir when I eat this medicine, I get a reaction like this…I get a lot of joint pain in my sleep- so please give me some injection that will give me some relief. SO I tell him see Uncle the injection you take for pain or the medicine you take for pain- it is not good. Go and consult an Ortho. There is a Uric Acid, etc. test that can be done, or RF Factor test, Sugar test, etc. Whatever it is, do the test and better than taking the medicine, you consult an Ortho first. I think the patient must have been approximately 60 years old- he was very troubled by the pain- he came crying to me. He didn’t have a single paisa. I…Diclophenic injection- meaning I had an assistant get a Diclophenic injection. And I said okay uncle I will give you an injection since you are requesting so much. So as I was giving the injection, while giving it fell from my hand- must have been 7 ml in my injection and 2 ml fell on the ground, this was saving by God’s grace- I pushed the injection into him. After pushing, the patient went to the road and saying “doctor Sir, doctor Sir” he came back to me. Said what happened uncle? Take a look please, there is a lot of swelling. I understood there was some reaction. I asked him if he ever has a reaction? Then he tells me he used to take a green tablet, it used to give me a reaction. So I said uncle you should tell me this first right? I gave an injection of the same medicine- Diclophene. Do one thing- I am sending you to the doctor from here. Immediate- the patient got very nervous while talking. I- there were a few other patients sitting here and with their help, I got him in an auto rickshaw, I took him to the doctor. The doctor gave him an IV injection, I still remember, and pushed an IV. He asked me how this happened, so I said Sir see it’s like this- this is what happened, etc., so I brought him right away to you. After that 15-20 minutes, we were sitting silently in his chamber. And we saw that the patient was not in good condition and his pulse was slowing. I didn’t even tell his family members. I didn’t have the time. Then I took that patient with another lady to ADH. Leaving my clinic as is. After that, we gave the full history of the patient, there was an IV done there- the patient woke up at 11 pm. I told his family at home, okay? After doing that, his family members told me we are very grateful to you- that you saved my father. I told him I did not save him, I myself was saved. I didn’t tell your father but I myself was saved. Because if something had happened to him tomorrow, wouldn’t you say what injection did you give to my father? And that you killed him. Your father did not tell me that he had a reaction to Diclophenic tablet or injection. Now, whatever I say, they said no you supported us that’s great- after that, at 11 pm or midnight I came back to my house. And I’m telling you this also- motorcycle at that time betrayed me- it was during the cold. How I brought that patient from hospital to Railpar only I know. Even today, these patients come to me, saying doctor Sir you helped my father- he expired 2 years back. So in this way, whatever happens, we cooperate with the patients.

Say something else?

I: That’s it, that’s it for now.

R: It was really great talking to you! **respondent beaming** You came and helped us too. Thank you-

I: Thank you so much.

**Observations:** RMP said that there are many other RMPs who see themselves as MBBS doctors- they will give the patient any kind of medicine, without considering the effect on the patient’s body. RMP said he only charges 5-10 rupees above the cost of medicine, when he charged 100-200 rupees to patients in front of us. He also said he did not do any procedures or administer many injections, but there is an operating room inside. Finally, he said he sees 20-25 patients per day, whereas the actual number seemed to be at least triple this.
