# Supplementary material for: “Without antibiotics, I cannot treat”: A qualitative study of antibiotic use in Paschim Bardhaman district of West Bengal, India
Source: PLoS One. 2019 Jun 27;14(6):e0219002. doi: 10.1371/journal.pone.0219002 (PMC6597109; doi:10.1371/journal.pone.0219002)
Supplement: S2 File — (ZIP) [file pone.0219002.s002.zip › S2_Transcripts/V1.doc]

**V1**

I: So can you tell me a little bit about in which situations you, meaning, which things do you come to the hospital for?

R: That when there’s a bigger emergency, then I come.

I: Okay. Like which kinds of illnesses?

R: Like if there is a bigger emergency- vomiting, etc. or it’s happening regularly and not getting fixed from outside, then I come to government hospital.

I: Okay. When you’re saying that if it’s not getting better outside, meaning first where do you go?

R: First local medicine…I go to a shop or I go to the doctor who sits locally.

I: OK, OK. So when you go to the shop, what do you ask them?

R: Normal like, if it’s very…like normal, like if there is a headache or a fever, then I go and ask them to give me a tablet for fever. If it gets better with that, then okay, otherwise I go to a doctor.

I: So, meaning you first ask the man who sits at the counter of the shop?

R: Yes first.

I: I see. So in that, do you know any of the medicine names that you must be asking for or do you just ask them like that?

R: Well, I know the name of a medicine for fever…what, Paracetemol, CALPOL which is there. That, otherwise, I ask the shopkeeper that this is the problem I have- if you have something for this, please give it to me.

I: And in this situation, does the shopkeeper generally give it to you…the medicine?

R: Yeah he gives.

I: I see. And uh, and uh, does it ever happen that the shopkeeper tells you no you go somewhere else? For this illness?

R: Like if it’s very serious, then they tell me to go and see a doctor. If it becomes very serious, then we ourselves do not go to the shop.

I: I see, okay. So tell me more, like if there is vomiting. In what other situations would you come to the hospital?

R: Like…if vomiting starts or if things get really serious…food poisoning, etc. happens, then I go to…hospital.

I: Okay. If you have a general fever, so then what do you…what, first a local doctor or first…?

R: I go to the local doctor first only. Fever, or what happens in the season- cough and cold- we do not go show it outside [consult] for that.

I: Okay so can you tell me a bit about the local doctor, about what the interaction is like when you go- what do they ask you, what do they give you?

R: [*pauses*] What they give, they give me medicine! What medicine they give, this I do not know. Okay but they…ask what is happening, not happening- according to that, they give their medicine.

I: Okay. And what are your opinions about the local doctor…who sit over there in your community?

R: Look, the local doctors who have, meaning which have a normal physician’s degree, they are okay. And…the bigger doctors outside- they have higher fees, and nowadays it feels like they make a customer out of us. If you come once, then you have to go again. Meaning like…this is my daughter [pointing to his young child who is cooing in the background]. During the time of her delivery, meaning we went to show outside…we had not come to the government hospital, so they were making us take medicines every month. Per month, she used to prescribe 2000, 2500 rupees worth of medicines- Vitamin A this and that, and they used to take fees also…so I see that outside, meaning, the delivery happened, but a lot of money was spent, and even after all that, the girl wasn’t normal, meaning she wasn’t right. You’ve given her so much medicine, Vitamin etc. everything was given, but it wasn’t like that- even now, she’s 2.5 years old. But you cannot tell by looking at her that she is 2.5 years old. So, after that, then…meaning she was continuously ill soon as she was born. So she is having diarrhea and many problems, so we took her to outside providers only- we had not brought her to ADH. Then she went to her grandmother’s house. She got sick there as well, and then took her to some hospital where they charged 2000 rupees daily in fees. Tell me!

I: This is in the private, right?

R: Yes, private. They were taking 2000 rupees daily and I mean, nothing much was happening. And we did not even know what the problem is with my girl. Then, when she recovered there, then we took her here to a private practitioner, and now she is okay, but there is a lot of expense in private. And she has a little sister who is…okay I guess, no particular treatment since she was born. But it’s ongoing. So, if you show a big doctor from outside, there is a lot of expense.

I: That’s why you came here [to ADH]?

R: Yes.

I: I see. And is that the only reason? Is there any other reason why you came to ADH? Leaving private?

R: See over here…no one is satisfied completely. Okay? But even then it’s true that no money is being spent and where there is a problem, everybody is coming here.

I: Mhm mhm mhm. What doesn’t happen here, meaning what is…lacking?

R: Like see, you’ll have…here there is still this thing with the local politicians. If you are referred by someone, then you will be treated well. If you’ve just come like that, then you’ll be treated like normal care… [*trailing off*] now, until recently it was just like that. Yes. Now, since the new hospital has been constructed, I don’t know what the system is since then. But when the old one was there, it was just like that, that if they feel like it, they see you, if they don’t, they don’t see you. But it’s true that the doctor comes, and the treatment happens. It’s not that treatment doesn’t happen. It happens, but it’s the same thing- if you are referred by somebody, they will see more carefully what happened, and if you came normally, then normal- how they are seeing everyone, like that they’ll see you too.

I: Mhm mhm mhm- and how much time must the doctor give you…for each patient?

R: How much time? 2-3 minutes?

I: 2-3 minutes? They explain in that much time- what medicine it is, how it must be eaten, you’re able to understand?

R: [*pauses, then chuckles*] Here, mostly you get the needle [respondent was waiting at the vaccination center].

I: You get a needle? I see. Tell me more about what all you get done.

R: Medicine, etc. like they say, the nurses come on time and administer it.

I: So you come here for vaccination, right?

R: Yes, over here.

I: I see, I see. OK, OK. So have you ever…the doctor must be writing a prescription?

R: Yes.

I: He must be giving it to you?

R: Yes.

I: So what do you with the prescription afterward…after taking medicine?

R: I go and give it to the medical [shop].

I: I see, so you give them the prescription.

R: Yes.

I: Then, the medicine comes.

R: Yes.

I: Then, what happens to the prescription?

R: Then it stays in the house.

I: It stays? Do you use it ever again? Ever?

R: Like that, we never use it again. If something happens again, then we go and show it again.

I: You show it again, so a new prescription comes?

R: Yes.

I: I see. And the old one remains lying there?

R: Yes.

I: I see, I see, I see. And mostly, when you go to the shopkeepers, so do they- if you ask for some medicine- do they ask for its prescription first or not?

R: See, if I have shown a doctor and taken his prescription to him- if I give that, then it’s okay. And if it’s cough, cold, etc., you get it even if you just ask like that. For example, if you’re coughing, if you ask for any syrup, etc…they give to me.

I: I see. Have you ever heard of the name antibiotics? Antibiotic medicine?

R: I don’t know a lot, but a little.

I: A little. What do you know about that? Can you tell me a little bit?

R: Meaning…that is like antibiotic does that to some thing….what do you call it, I mean…like…[**word at 6:39**] right? I think you call it that in Hindi? Yes, so that…[*long pause*] even though I have heard that it isn’t right to take antibiotic.

I: Really?

R: A lot.

I: Who have you heard this from?

R: This, maybe, I don’t know- I had read it somewhere, or else…just like that.

I: Uh huh. Why isn’t it right?

R: Because this, meaning, like the way evolution is…if we use a lot of antibiotics, so like, if we are using a lot of antibiotics for a fever, then the thing with that is that the fever…will…happen like that, the fever will only start happening according to the antibiotic. So then the antibiotic will also not work. If we use it a lot. Which is why we should use antibiotics as little as possible.

I: I see, so where did you hear this? This thing?

R: This, Sir, I had read in some book.

I: Read in some book, I see. That’s a good thing. That’s a good thing. What else can you tell me about antibiotics?

R: Just that only, I mean, antibiotics are not even used a lot in foreign countries. Like there are…a lot of other people, like when they come from outside, and say they do not eat antibiotics. Because they say that by eating it, I mean, they then…are not able to get treated…properly. So this I know, but in our India, a lot of things are used, mostly antibiotics are used.

I: So which illnesses are antibiotics used for?

R: Mostly- like I know, mostly- like I don’t know a lot of them, but mostly for fever, etc-, antibiotics are…what is used.

I: I see- fever, etc. And why do people fall sick generally? What are the various reasons…for illnesses?

R: Generally there is this…water, the water.

I: Water. Water, meaning?

R: Water means, does not come clean. That’s why we fall sick. Then the population is so big. There is pollution in the cities- it happens because of this. There are many other…many reasons [*smiling*], but mostly it’s because of water, changing seasons, like this. Water is the reason behind most illnesses over here.

I: When you say water, are you speaking of drinking water or the water you bathe with?

R: Drinking water.

I: Drinking water, I see. And when it comes to bathing…

R: [interrupting] Bathing is done…with the Municipality water tap.

I: Yes yes yes. So you never go and bathe in the rivers? You have ponds and all right?

R: That is done once or twice in the year, something like that.

I: I see. Is there any link to illness from that? Any connection?

R: I don’t know.

I: Don’t know. Okay, okay. Ummm….you know what antibiotic medicine is. Can you tell the difference between antibiotic medicine and other medicines, meaning by looking…would you see a difference?

R: I don’t know…this…

I: Don’t know, okay. Can you tell me to what extent antibiotics are available- meaning to what extent it is available in the community? Like, if you want it, where can you get antibiotics from? Where all will you get antibiotics?

R: That is in general…you get it in whatever medical store is around…maybe.

[*long pause as interviewer walks to shut the door and cut off background noise*]

I: Um so you said where the medical stores are…

R: Yes.

I: Okay, I see, I see. Has it ever happened to you that somebody has given you an antibiotic…and you meaning did not get better after taking it? Meaning, do you ever take antibiotics yourself?

R: No, sometimes if I get a fever, etc., then I told you right, I’ll take Paracetemol, etc. or something if I decide to take anything.

I: I see, I see. And has the shopkeeper ever told you that you should take this medicine, or take this medicine?

R: No, never said.

I: Never said, okay.

R: If I ask, then he gives it from his heart [willingly, on his own] that this medicine…yes…

I: So generally, you tell them your illness?

R: Yes, illness…

I: What is your preference? Will you first go to a shopkeeper, RMP, or first to a doctor here?

R: [*pauses*] This…see if I have a normal…a little fever, then I go to medical, otherwise RMP. In the end, a hospital or doctor.

I: In the end? I see. And what is the main reason that you go to the doctor at the very end?

R: Over here, by coming here, first of all, there is a long line and everything. And then, after that…it takes a lot of time over here. That’s why…when there’s something big, like I think no, it would not improve if I consulted outside or that it would take a lot of money outside, then I come here [to ADH].

I: I see. And what kind of fees do the RMP take?

R: [*pauses*] Everyone has their own thing. Over in our area, some take 100 rupees, some take 50 rupees [*chuckling*].

I: 100 rupees, 50 rupees? And then on top of that, what about the medicine cost?

R: Yes on top of that [in addition to that].

I: Okay, and you give everything to the RMP- do they have medicines to give you?

R: Yes they have medicines, otherwise they also prescribe from outside.

I: Prescribe from outside? I see, I see. Okay, ummm…hmm hmm hmm…tell me this, you…if someone gives you an antibiotic, okay? Gave you a full course, saying that you have to eat it for 5 days. [*Young girl trying to get her father’s attention*- *respondent trying to pay attention*] So 2- after taking it for 2-3 days, you think you are feeling better. So do you complete all 5 doses? For 5 days?

R: Like that, I complete it…yes…I…complete it [*appears hesitant while speaking*].

I: So if you’ve been given a 5 day course, you will go to the medicine and take it for all 5 days? From them?

R: Yes.

I: And…if, meaning, if it’s a little expensive, meaning, if you are not coming to the government thing, you will have to give the money on your own.

R: Yes.

I: Then also you take all 5 doses?

R: [*hesitates and pauses*] Yeah I take it.

I: You take it, I see, I see [*smiling*]. Do you think there can be any problem from not completing the course, or any difference- ?

R: [*interrupting quickly*] It will come back!

I: It will come back. Where did you hear this from?

R: No…this I have felt as well, that many times if I have left it, then it has come back. That’s why complete…and I tell at home also to eat it completely.

I: I see. So you generally eat?

R: Yes.

I: That’s good, that’s good. And the- the- if you do not eat it, what will happen to the pills that are left? If there are some medicines left…?

R: That is left as is.

I: Left as is? You don’t throw it?

R: I throw it also, or it just remains. But I do not use it again!

I: You don’t use, so if you- you- you think the same illness is coming back, then will you, meaning, return to the doctor or use the same medicine?

R: No, I go to the doctor.

I: You go to the doctor, okay. Suppose you had a fever before. So you got a syrup right? You get a syrup from the government. So that syrup…if you get a fever next time, what will you do with it?

R: Yes…like that, I didn’t even know that you get a syrup for fever from the government. I do not even come here for fever, so…if I take it from outside, then I get a pill. [*Fussing child in the background*]. And one- [*chuckling*] I told you right I know one name of the fever tablet, that’s it.

I: That you got.

R: Yes.

I: So, do you give the whole thing to them, or [*stuttering*] do you give it until the fever goes away?

R: [*long pause*] Whatever we get from the doctor, we do not use it again.

I: Let’s talk about a few illnesses. Like you said, there is a fever. And you had said earlier, I had heard you had said there was vomiting. What other kinds of illnesses are there?

R: Yes [*hesitant*]. That is what occurs over here mostly…fever, vomiting, this…what do you call it…loose motion.

I: Loose motion is happening, okay. Anything else you have seen?

R: Anything else, something special…[*pauses*] now whoever has an illness will have it. This happens normally once or twice in the year on its own.

I: Occurs on its own, okay. Have you ever seen rashes on the skin? Like you have itching, right?

R: Yes.

I: Have you ever seen that?

R: No, in our area…

I: Not there, okay. Umm…what is your expectation, meaning when you go to the doctor [*child talking to father*], when you come to the doctor, what do you wish from the doctor? Meaning what all, this…

R: Just that we get…proper time. [*Father interrupts to entertain his child*]

I: Yes, so what is your expectation? What do you think- what will you get from the doctor?

R: Like here, like that…just that whatever I am telling about, I get proper information about it, and gets treated correctly, that’s it. What else?

I: How do you know whether this is correct?

R: No this we will not find out, because we do not have knowledge about this. We go based on whatever the doctor says.

I: Now, tell me one thing- if I am a doctor, you are coming to me. You think- you have been sick for 2-3 days, okay? You have a cold, you have a fever…now I am the doctor, and I will tell you that you drink water, rest, and you don’t need anything else. You…if you want to eat a Paracetemol, you can eat it. You don’t need anything else. Then how will you feel?

R: I will try it out and see.

I: Okay. So meaning you won’t tell the doctor to…prescribe a medicine?

R: No, like that I don’t say. When he is saying, I will try it out and see. If it doesn’t work, then I’ll come back 2-3 times.

I: You’ll come back. And generally, the RMP doctors that you have…do they give you…meaning do they always give you medicine, or do they also say like you should rest and it will go away on its own?

R: They do not say like this. They always prescribe medicine.

I: Prescribe medicine…have you ever noticed what all medicines are prescribed in that?

R: No.

I: No? No. So you save the prescription…or not?

R: The prescription is kept at home.

I: Okay but you haven’t noticed what is written, okay, okay. Umm…any negative effects of medicine, meaning any risk- are you…able to understand? Meaning any…

R: Yeah like that I know that you shouldn’t take medicine without seeing the doctor, otherwise it can have a reaction. All this.

I: I see. Have you ever noticed any reaction? Have you seen anything?

R: No like that- like that, I haven’t done anything. I haven’t…noticed anything.

I: I see. That’s good. Okay, so umm…what other expectations do you have from the doctor? One thing is that they prescribe the right medicine.

R: Yes!

I: Anything else?

R: Doctors…are right only. It’s not like doctors are wrong. The doctors are proper, but the system should be improved a little.

I: Such as?

R: Such as like there is such a long line- there should be some more counters. Some…time should be increased. Like doctors all- 10 is the reporting time and the doctor comes at noon. If he had come at 10, then maybe the line wouldn’t be as long. If the system improved a little, it would be good.

I: I see. By the way, what do you do? I hadn’t asked- your?

R: Well, I have a transporting business. I have just started it- not started it, meaning I have been running it for 1.5-2 years.

I: I see. Here, meaning in this area only?

R: Yes, in this area only. It operates in the local. Before that, I was studying.

I: You were studying. What were you studying?

R: Economics…from BC college. Bardhaman University.

I: So what is your highest level of education?

R: Honors.

I: Honors?

R: Yes.

I: That’s very good. I had one question- uh, when you say this doctor-doctor, that doctor should do this, doctor should do that…which doctor are you talking about in this? Meaning, the doctor who sits here [ADH] or the local doctor?

R: Local doctor, see, he is taking money from you, so he definitely gives you his time. And here you will have to…struggle a bit to get seen. There’s a line, etc., and many other…and meaning, the time is also not right. For everything, you are first standing in line to take a ticket, second time standing in line to see a doctor, third time standing in line to take medicine. Meaning, if you have come once, you can assume you’ll be here for 4-5 hours of your whole day.

I: Hmm hmm hmm. That’s true. And if you go to someone local, then?

R: Why, you’ve been to their place, at most it would take 15 minutes. They will also see you for 10 minutes. And the same doctors that work here give you time when they sit outside [in private practice].

I: I see. So how do you know whether the doctors who are sitting outside are doing the right thing or wrong thing?

R: I do not know this, Sir.

I: Okay have you ever- RMP doctor and the MBBS doctor- have you ever noticed a difference between them?

R: Noticed- like, RMP doctor…normally…tell us, we try and do it. If they don’t understand, then they…

I: Refer?

R: Yes.

I: I see, I see. So this doctor that you normally go to- are they generally MBBS doctor…RMP doctor?

R: *[pause].* Like if it’s very small, cough and cold, then an RMP doctor, otherwise we see an MBBS doctor.

I: I see, I see. Okay. So, do you ever feel like this doctor is not working for me- you went once, gave medicine, did not work- so what do you do then?

R: So then the same thing- go outside and visit- I go and show again [elsewhere in private]. Like over here, one time I had…food poisoning. So…I had gotten better, but along with that, I had developed a cough. Meaning that got better and then I got a cough. So I come here [to ADH] and I showed it at the counter. Long back, I showed the RMP doctor, and it wasn’t cured. Then, we- came here, and they had prescribed a syrup, etc., medicine and all- it still wasn’t getting cured. But it didn’t stop, so then I paid fees outside- he’s an MBBS doctor only. There’s SAIL hospital in Burnpur right? He sits just outside that. So when I showed him, so then…I am better.

I: And what would your preference be? First, like you said, would you first go to the shopkeeper or RMP- some small?

R: Well…like that we not prefer going to any shopkeeper that much [*child talking to parent*]…we go to the RMP only, but many go. They go first to the shopkeeper only.

I: And you’ll come here last? At the end?

R: Yes. Like last what, I mean, we see that it’s big- won’t improve by showing it outside. It feels like no, we should admit in the hospital, then we come and bring.

I: How often must you change doctors? Like you said you are going to an RMP and it’s- it’s not getting better…

R: See like…I see the situation and how it is. If I think that no it’s a big problem, then I show it directly to an MBBS doctor. And otherwise, like no it would be fixed with a normal medicine, then RMP is fine.

I: Okay, okay, okay, okay. Uhh…have you ever heard about antibiotics being used outside, like meaning, antibiotics being used in farming? Have you ever heard anything about this? In the environment?

R: That much I do not know.

I: Okay. Sometimes there is chickens- poultry- may be used in poultry sometimes. Or in the farm, it can be used…

R: Yes this I have heard! That in farms, urea etc. are used which is not right. And I did not have knowledge that antibiotics are used.

I: What would you want to say to improve the health situation? Meaning, if I give you the mic, and I ask you- what can we do to improve?

R: To improve, see, the doctors over here are…okay. They are capable- it’s not like doctor or nurse…the system is such that they are not able to work. Or…like one doctor has so many patients that what would the poor chap do? He will only give 2 minutes. If he gave 10 minutes, it will be night-time. That’s why meaning, if the system changes, then something can happen. Otherwise, like this…

I: So what can the government do for this?

R: The government will have to see that when so much money comes for healthcare from top, meaning where is it used? It’s not being used, na? Where it is going, how it is happening, what is happening- meaning, even then people, I mean, the situation here has improved a lot in the past 4-5 years.

I: What have you seen?

R: Like this hospital which was newly built. And others, I mean, many other- outside also things are fine, like this store here in front has 57% discounted prices for medicines. So all of these things have come about mostly in the past 4-5 years. Earlier, this was not there. Earlier, it was that old hospital- used to operate however it was. So this is what it is...all things…you know right, politics is involved in everything, meaning in everything…anything…so this is what it is. If it is used properly, then it’s good.

I: Anything else you would like to say?

I have a last question…uh, which is that if someone gives you medicine- if it’s an RMP, medicine over here [*child making a ton of noise*], so does anyone explain how it needs to be taken, how much needs to be taken…does anyone explain like that? [*recording paused* *due to child’s noises- took a break*]

So if someone writes a prescription and the shopkeeper gives you medicine, so do they explain how you should take it, how much you should take…?

R: Yes, they explain!

I: Who explains it well to you, the medicine shop?

R: No, both, both explain. Like they have prescribed it…the doctor says that you have to take it at these times, and the medicine shop folks also say that I have to take it after dinner or in the morning. They tell me.

I: And here?

R: Over here, the people who give medicine…they…tell us, the ones outside tell us.

I: Does the doctor tell you or not, though?

R: Here, the doctor…[*long pause; hesitation*] prescribes. If he tells tomorrow, he tells; if he doesn’t, he doesn’t.

I: I see. Meaning the pharmacist folks normally tell you?

R: Yes.

I: When you go to get medicine?

R: Yes…where we get medicine for free, there is a long line.

I: I see, I see. Do you ever do something where the doctor gives you a prescription, here okay? Here, and tells you that you should go and take it from outside over there [in ADH] and the line is big, so you took it from outside? Does this also happen? Have you taken from private ever?

R: No, what happens if you take it from private…whichever pharmacy the doctor is sitting in, his medicine will only be available there. If you go look for it elsewhere, it will not be available. Mostly this is what happens- and many doctors are also such that to visit, first you go and get a number and there he’ll take 50-100 rupees from you. Then…he…meaning the pharmacy person will take it from you. Then he says take medicine and it will be cut. And with the doctor’s fees…this thing is there! Like you did a test or sonography from outside, that you should go over there or you go to the Medical, so like that…you go and get it done there. This happens…so they have their commission in that also. Yes, like if a doctor from here refers with a prescription, so his commission is kept over there that…he’s come from there, so this thing happens.

I: Do doctors from here ever tell you that you should go to his private clinic? To him?

R: No the doctors here don’t say that. Like that well, I have not come many times. But however many times I have come, they have never asked me to come to his private clinic.

I: I see, I see.

R: This thing is not there.

I: So does anyone else say that over here? Any staff?

R: No no no. No one says that…yes, but one thing I have heard. A few days back, I had admitted my mother- for the same, loose motion and vomiting. So what happened…I was standing right over there, and there was a man…I think he wanted to get a photo done or something…I don’t know…the nurse said it will be done in the morning here. Go, get it done outside. She was referring somebody, meaning you go outside, go do it outside and come here. So this thing is there…that…they send outside, even though the facility is here and they can get it done here. Even then, they say to go get it done outside.

I: I see.

R: This thing happens over here.

I: One last question I would like to ask…you, meaning, do you also believe in Ayurveda and Homeopathy?

R: Homeopathy, well, mostly we do not use. As good as never. Ayurvedia…some things such as…what do you say…food, that we use, like there is a product- Chawanprash and all we use- soap, etc. I have used a bit. And more…meaning general things that are used around the house, Ayurvedic and other things [*child starts screaming again*]. Meaning not like medicine.

I: Okay okay ji [*chuckling at child*] thank you so much!
