# Supplementary material for: “Without antibiotics, I cannot treat”: A qualitative study of antibiotic use in Paschim Bardhaman district of West Bengal, India
Source: PLoS One. 2019 Jun 27;14(6):e0219002. doi: 10.1371/journal.pone.0219002 (PMC6597109; doi:10.1371/journal.pone.0219002)
Supplement: S2 File — (ZIP) [file pone.0219002.s002.zip › S2_Transcripts/V2.doc]

**V2**

I: Where is your house?

R: Over here, my permanent address is Sukantapally.

I: Who is in the house?

R: Well, I just came back after 5 days from Chapra, Bihar. I had gone for 7 days, so I just got back tonight at 3 am. Today was my wife’s last checkup in the hospital on the 15^th^. So I just brought her- and otherwise- now, there is no one in the house. At 2 pm, my father will come back from duty. And then my wife and my daughter.

I: I see. So, uh, mostly what all- what kinds of illnesses do you see in your house?

R: My father, approximately, has 3-4 permanent illnesses: sugar, pressure- recently, I just spent 2 lakhs in Chennai to see you know what they call glaucoma in the eye? I did that and came- it’s coming up to one month since then now. And my mother has pressure [blood pressure], no sugar [problem], and I think that I have- my heart has a problem.

I: In the kids?

R: In kids- she has nothing, she has just turned 5, so she gets pneumonia, cold, fever, cough- things like that. That happens.

I: OK. So when cold, fever, and this happens to your child, or to anyone, what all do you do?

R: Like when she has a cold or fever normally, we take her to the doctor- not the main doctor like your medical- there is someone we have, called Kallu- we go there only. He gives us syrup etc. Medicine for 100-150 rupees. She gets better with that. If she doesn’t get better with that, then we have to go and show the doctor...

I: This doctor that you go to- is it just any doctor?

R: Any doctor, yes. Meaning that there isn’t…MBBS or anything here. Normal, as people go, taking medicine for 5 rupees or 10 rupees. That’s it.

I: You get better with just that?

R: If I get better with just that, then okay, otherwise people go to the doctor [MBBS doctor]. But mostly people get better from just that. Recently, our…it’s ward 21…so in ward 21 over there, a health centre is there- has opened. There is a doctor- for the past two months, that is where I have taken my child. It’s a government hospital. The doctor over there sees it. After looking, he will prescribe a medicine, syrup, etc. Then, I am seeing that my daughter is getting better from his medicine too.

I: So who do you go to more often- the local doctor or?

R: [*interrupting*] Yes, local doctor. If it doesn’t get better with him, then the doctor.

I: I see. So what is the reason for going to him?

R: There is no reason to go, but people do not go directly to the doctor. He sees if he can get better in 10-50 rupees, then great. After that, a person goes to the doctor. This is the thing- nothing else.

I: But this health centre that is there, or this district hospital- this happens for free.

R: Yes, but we are not able to come because it is far…[*pause*].

I: I see. Have you heard of antibiotics?

R: Antibiotic means cold, cough, fever…all this.

I: Yes, so antibiotic medicine- have you ever heard anything about it? Do you know- know anything about it?

R: I do not know anything about this.

I: Have you heard from anyone else about antibiotics?

R: No.

I: Okay. So when you go to medical, and you go to the doctor, do you say the name of a medicine or does he give medicine on his own? How does it happen?

R: If I have been eating medicine from before, then I will have to show him the medicine slip or…I’ll have to show him. If I haven’t eaten, gone directly to the doctor, then I’ll say there has been no treatment yet, nor have I had any medicine- I have just come to you. And whatever he prescribes from his opinion. [*pauses*] People go to the doctor with hopes. Now, according to that, whatever medicine we get, we will accept that.

I: What do you think before going to the doctor?

R: At the doctor, I think just that- if I have fever, cold, cough, then it will not get better at home. Whatever medicine I will have to take. According to this, a person goes to the doctor.

I: Do you go on the first day of illness or some days later…?

R: No, some days- like 2-3 days- like I will first take medicine from a normal medical shop. If it doesn’t get better from that, then we go to a doctor.

I: [*long pause*] When you go the medical, what do you say?

R: When I go over there, I just say…I have a fever. I also have a cold and cough. So that- what do you call it- he will bring the thara-thermometer and check how high my temperature is. Then, after measuring, he will then give his medicine.

I: [*pauses*] So do you just say the name ‘fever’ or do you also take some prescription along with you?

R: No, meaning, whatever problem has happened, like fever or cough, that is all I will say. And he asks how many days I have had it- how many days- so I tell him 2 days- 3 days. That’s it- according to this, he gives his medicine.

I: I see. So your- the medicine which you take- how do you take the full course?

R: Like they have given 6 pieces. Take it, it’s a 2 day course. Morning, evening and afternoon- eat it today and tomorrow. So we eat it…for two days. [*phone ringing in background*] If he gives a two day course, I will eat it…then see, if he gave for 2 days, and we see that it got better after 1.5 days…there are a lot of people who would just leave it from that day and not finish the full course.

I: What do you do?

R: Well, I eat the medicine. I used to leave it earlier, when I used to see that my fever has gotten better, I used to leave. Now recently, since the last 4 years, I have neither gotten a fever, nor a cold or a cough or anything.

I: At home? Your daughter?

R: At home, it keeps happening. It keeps happening with my daughter every 2 months or one month. I have to take her with me to the health hospital.

I: So that medicine is left over?

R: Yes, that medicine is left over. She is not able to eat all of it. If she’s gotten better, then what is the use of eating the medicine?

I: What do you do with the medicine afterward?

R: Afterward, the medicine passes its expiry date, then what- then if it comes again [if she gets ill again] within 5-10 days, then I feed her the same medicine.

I: By asking the doctor, or?

R: On my own. I know that he had said give her half of this, give her that- so if in one week, her fever goes away- the medicine is left over, there is a lot of medicine- so then, she gets it again in one week, so then according to that, we…give it to her again.

I: And uh, the local doctor who you go to, what medicine- does he just give you medicine or does he prescribe something too?

R: No he gives medicine on his own.

I: Does he prescribe anything or?

R: No, nothing. He says that if it hasn’t gotten better after 3 days of giving it, come back to me.

I: I see. What kind of fees does he take?

R: He doesn’t take any fees. This is a normal, pharmacy shop. If I go to the doctor, then he will take fees and give the medicine. He doesn’t take any fees- in 40-50 rupees, he will give you medicine for fever and cold.

I: I see. Okay, so do you ever- you go to the hospital at the very end here, meaning-?

R: [*interrupting*] Yes as the last reason.

I: Last, okay. So what all things do you think you come to the hospital for? Which time?

R: Which time? [*pause*] I don’t understand.

I: When do you think about coming to the hospital? In which circumstances…

R: Oh, when. When it’s the last step, when the doctor also fails and the medical shop also fails, and my- these are my only two options- in that position, we think about admitting to the hospital.

I: So do they refer you or do you come on your own?

R: They do not refer, I have to come on my own. Have to come on my own.

I: [*pauses*] Okay, uh, if you go to the doctor…okay? So, it did not get better with one doctor’s medicine. It didn’t suit you. Then, what do you do?

R: So then I look for a good doctor elsewhere. Go there. This has happened to me once- I showed a doctor and I did not get better there. Then, we, then went to a different doctor. I told him the full process- showed this doctor, like this, like that, took his medicine for 4 days, 5 days, did not get better, that didn’t happen- I told him about the process, and then he gave me medicine according to him. Then by eating his medicine, I felt better.

I: How many doctors do you change like this on average if you don’t get better?

R: No see, that much we- we are educated, not like that- I see once or twice. If it doesn’t happen with that, then I come directly to the hospital. Here, I have some linkages in this hospital. The work gets done more easily. It’s not that much trouble- stand over there, get a number in a line, do this, do that- it’s not like that. That’s the thing.

I: So…everything here is free. Even then, why do you come to the hospital at the very end? If you could say a little…there is some reason, so some- ?

R: [*interrupting*] Yes, it’s like that the hospital is a bit far from our house. And the second thing is we think- what…we’ll get free medicine for 10-50 rupees, let’s just take it here from the medical shop. Why go for so little to the hospital? If you’re going to the hospital, means something big- something big happened- would go for that. For this reason only, we go and take medicine from outside.

I: And how do you find the treatment at the hospital?

R: Here? Here, the facilities are okay. The situation has improved from before. It’s okay here. Sometimes, they put more than one person on the bed. Hmm.

I: Anything more?

R: No other problem. Now see, wherever you go without money, you will have to bear a little trouble. That’s the thing. It takes more time. We come at 10 am, and now it’s looking like I’ll only be able to go back after 2 pm. Give some time. If you won’t give money, you’ll have to some time at least. And this we did not know about- she [daughter] just turned 3- so we used to get her check-up done from outside. A lot of money was spent then. Then later, we found out about this hospital, so now we get it done here.

I: I see- you didn’t know about the hospital at all?

R: No, I knew there was a hospital, but we knew there are 100 people there usually- how would we go- they’ll make us run around, go here, go there- that’s why we didn’t used to come.

I: When you go for any illness to the doctor, when they give medicine or prescribe medicine, so do you ask what all medicines have been given? Why they have given it?

R: What reason, meaning, they must have given it for whatever illness I have.

I: So do you ask the name of the medicine or anything?

R: The name of the medicine, etc., the doctor…some…like a doctor is sitting next door here there’s a medical [shop]- he prescribed the medicine to you, and he’ll say go take it from next door- then, we take it from next door.

I: Meaning has he given an antibiotic or not, do you ever ask that?

R: No.

I: Okay, the- what you get from the hospital, all- are you able to get everything he prescribes from here?

R: No I do not believe in the medicine provided by the hospital.

I: Why?

R: Because, our- our village, right- all of our relatives who are there, uncle, brother, etc. are in the army- they are in the medical unit of the army. So they bring the medicine from there. Cold, cough, fever- hordes of it. They throw it away also- the medicine does not heal. What do you say? Like rubbish- it does not any power, it does not have any resistance [*incorrectly using this term*]- the medicine- meaning it doesn’t have that thing- that’s why we do not believe in the hospital’s medicine. We take it from outside.

I: Have you ever tried eating it, whether it works or not?

R: I have tried to eat it once or twice. I get no effect from it.

I: Nothing happens?

R: Hmm [yes].

I: I see. So when you go to the medical- whatever the doctor writes from here- do you show the prescription or do you just say outside that it’s this and - ?

R: [*interrupting*] If we go to medical, then medical only gives us medicine- does not prescribe.

I: No, no, if a doctor here prescribes a medicine- the doctor prescribes something here, right-

R: Yes he prescribes.

I: So what do you with that medicine?

R: I bring that medicine.

I: From outside?

R: Like my wife has, so the wife’s medicine is ongoing. The doctor prescribed and we have brought the medicine and kept it. And the course is ongoing.

I: You get that from outside too?

R: Yes…no some medicines you do get here, but others they say no go take it from outside. So those medicines we take from outside, whatever we can’t get.

I: Do you ever go and say ‘give me more medicine, give me medicine with more power’? Do you ever say anything like that?

R: What will someone say in a government hospital?

I: In the medicine shop or wherever you show outside?

R: Yes, I say! Give me some good medicine. It hasn’t gotten better from this medicine- this time, give me some good medicine.

I: What is good medicine according to you?

R: Meaning, the medicine you gave did not work- what kind of medicine did you give- give me better medicine than that! So they give according to them.

I: Okay, so when the medical shop gives medicines, do they ever tell you that this medicine should be eaten this many times, this many times in the day?

R: They explain and give. Gave for 2 days- gave for 2.5 days- so they will tell us and say…that the medicine is for this many days, 3 days, 2 days- according to that, the person takes the medicine.

I: Does he ever say that this is an antibiotic? Does he say anything- anything like that?

R: No. Sometimes, maybe in some problems, they say…antibiotic.

I: I see. So then, do you eat it completely as he instructs? What do you do?

R: Yes I eat it completely.

I: What do you think- why should you eat it completely?

R: Completely means, we think that maybe it’s the course, that’s why he has given the medicine- and…until he gets better, the medicine should…

I: And if he gets better, then?

[*long pause*]

He gave you medicine for 5 days, but you got better in 3 days. What would you do then?

R: I normally leave it then, but my mom forcefully makes me eat the remaining two days and says no, eat it.

I: What do you do about the child?

R: The child?

I: What you just said- 5 days, 3 days. So what do you do- if the child?

R: [*interrupting*] No I give it to her completely, however many days the doctor says. I give it to her.

I: If some illness happens- who do you find it easiest to go to? Who do you prefer going to- a local doctor or any MBBS doctor?

R: If there is any special illness, then a person will go to the doctor. Any good doctor.

I: A special illness, meaning?

R: Meaning aside from this cold and cough. And other than that, if there is some serious illness. Then, I’ll have to go to the doctor. Can you give one or two examples of illnesses for which you would go to the doctor?

*[pause]*

Meaning, you are saying right, some serious illness- for which illness do you go to the doctor?

R: Like there is no vitality left in the body-, there is paresthesia at joint. There is a lack of water, a lack of blood- something doesn’t feel good- I’m sleepy or something else- in this position, a person would go to the doctor.

I: I see. What would you do if you have loose motion?

R: If I have loose motion, then I’ll go and take it from a normal doctor.

I: I see. Normal doctor meaning chemist shop?

R: Yes, chemist shop.

I: Okay. Do you go to homeopathy or allopathy?

R: I go to Allopathy.

I: Why, why so?

R: I have heard that homeopathy takes a long time to get cured. That’s why-

I: It shows an effect after a long time?

R: Yes, after a long time. And here- means I have an illness, so it would be nice if it got treated quickly.

I: Okay, uh, the medicine that is left over- you had said that you leave the medicine after eating for 3 days- the medicine that is left over with you- so how do you use that then?

R: That medicine, if it is known to me, so then if a similar illness is coming within 5 days in my house, I give that medicine. Yes- and if it doesn’t come, then it gets expired and discarded.

I: I see and you check the expiry date?

R: Yes I keep the medicines in a box at home- there is a type of box- I keep this Dispirin, Aspirin, etc, 5 medicines, in that is there at any given time.

I: What all medicines do you keep?

R: There is medicine for vomiting, pressure, sugar, then there is 1-2 medicine for fever, 6-7 types of medicines are there. Whomever I give it to, I check [the medicine] before giving.

I: What you just said- that you give leftover medicine to other folks too- the- medicine for older-

R: [*interrupting loudly*] When I give leftover medicine to anyone, like, if her medicine [pointing to daughter] is left over, and something is wrong with me, then I won’t eat it- why, because her age is different and my age is different. My wife and I are the same, only apart by 1-2 years. So that I give. But not that someone is aged and I give her medicine to a child. That much I understand.

I: I see. What are your views on antibiotic use? Meaning, what do you think?

R: About antibiotics…all I understand is that antibiotic means fever, cough, cold happens- that’s it- I don’t know anything more.

I: What do you think about its use- that it gets better fast or by not using it- what do you think, what can happen?

R: By eating antibiotic?

I: Yes.

R: No, I do not have that much knowledge.

I: No, meaning, when it happens to you- what do you think that by eating this, do you get better faster? Antibiotic-

R: No it’s not like that…[*pauses*]

I: When was the last time you or anyone in the house got antibiotics? Do you remember? Were you ever given like that? Do you remember? Doctor…

R: Antibiotic…perhaps the doctor had given it to her only [*referring to his daughter*]. Her…[*trailing off*]

I: Uh, so have you ever thought that you need an antibiotic and the doctor isn’t giving it to you? Have you ever thought that? That he isn’t giving good medicine- what you, like you said, not giving medicine- have you ever thought that?

R: Like that, it happened once with me. I had gone to 2-3 places to show a doctor, but even then my fever…was not getting better. So at last, I said I will have to go to the hospital. So then I had a friend… he said no no, there’s a doctor here you go to him, and then at last, you go to the hospital. Then, I went to the doctor, I told him all…this is the whole process, these doctors I changed, these medicines I ate…and I’ve come to you, and my condition is not improving for one week- 10 days. Now give me some solution. Some way. So he listened and saw everything, and then he prescribed his medicine. He said drop all medicines, and use my medicine for 3 days. I ate his medicine and really I…got better.

I: I see. In your opinions, what things should be improved in this hospital? For treatment?

R: What all should be better?

I: Meaning, what all should happen- such that you come here first and don’t go outside?

R: Now, I am seeing approximately all facilities at the hospital. Going somewhere else from here…now if the doctor refers me, that’s another thing. But approximately everything a person needs, all facilities are here.

I: No meaning, what I meant to ask is…what all facilities should be here so that first you won’t go outside, but you will first come here? What all facilities do you think should be present here?

R: Facilities meaning like the medicine…like I have come to the hospital…you may not even know whether you get medicine here or not. Like I have a fever, will you get medicine here? [*asking the interviewer*] Where will you get?

I: Here, in any hospital, you will go, the doctor will see. There, there is something called a pharmacy. It's written as “dispensary.” You will show your slip there, and you will get medicine free of cost.

R: No no, not the slip. Like I already have a fever. I want a medicine from medical, so I take from government hospital. So after coming here, where will I have to go? What do I have to do? Like you said the medicine is not good…

I: First, you will have to- the OPD that is there where the doctor sits, every day the doctor comes- you have to show over there. Okay? You have to get a slip, make a slip. Then you have to take that slip to the doctor. The doctor will see you, see and prescribe a medicine. And that medicine you will get from the hospital. Without seeing the doctor, you will not get it.

R: Oh, even if there is a normal, little bit of fever, then also the doctor will see? [*Interviewer nodding*].

I: Okay? So that is how the medicine is given over here. So uh, you have already said that for cold, cough, fever, there should be antibiotic. [*nodding*]. So tell me this, that, if antibiotic…uh…if you do not want to use antibiotic, then what will you do?

R: If I do not want to use antibiotic, then? [*interviewer nodding, long pause*]. See, how will I say this? Whatever the doctor says, that you have to eat this along with the antibiotic, then I’ll have to eat it with that. It won’t happen based on what I say.

I: No what I’m saying is- you took the medicine from the medical [chemist shop]. Okay? You took an antibiotic. So do you ever think that no, I should not have eaten this medicine right now? Have you ever felt this way?

R: No I have not felt this way.

I: Okay. Do you feel that antibiotics are overused by us- and you use in the home? Have you ever felt this way?

R:[*long pause*] We are using a lot of antibiotics?

I: Have you ever felt this way?

R: No.

I: Do you ever feel that a medicine is not working, or, meaning…the medicine might also be bad for the body. Do you ever feel this way? Have you heard this? [*pause*] That there may be a bad effect?

R: It has not happened with me, but…it must have happened with some person.

I: Have you ever heard about this?

R: Yes I have heard that…he [referring to someone random] had gotten lots of medicines…he used to think that it isn’t suiting him and the doctor is playing a game with him or something. This kid used to say something like that…that he did not prescribe a medicine for the problem that I have. Prescribed for something else. That’s what he used to say to me. [I said] it cannot be like that! There is a friend I have in the neighbourhood…[*trailing off*]

I: Do medicines have any bad effect, like, have you ever heard this?

R: Yes, I have heard about reaction, that a medicine can do like this.

I: Like?

R: Like after eating it, you’re feeling anxious or there will be itching. Like that. Reaction, like people say, it happened from eating the medicine. [*Pauses*]

I: So when you go and show it, and uh, when uh, you take medicine from the medical. When they tell you to eat the medicine like this and that, do they also tell you the reasons why they are giving you the medicine?

R: No they do not go into so much detail. There is a big crowd- whatever problem I told him, he took out medicine from his office and gave it…doesn’t ask clearly that it’s been having for so many days, what problems do you have, this medicine I have given for this reason- he does not say that in this much detail. No medical shop will tell you. He will just explain your problem; give you medicine, that’s it- go. Now if you go to the doctor, then you there will be discussion, doctors take time also, if you to medicine shop there is number of 10 people he will try to clear his shop, give medicine and make it clear.

I: What do you think- in this society and where you stay, among you- how much knowledge is there about antibiotics among people?

R: Antibiotic, mostly, people don’t have much knowledge. Don’t know much…a few educated people are the ones who may know, but they also may not know properly.

I: And…what do you think…what should be done? If we have to explain the proper uses and talk to them about it, what all should we do?

R: About antibiotics?

I: Yes, about proper use.

R: Just like that, like you have called me. [25:45-25:50]. Meaning if you want to make the public aware about antibiotics, then you will have to do work like this.

I: Like what- can you tell me in more detail?

R: Like now, how you have brought me here. Like this, other people- meaning, it will take time! For some days, you call people and share this knowledge with them, put some apps in mobiles, put some information in local channels on TV. If you do like this…even if 10-20% become aware of antibiotics, it isn’t small. This will also…[*trailing off*] you will have to put in some work, your branch.

I: Anything else…you think that by doing this, the community will live much better?

R: What other ways…you can also roam streets, neighborhoods with a car.

I-For promotion?

R-For promotion, these are rules means to give information to people. Other thing I don’t think .

I: Have you heard about the wrong uses of medicine?

R:Yes wrong use of medicines means there is 10, Nitrozen 10, that is used as drug by people at our neighbourhood . did you hear about Nitrozen 10?

I: They take?

R: That all is given to the patient for sleeping. I also was addicted for few days. So what happens when you eat it is that…it is given for sleep to the patient- he will eat it and sleep automatically you get sleep. And the boys use it as a high. If you have eaten that and you are awake, you are high…if you are awake after eating it, you are high.

I: And the normal medicines which we have, like antibiotic, have you ever heard of incorrect uses of this?

R: No.

I: You haven’t heard. Do we normally misuse medicine in the home? Normally, not to get high. Anything else?

R: No, what misuse- if you have some illnesses, whatever medicine is for that, you should eat it. How will you eat other medicine? You will have that fear- that something might happen. Shouldn’t have a reaction- shouldn’t die- this comes in people’s heads and that’s why they don’t eat it.

I: Okay my last question- when you go to the doctor and medical, what expectation do you go with?

R: All people go in hoping that they get better with the medicine. Now, if they don’t get better over there, then a person takes…then people take other step to go to doctor. Normally this happens in every household. That if you have a fever- if it’s cold and fever, you go to a shop or a normal medicine shop. You see they are saving it. Around 70% of people at home do this. If they have a fever, cold, cough- I’m telling you for all these “chota-mota” illnesses. So normally, they go to a chemist shop. If they don’t get better from there, then show a doctor or specialist. If you don’t get better, don’t feel good, then you get admitted in the hospital. If you don’t get better from the hospital, our people will do something like they will discuss themselves and will refer to other place.

I: In your neighbourhood, how many people out of 10 go right away to a medical shop?

R: In our neighbourhood? In our neighbourhood, about 60% people go to a medical shop when they have a cold or fever. Do not go to the doctor. Yes, if they do not get better from there- from the chemist shop- then they go to the doctor! The main thing is a person also thinks about money! If we go to a doctor, you need at least 500 rupees to go to a doctor. No doctor has fees of 200-300 rupees. Okay? On top of that, he’ll also prescribe medicines for 400-500. If it’ll be above 500, then someone will go to the doctor. That’s why a person does not go to the doctor in the first step. Thinks that he’ll go to the chemist shop and if he gets better, it’ll be good. Now if he doesn’t get better, then they are forced to go to the doctor. Then he goes…to the doctor.

I: Okay you mentioned priest- do you go to the priest?

R: Priest meaning?

I: You said right that you go to the doctor- priest – [*other interviewer explains he was referring to fees*]. Ah, fees. But do you go to a priest or anything?

R: No, no one in our family believes in all that. We do not go for all that.

I: In your neighbourhood?

R: There are one or two in our neighbourhood- his there are still people like that. That- if a fever comes, it’s because a ghost has come, hit him, he is increasing his fever more- hitting, the smoke that comes from the pepper.

I: Snake bite.

R: Yes, if a snake bites, then he won’t go to medical or hospital, but he will go to get it [30:52]. There are still people like that.

I: You don’t believe in that?

R: Yes, we don’t believe in this stuff- ghosts, souls, don’t go in this street, this house has a ghost- we don’t believe in all this, we say it’s all lies.

I: Okay…do you remember any names of medicines that you used, for the kids or for elderly?

R: We eat medicines anytime for 2 days like you have Dispirin, there is a tablet for headache I think- that’s there.

I: Any antibiotic medicine?

R: No.

I: You don’t remember the name. Okay.
