# Supplementary material for: “Without antibiotics, I cannot treat”: A qualitative study of antibiotic use in Paschim Bardhaman district of West Bengal, India
Source: PLoS One. 2019 Jun 27;14(6):e0219002. doi: 10.1371/journal.pone.0219002 (PMC6597109; doi:10.1371/journal.pone.0219002)
Supplement: S2 File — (ZIP) [file pone.0219002.s002.zip › S2_Transcripts/V3.doc]

**Interview- V3**

I: Where are you from?

R: Chinakuri.

I: What is your profession?

R: Auto driver.

I: How many members at your home?

R: One son, one girl and wife.

I: What is your age?

R: 50 years

I: Could you please tell me what kind of diseases are mostly occur at your home?

R: Now problem of gas is much. At my house everyone is affected by Gas.

I: For children.

R: For children cold and cough.

I: Tell me more

R: Cold, cough, and fever. I don’t know more. They are not adult.

I: What do you do in case of cold and cough?

R: We go to the doctor.

I: Which doctor?

R: In Chinakuri there is a doctor A Kumar. We go there. Also in Bikash pharmacy many doctors sit there. Like Sadhu. We also visit there. In emergency we follow the medicine of previous doctor’s prescription.

I: Does the doctors are MBBS are local doctors?

R: No, MBBS doctors.

I: Are they passed doctors? [In reference of MBBS]

R: Yes. They are passed doctors. [In reference of MBBS] Only the shop keeper of the medicine is not passed but became experienced as he was giving medicine from long time.

I: Where do go first if any illness occurs at home?

R: We go to the big doctor [in reference of MBBS doctor]. Like our A Kumar. He sits at the Maithan more and Kalyaneswari more. He is a MBBS doctor.

I: Don’t you go to the clinic where the treatment is free?

R: Yes. We also go there.

I: Do you visit there at the end?

R: No, we don’t go at the end. Like our LM hospital [in reference of Asansol district hospital], we go there in emergency. We are poor people. If we can’t pay money at the time of emergency then we go to the LM hospital [in reference of Asansol district hospital]. This is the fact. Those have money [didn’t complete the sentence]. You don’t have money all the time. If someone has diarrhea or vomiting then we have to go to the LM hospital. Those have money they go to the private hospital.

I: In which hospital do you go?

R: LM hospital. [In reference of Asansol district hospital] Asansol. We also come to the sodepur health center. Every time we come to the sodepur health center. If there is any delivery patient or any other type illness we visit here in the health center.

I: Does the doctor is available every time?

R: The present doctor sits here thrice or 4 days in a week. We don’t come here everyday. We always get him at the time of our visit.

I: The clinic has a fix time. If something happen after that then where do you go?

R: Then we go to the private. Many nursing homes are available here. MBBS doctors are also available with so many nursing homes. Like in Niyamatpur there is a nursing home run by Akash doctor. He has two nursing homes. One is at Chittaranjan road and another one is in Niyamatpur.

I: So you go there.

R: There was Jibita nursing home that has been closed now. Hansda is available there. We send the patient there.

I: Where do you get better treatment?

R: Good treatment means where I go will get good treatment. There is no such thing that here I get good treatment and there don’t. But in my family personal family doctor is A Kumar.

I: Is he family doctor?

R: Yes family doctor. We have faith on him also we feel comfortable with him.

I: Where do you like to go, private doctor or free health center?

R: Free health center means LM hospital [referring to Asansol District Hospital]. They mostly send the patients to the outside medical shop. The only facility we get that the bed charge is free. Also we don’t need to pay doctor’s fees. But we have to buy medicine from outside.

I: Medicine is available there [Referring to ADH]

R: Not all the time we get medicine. You don’t get all the medicine in the health center. You don’t get all the medicines in any government hospitals. Some medicines are available and some medicines are not.

I: According to you what are reasons of cold and cough?

R: It is cause of water. If the weather is bad like the weather is changing now. In three weathers most are illnesses occur. One time is monsoon, another time is winter and other one is summer. In this three times the illness like cold and cough will occur. During the weather change it mostly happens.

I: Anything else

R: What else. [Smiling]

I: Do you know any other reasons?

R: I know about these three weathers. Also for stomach pain if you don’t take food then gas will happen. Like most of the people are affected by diabetes. Sugar patients. Why most of the patients are affected by sugar? Now all the foods are infected by Salfet. [types of fertilizer]. You are taking the vegetable of salfet, rice of salfet. Where do you get the vitamin? For this salfet more 50-70% people are affected by sugar. Sugar and heart disease.

I: When you visit to the doctor do they write prescription or give the medicine directly?

R: No, first they send us for blood check. Then he prescribes medicines. Only through blood test you can diagnose the disease.

I: What they ask you at the time of your visit?

R: They ask how you are feeling. I tell them that I am not feeling well. Then they understand why I am not feeling well. Sometime for lack of vitamin body doesn’t feel well. In that case they [doctor] send for test.

I: Does he send you for test every time?

R: No. When they see patient is not curing after taking medicine then they send for test. Where is the fault? Treatment is needed to cure the fault. Suppose I have chest pain but the pain is not going away even after taking medicine then I need to do X-ray to find out the reason of pain. After checking the X-ray they can start the proper treatment. After that the patient gets proper medicine and get cure.

I: Did the doctor write the prescription or give medicine by himself?

R: No, as the doctor sits in the medicine shop, he writes the medicines from there. Doctor doesn’t give medicine by himself.

I: Did he write prescription?

R: Yes.

I: What do you do if you have to buy medicine from medical shop from the compounder?

R: What? [didn’t understand the question]

I: As you told me before that sometimes you take medicine from the compounder. What do you say to him at the time of purchase medicine?

R: I say him that I have fever, runny nose and body pain. In emergency he gives medicine. Then he gives suggestion to visit doctor immediately. What to do in emergency when the doctor is not available? Suppose I have pain at mid night. Whom do you get around 10-11 pm? If there is someone then you go. No option is there at that time. You have to take medicine from the nearby shop to control the pain. This is the matter.

I: In that case do you tell them the name of the medicine or they give you by themselves?

R: He gives and also I know the name of many medicines. So, I take by myself also.

I: Could you please tell me the name of some medicines that you know?

R: For sugar patient there is Triglycomet , for gas Omez D and Omez gel. These are for gas. Zintac, Rantac are also there as normal medicine.

I: For cold, cough, fever

R: We take “halka fulka medicine” [light dose medicine]. Like for cold and cough I take Capazine. But Capazine is now not available in market. I eat Lemonade. After getting relief from body pain we go to the doctor.

I: Do you take antibiotic?

R: Antibiotic means sometimes I take Betnesol.

I: What?

R: Betnesol. I take it by myself.

I: Where do you get the medicine?

R: From medical

I: Means you take the medicine directly from the shop

R: Yes.

I: What is the use of the medicine?

R: That is antibiotic.

I: What is the use?

R: The medicine doesn’t have any reaction. Medicine doesn’t suit to everyone. Suppose for low cost medicine there is no reaction but for the costly medicine there is reaction like itching in the body. Betnesol has no reaction in this case. Betnesol is there, also Avil is there.

I: Why do you take the medicine?

R: When I have severe cold, cough and body pain then I take it by myself.

I: How many tablets do you take at the time of taking the antibiotic?

R: One tablet. 1 or 2, what else.

I: Did the shop keeper of medicine shop explain you the process of taking of antibiotic at the time of buying?

R: No. If I take 1 tabet then what to say. I take 1 tablet. If I take more medicines then he can explain. They told that ‘I don’t have rules to give medicine.’ No shop keeper has the permission to give medicine. Why they take risk. If something happen to any patient then it will be a problem. They give because we are known to them. Otherwise they don’t give medicine to outsiders. If something happen to patient then the shop keeper has to face the problem. They [shop keeper] told directly that go to the doctor first then I will give you medicine.

I: Did the doctor explain you the process of taking antibiotic at the time of prescribing?

R: Those medicines are written in the prescription. This has to take for 5 days or 6 days or 10 days. Doctor writes it in the prescription. Then the medicine shop explains.

I: Did the doctor explain you?

R: No they only write the medicines.

I: Did they write it on the envelop?

R: Yes. They write everything on the paper. It has to take for 2times or this has to take for 5days or 10 days.

I: Did you follow the days of taking medicine as per the prescription?

R: Yes.

I: If you get better before then?

R: Then I left the medicine.

I: How do you use the leftover medicine?

R: I don’t buy full course medicine.

I: You don’t buy.

R: No.

I: For how many days do you buy?

R: For 2-3 days.

I: Why do so?

R: May be I don’t have that much money. If get better then I left the medicine otherwise I have to buy more if not cured.

I: Do you follow it for everyone at your home or only for yourself?

R: No I follow for everyone. We don’t buy full course of medicine.

I: What do you think you have cured and the full course [cut off by respondent]

R: We can’t return rest of the medicine. I can’t get back my money. Suppose for a strip of medicine I have to pay 10 rupees but they don’t take back rest of the medicine. Also for syrup we will take small size then it will finish within 5days. Otherwise for big size we have to pay 200 rupees. We are poor people. How can we spend that much money? That’s why we search for small size syrup. If doctor write for 10 days then in small size the dose will be completed. If he [doctor] writes for 20 days then we will think after taking 10 days of medicine. Syrups are available in 5 days, 10 days and 15 days package.

I: As you told us doctor write for 10 days but you take medicine for 2-3 days. Why do you do that? What do you think before taking medicine?

R: Medicine? [Asking question]

I: Doctor told you to take but why you are taking for 2-3 days?

R: It’s true. Doctor told me that but I have told you money is a big problem. If don’t have money then how can I buy. Suppose the doctor prescribe me medicine for Rs. 250 but I don’t have money at that moment then how can I buy all the medicine. At medical shop they don’t sell loose medicine those are costly from the strip but they give us as we are known to them.

I: Don’t you think that doctor prescribes me for 5 days, but I am only taking for 2-3 days, if any problem occurs due to that?

R: If anything happen then I will buy again. I am not leaving the medicine after taking of 5days. I am buying for 3days as I don’t have money to buy full course. After that I will buy medicine again for 2 days.

I: Did you realize sometimes doctor prescribes too many medicines?

R: No. We don’t go to that doctor who writes many medicines. These types of doctors are also available. IF you need 2 medicines then they write 6 medicines for you. They write to sell the medicine. We don’t consult with that type of doctor.

I: You told us doctor prescribes for 10 days but I take for 2-3 days and stop it to take if cured. [cut off by respondent]

R: If get cured then stop the medicine.

I: At this time don’t you think doctor prescribe me too many medicines unnecessarily?

R: Every medicine has fixed days of course. 5 days or 6 days course. You have to finish the course even after you are cured. If you don’t complete the course then illness will come back again. Do you pay fees again to the doctor?

I: Did someone tell you about to finish the course? How do you know that?

R: Doctor told that I am giving you 10 days of medicine but after taking 5 days I am feeling good but also there is chance that the illness will come back again. He definitely writes it for good reason. He prescribed 10 days of medicine for curing me. Even though I am feeling better after taking 5 days of medicine but it is not good to leave the medicine after 5 days.

I: You buy rest of medicine later.

R: Yes. Like TB patient [Tuberculosis] has to take medicine for 6 months bit if the left taking medicine after 2 months then the illness will come back again after 3-4 months. They do the wrong thing those who don’t take full course of medicine. That’s why distribution of medicine has been stopped from our health center. Now monthly medicines are given from the health centers. Previously people were taking 2 months of medicine at home for TB patient. After 2-3 months patient is coming back again. They are not taking medicine at home. After 3-4 months when the illness is coming back again then they are coming to the doctor. They didn’t complete the course and also they don’t admit their fault.

I: How do you leftover medicines at home?

R: I through them. But we don’t buy that much medicine that we have to through. I am telling about them those who take medicine from health center. They don’t have to pay for the medicine. They [patient] are getting free of cost medicines. They are having it for 1-2 months and then throwing it. Again after 6 months they are coming back with the same illness.

I: Suppose you have fever and you are prescribed medicine for that. Now any other members of your family have fever after some days. Do you same medicine?

R: Which one.

I: The medicines are left with you. Do you give it to others?

R: [interrupting by the respondent’s wife] No. I send them to doctor. Dose of medicine depend upon the age. Suppose I have 10 years son. Why do I give him the medicine of 25 years old person? Why should I give him that medicine? Dose is depend upon the age.

I: If there is someone at your age like your wife?

R: Yes then it is okay. If my wife has same illness like me then I give her my medicine.

I: What do you think before visiting to a doctor? Or what are you hoping from him?

R: People going with the hope that we are going to the doctor to cure illness. People don’t know that I am going to the doctor and I will die there. If there are some severe illness [didn’t complete the sentence]. Listen if someone in your family severely ill but still no one thinks that he [person] will die. Even at the last they take the patient to the doctor with the hope that doctor will cure the patient. Even though they [family of patient] know that the patient will not survive but also they take the patient to the doctor.

I: Where do you prefer to go at the time of illness? Local doctor or [Cut off by respondent]

R: At first we go to the local doctor. When the local doctor can’t cure the illness then we go to outside.

I: Outside means.

R: We go to Asansol.

I: Do you go to the private doctor in Asansol?

R: We visit less number of times in govt. hospital.

I: Why do you visit less in govt. hospital? Also what are facilities you want to be available in govt. hospitals?

R: The govt. hospital is far away from our locality. If we have to spend 100 rupees in travel then we get medicine here in 100 rupees. Why should I go there then? We go to private.

I: Do you prefer to go allopathic doctor or homeopathic doctor?

R: No we visit to allopathic doctor. Also we were used to go to homeopathic doctor in previous days. My sons used to visit homeopathic. Now I became old and left homeopathy.

I: Which one is good, Homeopathy or Allopathy? What do you think?

R: Homeopathy and Allopathy has different rules to take. Allopathy suppresses the illness and homeopathy gets rid of illness from its roots. Any doctor tells me that is it true or false? I was even buying medicine for my family from 35 years. Sometimes for heart, sometimes for liver or sometimes for gas. I have enough knowledge that which medicines work for what diseases.

I: Do you have any medicine business?

R: No. When I visit to the doctors then buy medicine for family.

I: He visits many doctors.

R: No doctors are left in Asansol whom we didn’t visit.

I: Why do you visit doctors frequently?

R: Reason means I go to the first doctor but didn’t cure. Then again go to another doctor and take 2-3 medicines but still didn’t cure. Again go to another doctor and take medicine for 3 months but didn’t improve. Finally if the medicine of next doctor works for me then we go to that doctor every time.

I: Do you think there is any side effect of medicine?

R: That doctor will understand which medicine will suit to what patient. How do we understand?

I: Did they [doctor] explain to you the reason?

R: No. They didn’t. They tell if you want to be cured then take medicine. If we see the medicine is not working for the patient then we go to another doctor.

I: Do you know any rules of taking antibiotic? How to take [Cut off by respondent]

R: No. I don’t know any rules. I follow the doctor’s prescription. They write the medicine according to the illness. Antibiotic used for several diseases. This is not used for any particular illness. If someone have ulcer in liver then doctor prescribe antibiotic because it will cure the disease quickly.

I: Does antibiotic cure the disease quickly?

R: As doctor prescribe me antibiotic is good for my illness.

I: Does antibiotic use for all diseases?

R: No. Not for all diseases. This is used for main diseases. When doctor understand the reason of illness then he prescribes antibiotic.

I: Do you think antibiotic is harmful for human being?

R: It doesn’t harm. Does it used for doing harm? Antibiotic is used to cure illness like people take rice with vegetable. Did they take rice alone? No they can’t take. It is same here. Antibiotic has to take with other medicines. This is the rule.

I: Does antibiotic is useful for common cold, cough? What do you think?

R: If doctor prescribe antibiotic then it is good. Now all the doctors are using antibiotic. It may be low dose or high dose.

I: Do you prefer to use antibiotic? Do you like all the doctors are using antibiotic?

R: Before I heard that if someone takes antibiotic then no other medicine will work on him.

I: Why?

R: I don’t know. I heard it sometime in TV. But now neither the use of antibiotic nor the use of other medicine has been stopped. Antibiotic are using.

I: Do you know about any side effect of antibiotic if used irregularly? Did someone explain you that you have to take full course?

R: No. Antibiotic doesn’t use for long time. It is used for 4-5 days. It doesn’t used for 2-3 months. Antibiotic use depends upon the type of illness. If someone is needed 10 days of antibiotic then they are prescribed for 10 days. If someone need for 20 days then are prescribed for 20 days.

I: Does antibiotic only used for human being or it has use in animals also?

R: Only the veterinary doctors know about that. We know that human beings are using.

I: Do you think people should be aware about the proper use of antibiotic?

R: If there is fault in antibiotic then no doctor will prescribe it. If I see it is harmful for me then I will not take it. Doctors are writing to cure patient not to do any harm. Doctors are taking fees of 400-500 rupees. Why they write medicine to harm me? He [doctor] knows if I write good medicines then the patient will come back again. That’s why they prescribe antibiotic.

I: Does sometime we use medicine wrongly?

R: No. We don’t.

I: Do you think your neighbors and your family members know about antibiotic?

R: Many people are using antibiotic now a days. I see people take antibiotic from medical shops.

I: Do they come with prescription?

R: No.

I: What are methods we have to take to aware more number of people about the proper use of antibiotic?

R: You have to explain people.

I: How?

R: you have to explain like salesman. Have you seen how medical representatives explain the medicines to the doctors? This medicine for liver, this is for gas. They visit to all the clinics. You have to explain like that to the community.

I: What govt. can do?

R: You say [referring to the interviewer]

I: What do you think? What are steps are needed to take?

R: If they organize camp frequently. They have to visit to the rural areas to explain common people. If they don’t go to the community then how can they reach to the community?

I: Does it happen with you that you are taking antibiotic for long time but it is not curing you?

R: No it doesn’t happen to me.
