# Supplementary material for: “Without antibiotics, I cannot treat”: A qualitative study of antibiotic use in Paschim Bardhaman district of West Bengal, India
Source: PLoS One. 2019 Jun 27;14(6):e0219002. doi: 10.1371/journal.pone.0219002 (PMC6597109; doi:10.1371/journal.pone.0219002)
Supplement: S2 File — (ZIP) [file pone.0219002.s002.zip › S2_Transcripts/V4.doc]

**V4**

I: Where are you from? Where is your home?

R: Raniganj.

I: Raniganj? Okay. What is your age?

R: 28.

I: What work do you do?

R: Now I…work in the salon.

I: I see, I see. And who is at your home?

R: In the home, there are my mother, father, and sister.

I: Are you married?

R: Yes I am recently married. Last week, I got married.

I: So at home, illness…meaning, if I say illness, what do you understand?

R: Illness- mentally, there is a little harassment, that is what illness is. A little…what should I say? I don’t have that much knowledge.

I: That’s okay- say whatever you can. Your…

R: Whoever is not well all around. Cannot walk around on their own- he has to do that. Has to get treatment done by doctor.

I: Meaning, if someone falls ill at home, so which illness do you see most commonly at home?

R: Mostly, cold, cough, all this- we get to see mostly.

I: And why does all this happen? Cold, cough…what’s the reason for it?

R: Some are caused by the weather also. Some is because of our own mistake- a little negligence that we have. A little…

I: Such as?

R: If I get a little chest pain, I eat some medicine and I feel better. Then, when the problem…first it’s minor, then it becomes major. It keeps progressing. That’s…

I: So you said you eat “choti moti dawa” – what do you eat in this? What do you take?

R: That- medicine- suppose I ate any cough-cold medicine…I eat any medicine. In the market, you get a lot of medicines, syrups. I eat all that.

I: So where do you bring this from?

R: You get it all from the medical store. You have to say- I have a cough, cold- they give it. Anything.

I: If you have a lot of symptoms, do you go to the doctor?

R: Maximum…for chota mota illness, we don’t go to the doctor. If I see it’s a minor problem, what will we do with the doctor? I don’t go. Maximum percent- 90% of the time, I don’t go. Until something big- if something bigger happens, then we run to the doctor. Then, when we run, we notice that it’s become very late [presented to the doctor too late].

I: When you go to the doctor, which doctor do you go to? Which type of doctor?

R: First of all- now- see- now the situation is such that maximum people- what maximum…actually maximum have gotten okay now…so, the people who see how the government hospital is or not…he doesn’t see that he has chest pain so he should go to a cardiologist, for a kidney problem he should go to a nephrologist, this they don’t see. The patient goes, sees which doctor to go to, and goes and shows it. The doctor saw, understood what problem is happening, he refers accordingly…you have to go to the cardiologist, you have to go to the nephrologist, you have to go to the urologist- he sees but otherwise- they just don’t go like that.

I: But like you have some of your RMP doctors who sit in the community itself. Right, in the local, local-

R: In the locality, yes.

I: The rest are clinics- proper clinic- the MBBS doctors who are there- so which one do you prefer to go to first? Who will you go to first?

R: First, I will- if something happens to me, I will think it’s better to go to a general physician- the ones who are an MBBS. I feel better going to them.

I: So in which situations- RMPs…have you ever been to RMPs?

R: Until now, I have not gone.

I: So in the house also-?

R: [*interrupting*] In the house also, actually my mom had a major problem- a year or 1.5 years ago- a little bit now too- meaning, my mother could not urinate. So like Dr…S was there. Dr. ML I don’t know his name. M! M was his name. He has died now. So first I used to show him only. If anything happened, I used to show him only. [*inaudible*] He had said mummy has a problem- she has problem with urinating- her urine bladder has become small and we may need to do an operation. We were not serious about it at the time. So…kept showing it to him…he used to keep giving medicine. He gave medicine, so it got better, and then we left it [the medicine].

I: Which medicine?

R: Medicine? I don’t know the name.

I: And was this an MBBS doctor? This doctor who was there?

R: Dr. ML…MBBS? I don’t know.

I: Did he sit in your community?

R: No he has a chamber- very big. His own. He has his own chamber. Used to give medicine from there.

I: How was the chamber, meaning was it a big chamber or?

R: The chamber was big. Big chamber- ML’s chamber.

I: I see. And what were his fees?

R: Fees, I think he used to take Rs. 200.

I: How much?

R: 200 rupees.

I: 200 rupees, I see. So if you get a cold and cough- when you buy a medicine from the shop, do you say the name of the medicine or what?

R: Where we know the name of the medicine, I ask for it. Give me Cerzin or give me this thing. And what we don’t know, we say I have a cold, or I have this. They [chemist shops] give it on their own. The medicine.

I: What- what medicine do you name for a cold and cough?

R: Generally, all people don’t know the names of medicines- whoever knows the name, says it, and whoever doesn’t know- what will he do? He’ll say I have a cold, my chest is congested, I have been coughing for two days- they [chemist shops] give it on their own. Take this medicine- it will get better. That’s why everyone doesn’t go to the doctor right away.

I: Do you know anything about antibiotics?

R: No.

I: Have you heard about antibiotics?

R: I have heard about antibiotics.

I: I see. What have you heard?

R: Antibiotics are pills- all that- I don’t have that much knowledge.

I: I see. In your knowledge, have you ever taken antibiotics, or has anyone in your family ever taken it?

R: [*shaking head, indicating he does not know*]

I: You don’t know? Okay. Uh…when you take medicine from the chemist shop, do they ever tell you anything- how much you have to eat, how you have to eat, till when you have to eat?

R: No, once he gives medicine, he says you have to eat it on an empty stomach, or you have to eat it after food- they give it that’s it and we eat it. Like that, generally.

I: Do you ever hear them say this is an antibiotic and that you have to eat this course completely, or anything?

R: Nothing like that.

I: Nothing like that? Okay tell me this- when they give you the medicine, what does the doctor tell you- how do you have to eat it? And how many days do you have to eat it for?

R: Yes, when the doctor- when I used to eat it at the doctor’s, they used to always say- that you have to eat this for 2 months- eat it for 3 months. You have to eat it like this- this is the course, meaning there is a course, right? For every company. [*phone rings*]

I: Yes so you were telling me about the course.

R: Yes, the course. So they used to tell me- that you have to eat it like this, and this.

I: So, do you follow this course?

R: As he says. I had followed it. During mummy’s time, I had followed it, and I had also followed it during papa’s time. I had followed it.

I: I see, I see, I see. What else does the doctor explain?

R: Just that- how you have to eat the medicine, how you should live, what…you have to prevent, what all you should not do- all that.

I: So, what are other possible reasons for illness in society?

R: Don’t know.

I: What do you think, in your view?

R: Negligency- what else? The carelessness we have, that is the reason- for the illness. Not paying attention to cleanliness, not paying attention for a small illness when it occurs…and all this.

I: So when you go to the doctor for any illness, what is your preference- do you normally go to public or private? First the government or private?

R: First, see- if it’s something small, then first private. Then he goes to the government. In the government, all this…staying in line…it’s not always possible. Everyone who has money goes to private. They go to private- whoever has whatever- it depends on the income.

I: We are talking about you though. You- your family- what do you do?

R: It depends on income for everybody.

I: What do you do? If you get something small, where do you prefer to go?

R: I prefer to come here [to the government setup].

I: In the government?

R: In the government.

I: I see. What are your opinions on the services here?

R: Services are good. I am eating medicine- I have been eating medicine for 2-3 weeks here- for 2-3 weeks. My uric acid had increased in my leg- that’s why I had come here. After eating medicine, I had felt relaxed. I had to get a blood test, so I got a blood test- it turned out to be uric acid. So, I have come to get medicine today.

I: To get the blood test done, did you get it done from private or-?

R: I got it down from private. I didn’t get it done here.

I: I see. So did you get the test done and bring the report here?

R: Yes.

I: I see. And how much time does the doctor give you approximately? For one consultation.

R: Here? What should I say about here…5-10 minutes.

I: 5-10 minutes?

R: Yes…they have so many patients here, right? They have to handle everybody. It is not possible, per…meaning, if they gave 15 minutes per person, then it would take too long. They won’t be able to see everyone- this also happens. This is the situation in the government hospital [*laughing*]. Everybody knows.

I: Do the other people in the community where you stay also come here or go elsewhere?

R: Everyone…in our neighbourhood, 30% come over here.

I: Here, means in the government? And the rest go to private?

R: They go to private.

I: So mostly, people go to private…

R: They go to private.

I: Is there anything like a preference to go to government or private based on illness?

R: For choti-moti illnesses, I go to government.

I: Even though the line is long- still the government?

R: [*chuckling*] Choti-moti, like cold and cough, a little infection, some medicine…

I: But why- why do you prefer to go to private for bigger things?

R: I am afraid over here. There is such a long line- it might be too late. Get the treatment done fast. I told you right, when there is negligency, people come here. First if it gets better from medicine here, then it’ll be better- otherwise, there’s the hospital, right! Big hospital- where there is a big doctor…mentality.

I: And the money that it will cost there?

R: Mentality. There’s mentality too right? There’s *lakhs* of money. Mentality. Everyone’s. If it gets better from here, then it’s better- otherwise, we will have to go. We will go.

I: Okay. Okay, I would like to ask one question- what would you like to improve in the government system?

R: Doctor- doctor. There’s a big shortage.

I: I see. Tell me- tell me some more.

R: We need doctors here. There’s so many patients here, and there are 1-2 doctors who sit here. It is not possible to see so many patients. At once.

I: And the way the doctors talk to you?

R: Yes, the way the doctors talk over here- the manner in which they talk- it should not be like that. Anger…convince the patient- you should do like this, do like that. Do this.

I: What is the situation now- how do they explain?

R: What explaining now- did something happen to you? What happened, show me. That’s it. Saw, gave medicine, eat medicine. Then come- after 3 days. It should not be like this. There should be some improvement. It will only improve once there are more doctors.

I: Do you ever feel that the medicine that the doctor gives you is not right- that nothing is happening from it?

R: I have shown them for the first time, I told you right, I have come here for the first time. 2 times- I have shown two times. I ate medicine, and I felt relaxed. I didn’t understand anything else.

I: And when you go to the doctor, what do you think and come? What is in your mind when you come?

R: It’s in my mind that I have to show them. What kind of medicine they give, how they give medicine…first of all, we come here as a test. Let us eat medicine here and see what happens. Do they give good medicine or not? I heard from everyone that the government gives good medicine- they give good medicine, the treatment is going well- that is why we come here. I wanted to come here and see how it is. Everyone is saying that the situation has improved a lot- they give good medicine and the treatment is good. That’s why I came, or I would have never come here. I came to see. I didn’t see anything of the sort. But there is medicine. There is improvement after the medicine.

I: Do you tell the doctor you want to get treated quickly, uh…?

R: Quickly, means, quick- however much time it will take- who says to get it quickly [*snickering*] Those who don’t know that there is a course for medicine, a limit- they will say, why will I say? I know that it takes time for the medicine’s treatment. I will have to eat the medicine for this long. There is a limit.

I: So have you ever not completed a course entirely?

R: I haven’t done that with anyone in our house till now.

I: With no one? Everyone eats it completely?

R: Everyone eats it completely.

I: And get better? Have you ever noticed that- ?

R: Yes. [*interrupting*] Now mummy’s treatment is ongoing with medicine- mummy had a kidney problem. She had a, meaning, there was an infection in the kidney. Her treatment is going on over there, since 6 months.

I: And the medicine that gets left over in the house- what do you do with that?

R: We keep the medicine in a box. There’s a first aid box in the house- we keep it in that. The medicine- we see the medicine- there’s a medicine for headache, so there is a box and we keep all the painkillers in that. And all the medicines- we keep it separately. Keep it in a

I: What do you with the ones you keep?

R: If someone has a need for it by any chance, then we use it. If it hasn’t expired. Else, we throw it.

I: Do you do this on your own or do you ask someone and then do it?

R: Not by asking- if there is some pain, if I have a headache- if there is a lot of pain, then eat one medicine. I eat one medicine…that’s why.

I: And that, I mean, do you do the same thing for cough and cold?

R: The same thing…and then there’s no need to show the doctor. I told you right, we don’t show the doctor in chota-mota cases. If someone has a cold- a cough- we don’t go and show [to the doctor]. Now, if the head is paining- the body is paining- there is tiredness once in a while, right? The body is in pain, the head is in pain from tension. So eat a painkiller and sleep. You’ll be relaxed in the morning [*smiling*].

I: I see, so how often do you take the painkiller?

R: [*pauses*] The painkiller…maximum…sometimes…[*inaudible*]

I: Are there any negative effects of eating medicine? Anything bad about the medicine?

R: Nothing has happened till now.

I: Not happened? I see. Does the doctor ever explain that this medicine may have this side effect? Or by not eating this properly, this may happen? Does the doctor ever explain it to you like this?

R: The place I was taking my mummy to just now- the doctor was giving medicine over there- [*pauses*]. There is an Optineuron injection that comes for my mummy…when he used to give that injection, he used to say, that in this a little…mummy is a little weak…she may feel faint- it’s possible there may be vomiting. He used to say that. Say it’s possible – it will happen like that…but that doesn’t happen, generally- he used to say like that. He used to say- this happens by giving this injection, this happens by giving that injection- he used to say.

I: Mhm mhm. Is he a private doctor or a government doctor?

R: This is a private doctor.

I: Private doctor. I see. And here?

R: No one has said anything like that in the government till now.

I: Never said anything.

R: I have never heard that. I told you right- I’ve only been coming here since two visits. I had come the first time, and today, I am coming for the second time.

I: What was the name of the medicine you said?

R: For mummy? Optineuron. This is an injection.

I: Injection, I see. Do they administer the injection?

R: No no, me. After coming home- they give medicine over there, and then I administer it myself at home. To mummy.

I: Mhm mhm mhm. How far is this place [BPHC] from your home?

R: From here?

I: Where your house is- where you got the injection done, how far is that? The doctor that was there right, who administered the medicine?

R: No I give the medicine to mummy- I administer the injection.

I: You give the injection?

R: Yes, I give it. In Ahmedabad, the doctor used to give it.

I: I see. In Ahmedabad he used to give it…you know how to give an injection?

R: I learned. [*pauses*]

I: Do you ever- the medicine that is there which is finished or which completes its course- what do you do with the leftover pills?

R: The leftover pills- either generally it is kept, or I return it.

I: You return it? They take it back?

R: If they will take it, they take it. They say if it is a complete strip- if it’s a complete strip, then they don’t take it. The one that comes in a complete file- that they don’t take. And the ones that are spare on their own, they take that back. I sell the expensive medicine.

I: I see, I see. And you get money for that. [*Participant nodding yes*] And the few pills that are left over in the house- what happens to them?

R: I throw them away. If the course is over, and they won’t be used, finished.

I: If there is an illness that has come back, have you ever reused that medicine?

R: Never.

I: Never, why?

R: Never. Then I go and show the doctor- what’s wrong in that?

I: Always?

R: Yes, yes- what’s wrong in that? If it’ll be the same medicine or not, whether it will be required or not, why would I take the risk? Especially for mummy and papa, I don’t do that.

I: Have you ever seen that a doctor is giving medicine which is not working and it’s not working for you, for a cold and cough or for anything else?

R: No something like that has not happened.

I: You told me about the medicine. What about what the doctor writes, the prescription that is there- do you save that or that also?

R: That I keep.

I: You keep that? That’s always there?

R: That’s always there.

I: I see. What use is that, afterward you- ?

R: Afterward, if there is any problem by any chance, like- now my treatment is going on in Ahmedabad- the treatment is going on in Ahmedabad- if by the way, there is ever an emergency, I take it outside to any centre outside. The doctor will ask right, where the treatment is going on. There I will have- I will have to show the prescription. That’s why I save it.

I: Does the doctor ask for it?

R: If- what if he asks? By the way treatment is going on- what treatment is going on? So he sees. If I show, it’s better.

I: But has it ever happened that you’ve taken the prescription and gone to- a shop and asked for the same medicine again?

R: [*interrupting confidently*] No. No No No. Never, never.

I: Okay. If the [private] doctor is not available during an emergency, then what do you do?

R: If it’s an emergency, then I have to get admitted in some hospital. [*pause*]. I will have to do something for relief. I don’t give medicine myself- how will I give medicine? I’m not a doctor that I’ll be able to give medicine. If that medicine will work or not, who knows-

I: No, what about that prescription from before-

R: No, no, no. For what reason it happened or did not happen- how can I give medicine? That’s why I did not used to give it. [*pause*]

I: Okay. Would you like to say anything else about the medicine or about the government setup?

R: Government setup, well I told you right, increase the doctors here- it will be better if you increase the number of doctors. It will be really great.

I: Anything else?

R: However many patients come here will also feel relieved. You will have to give some time to each patient, because the way they see the patients- what happened, you have a cough, that’s it, how many days have you had it, this and that, give medicine, eat it, feel better- it doesn’t work like that! You will have to address it, and ask how did it happen, since when did it happen? You will have to ask.

I: And uh, how many medicines does the doctor usually prescribe?

R: 2-3. 3-4 medicines hardly! Here in government…community.

I: Hardly?

R: Yes.

I: And outside, in private?

R: Private means, they see. Whatever the need is, that’s how they give medicine over there.

I: So how many medicines do they prescribe at one time?

R: [*pauses while counting*] 6 medicines. 5 tablets and one injection. For mummy. Per day, 5 tablets and one injection for mummy. And the medicine is not that expensive either! I get very cheap medicine.

I: I see, I see. And earlier you were telling me about MBBS doctors- do you notice any difference between MBBS doctors and RMP doctors? If at all?

R: [*long pause*] No. Normally, no, if there is a difference, I don’t know.

I: But can you identify by looking- which clinic belongs to the RMP and which to the MBBS doctor?

R: No, I cannot tell like that.

I: Okay, okay. Uhh…what do you think- can there be any negative side effects as well of medicines in your opinion?

R: Yes, it happens- why wouldn’t it happen?

I: How- meaning what do you think?

R: There are many side effects of medicines, like if there is a high power medicine that is not suited well, it happens sometimes right. In that type, it happens- feeling faint, vomiting, if the medicine doesn’t suit [the patient].

I: What do you do in this situation, if this happens?

R: I have to show the doctor- right away. I’ll have to call the doctor- how all this is happening- why it is happening. So he will only tell why this is happening.

I: So have you done this before?

R: This had happened to mummy once, like this. Yes, it had happened. So I had shown the doctor here only. The doctor- the doctor had given an injection. [*long pause*]. At that time, mummy had a pain in her stomach. She had pain, that’s why…Rantac and [*inaudible name*]. There’s an injection like that. So he had given two injections, continue, meaning, he had given two consecutively. 10 minutes apart. So he had given those injections, and mummy had some vomiting from that. She was getting dizzy. Why did this happening after giving the injection? Then I called the doctor, and he said no it happens, it’s like a painkiller- headache, pain- it happens- she should rest and it’ll get better. So, I didn’t generally worry- she slept and it got better. He said it’s a painkiller, like a painkiller type- you should not take a lot of it. The patient had a lot of trouble, so I gave it.

I: Okay, what do you think about medicines- is the medicine you get from here better or is the medicine from outside better?

R: This- I cannot tell you, how can I say? Whatever medicine I eat and feel better, that medicine is good!

I: So have you ever noticed a difference when you eat medicine from here and when you eat medicine from outside? How fast you get cured?

R: How fast…I ate medicine from here for the first time. So I ate for the first time- I had just eaten it 3 days ago. He had given medicine to me for 3 days- I ate it today. I ate the last one today- [*inaudible mumbling*]- I felt some relaxation. When I had come on Friday, I wasn’t able to walk.

I: Does anyone at the house say- don’t go there, the medicine there isn’t good- the medicine at the private clinic is better? Like that…?

R: That they say, that government is government. They say the government’s work…it’s come from the government.

I: What do they say? A little, what do they say?

R: Yes, they say- you’ll go stand there for that long- whether they’ll give medicine or not, what they’ll give- pain- how will they treat- will he just leave or go for more treatment- go there and get a test done-

I: And what is a really good hospital here?

R: There are some private hospitals here…go to private, show him, show her- show this doctor, show that doctor- they say like that.

I: What do you think- to give people the right information about medicine and about illnesses- what should be done? How will the message reach them?

R: You should do some cam-campaigning. [*Probe: such as?*] Such as go to many places, like in some neighbourhood, some village- you should go to these places and do some campaigning. About medicines, about all illnesses, you should do some campaigning. You should educate people. Then people will find out that no, you should not limelight [referring to making it a bigger problem than it already is] small illnesses- by limelighting it, it becomes a bigger problem. Until- everyone knows! It’s not like no one knows- everyone knows. But you know- the mentality- they say right? Until something happens, nothing will happen. You have to change yourself. If it doesn’t change, nothing will happen- it will remain like that. Chota-mota illness…people…do like that…wait till it gets bigger, and when it gets bigger, then go here, go there- do this, do that-

I: Anything else that should be done with the community?

R: Campaigning will be better- you will have to raise awareness from house to house. That by doing this, you can do that- go here. All this.

I: Okay. Would you like to say anything else?

R: Something more…no, you say.

I: Okay- thank you so much!
