# Supplementary material for: “Without antibiotics, I cannot treat”: A qualitative study of antibiotic use in Paschim Bardhaman district of West Bengal, India
Source: PLoS One. 2019 Jun 27;14(6):e0219002. doi: 10.1371/journal.pone.0219002 (PMC6597109; doi:10.1371/journal.pone.0219002)
Supplement: S2 File — (ZIP) [file pone.0219002.s002.zip › S2_Transcripts/V7.doc]

**Interview- V7**

Gender: Female

I: What type of diseases usually seen at home?

R: Like loose motion. My husband is taking medicine from Vellore [Hospital]. He has breathing trouble. He is taking medicine from Vellore. Sometimes due to the reaction of that medicine he suffers from constipation. That time he takes medicine from outside like Dalcules. He doesn’t take it always. Sometimes he takes it. I give him dalcules from medical shop. It gives relief and the potty became easy with this. If there is cold and cough then we can’t go always to the specialized doctor. We belong to middle class family and monetary problem is there. We go to local doctor for cold, cough for my family members. I came to him [referring to the ECL pharmacist] today to take medicine. I need the medicine for Gas. We manage like this. If the illness is not curing then we go for specialized doctor.

I: What are the diseases mainly occur at your home?

R: Like during seasonal change, now cold, cough and fever are occurring. Once it occurs before seasonal change and another time after seasonal change. When the winter is going that body pain, cold, cough and fever occurs. We can’t always go the specialized doctor. That time we go to the local doctor. Like now I came here to him [referring to the ECL pharmacist] to take medicine. My husband has cold and cough, also my son has cough. I told him all the symptoms. He gave the medicines. I took from him before. After that the illness decrease little bit. I told him to give me another file [more number of medicines] so that illness will be cured. He told me to wait.

I: Where is the clinic of local doctors?

R: At my locality. We visit them first. When we see the disease is not curing at all then we go the specialized doctor. I have 6 years old granddaughter. I take her to the local doctor, or for my daughter-in-law I visit to him. I also take my husband to the local doctor if the illness increases. From Vellore they told us to check with local doctor if there medicine doesn’t work. In winter sometimes he suffers more from [referring to husband] breathing trouble. He usually takes inhaler that time. But if it doesn’t cure with that then we take him to MD [specialized doctor].

I: How much fees the local doctors charge for treatment?

R: They don’t take fees. They give medicine of 50-60 rupees and it cures the illness. They don’t take fees. The fees are now not less than 300 rupees. It is same for the child and adult.

I: Does the fees for the specialized doctor?

R: Yes. No doctors take the fees less than 300. This is how we manage. When we see the illness is becoming severe and not curing at all like my grand-daughter had cold, cough, chest infection few days before. We went to the local doctor but didn’t cure. Then we went to the specialized doctor. See the diagnosis of a local doctor and a specialized doctor is absolutely different. Like the child specialist, he studied on child. The illness is being cured by him quickly. And for the local doctors it’s just a consolation for us. We hope the illness would cure with the medicines.

I: Why do you go to the local doctor at the beginning and what are the reasons behind that?

R: Reason is that if fever, cold and comes then we go to the local doctor with the hope that it would cure with the medicine. If we see the illness is not curing after taking medicine then we go to the specialized doctor. If the fever sustain for ling time, illness is getting worse, patient is vomiting then we go the specialized doctor. We can’t kill the person. I have severe knee pain. I can’t sit properly. I told the doctor that the pain is not relieving. Doctor told me the pain will remain as I have arthritis. I take pain killer sometimes to reduce the pain. Don’t take all time as they [doctors] said it is harmful for health. That’s why I take medicine sometimes and most of the time takes oil message to reduce the pain. I don’t take the medicine regularly. It is harmful. Suppose I take one medicine today then again after 2 days we take another one. This is how I am managing. We are from the middle class family.

I: Where do you go to visit specialized doctor?

R: Asansol.

I: There is also free health center. Don’t you go there?

R: No. We don’t go there because in our ICDS also there are many medicines available. We have medicines like paracetamol for cold, cough, and deworming medicines. But it doesn’t work. Suppose I give paracetamol from my center to my family member but doesn’t work that much. We are not doctor. We only treat with the help of leaflet that we have in ICDS. If we see the fever is not curing, then we go to the specialized doctor.

I: Means the medicine of health center doesn’t work?

R: No.

I: Do you go to the government hospital?

R: Yes. For knee pain I went to district hospital. Doctor told me to do photo [X-ray]. It will take 3 months to do the x-ray. I asked my doctor can I do it from outside. He said yes. I do it from outside and checked with my doctor. Doctor told me that you have {hare khoy} a kind of arthritis. It will not cure totally. From then I don’t go to the doctor. Now I know the problem. I go to the local doctor and take medicine for 2-3 days. If the doctor told me to take 2 tablets in a day then I take one tablet in every alternative day. I don’t take many tablets.

I: What you do in other illness? Do you take medicine irregularly?

R: No. That time I have to take. I asked the doctor do I have to come after some days. If doctor told me to come again then I go.

I: If you get cured before completing the medicines then what you do?

R: Now I finish the course. I finish course whatever given by doctor. How the illnesses go away if I don’t finish the course? He [doctor] gives the calculate dose. The illness will not go away if I don’t take all the medicines given by doctor. Like now he [ECL pharmacist] gives me medicine. He told me that your cough will not go away if you don’t take antibiotic. I told him to give me antibiotic. My husband and my son have cold and cough. Few days back I took the syrup. It helped to reduce the pain. But still I need more syrup to cure the illness completely. He said take the antibiotic it will cure the illness completely.

I: So you know about antibiotic.

R: Yes.

I: In which cases antibiotics are used?

R: I mainly use antibiotic for cold and cough. My husband has asthma. In that case we have to use antibiotic to cure the illness. Also I give antibiotic to my son and grand-daughter for cold and cough. Without antibiotic cold and cough don’t go away. Doctor also told that without antibiotic it will not cure. Then we have to take antibiotic. They [pharmacy] give liquid antibiotic for child. We have to mix it at home.

I: Did they explain any rules of taking antibiotic?

R: Yes. Doctor tells us also in the pharmacy they explain us also.

I: Do you know the rules?

R: Yes. For the child the powder of the syrup have to mix with Luke warm water and for the elder people they give tablets. They say this has to mix in this way also they explain the procedure of taking.

I: Did they explain you how many days and times you have to take?

R: Yes. They say. Sometimes they give 2 files of syrup [2 bottles]. They gave 2 files for my grand-daughter. I gave her full medicine.

I: If you get cured in the middle of the full course then what you do?

R: If we get cured then we ask to the doctor. We tell them now my cough and fever are cured. Do I continue the medicine? If he thinks it is needed to continue then he writes again otherwise he doesn’t give anything. Then we tell the doctor to prescribe good medicine because my grand-daughter doesn’t want to it. Doctor tells that they will take food by themselves. No need to give medicine. Children don’t want to eat food. So, they will take food when they want.

I: What do you know by good medicine?

R: Means it helps to increase the hunger. Like that I told him [ECL pharmacist] that I have back pain due to gas. So, he prescribes me tablet for gas along with iron tablet. I told him about my leg pain. He told me to do all the x-ray. I have showed him the reports that I have done before for the same problem.

I: What are the reasons is responsible for common daily illnesses?

R: What illness?

I: That we see daily like fever, cough, cold, loose motion.

R: Children take “ulto fulto” [Means unhygienic or street foods] foods that are responsible for loose motion. Sometimes cold is responsible for cough and fever. My grand-daughter takes bath very frequently in a day. She suffers from fever most of the time. Sometimes she use lots of water that is also responsible for cold and cough.

I: Do you know any name of the antibiotic?

R: No. I don’t know.

I: Do you think antibiotic does any harm?

R: Yes. It has. One doctor told me taking many antibiotics is not good for health. He told to take antibiotic as per doctor prescription, don’t take antibiotic by yourself. Don’t take self-medication for same type of illness. I asked the doctor that can I continue the medicines that were given previously. He told that taking more antibiotic is not good for health. Take the medicine according to the prescription. Sometimes we think we have to pay 300 rupees if visit a doctor. It is better to buy the previous prescribed medicine to save the money. I asked once to the doctor that is it harmful if take more antibiotic? He said yes it is harmful. Take medicine according to the need of your body. From then I don’t buy medicine without prescription. I always ask my doctor before buying medicine.

I: What are medical facilities your neighbor likes to access?

R: it depends upon the monetary situation of the people. They take medicines from heath center or RCH [Reproductive Child Health] also they visit to government hospitals. Also those have money they go to private hospital. I told you before that my locality is little poor. But still they visit first to the local doctor and then if doesn’t cure then visit to government hospitals.

I: What do you think or hoping before going to a doctor?

R: I don’t need to pay money there. Doctor is also good. Doctor will give me good suggestion if he can’t treat my illness. Suppose I don’t who will treat my illness affectively. They know about that and they will refer me there. Doctor doesn’t give me bad suggestion. They will give me good suggestions. Like my daughter had appendix. I took her to a good doctor. He referred us to a surgeon for checkup as he suspected it could be appendix. When I went to the surgeon then he told me yes it appendix. Then he did the operation in the hospital.

I: What do you say to the local doctor at the time of your visit?

R: I tell them I have fever, cough and cold. They also told me. There is a doctor in our locality in Kalipahari. He doesn’t want to give medicine to the children. In the market there is also a doctor named Hippi. He also doesn’t want to give medicine to the children. He always tells to go to the pediatrician for child problem. They give medicine to the adult but not the children. They say I am not a doctor. Always go to the big doctor [specialized doctor] for child problem. Then we take our child to the doctor.

I: Did they explain you about the medicine when prescribe?

R: Yes they explain. Take antibiotic in this process. If it doesn’t go away with the medicine then consult with the specialized doctor.

I: Did they prescribe antibiotic?

R: Yes. They give.

I: Did they explain the procedure of taking antibiotic?

R: Yes. They explain us about procedures.

I: Do you know about the course of antibiotic?

R: I told you before that I don’t use antibiotic without prescription. I take antibiotic as per the advice of the doctor.

I: Do you buy medicine of the medicine shop?

R: No. I don’t buy medicine like that. Neither for the adult nor for the children. I only take medicine from the person who has knowledge on medicine. Like, I came today to him [ECL pharmacist] for medicine. He should have knowledge on medicine. I will go to the person who has knowledge on medicine. I am not a doctor. How can I take medicine by myself? I have told you already if I don’t have money then I go to the hospital or visit to ECL clinic. I don’t buy medicine by myself.

I: Did you stock left over medicine at home?

R: No it doesn’t work. I asked one doctor that can I use syrup that is stored at my home. He told me not to use that one. Because after breaking it’s seal the action of the medicine doesn’t persist for long time.

I: Then what you do with the medicines?

R: I through it. May be few amount of medicine left in the bottle. Once in my father’s house over there one Behari person was staying. One day his wife had eye infection and he gave the medicine which was stored at house from long time. Immediately after using the medicine her eyes came out. We asked him what medicine they have used. Did they ask any doctor before using the medicine or not? They used the medicine that was stored at house. Then we sent her immediately to the hospital. Her eyes had gone bad. From then we don’t use leftover medicines also we don’t use it for children too.

I: Do you think your neighbors and other people of the community use medicine in a wrong way?

R: No how they use wrongly. I don’t know about others. But I don’t use irregularly. Even I checked the expiry date of medicine before storing. For some extra tablets I checked the expiry date. If it is under expiry date then I take otherwise I through it.

I: Whom do you visit more, homeopathic or allopathic?

R: Allopathic. I don’t have belief and patience on homeopathy. No one belief on homeopathy at my house also they don’t have patience too. There are many rules for homeopathy. You can’t take onion, sour etc. I can’t maintain so many rules.

I: Do you think antibiotics are only used by human being or for other animals it is also used?

R: May be. They are same as human being. It might be used on them.

I: Do you know anything about it?

R: No I don’t know. As I told you I always visit to the doctor even for my cattle. Some day before my goat was not eating anything. I told my husband to take her to the doctor. Then I put a stick in her mouth and tied it with a rope. Then she started chewing the stick. Now the goat will be fine. Otherwise I have to take her to the doctor.

I: Do you use those types of home remedies for yourself during illness?

R: No I don’t belief on that. We don’t follow that. I don’t belief anything other than medicine. I don’t have belief on occultist. I don’t follow any spiritual activities. That doesn’t work. I don’t belief on that. Some people belief on that. They tell me about those spiritual activities. Is that possible? It is the problem of my health how others can do any harm? I have arthritis. How other people are involved in that? [Smiling]. I don’t belief on that.

I: Do you try any home remedies for common cold and fever?

R: Home remedies means I have paracetamol from ICDS center at home. I only give it to adults. I don’t give it to children. I keep paracetamol for adult. Suppose something happen at night then I give paracetamol to the adult. Then in the morning I visit to the doctor.

I: Did you take anything other than medicine?

R: What would I give other than medicine? I keep ORS for loose motion.

I: What are steps are needed to take to stop irregular use of medicine in the community? What do you think?

R: We explain to the pregnant and anti natal mothers to come to our ICDS center for Iron tablets. It is good for your health. At this time you would have shortage of blood [anemia]. The tablet will help you prevent that. Also we tell don’t take any leftover medicines. They are mostly tribal people. I explain them properly. I referred someone to the ECL clinic as I don’t have the supply of one medicine. Many times I brought patients to the ECL clinic. I came on Friday and Saturday. Also I came today with one patient. This is how we refer patient if we don’t have supply of medicine. We also give them medicine directly if we have supply. We also tell them always cover your child with clean clothes. Always clean the utensils of your baby.

I: What are the steps can be taken to inform most of the people about these?

R: What can I do? [Didn’t understand the question]

I: We are not saying that you have to do. That can be done by anyone. By ourselves or others. How more number of people will get the message? How people will aware more about the issue?

R: You know better. [Referring to the interviewer]

I: What do you think? As you belongs to the grass root level of the community.

R: Doctor comes once in a month in our ICDS center. The children and mother are being checked by the doctor on that day. Also he [doctor] referred the children for vaccination.

I: What are procedures can be taken to inform people about the correct use of medicine? What do you think?

R: Procedures means, we explain people from our ICDS center. Those are written in our book. We explain them according to the book.

I: One of the procedures is ICDS center. What are the other procedures?

R: RCH [Reproductive child health] centers are there. They give the vaccination to the pregnant mothers and anti natal mothers. They also give the medicine to them.

I: If we have to reach to other people except pregnant mothers? Means to the overall community members.

R: [Long silence] you have to set medical camp. The poor people would be benefitted. They will go to you for medical checkup.

I: What is opinion about irregular use of medicine?

R: According to me it should not be used irregularly. Medicine is not a good thing. As it is not good also it is not bad. Medicines helped to alive people. More than 90% people are alive on medicine. We are the common people. How do we understand about the dose of medicine? We should follow the advice of doctor. If we don’t complete the dose then what is the meaning to blame the doctors.

I: Why do you say taking medicine is not good?

R: Means it is not good also it is not bad too.

I: Why it is not good?

R: It is not good to use anything more than need. If I take more food then also it is not good for my health. I can’t digest it. Medicine is also like that. It should be used according to the need. Overusing of medicine is harmful for health.

I: How it can do any harm?

R: It can effect to my kidney or heart.
